# Discovery and Characterization of BAY-805, a Potent and Selective Inhibitor of Ubiquitin-Specific Protease USP21

**DOI:** 10.1021/acs.jmedchem.2c01933

**Published:** 2023-02-20

**Authors:** Fabian Göricke, Victoria Vu, Leanna Smith, Ulrike Scheib, Raphael Böhm, Namik Akkilic, Gerd Wohlfahrt, Jörg Weiske, Ulf Bömer, Krzysztof Brzezinka, Niels Lindner, Philip Lienau, Stefan Gradl, Hartmut Beck, Peter J. Brown, Vijayaratnam Santhakumar, Masoud Vedadi, Dalia Barsyte-Lovejoy, Cheryl H. Arrowsmith, Norbert Schmees, Kirstin Petersen

**Affiliations:** † Research & Development, Pharmaceuticals, Bayer AG, 42096 Wuppertal, Germany; ‡ Research & Development, Pharmaceuticals, Bayer AG, 13353 Berlin, Germany; § Nuvisan Innovation Campus Berlin, 13353 Berlin, Germany; ∥ Structural Genomics Consortium, University of Toronto, Toronto, Ontario M5G 1L7, Canada; ⊥ Department of Pharmacology and Toxicology, University of Toronto, Toronto, Ontario M5S 1A8, Canada

## Abstract

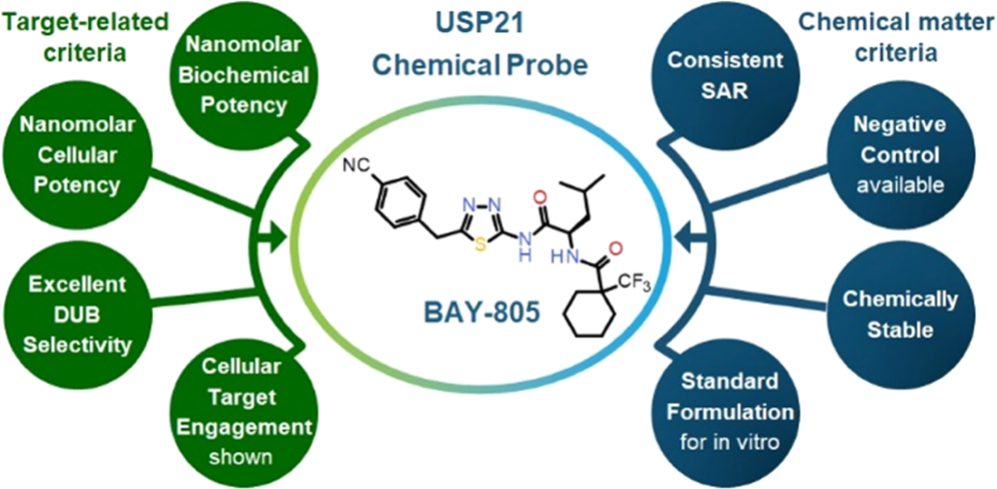

USP21 belongs to the ubiquitin-specific protease (USP) subfamily of deubiquitinating enzymes (DUBs). Due to its relevance in tumor development and growth, USP21 has been reported as a promising novel therapeutic target for cancer treatment. Herein, we present the discovery of the first highly potent and selective USP21 inhibitor. Following high-throughput screening and subsequent structure-based optimization, we identified BAY-805 to be a non-covalent inhibitor with low nanomolar affinity for USP21 and high selectivity over other DUB targets as well as kinases, proteases, and other common off-targets. Furthermore, surface plasmon resonance (SPR) and cellular thermal shift assays (CETSA) demonstrated high-affinity target engagement of BAY-805, resulting in strong NF-κB activation in a cell-based reporter assay. To the best of our knowledge, BAY-805 is the first potent and selective USP21 inhibitor and represents a valuable high-quality in vitro chemical probe to further explore the complex biology of USP21.

## Introduction

Protein homeostasis is highly regulated in cells and has been extensively linked to human disease when dysregulated.^1,2^ Protein ubiquitination is one of the primary mechanisms for the regulation of protein levels in cells, as well as many additional cellular processes, including signal transduction, gene expression, DNA repair, and protein trafficking.^3−6^ Ubiquitination is a highly specific post-translational modification in which the C-terminus of a single ubiquitin (Ub) or a polyubiquitin chain is covalently conjugated to lysine residues of a substrate, catalyzed by an Ub-activating (E1), Ub-conjugating (E2), and Ub-ligating (E3) enzymatic machinery.^7^ This enzymatic cascade can produce mono- or polyubiquitination (poly-Ub) of proteins, the latter with variable poly-Ub chain structures and lysine linkages, which determine different signaling pathways.

Ubiquitination is a reversible modification tightly regulated by deubiquitinating enzymes (DUBs) that catalyze the removal of ubiquitin chains from targeted proteins. While DUBs have been implicated in the regulation of many biological processes and are involved in pathogenic pathways,^4,5^ their function and natural substrates are largely underexplored.^8^ Among seven known and putative DUB families encoded by the human genome, the ubiquitin-specific proteases (USPs) comprise the largest subfamily (50+ proteins) with a conserved protease domain possessing a catalytic cysteine.^9−11^ Recently, USPs have emerged as novel targets for cancer treatment due to their over-expression and activation in various malignant tumors.^12^

USP21 is a prominent member of the USP subfamily playing a role in apoptosis, DNA repair, and signal transduction.^13,14^ USP21 downregulated tumor necrosis factor-α (TNFα)-induced nuclear factor κB (NF-κB) activation through deubiquitination of RIP1^15^ and was reported to deubiquitinate RIG-I as well as STING to negatively regulate antiviral responses.^16−18^ Recent studies indicate the relevance of USP21 in promoting tumor development and growth, including in non-small cell lung cancer,^19^ bladder carcinoma,^20^ gastric cancer,^21^ hepatocellular carcinoma,^22^ basal-like breast cancer,^23^ cervical cancer,^24^ esophageal cancer,^25^ colorectal cancer,^26^ and pancreatic cancer.^27^

To assess the potential of DUBs as therapeutic targets, tool compounds (Cpd) are urgently needed to complement data derived from genetic target validation strategies. However, despite significant efforts, the identification of highly selective and potent small-molecule DUB inhibitors of chemical probe quality has remained a major challenge.^28^ To date, only a few USPs (e.g., USP7, USP1, USP9X, and USP30) have been targeted with high-quality chemical probes.^28−37^ Recently, disulfiram and 6-thioguanine were reported to synergistically inhibit USP2 and USP21 but with low affinity in the micromolar range.^38^ To investigate the cellular function of USP21 and explore its potential as a therapeutic cancer target, we developed the first highly potent and selective inhibitor for USP21. Herein, we present the discovery of BAY-805, a non-covalent, potent, selective, and cell-active inhibitor of the catalytic activity of USP21. This high-quality chemical probe will be a valuable tool to further investigate the complex biological pathways of USP21.

## Results and Discussion

### High-Throughput Screening of USP21 Inhibitors

**Figure 1:**
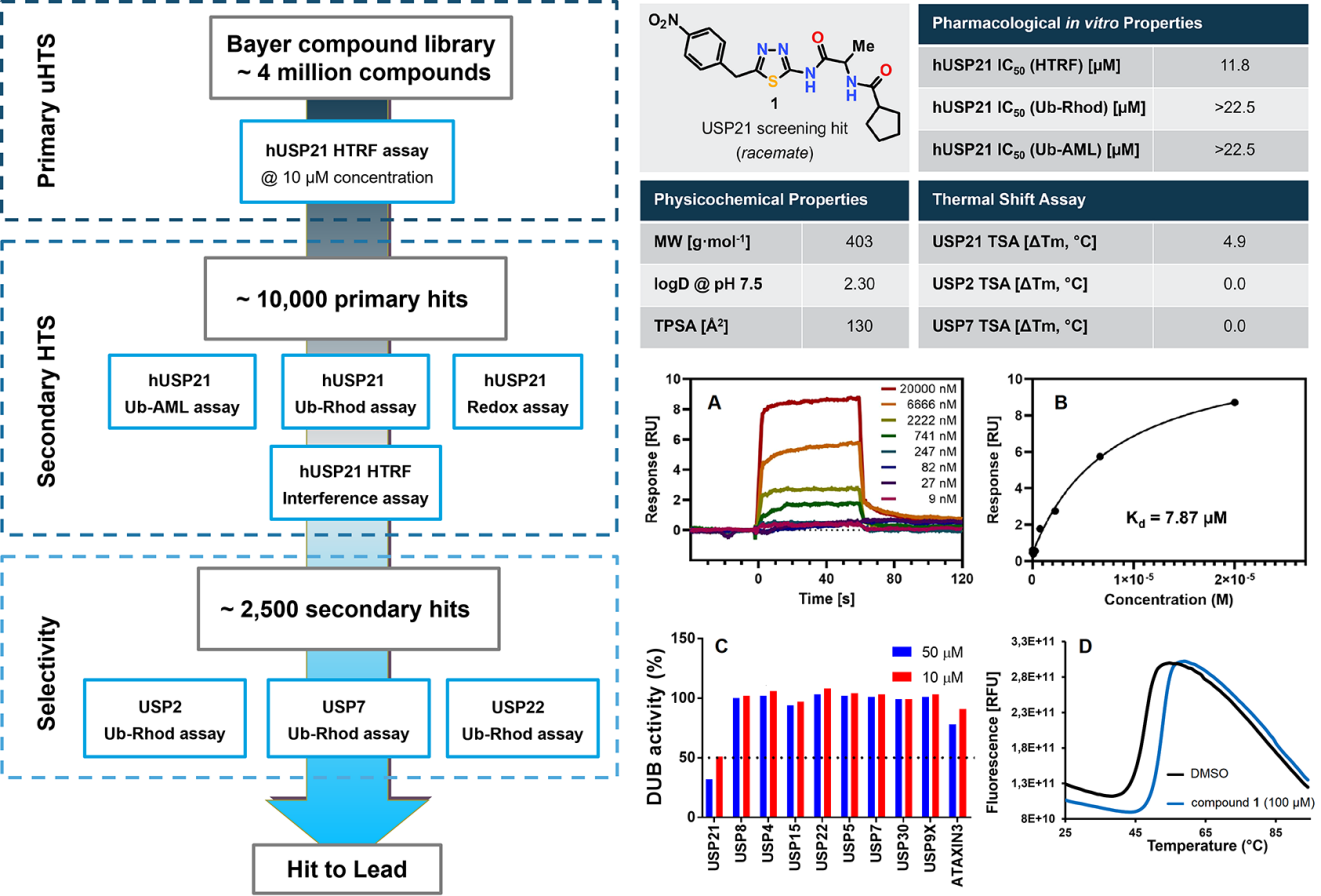
Screening cascade for USP21 inhibitors and characterization of screening hit **1**. (A) Overlaid surface plasmon resonance (SPR) sensorgrams and (B) equilibrium binding of screening hit **1** over immobilized USP21. *K*_d_ values were determined by fitting equilibrium binding data using a one-site specific binding model. (C) Selectivity of **1** against 10 individual USPs. The remaining DUB activity reported at 10 and 50 μM concentrations of **1**. (D) Thermal shift assay (TSA) experiment of USP21 with compound **1** or dimethyl sulfoxide (DMSO) control.

Motivated by the relevance of USP21 as a promising target for cancer treatment, we screened ∼4 million compounds of the Bayer compound library to identify small molecules inhibiting the catalytic activity of USP21. To this end, we developed a USP21 activity assay that is based on the deubiquitination of a STING-derived peptide (a USP21-specific substrate^17,18^) by purified recombinant human full-length USP21 using time-resolved fluorescence resonance energy transfer (TR-FRET) technology (referred to as a homogeneous time-resolved fluorescence (HTRF)-assay, for details see the Supporting Information).^39^ Screening of compounds at a single concentration of 10 μM in the HTRF assay resulted in ∼10,000 primary hits with ≥30% inhibition, which was reconfirmed in triplicate retests. To filter out false-positive fluorescent compounds, hits were confirmed with an orthogonal luminescence-based (Ub-Aminoluciferin substrate, Ub-AML) or another fluorescence-based (Ub-rhodamine 110 substrate, Ub-Rhod) deubiquitination activity assay using Ub-AML^40^ and Ub-Rhod^41^ as generic USP21 substrates. This approach identified more than 2500 confirmed hits after additional verification in interference control and redox-cycling assays. To further characterize our primary hits in terms of DUB selectivity, we tested for inhibition of closely related USP2, 7, and 22 in Ub-rhodamine assays. Most of the screening hits turned out to be non-selective. However, a subsequent hit-to-lead process resulted in the identification of the 1,3,4-thiadiazol derivative **1** as the starting point for further structural optimization (Figure 1).

### Characterization of Screening Hit

Screening hit **1** already exhibited promising inhibitory activity on USP21 with a mean IC_50_ value of 11.8 μM in the HTRF assay. However, the inhibitory activity of **1** on USP21 was not confirmed up to 22.5 μM concentration in either of the orthogonal biochemical assays using Ub-rhodamine or Ub-AML as substrates. Therefore, we developed a binding/stabilization-based thermal shift assay (TSA) as a biophysical validation for on-target activity. Target engagement of screening hit **1** was confirmed via strong stabilization of USP21 of 4.9 °C at 100 μM concentration of compound **1** (Figure 1D). Furthermore, no significant temperature shift was observed for USP2 and USP7 (Figure 1), suggesting no off-target inhibition of structurally similar USPs. Additionally, we employed concentration-dependent SPR measurements to provide further proof of target engagement and to distinguish between specific target-binding and non-specific effects. The SPR experiment confirmed the specific binding to USP21 with a *K*_d_ value of 7.87 μM (Figure 1A,B). To further characterize the screening hit in terms of selectivity, we screened compound **1** at two concentrations against a panel of 10 individual deubiquitinating enzymes (Figure 1C). Besides USP21, none of the other tested DUB targets were inhibited >50% at 10 and 50 μM, which emphasizes the high USP selectivity of compound **1** and coincides with the TSA results for USP2 and USP7. Altogether, these observations prompted us to prioritize this singleton compound for further optimization toward a selective in vitro chemical probe for USP21.

### Structure–Activity Relationship (SAR)

Initially, our primary focus of optimization was the improvement of affinity toward USP21. Due to the lack of structural information, we started a ligand-based SAR exploration by introducing different substituents at the R2 position (Table 1).

**Table 1:**
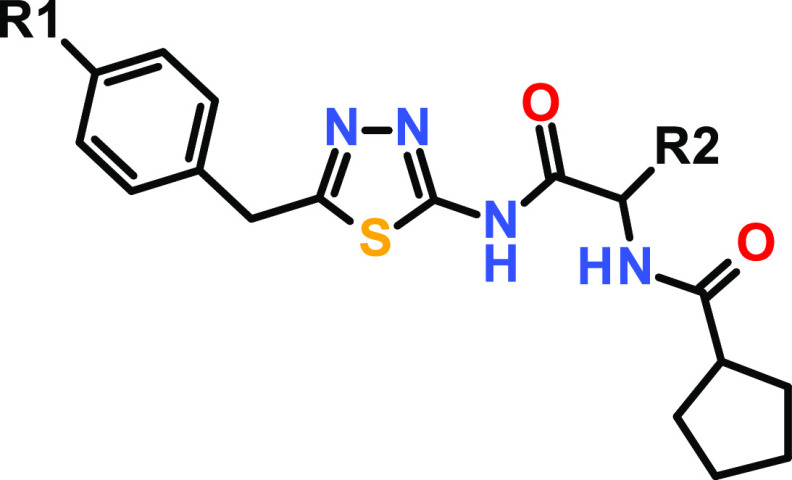

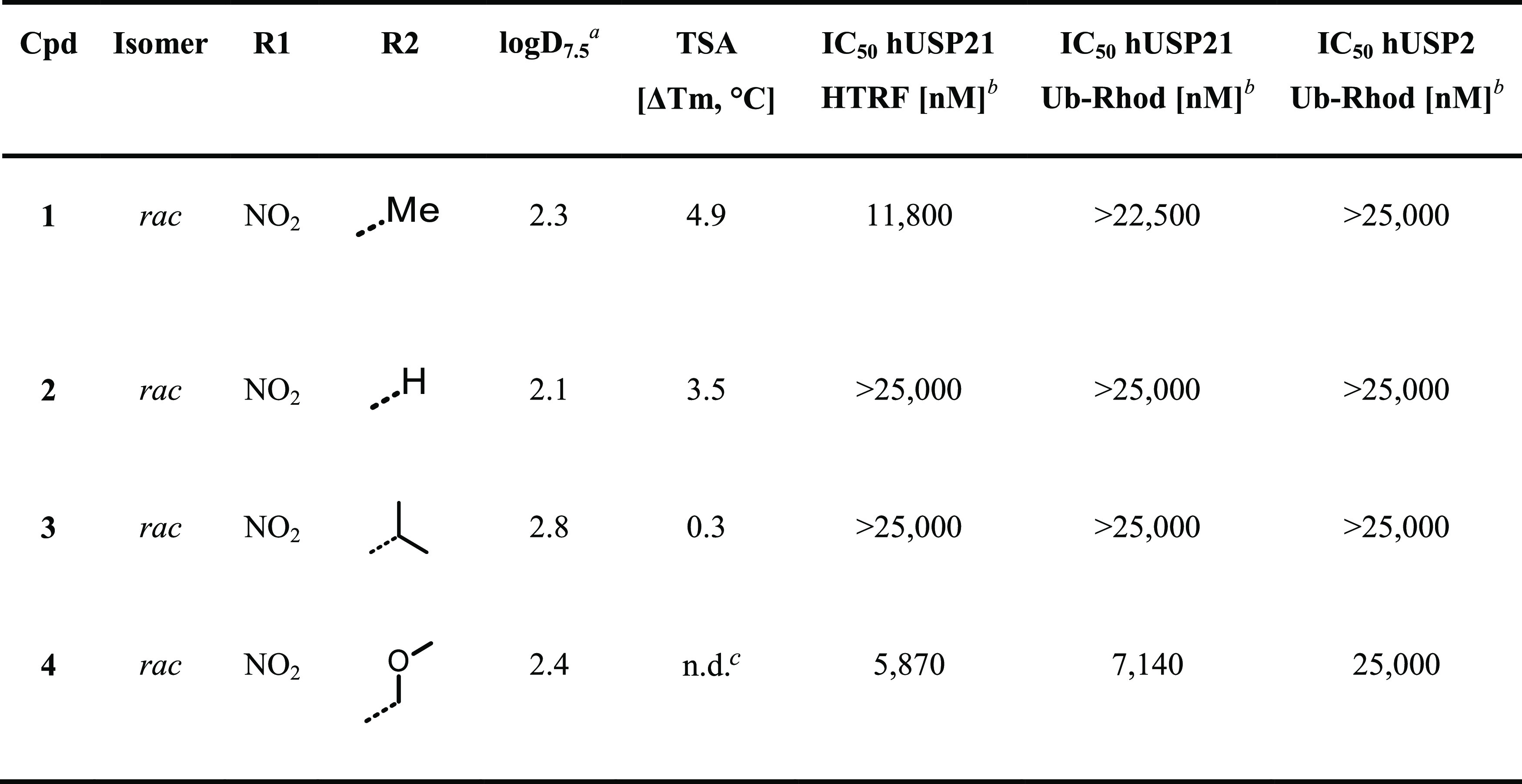
Structure–Activity Relationship (SAR) around Screening hit **1**, Featuring Different R2 Substituents

a log *D*_7.5_ was determined via a high-performance liquid chromatography (HPLC) method.

b IC_50_ values are arithmetic means of multiple measurements.

c n.d. = not determined.

Replacement of the methyl group of screening hit **1** with hydrogen (compound **1**) or an isopropyl substituent (compound **3**) resulted in a complete loss of USP21 potency. Interestingly, compound **2** did not inhibit USP21 activity up to 25 μM in the HTRF and Ub-rhodamine assay but showed a significant TSA stabilization of USP21 of 3.5 °C. However, introducing a methoxymethyl group gave rise to derivative **4** with an improved IC_50_ value of 5870 and 7140 nM for HTRF and Ub-rhodamine assays, respectively. For the first time, we observed biochemical activity in the USP21 Ub-rhodamine assay while still being selective against USP2. This initial finding prompted us to select compound **4** for further optimization on the R3 substituent (Table 2).

**Table 2:**
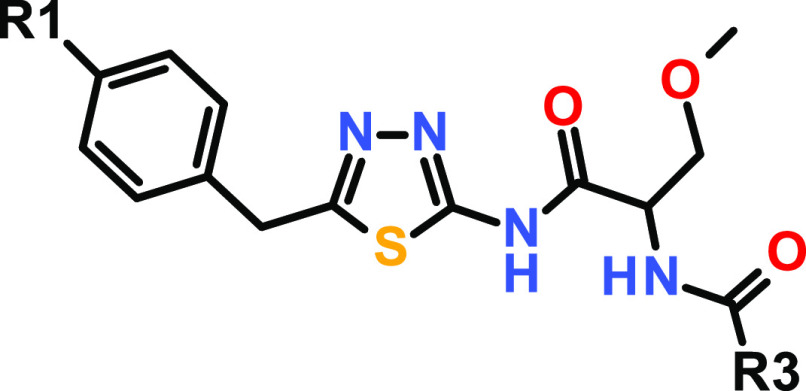

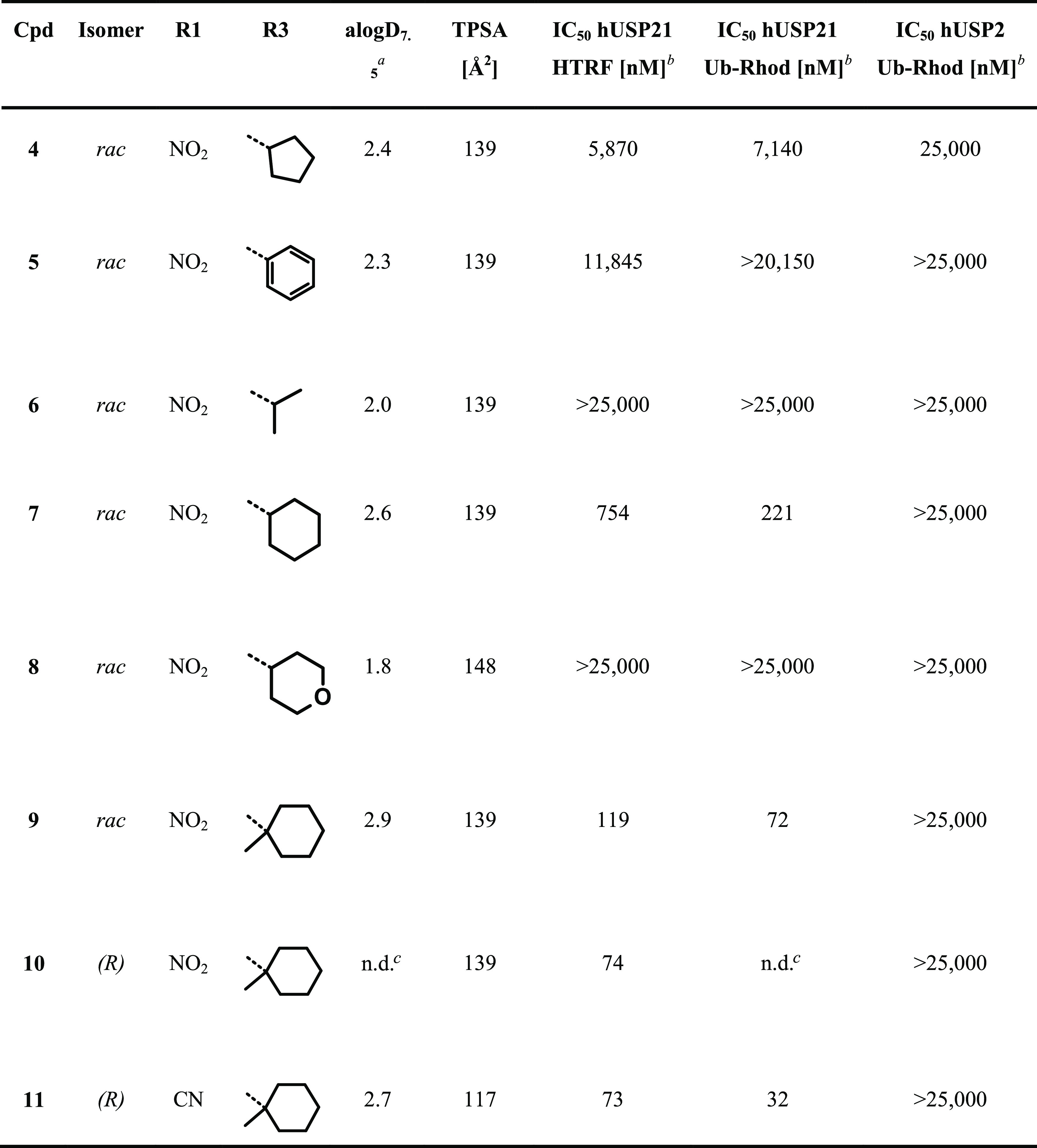
SAR around Compound **4**, Featuring Different R1- and R3-Substituents

a log *D*_7.5_ was determined via an HPLC method.

b IC_50_ values are arithmetic means of multiple measurements.

c n.d. = not determined.

In the next step, we focused on the SAR around the R3 substituent and replaced the lipophilic cyclopentyl group of **4** with a phenyl substituent leading to compound **5** with a potency comparable to screening hit **1**. However, additional substituents on the aromatic ring did not result in any potency improvement (not shown). Interestingly, compound **6** with an isopropyl group proved inactive in both biochemical assays. In contrast, the cyclohexyl derivative **7** showed significantly improved submicromolar potency (∼8-fold for IC_50_ in HTRF) compared to compound **4**. However, introducing an additional oxygen atom, as in compound **8**, resulted again in a complete loss of activity. Likewise, a broad range of both saturated and unsaturated heterocyclic substituents (not shown) significantly diminished biochemical potency. We speculated that the R3 substituent points toward a lipophilic pocket and hydrophobic interactions are required for high in vitro activity. Based on the observed steep SAR, we decided to accelerate compound optimization by parallel synthesis, especially as we could not obtain a co-crystal structure of our inhibitors with USP21 to structurally guide our optimization efforts. Consequently, a broad range of substituents was introduced to the cyclohexyl group of **7**. An additional methyl group at the attachment point of the cyclohexyl residue resulted in a 6-fold improvement of potency for compound **9** with IC_50_ values of 119 and 72 nM for HTRF and Ub-rhodamine, respectively. Stereoselective synthesis revealed the (*R*)-enantiomer **10** as a eutomer (IC_50_ = 74 nM for HTRF). Then, we intended to replace the nitro group to avoid obvious toxicity issues. The isosteric replacement of nitro by a cyano substituent resulted in compound **11** with comparable potency. Additionally, the topological polar surface area (TPSA) dropped to 117 Å^2^, improving the potential for favorable permeability properties. Compound **11** represented the first derivative of screening hit **1**, which fulfilled the probe criteria with respect to the potency of <100 nM in biochemical assays and still high selectivity against USP2 (>780-fold). However, compound **11** displayed only partial USP21 inhibition in the Ub-rhodamine assay, with an efficacy of only <40% (see the Supporting Information).

### Cellular Profiling of Compound **11**

**Figure 2:**
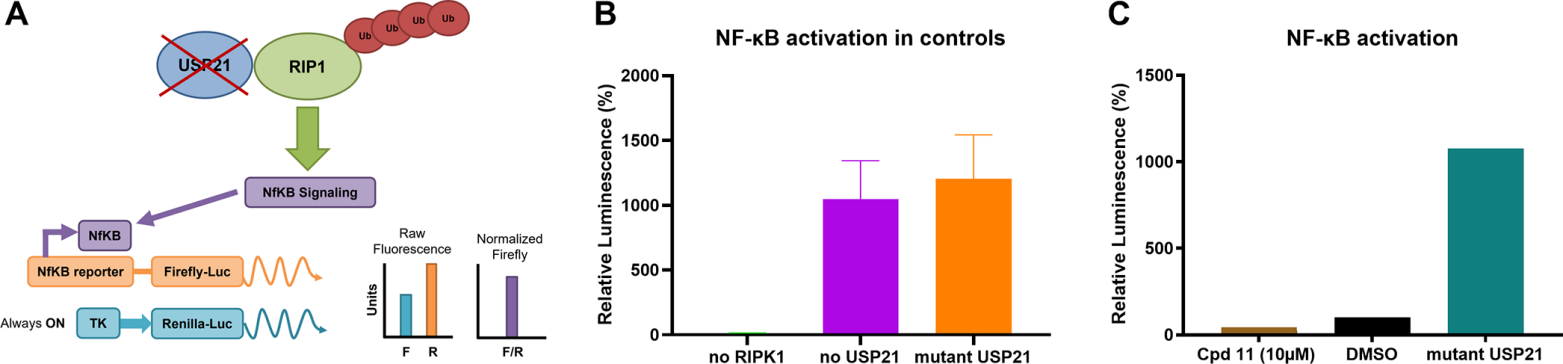
Cellular profiling of compound **11**. (A) Principle of the cell-based NF-κB activation reporter assay. In the absence of, or inhibition of USP21, ubiquitinated RIP1 induces NF-κB activation, and an NF-κB reporter produces a measurable Firefly luciferase signal that can be normalized to the background TK promoter Renilla luciferase signal. (B) NF-κB activation in controls (*n* = 2, technical quadruplicates, mean plotted). (C) The cellular effect of compound **11** on NF-κB activation levels (*n* = 2, technical triplicates, mean and standard error of the mean (SEM) plotted).

Our first optimization campaign resulted in compounds with significantly improved biochemical potency and high selectivity against USP2. As a next step, we investigated the cellular downstream effect of compound **11**. Yang and colleagues reported that USP21 inhibition induces NF-κB activation by preventing deubiquitination of RIP1.^15^ K63 ubiquitinated RIP1 is required for NF-κB activation, and thus USP21-mediated deubiquitination of RIP1 shuts down the NF-κB pathway. To demonstrate the cellular effect of compound **11** on the NF-κB pathway (Figure 2A), we re-established Yang’s cell-based NF-κB dual-luciferase reporter assay. As the catalytic cysteine of USP21 is mainly responsible for the deubiquitinating enzymatic activity,^54–57^ expression of C221R mutant USP21 in cells revealed a strong NF-κB activation and, thereby, increase in the luciferase signal (Figure 2B). Accordingly, the USP21 mutant was implemented as a positive control for effective USP21 inhibition in the cellular system. In contrast, compound **11** showed, unexpectedly, no NF-κB activation up to a concentration of 10 μM (see Figure 2C).

**Figure 3:**
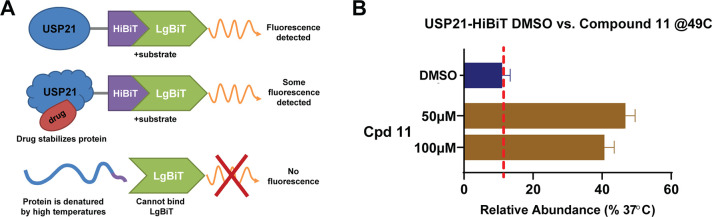
Cellular target engagement for compound **11**. (A). Design of USP21 HiBiT CETSA. (B) Cellular stabilization of C-terminal HiBiT-tagged USP21 with 50 and 100 μM concentrations of compound **11** (*n* = 3, technical quadruplicates, mean and SEM plotted) at 49 °C.

The absence of any significant activity of compound **11** in the cellular NF-κB reporter assay prompted us to establish a cellular thermal shift assay (CETSA) to investigate cell permeability and cellular target engagement (Figure 3A). In particular, we utilized a HiBiT CETSA assay format^42,43^ conducted in intact HEK293T cells. Cells were transfected with USP21 fused to a small proluminescent NanoLuc HiBiT tag, which, when complemented with the Large BiT NanoLuc fragment, produces a quantifiable luminescence signal. Target engagement of HiBiT fusion proteins with compounds stabilizes the proteins in a native cellular environment, allowing them to withstand higher temperatures before denaturing, aggregating, and degrading the luminescence signal. Interestingly, compound **11** induced strong stabilization of USP21 at 49 °C compared to DMSO control (see Figure 3B) which confirmed the binding of **11** to the USP21 protein in the cellular system.

Up to this point, it remained unclear whether limited permeability and/or cellular target engagement contributed to the observed disconnect between biochemical and cellular activity. As compound **11** already showed nanomolar biochemical potency, we speculated that the partial inhibition of USP21 activity (as observed with low efficacy in the Ub-rhodamine assay) did not effectively translate into NF-κB activation in the cell-based reporter assay. Therefore, the next step was to optimize our compounds toward full inhibition of USP21 activity. Thus, improvement of efficacy in both biochemical assays appeared to be our main optimization parameter.

### Optimization of USP21 Inhibition Efficacy

We investigated the SAR of the five-membered heteroaromatic core of compound **11** by varying either of the nitrogen atoms of the 1,3,4-thiadiazole individually (see Table 3). Interestingly, thiazole derivatives **12** and **13** displayed good potency in the HTRF assay but proved inactive in the Ub-rhodamine assay, most likely due to even lower efficacy compared to compound **11**. Additionally, the replacement of the nitrogen atom by *C*-methyl led to increased lipophilicity at pH 7.5 and a significantly lower aqueous solubility. Therefore, we maintained the 1,3,4-thiadiazole motif and turned our attention to the substitution pattern at the cyclohexyl ring.

**Table 3:**
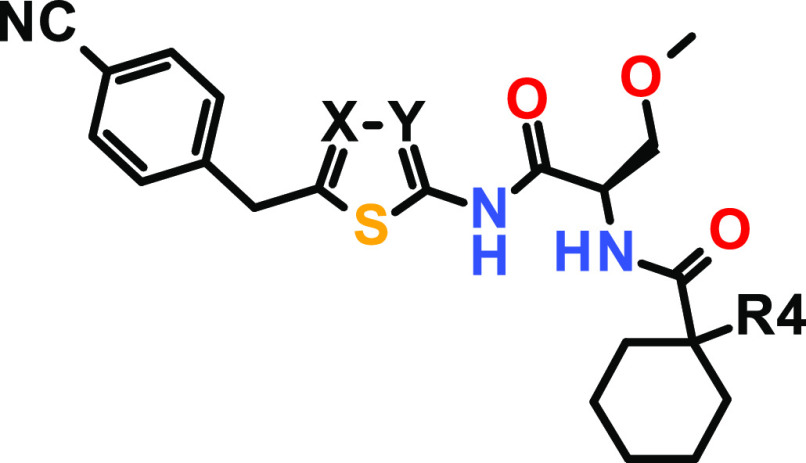
SAR around Compound **11**, Featuring Different Core Variations and R4 Substituents

cpd	X	Y	R4	IC_50_ hUSP21 HTRF (nM)/efficacy (%)^a^	IC_50_ hUSP21 Ub-Rhod (nM)/efficacy (%)^a^	log *D*_7.5_^b^/LLE^c^	solubility PBS pH 6.5 (μM)
**11**	N	N	Me	73/59	32/26	2.69/4.4	126.46
**12**	C(CH_3_)	N	Me	72/50	>25,000/–	3.30/3.8	19.42
**13**	N	C(CH_3_)	Me	65/50	>25,000/–	n.d.^d^/–	n.d.
**14**	N	N	Et	32/76	28/39	2.95/4.5	71.03
**15**	N	N	OMe	21,480/93	>25,000/–	2.59/2.1	181.12
**16**	N	N	CF_2_H	84/80	32/41	2.68/4.4	130.92
**17**	N	N	CF_3_	45/88	36/72	2.95/4.3	10.43

a IC_50_ values are arithmetic means of multiple measurements. Efficacy is referred to as maximal response, i.e., enzyme inhibition, at the highest tested concentration.

b log *D*_7.5_ was determined via an HPLC method.

c Lipophilic ligand efficiency (LLE) was calculated as LLE = pIC_50_ (HTRF) – log *D*_7.5_.

d n.d. = not determined.

In general, lipophilic substituents at the R4-position were well tolerated in terms of potency. Namely, compound **14** with an ethyl group already showed improved potency and efficacy in both biochemical assays, whereas the activity dropped significantly for the methoxy-substituted derivative **15**. Introducing fluorines to the methyl group of **11** provided equipotent compounds (Ub-rhodamine: IC_50_ = 32 nM for **16** and IC_50_ = 36 nM for **17**). The striking difference was the significantly improved efficacy in the Ub-rhodamine assay compared to compound **11**, which was notably observed for compound **17**.

Based on the efficacy improvement of compound **17**, we reinvestigated the SAR for substituent R2 (see Table 4). Introducing a hydroxymethyl group provided compound **18** with a ∼3-fold enhanced potency and significantly improved aqueous solubility, yet, with lower efficacy in both biochemical assays and strong efflux in the Caco-2-assay (efflux ratio (ER) of 14).

**Table 4:**
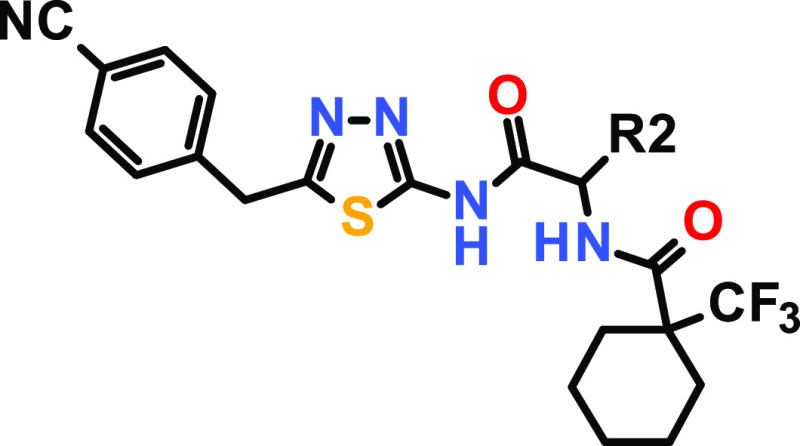

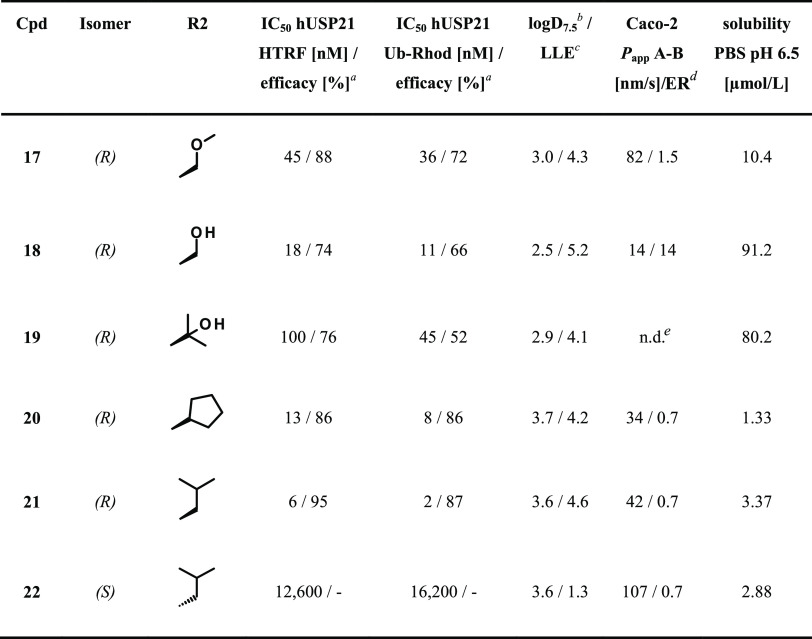
SAR around Compound **17**, Featuring Different R2 Substituents

a IC_50_ values are arithmetic means of multiple measurements. Efficacy is referred to as maximal response, i.e., enzyme inhibition, at the highest tested concentration.

b log *D*_7.5_ was determined via an HPLC method.

c Lipophilic ligand efficiency (LLE) was calculated as LLE = pIC_50_ (HTRF) – log *D*_7.5_.

d ER = efflux ratio.

e n.d. = not determined.

Additional methyl groups, as in compound **19**, resulted in slightly reduced potency. To our surprise, the installation of cyclopentyl and isobutyl groups as R2 substituents yielded compounds **20** and **21** with single-digit nanomolar inhibitory activity for USP21 (based on IC_50_ values in the Ub-rhodamine assay). At the same time, compound **21** displayed almost full USP21 inhibitory efficacy in both biochemical assays and improved Caco-2 cell permeability (*P*_app_ = 42 nm/s, efflux ratio = 0.7), likely resulting from the reduced TPSA of this replacement. The corresponding (*S*)-enantiomer **22** turned out to be 2100-fold (based on HTRF) and 8100-fold (based on Ub-rhodamine) less potent compared to **21** and may therefore serve as a structurally close negative control (see below).

### Cellular Profiling

**Figure 4:**
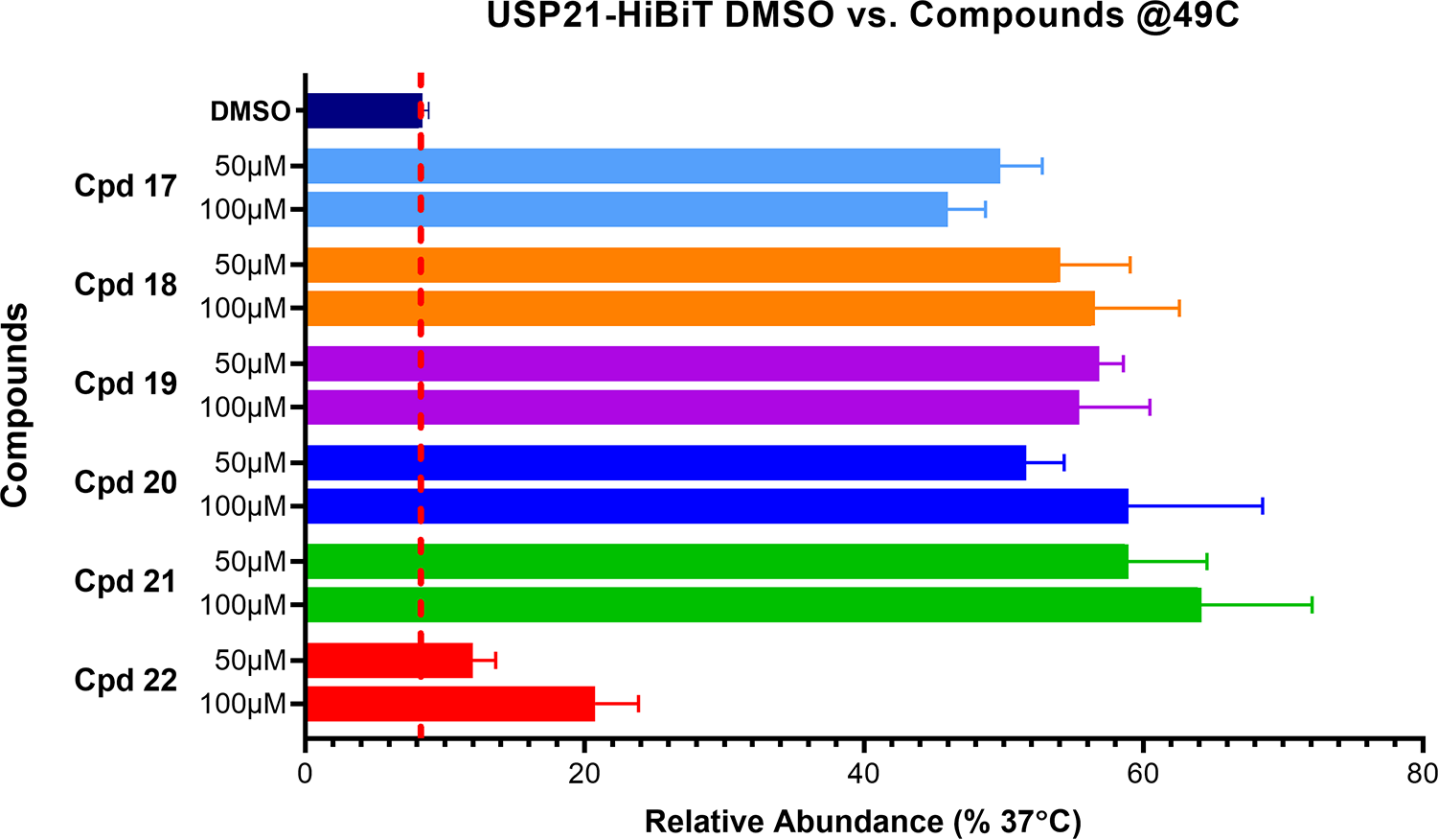
Profiling of compounds **17**–**22** for cellular target engagement in C-terminal HiBiT-tagged USP21 CETSA at 49 °C (*n* = 3, technical quadruplicates, mean and SEM plotted).

Encouraged by the significant potency and efficacy improvements, we profiled compounds of Table 4 in the C-terminally HiBiT-tagged USP21 CETSA (see Figure 4). Strong thermal stabilization of USP21 was observed for compounds **20** and **21**, resulting from optimization toward low nanomolar biochemical activity. Likewise, strong cellular target engagement was confirmed for compound **18**; however, it only showed moderate efficacy in both biochemical assays and low permeability through Caco-2 cells. In contrast, the significantly less potent enantiomer **22** did not substantially affect the thermal stability of USP21 compared to the DMSO control.

**Figure 5:**
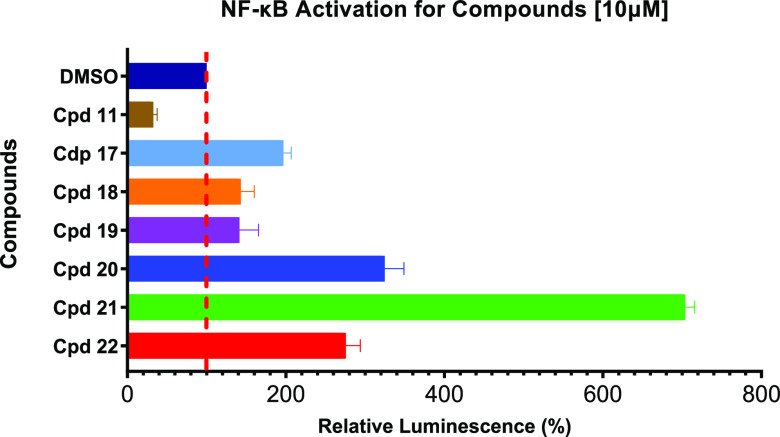
Cellular activity of compounds **11**, **17**–**22** in the NF-κB reporter assay (*n* = 3, technical triplicates, mean and SEM plotted).

Subsequently, we tested compounds **17**–**22** for cellular activity in the NF-κB reporter assay in comparison to compound **11** (see Figure 5). Cellular inhibition of USP21 with compound **20** showed significant NF-κB activation levels; however, when compared to treatment with compound **21**, the NF-κB activation level is less pronounced. For the first time in our hands, the observed cellular target engagement of our USP21 inhibitors translated into cellular activity. As outlined by our results, low nanomolar biochemical potency and high efficacy in both biochemical assays translated into the anticipated activation of the NF-κB pathway. The less active enantiomer **22** mildly increased cellular NF-κB levels at 10 μM compound concentration. However, cellular activity was not observed for compound **18**, although a strong thermal stabilization of USP21 was confirmed in the HiBiT CETSA. The apparent disconnect between cellular target engagement and activity in the NF-κB reporter assay of compound **18** prompted us to investigate ligand-binding to USP21 with SPR experiments.

### Competitive Binding

**Figure 6:**
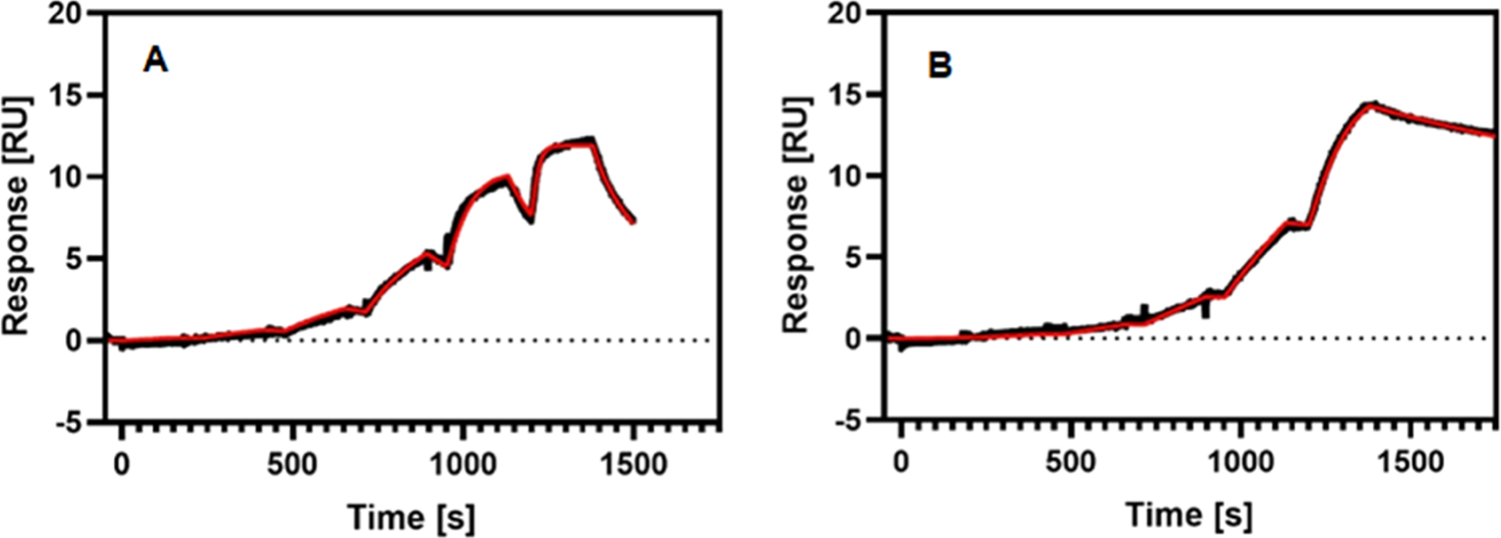
Binding kinetics for **18** and **21** to USP21. The SPR sensorgram for the single-cycle kinetics analysis of **18** (A) and **21** (B) injected in six steps of threefold dilutions over immobilized USP21 measured at 15 °C.

We established an SPR-based competition assay to investigate the competitive binding between wild-type ubiquitin as a physiological substrate of USP21 and compounds **18** and **21**. First, we analyzed the binding kinetics of the two compounds to USP21 in the absence of the ubiquitin substrate. The two competitors, **18** and **21**, despite having similar binding affinities with *K*_d_ values of 5.8 and 2.2 nM, respectively, differ more significantly in their binding kinetics (Figure 6). The binding of compound **21** to USP21 is characterized by a slower on- and off-rate compared to **18**, resulting in an increased half-life for the respective protein–ligand complex.

**Figure 7:**
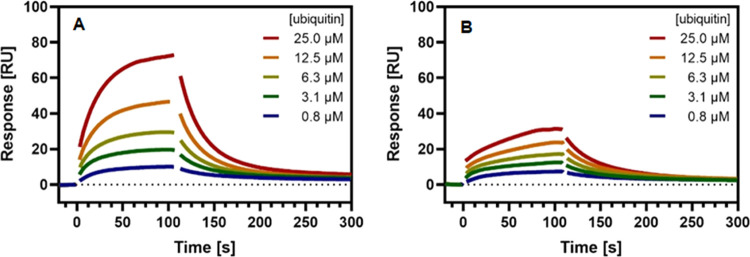
Competitive binding of ubiquitin to USP21 in the presence of compounds **18** and **21**. Overlaid SPR sensorgrams for five serial injections of ubiquitin over immobilized USP21 measured at 15 °C in the presence of 1 μM competitor compounds **18** (A) and **21** (B), respectively.

Subsequently, we studied ubiquitin binding to USP21 in the presence of compounds **18** and **21**. For both compounds, increasing ubiquitin concentration resulted in a gradual displacement of the inhibitors allowing ubiquitin to bind to USP21 (Figure 7). In direct comparison, significantly less ubiquitin binding to USP21 was observed in the presence of **21**. Consequently, compound **21** shows the strongest competition effect and effectively prevents ubiquitin binding. The observed low off-rate for compound **21** probably leads to a strong USP21 occupancy. Both effects are less pronounced for compound **18** and may explain the absence of cellular activity in the NF-κB reporter assay.

### Cellular Potency

**Figure 8:**
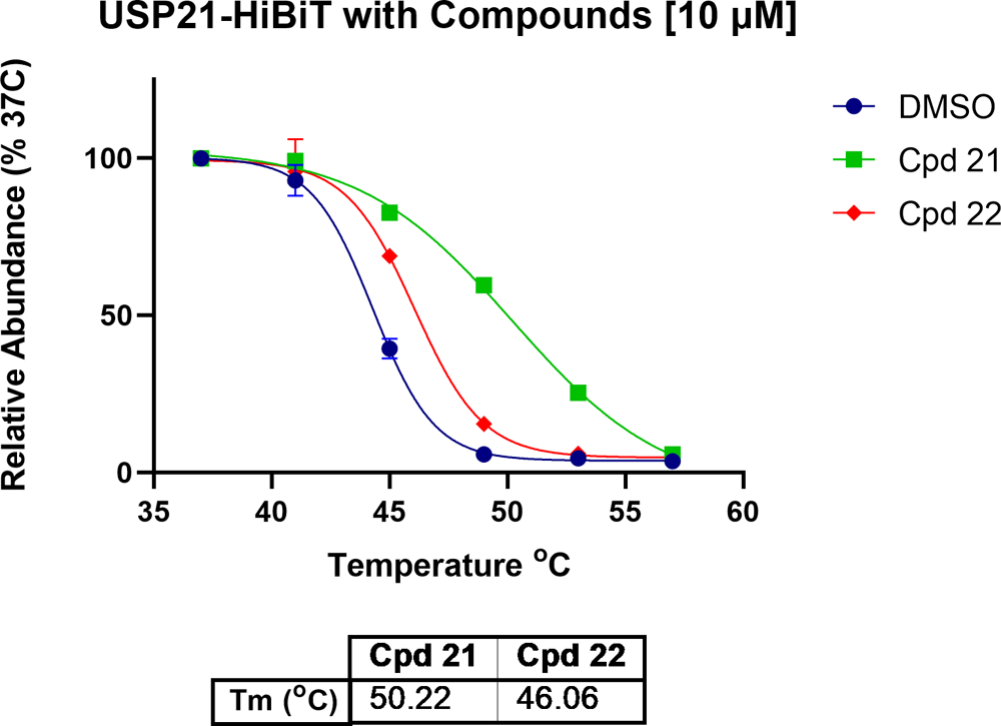
C-terminal HiBiT-tagged USP21 CETSA melting curves in the presence of **21** and **22** at 10 μM compared to the DMSO control using the temperature range 37–57 °C (at 4 °C intervals) (*n* = 3, technical quadruplicates, mean and SEM plotted).

Based on our results, we selected compound **21** as a chemical probe and compound **22** as a corresponding negative control for in-depth cellular target engagement profiling. First, we studied the USP21 interaction of **21** and **22** at different temperatures with the C-terminal HiBiT-tagged USP21 CETSA (Figure 8). In comparison to DMSO control and the less potent enantiomer **22**, compound **21** revealed strong ligand-induced protein stabilization resulting in a substantial thermal shift in the melt curve of USP21 (∼4 °C difference).

**Figure 9:**
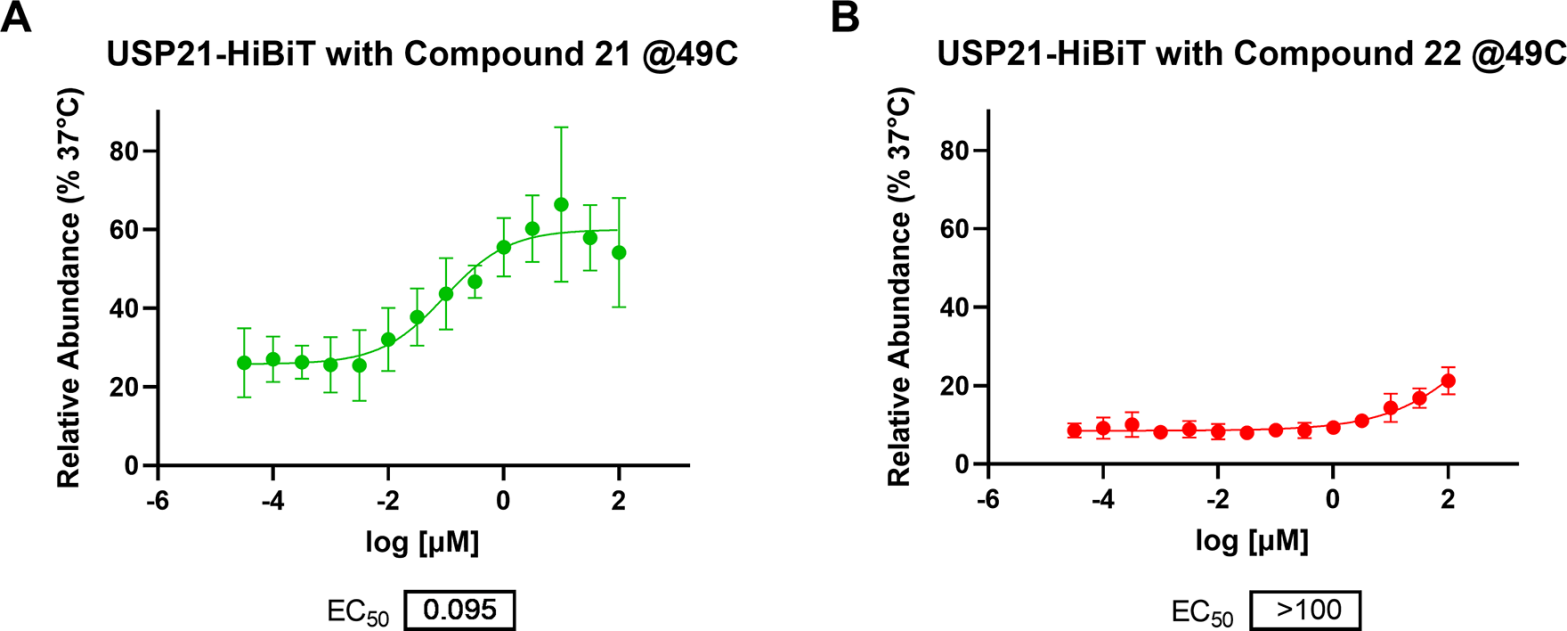
Determination of CETSA EC_50_ potencies for compounds (A) **21** and (B) **22** at 49 °C (*n* = 5, technical quadruplicates, mean and SEM plotted).

For comparative compound profiling, we determined CETSA potencies based on concentration-response experiments resulting in EC_50_ of 95 nM and >100 μM (using compound **21** as the top constraint in the EC_50_ calculation) at 49 °C for compounds **21** and **22**, respectively (Figure 9).

**Figure 10:**
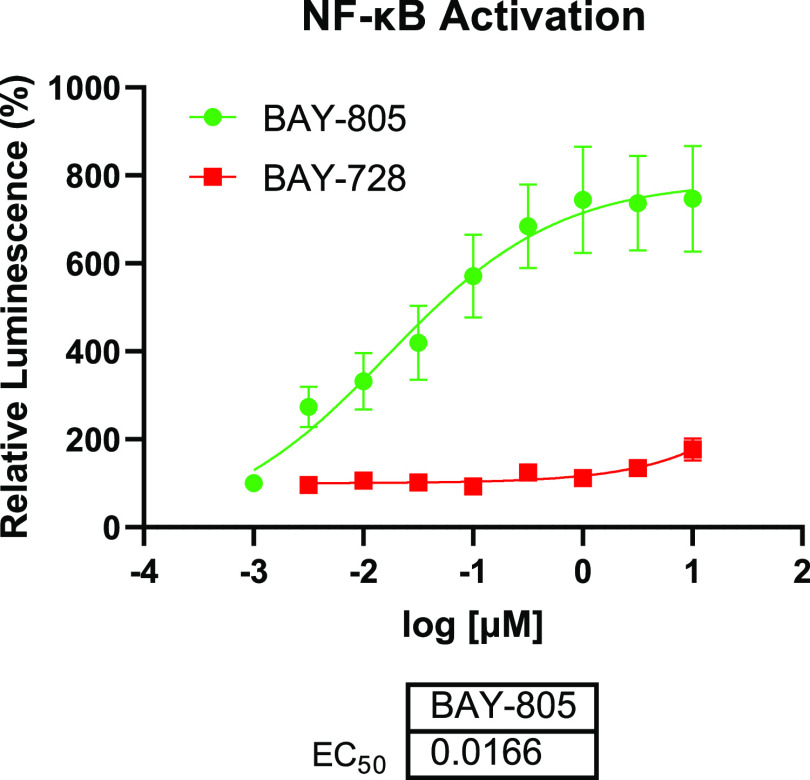
Cellular potency of compounds **21** and **22** in the NF-κB reporter assay (*n* = 5, technical quadruplicates, mean and SEM plotted).

Additionally, we measured the cellular potency for both compounds in the NF-κB reporter assay (Figure 10). As a result, compound **21** induced cellular NF-κB activation with an EC_50_ of 17 nM, whereas enantiomer **22** did not show substantial cellular activity. Therefore, we confirmed the nanomolar activity of compound **21** in both biochemical and cellular assays.

### Antiproliferative Activity

After demonstrating cellular target engagement and the effect on NF-κB, we investigated whether USP21 inhibition affects the cell viability of different human tumor cell lines. However, no antiproliferative effect was observed in Jurkat, Molm-13, A549, MDA-MB-231, and U2OS cell lines up to 30 μM concentration of compounds **21** and **22**, respectively.

### DUB Selectivity Profiling

**Figure 11:**
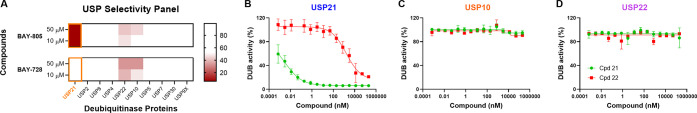
Selectivity profile for **21** and **22**. (A) Selectivity profile for **21** and **22** on a DUB panel comprising 10 individual USPs. USPs are arranged from highest to lowest catalytic domain conservation with USP21 (from left to right, respectively) based on Clague et al.^11^ (B–D) IC_50_ determination of compounds 21 and **22** on USP21, USP10, and USP22 revealed high selectivity of compound **21** for USP21 over other USPs.

As a next step, we investigated the selectivity within the target family and profiled compounds **21** and **22** in the Structural Genomics Consortium (SGC) DUB panel comprising 10 individual USPs (see Figure 11). As a result, **21** displayed a strong inhibitory effect on USP21 activity at 10 and 50 μM and less than 50% inhibition for seven other DUBs of the panel. However, we observed a slight activity on USP10 and USP22 with about 50% residual DUB activity for both enzymes. To determine potential non-specific inhibition more accurately, we measured IC_50_ values for both proteins. In this attempt, inhibition of USP10 and USP22 DUB activity was not observed, whereas IC_50_ data for USP21 were reproducible (see Figure 11B–D). Accordingly, compound **22** did not show a significant effect on the activity of any of the tested DUB targets at 10 and 50 μM concentrations.

**Figure 12:**
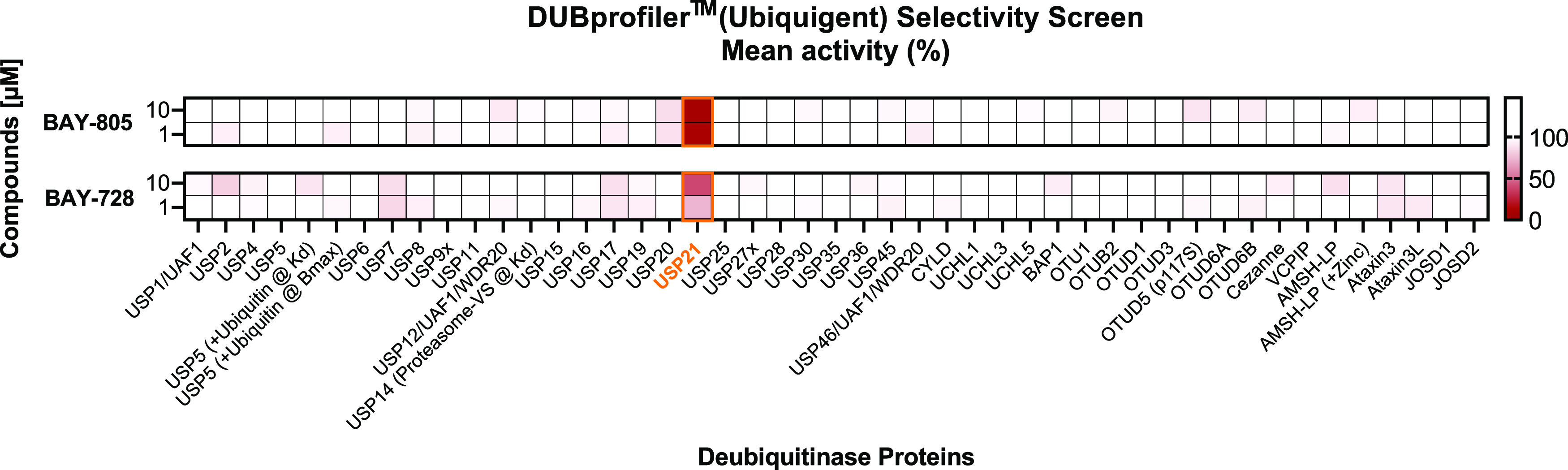
DUB Selectivity Screen in the DUB*profiler* (Ubiquigent) for compounds **21** (BAY-805) and **22** (BAY-728) at 10 and 1 μM concentration, respectively.

In addition, we also screened compounds **21** and **22** in the DUB*profiler* (Ubiquigent) across 44 individual DUB targets in a dose–response assessment at 1 and 10 μM concentrations (see the Supporting Information).^44^ Both compounds showed very low activity (<15% inhibition) across various DUBs, while compound **21** showed 91 and 88% inhibition of USP21 at 10 and 1 μM concentrations, respectively, and thus demonstrates high selectivity for the inhibition of USP21 (Figure 12).

### Off-Target Profiling

We extended the off-target screening and profiled compounds **21** and **22** in a safety screen against 70 individual off-targets, including enzymes, receptors, transporter, ion channels, etc. (see the Supporting Information). Compound **21** was tested at 10 μM concentration with only acetylcholine esterase and adenosine transporters being inhibited with 72% (IC_50_ = 7.61 μM) and 62%, respectively. Likewise, a moderate effect of **22** on acetylcholinesterase was observed with 75% inhibition at 10 μM. In addition, compounds **21** and **22** were also tested in-house against six cysteine proteases (all IC_50_ > 20 μM) and in a kinase selectivity panel (Eurofins/Panlabs) against more than 360 kinases at 10 μM concentration (see the Supporting Information). Minor inhibitory activity of compound **21** was noted for PRAK(h) (58% inhibition, IC_50_ = 8.6 μM) and TrkA(h) (57% inhibition, IC_50_ > 10 μM). Overall, both compounds exhibit favorable selectivity profiles not only within the DUB target family but also against several off-targets.

### Chemical Probe

**Figure 13:**
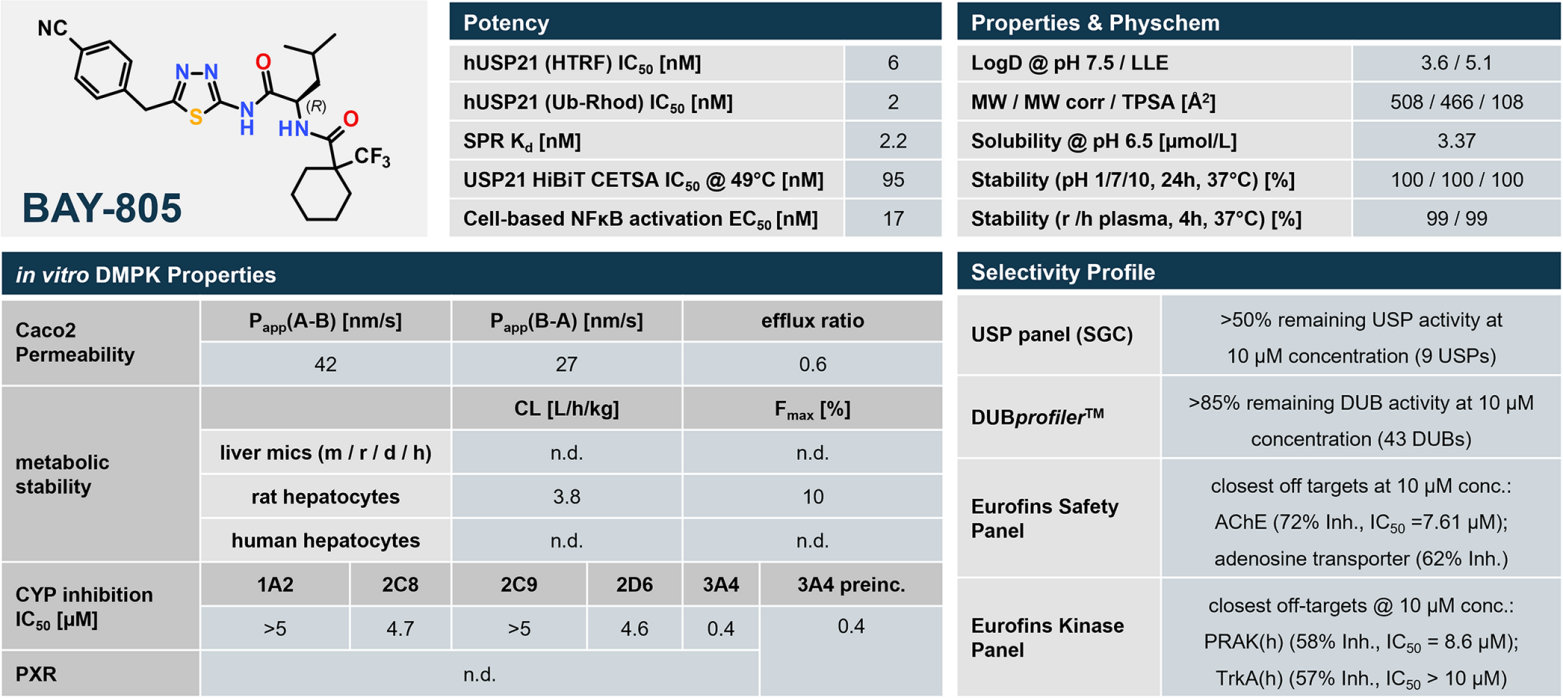
Profile of BAY-805 (**21**), including physicochemical, in vitro pharmacological, and DMPK properties.

Based on the overall pharmacological profile, compound **21** surpasses the stringent target-related criteria for chemical probes^45−48^ typically applied by the SGC^48^ and others (see Figure 13). BAY-805 (**21**) is a highly potent inhibitor with low nanomolar potency in biochemical and cellular assays and shows impressive selectivity not only within the DUB family but also against various off-targets. Binding and cellular target engagement have been demonstrated with high affinity in SPR experiments and strong thermal stabilization in the C-terminally tagged HiBiT USP21 CETSA. Additionally, we provide the less potent enantiomer BAY-728 (**22**) as valuable negative control that was profiled concomitantly to our probe.

### Physicochemical and *In Vitro* DMPK Profiles

Finally, we investigated the solubility and stability of compound **21** in physicochemical and in vitro DMPK assays. Thereby, compound **21** revealed decent physicochemical properties, such as sufficient aqueous solubility for in vitro pharmacological assays in combination with excellent plasma and hydrolytic stability at different pH values (Figure 13). Additionally, compound **21** exhibits moderate lipophilicity with a log *D* value resulting in a good lipophilic ligand efficiency (LLE) of 5.1 based on the IC_50_ in the Ub-rhodamine assay. Along with a favorable TPSA of 108 Å, compound **21** showed moderate permeability in combination with a low efflux ratio (*P*_app_ = 42 nm/s, efflux ratio = 0.6) in the Caco-2 assay. Upon incubation with rat hepatocytes, compound **21** exhibits rather low metabolic stability (CL = 3.8 L/(h kg)). Furthermore, compound **21** showed low micromolar inhibitory activity on cytochrome P450 (CYP) enzymes, in particular on CYP3A4.

### Synthesis of BAY-805

**Scheme 1:**
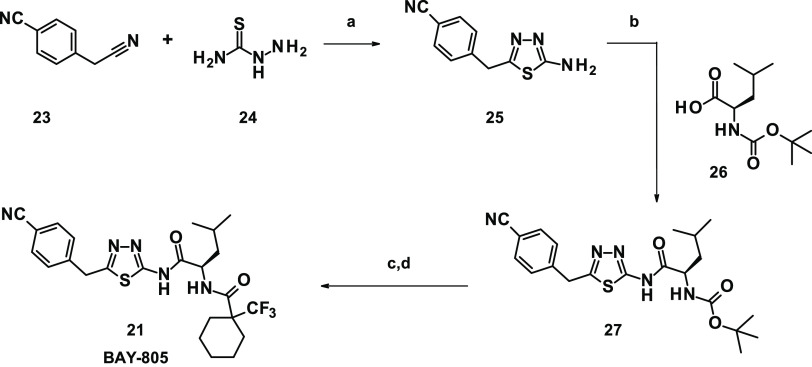
Synthesis of Compound **21** (BAY-805) Reagents and conditions: (a) TFA, room temperature (rt); (b) hexafluorophosphate azabenzotriazole tetramethyl uronium (HATU), *N*,*N*-diisopropylethylamine (DIPEA), dimethylformamide (DMF), rt; (c) 4 N HCl in dioxane, rt; and (d) 1-(trifluoromethyl)cyclohexanecarboxylic, 1-(3-dimethylaminopropyl)-3-ethylcarbodiimide hydrochloride (EDC·HCl), 1-hydroxybenzotriazole monohydrate (HOBt H_2_O), DIPEA, DMF, rt.

The modular structure of our USP21 inhibitors allowed us to develop a straightforward and highly efficient synthesis (Scheme 1). Our short synthetic route comprised only four linear steps and was designed to be easily applicable to parallel synthesis allowing for rapid optimization of screening hit **1**. For the synthesis of compound **21**, building block **25** was prepared on a multigram scale starting from commercially available 4-cyanophenylacetonitrile (**23**), which was reacted with thiosemicarbazide (**24**) in trifluoroacetic acid (TFA). Subsequent coupling with Boc-d-leucine (**26**) provided intermediate **27**. Boc-deprotection with 4 N HCl in dioxane followed by amide coupling with 1-(trifluoromethyl)-cyclohexanecarboxylic acid gave rise to compound **21** (BAY-805).

In analogy, the negative control compound **22** was synthesized using Boc-l-leucine in the first amide coupling step. Detailed descriptions for the synthesis of screening hit **1** and derivatives **2**–**22** can be found in the Experimental Section and Supporting Information.

## Conclusions

In our pursuit of new anticancer drugs, we screened ∼4 million compounds of the Bayer compound library on USP21 using a biochemical HTRF assay and identified potent and selective USP21 inhibitors following optimization of screening hit **1** via parallel synthesis. Cellular target engagement for key compounds was confirmed with CETSA. However, only compounds displaying high potency and efficacy in both the HTRF and Ub-rhodamine biochemical assays were able to demonstrate a USP21 inhibitory effect in a cellular NF-κB reporter assay. Consequently, we directed our chemistry optimization efforts toward improving potency and efficacy in the USP21 Ub-rhodamine assay, culminating in the identification of BAY-805 (**21**), a single-digit nanomolar inhibitor of USP21 with high selectivity versus DUB family members and a variety of off-targets. NF-κB levels were strongly increased in the presence of BAY-805 (**21**) to an extent comparable to those of a USP21 catalytic-dead mutant. Importantly, using BAY-805, we were able to demonstrate, for the first time, the relevance of USP21 chemical inhibition for the enhancement of the NF-κB pathway. To the best of our knowledge, BAY-805 (**21**) constitutes the first highly potent USP21 chemical probe with an extensively characterized on- and off-target profile. BAY-805 (**21**) surpasses the stringent criteria for chemical probes typically applied by the SGC^48^ and others, including the availability of its less active enantiomer BAY-728 (**22**) as a corresponding negative control.^46,49,50^ Both compounds will be available upon request^51^ and are recommended to be used in cellular studies to interrogate the function of USP21 in normal physiology and multiple diseases, from viral infections to cancers.^52^

## Experimental Section

### Synthetic Procedures

#### General Methods and Materials

Commercially available reagents were used as provided by the supplier without further purification. Solvents for synthesis, extraction, and chromatography were of reagent grade and used as received. Moisture-sensitive reactions were carried out under an atmosphere of argon, and anhydrous solvents were used as provided by the commercial supplier. Reaction progress was monitored by thin-layer chromatography (TLC) and/or LC/MS with an Agilent MS Quad 6150 instrument and Agilent 1290 HPLC; column: Waters Acquity UPLC HSST3 1.8 μm 50 × 2.1 mm^2^; eluent A: 1 L water + 0.25 mL 99% formic acid, eluent B: 1 L acetonitrile + 0.25 mL 99% formic acid; gradient: 0.0 min 90% A → 0.3 min 90% A → 1.7 min 5% A → 3.0 min 5% A; oven: 50 °C; flow: 1.20 mL/min; and UV detection: 205–305 nm. Crude products were purified using preparative reverse-phase HPLC methodology with UV detection or flash chromatography using an Isolera chromatography system with prepacked Biotage silica cartridges. The fractions obtained were concentrated in vacuo to remove organic volatiles. Unless otherwise indicated, all compounds have greater than 95% purity as determined by LC-MS. ^1^H NMR and ^13^C NMR spectra were recorded in solvents indicated below at rt with Bruker Avance spectrometers operating at 400, 500, or 600 MHz for ^1^H NMR and at 126 MHz for ^13^C NMR. Chemical shifts are reported in ppm relative to tetramethylsilane (TMS) as an internal standard. The descriptions of the coupling patterns of ^1^H NMR signals are based on the optical appearance of the signals and do not necessarily reflect the physically correct interpretation. In general, the chemical shift information refers to the center of the signal. In the case of multiplets, intervals are given. Spin multiplicities are reported as s = singlet, br s = broad singlet, d = doublet, dd = doublet of doublets, t = triplet, q = quartet, and m = multiplet. High-resolution mass spectra (electrospray ionization, ESI) were obtained via UHPLC-MS. Method A: system MS: Thermo Scientific FT-MS; system UHPLC+: Thermo Scientific UltiMate 3000; column: Waters, HSST3, 2.1 × 75 mm^2^, C18 1.8 μm; eluent A: 1 L water + 0.01% formic acid; eluent B: 1 L acetonitrile + 0.01% formic acid; gradient: 0.0 min 10% B → 2.5 min 95% B → 3.5 min 95% B; oven: 50 °C; flow: 0.90 mL/min; and UV detection: 210 nm/optimum integration path 210–300 nm. Method B: system MS: Thermo Scientific FT-MS, system UHPLC+: Thermo Scientific Vanquish; column: Waters, HSST3, 2.1 × 75 mm^2^, C18 1.8 μm; eluent A: 1 L water + 0.01% formic acid; eluent B: 1 L acetonitrile + 0.01% formic acid; gradient: 0.0 min 10% B → 2.5 min 95% B → 3.5 min 95% B; oven: 50 °C; flow: 0.90 mL/min; and UV detection: 210 nm. Preparative HPLC was carried out with a Waters Prep LC/MS System; column: Phenomenex Kinetex C18 5 μm 100 × 30 mm^2^; eluent A: water, eluent B: acetonitrile, eluent C: 2% formic acid in water, eluent D: acetonitrile/water (80/20 vol %); flow: 80 mL/min, room temperature, UV detection: 200–400 nm, At-Column Injection (total injection); gradient: eluent A: 0 → 2 min 55 mL, eluent B: 0 → 2 min 15 mL, eluent A: 2 → 10 min with 55 mL → 31 mL and eluent B: 15 mL → 39 mL, 10 → 12 min 0 mL eluent A and 70 mL eluent B. Eluent C and eluent D with a constant flow of 5 mL/min each. Optical rotations were recorded on an Anton Polarimeter MCP200 with parameters (solvent, wavelength, temperature) as indicated.

### Experimental Procedures

The synthesis of the compound of BAY-805 (**21**) can be found in this section. For all other compounds, see the Supporting Information.

#### Synthesis of BAY-805 (**21**)

##### 4-[(5-Amino-1,3,4-thiadiazol-2-yl)methyl]benzonitrile (**25**)

4-(Cyanomethyl)benzonitrile (compound **23**, 5.00 g, 35.2 mmol, 1.0 equiv) and thiosemicarbazide (compound **24**, 6.41 g, 70.3 mmol, 2.0 equiv) were dissolved in TFA (30 mL) and stirred at room temperature for 3 days. The reaction mixture was concentrated, poured on ice water, and basified with aq. NH_4_OH. The precipitate was filtered and purified in three portions by column chromatography (silica gel, eluent: ethyl acetate/methanol 99:1–1:13) to yield the desired compound **25** (1.37 g, 100% purity, 18% yield). ^1^H NMR (600 MHz, DMSO-*d*_6_) δ ppm 4.27 (s, 2H), 7.09 (s, 2H), 7.49 (d, *J* = 8.22 Hz, 2H), 7.81 (d, *J* = 8.41 Hz, 2H). LC/MS (method A): *R*_t_ = 0.91 min; HRMS: *m*/*z* [M + H]^+^ calcd for C_10_H_9_N_4_S: 217.0547, found 217.0543.

##### *tert*-Butyl *N*-[(1*R*)-1-[[5-[(4-Cyanophenyl)methyl]-1,3,4-thiadiazol-2-yl]carbamoyl]-3-methylbutyl]carbamate (**27**)

Boc-d-leucine (compound **26**, 128 mg, 554 μmol, 1.2 equiv) and HATU (211 mg, 554 μmol, 1.2 equiv) were dissolved in DMF (1.1 mL), and DIPEA (120 mg, 161 μL, 925 μmol, 2.0 equiv) was added. After stirring for 15 min at room temperature, 4-[(5-amino-1,3,4-thiadiazol-2-yl)methyl]benzonitrile (compound **25**, 100 mg, 462 μmol, 1.0 equiv) was added, and the reaction mixture was stirred at room temperature overnight. A few drops of water were added, and the reaction mixture was purified using preparative HPLC to yield the desired compound **27** (132 mg, 100% purity, 67% yield). ^1^H NMR (600 MHz, DMSO-*d*_6_) δ ppm 0.87 (t, *J* = 6.14 Hz, 6H), 1.36 (s, 9H), 1.37–1.42 (m, 1H), 1.47–1.56 (m, 1H), 1.59–1.70 (m, 1H), 4.18–4.29 (m, 1H), 4.48 (s, 2H), 7.17–7.27 (m, 1H), 7.55 (d, *J* = 8.07 Hz, 2H), 7.82 (d, *J* = 8.07 Hz, 2H), 12.63 (s, 1H). LC/MS (method A): *R*_t_ = 1.92 min; HRMS: *m*/*z* [M + H]^+^ calcd for C_21_H_28_N_5_O_3_S: 430.1912, found 430.1908.

##### *N*-[(1*R*)-1-[[5-[(4-Cyanophenyl)methyl]-1,3,4-thiadiazol-2-yl]carbamoyl]-3-methyl-butyl]-1-(trifluoromethyl)cyclohexanecarboxamide (**21**)

*Step 1*: *tert*-Butyl *N*-[(1*R*)-1-[[5-[(4-cyanophenyl)methyl]-1,3,4-thiadiazol-2-yl]carbamoyl]-3-methylbutyl]carbamate (compound **27**, 125 mg, 291 μmol, 1.0 equiv) was stirred in 4 N HCl in dioxane (0.73 mL, 2.91 mmol, 10.0 equiv) for 2 h at room temperature. The reaction mixture was concentrated and dried in vacuo to yield (2*R*)-2-amino-*N*-[5-[(4-cyanophenyl)methyl]-1,3,4-thiadiazol-2-yl]-4-methyl-pentanamide dihydrochloride (109 mg, 100% purity, 93% yield). LC/MS (method B): *R*_t_ = 0.92 min; HRMS: *m*/*z* [M + H – 2HCl]^+^ calcd for C_16_H_20_N_5_OS: 330.1388, found 330.1383.

*Step 2*: 1-(Trifluoromethyl)cyclohexanecarboxylic acid (65.8 mg, 336 μmol, 1.5 equiv), EDC·HCl (64.3 mg, 336 μmol, 1.5 equiv), and HOBt hydrate (51.4 mg, 336 μmol, 1.5 equiv) were dissolved in DMF (1.00 mL). DIPEA (145 mg, 195 μL, 1.12 mmol, 5.0 equiv) and (2*R*)-2-amino-*N*-[5-[(4-cyano-phenyl)methyl]-1,3,4-thiadiazol-2-yl]-4-methyl-pentanamide dihydrochloride (90.0 mg, 224 μmol, 1.0 equiv) were added, and the reaction mixture was stirred for 3 days at room temperature. The crude mixture was purified using preparative HPLC to yield the desired compound **21** (41.5 mg, 100% purity, 37% yield). ^1^H NMR (600 MHz, DMSO-*d*_6_) δ ppm 0.84 (d, *J* = 6.65 Hz, 3H), 0.89 (d, *J* = 6.65 Hz, 3H), 1.09–1.19 (m, 2H), 1.27–1.49 (m, 4H), 1.52–1.71 (m, 4H), 1.75–1.82 (m, 1H), 2.32–2.39 (m, 1H), 2.45–2.49 (m, 1H), 4.47 (s, 2H), 4.61–4.70 (m, 1H), 7.55 (d, *J* = 8.02 Hz, 2H), 7.81 (d, *J* = 8.22 Hz, 2H), 8.16–8.21 (m, 1H), 12.72 (br s, 1H). ^13^C NMR (126 MHz, DMSO-*d*_6_) δ ppm 20.9, 22.2, 22.2, 23.6, 24.8, 24.9, 27.0, 27.3, 35.2, 52.0, 52.2, 52.6, 110.4, 119.2, 125.9, 128.1, 130.5, 133.1, 143.9, 159.3, 162.6, 167.1, 172.0. LC/MS (method B): *R*_t_ = 2.10 min; HRMS: *m*/*z* [M + H]^+^ calcd for C_24_H_29_F_3_N_5_O_2_S: 508.1994, found 508.1989. [α]_D_^20^ = +53.44 (*c* = 0.320 in MeOH).

### Biochemical Assays

#### hUSP21 HTRF Assay

HTRF assay was performed in an assay buffer consisting of 25 mM *N*-(2-hydroxyethyl)piperazine-*N*′-ethanesulfonic acid (HEPES, pH 8.0) (Alfa Aesar), 50 mM NaCl (Sigma-Aldrich), 2.5 mM dithiothreitol (DTT) (Sigma), 0.002% Tween-20 (Sigma), and 0.0025% bovine serum albumin (BSA) (Sigma) in a final volume of 8 μL at room temperature. All substrate and enzyme solutions were diluted in an assay buffer. First, 2 μL of peptide solution (Btn-Ahx-PNIRFLD-K(Ubi)-LPQQT-GD-amide, Biosyntan) was added to the 40 nL 1 mM compound solution in 100% DMSO (Sigma) at a final concentration of 10 nM (=*K*_m_) for screening. For compound profiling, 50 nL of the compound at a max 25 μM at 11 concentrations (diluted 1:3.5) was tested. Incubation time was 15–20 min at room temperature. The reaction was started with the addition of in-house produced 2 μL of enzyme solution (construct pD-Ins6Z-USP21#101, human, full-length ZZ Tag, PPB:15819) at a final concentration of 10 nM and incubated for 25 min at room temperature. The stop solution (DUB inhibitor PR619, Abcam) was prepared in 25 mM HEPES, pH 7.5 (Alfa Aesar) containing 0.01% BSA and then left for 10 min at room temperature before filtering with a 0.2 μM PES Membrane (Thermo Fisher). The reaction was stopped with the addition of 2 μL of 200 μM PR619. Afterward, 2 μL of detection solution consisting of 10 nM DY-648 streptavidin (DYOMICS, lyophilisate), 2 nM primary ubiquitinylated antibody (clone FK2, monoclonal, mouse IgG1, Millipore), and 1 nM secondary antibody (LANCE Eu-W1024-labeled anti-mouse IgG, PerkinElmer) was added and incubated for 90 min at room temperature for screening or 120 min for compound profiling. The reference compound for the primary screening was Ubv21Cδ2. For the assay development, 384-well plates (small volume (SV), black, Greiner #784101) and for the screening, 1536-well plates (HIBASE black, Greiner #782076) were used. The plates were measured on the ViewLux in screening or PHERAstar FSX in compound profiling. For PHERAstar FSX, the Optic module was HTRF at an excitation of 337 nm, detection A-counts = 665 nm, and detection B-counts = 620 nm. The number of flashes per well was set to 3. All of the experimental data were plotted using Genedata analysis. The *Z*′-factor and S/B of the assay were >0.7 and ∼2, respectively.

#### hUSP21 HTRF Interference Assay

The HTRF interference assay was performed during the retesting to further eliminate false positives caused by the effect of compounds on the assay format. The assay is performed similarly to the HTRF assay but in the absence of the enzyme to identify target-independent activity.

#### Ubiquitin (Ub) Rhodamine 110 Assays

USP21, USP2, USP7, and USP22 Ub-rhodamine (Ub-Rhod) 110 assays were performed in a buffer consisting of 25 mM Tris–HCl, pH 7.4, 2.5 mM DTT, 0.002% Tween-20, and 0.0025% BSA at room temperature. The final assay volume was 6 μL. USP2 (catalytic domain aa259–605, His Tag Boston Biochem #E-506), USP7 (full length, His Tag, Boston Biochem #E-519), and USP22 (complex of four subunits) were tested in the Rhod assay for selectivity at a final concentration of 0.5, 0.5, and 5 nM in the assay buffer (for USP22, 5 μM ZnCl_2_ was added in the buffer), respectively. First, 2 μL of 1 nM USP21 (or selectivity enzyme) solution was added to the compound plate and preincubated for 15–20 min at room temperature. The USP21 enzyme concentration was chosen to be in the linear range of reaction and had an S/B of about 20. Ub-Rhod 110 (UBPBio) as a substrate (*K*_m_ ∼ 50 nM) was added at a 50 nM final concentration and incubated for 25 min at room temperature. The reaction was stopped by 50 mM citric acid in 25 mM HEPES, pH 7.5, containing 0.01% BSA. Fluorescence intensity was measured by PHERAstar at ex/em 485/520 nm after incubation of the plates for 60 min at room temperature. The USP21 and USP22 were measured with five flashes and USP2 and USP7 in fly mode.

#### Ubiquitin Aminoluciferin (Ub-AML) Assay

The Ub-AML (Boston Biochem) assay was performed in an assay buffer consisting of 25 mM Tris–HCl, pH 7.4, 2.5 mM DTT, 0.002% Tween-20, and 0.0025% BSA at room temperature. The final assay volume was 8 μL. First, 2 μL of 1 nM USP21 is added to a 2 μL of 300 nM Ub-AML (*K*_m_ ∼ 350–400 nM) and incubated for 25 min. The reaction was stopped with 2 μL of 200 μM PR619. Then, 2 μL of luciferin detection reagent (Promega V8920/1) was used to detect luciferin at a final concentration of 0.5-fold. For the luminescence detection, 1536-well plates (Greiner, white) were measured on the ViewLux at 120 s exposure time.

#### USP Selectivity Assay

DUB activity was monitored via a ubiquitin rhodamine 110 fluorometric assay in buffer comprising 20 mM Tris–HCl (pH 8.0), 30 mM NaCl, 0.01% (v/v) Triton X-100, and 5 mM DTT. Briefly, compounds were diluted to 10 and 50 μM before adding 200 nM ubiquitin rhodamine 110 (# M3022, UBPBio). Within the panel, the lowest substrate *K*_m_ is 200 nM (with USP5); therefore, compounds were screened with a 200 nM substrate. Individual DUBs were then added (see the Supporting Information) and mixed briefly within a 20 μL of the final reaction volume (with 0.5% DMSO). Plates were then briefly centrifuged (1200 rpm, 23 °C, 1 min). Final fluorescence signals were acquired (excitation: 485, emission: 528) using a Synergy H1 microplate reader (Biotek) with GenX5 software (Version 3.03). Linear regression analysis was performed, and data was reported as a percentage of the enzymatic activity in the presence of the compound relative to DMSO controls (defined as 100% activity) using GraphPad Prism software (Version 7).

### Biophysical Methods

#### USP21 Protein Production

Cloning of expression vectors for recombinant USP21 used for SPR experiments was performed as follows. The cDNAs encoding the protein sequence of human USP21 (Q9UK80, 209–563) with an N-terminally fused thrombin cleavable Hexa-His Tag were optimized for expression in *Escherichia coli*. Proteins were expressed in *E. coli* BL21(DE3) following 0.25 mM isopropyl β-d-1-thiogalactopyranoside (IPTG) induction at 17 °C overnight. For purification, cell pellets were resuspended in buffer A (50 mM Tris pH 7.5, 500 mM NaCl, 10 mM imidazole, 10% glycerin, 1 mM tris(2-carboxyethyl)phosphine (TCEP)). Cells were lysed using a high-pressure microfluidics apparatus, and the cell debris was pelleted by centrifugation. The supernatant was applied to Protino Ni-NTA beads, washed with buffer A until the baseline was reached, and eluted with buffer B (buffer A with 300 mM imidazole) using a linear gradient over 4 CV. The elution pool was diluted 1:10 with buffer C (20 mM Tris pH 7.5, 10% glycerin, 1 mM TCEP), filtered, and then applied to a pre-equilibrated MonoS 10/100 GL (Cytiva) cation exchange column. The protein was eluted by running a linear gradient from the low salt buffer (buffer C + 50 mM NaCl) to the high salt buffer (buffer C + 1000 mM NaCl). As a final step, the protein pool from the cation exchange chromatography step was purified by size exclusion chromatography using a HiLoad 26/600 Superdex 200 pg column in buffer D (20 mM Tris pH 7.5, 150 mM NaCl, 10% glycerin, 1 mM TCEP).

#### SPR Experiments

Determination of *K*_d_ for screening hit **1**: SPR was performed using a Biacore T200 instrument at 15 °C. Recombinant N-terminally His-tagged USP21(aa209–563) diluted to 20 μg/mL into HBS-P+ buffer was injected onto a Tris-NTA converted streptavidin sensor chip (Cytiva) with a flow rate of 10 μL/min and a contact time of 500 s to reach a density of ∼8000 resonance unit (RU). The screening compound **1** was serially diluted into DMSO with a start concentration of 20 μM in 1:3 dilution steps and transferred to the assay buffer to achieve the final test concentration at a DMSO concentration of 1%. For binding analysis, a multi-cycle protocol was chosen with a contact time of 60 s, a flow rate of 30 μL/min, and a dissociation time of 200 s. Binding constants were calculated from the steady state using Biacore Insight Evaluation Software, assuming a 1:1 binding model as an average of four titrations. Determination of *K*_d_ for compounds **18** and **21**: SPR was performed using a Biacore 8K instrument for single-cycle experiments. Recombinant USP21 diluted to 20 μg/mL into 10 mM acetate buffer at pH 5.5 in the presence of 1 μM of an early project compound from a different lead series (hUSP21 HTRF IC_50_ = 324 nM, hUSP21 Ub-rhodamine IC_50_ = 547 nM) was immobilized by amine coupling with a flow rate of 10 μL/min onto Series S CM5 sensor chips (Cytiva). Immobilization levels were kept similar to the above. Single-cycle experiments were used to generate the affinity (*K*_d_) and kinetics of small-molecule binding run in a 1:3 dilution series at a start concentration of 100 nM and six titration steps at a flow rate of 20 μL/min, with an association time of 180 s and a dissociation time of up to 1800 s at 15 °C. The running buffer of the duplicate measurements is 10 mM HEPES, pH 7.4, 150 mM NaCl, 0,005% surfactant P20 further supplemented with 2 mg/mL BSA and 1% DMSO. Affinity and kinetic analysis were performed using Biacore Insight Evaluation Software on double reference subtracted sensorgrams.

#### SPR Competition Assay

SPR competition assays were performed using a Biacore S200 instrument and the A–B–A inject function. Recombinant USP21 was immobilized as described before for the *K*_d_ measurements using the CM5 chip experiments. The A–B–A injection method was used with each of the SMOLs at 1 μM concentration with 30 s contact time of analyte A (SMOLs) followed by ubiquitin titration that was run in a 1:2 dilution series at a start concentration of 25 μM and eight titration steps at a flow rate of 30 μL/min, with an association time of 120 s and a dissociation time of 200 s at 15 °C. Each titration was performed four times, and measurements were analyzed using Biacore Insight Evaluation Software on double reference subtracted sensorgrams.

#### Thermal Shift Assay

Experiments were carried out with the QuantStudio7 Flex Real-Time PCR system (Thermo Fisher Scientific) in a 384-well plate format with 5 μL of reaction volume. Melting curves were obtained at an USP21 protein concentration of 3.8 μM and 5xSYPRO Orange (Invitrogen) using a buffer containing 20 mM Tris, pH 7.5; 150 mM NaCl; 10% glycerol; and 1.0 mM TCEP. USP2 (Boston Biochem #E-506) and USP7 (Boston Biochem #E-519) proteins were used with the concentrations of 2 and 0.8 μM in 50 mM HEPES pH 7.5; 100 mM NaCl; and 1 mM TCEP with 8xSYPRO Orange. For binding experiments, compounds were added from 10 mM stock solution to a final concentration of 100 μM. As a control, 1% DMSO was used. Scans were measured from 25 to 95 °C at a scanning rate of 4 °C/min. All TSA data were analyzed using a Genedata Assay Analyzer.

### Cellular Assays

#### NF-κB Reporter Assay

HEK293T cells were seeded in a six-well plate at 8 × 10^5^ cells/well in 2 mL of Dulbecco’s modified Eagle’s medium (DMEM) supplemented with 10% fetal bovine serum (FBS), penicillin (100 U/mL), and streptomycin (100 μg/mL) and transfected 4 h after seeding using an X-tremeGENE HP transfection reagent, following the manufacturer’s instructions with a total of 2 μg of plasmid constructs (pGL4 NF-κB-FLuc (NF-κB reporter Firefly luciferase, Promega), RIPK (Addgene #78842), pGL4 TK-RLuc (Renilla luciferase driven by a TK promoter, Promega)) for normalization, full-length USP21 (cloned into pcDNA3.1, Myc-tagged) or USP21-C221R mutant (mutagenized USP21 in pcDNA3.1, Myc-tagged), or pCDNA3.1 empty vector for no USP21 controls. Twenty hours after transfection, cells were replated at 2 × 10^5^ cells/well in 100 μL in 96-well white plates (white, 6555098, Greiner Bio-One). Four hours after replating, cells were treated with compounds or DMSO (note: DMSO concentrations were kept consistent across all cells). Promega Dual-Luciferase Reporter Assay kit was then used to prepare samples for Firefly and Renilla signal measurement on a CLARIOstar microplate reader (BMG) 20 h after compound treatment. Relative luciferase units were calculated by normalizing the Firefly signal to the background Renilla signal, followed by normalization to DMSO controls of wild-type USP21.

#### USP21 HiBiT CETSA

HEK293T cells were seeded in a six-well plate at 8 × 10^5^ cells/well in 2 mL of DMEM supplemented with 10% FBS, penicillin (100 U/mL), and streptomycin (100 μg/mL) and transfected 4 h after seeding using an X-tremeGENE HP transfection reagent, following the manufacturer’s instructions with a total of 2 μg of plasmid constructs (C-terminally tagged USP21 HiBiT and empty pCDNA3.1 vector). The next day, HEK293T cells were trypsinized and transferred to 96-well PCR plates (50 μL/well, at 2 × 10^5^ cells/mL), treated with compound or DMSO, covered with a breathable paper adhesive film, and incubated for 1 h at 37 °C. The breathable paper adhesive film was then replaced with a PCR adhesive film, and cells were heated at indicated temperatures in an Applied Biosystems VeritiPro thermal cycler for 3 min, followed by 3 min incubation at rt before the addition of LgBiT solution (200 nM LgBiT, 2% NP-40, protease inhibitors (Roche cOmplete, ethylenediaminetetraacetic acid (EDTA)-free Protease Inhibitor) in OptiMEM no phenol red (Gibco)) to lyse cells. After 10 min in the LgBiT solution, 25 μL of Nano-Glo substrate (Promega, 8 μL/mL OptiMEM no phenol red media) was added, the solution was mixed once, 20 μL was transferred to 384 white plates in quadruplicates, and the luciferase signal was read using a CLARIOstar microplate reader (BMG). Relative protein abundance percentage was calculated by taking the mean of technical quadruplicates and normalizing to 37 °C DMSO samples.

#### Proliferation Assays

Cells were plated in 384-well plates, and after 24 h, the cell viability of one plate (zero-point plate) was determined using the CellTiter-Glo Luminescent Cell Viability Assay (Promega). The test compound was added to the wells of the other plates employing an HP D300 Digital Dispenser. Cell viability was assessed after exposure for 72 h, using the CellTiter-Glo Luminescent Cell Viability Assay (Promega). IC_50_ values were determined by means of a four-parameter fit on measurement data, which were normalized to a vehicle (DMSO)-treated cells (=100%) and measurement readings taken immediately before compound exposure (=0%).

### Pharmacokinetic Assays

#### Caco-2 Permeability Assay

Caco-2 cells [purchased from the German Collection of Microorganisms and Cell Cultures (DSMZ)] were seeded at a density of 4.5 × 10^4^ cells/well on 24-well insert plates [0.4 μm pore size, 0.3 cm^2^ (Costar)] and grown for 13–15 days in DMEM medium supplemented with 10% fetal calf serum (FCS), 1% GlutaMAX (100×, Gibco), 100 U/mL penicillin, 100 μg/mL streptomycin (Gibco), and 1% nonessential amino acids (100×, Thermo Fischer Scientific). The cells were maintained at 37 °C in a humidified 5% CO_2_ atmosphere. The medium was changed every 2–3 days.

The bidirectional transport assay for the evaluation of Caco-2 permeability was undertaken in 24-well insert plates using a robotic system (Tecan). Before the assay was run, the culture medium was replaced by a transport medium (FCS-free HEPES carbonate transport buffer pH 7.2). For the assessment of monolayer integrity, the transepithelial electrical resistance (TEER) was measured. Only monolayers with a TEER of at least 400 Ω·cm^2^ were used. Test compounds were pre-dissolved in DMSO and added either to the apical or basolateral compartment at a final concentration of 2 μM. The evaluation was performed in triplicate. Before and after incubation at 37 °C for 2 h, samples were taken from both compartments and, after precipitation with MeOH, analyzed by LC/MS-MS. The apparent permeability coefficient (*P*_app_) was calculated both for the apical to basolateral (A → B) and the basolateral to apical (B → A) direction using the following equation: *P*_app_ = (*V*_r_/*P*_0_)(1/*S*)(*P*_2_/*t*), where *V*_r_ is the volume of medium in the receiver chamber, *P*_0_ is the measured peak area of the test compound in the donor chamber at *t* = 0, *S* is the surface area of the monolayer, *P*_2_ is the measured peak area of the test compound in the acceptor chamber after incubation for 2 h, and *t* is the incubation time. The efflux ratio (ER) basolateral (B) to apical (A) was calculated by dividing *P*_app_ B–A by *P*_app_ A–B.

#### In Vitro Metabolic Stability in Rat Hepatocytes

Hepatocytes from Han/Wistar rats were isolated via a two-step perfusion method. After perfusion, the liver was carefully removed from the rat: the liver capsule was opened, and the hepatocytes were gently shaken out into a Petri dish with ice-cold Williams’ medium E (WME). The resulting cell suspension was filtered through a sterile gauze into 50 mL Falcon tubes and centrifuged at 50*g* for 3 min at rt. The cell pellet was resuspended in WME (30 mL) and centrifuged twice through a Percoll gradient at 100*g*. The hepatocytes were washed again with WME and resuspended in a medium containing 5% FCS. Cell viability was determined by trypan blue exclusion. For the metabolic stability assay, liver cells were distributed in WME containing 5% FCS into glass vials at a density of 1.0 × 10^6^ vital cells/mL. The test compound was added to a final concentration of 1 μM. During incubation, the hepatocyte suspensions were continuously shaken at 580 rpm, and aliquots were taken at 2, 8, 16, 30, 45, and 90 min, to which an equal volume of cold MeOH was immediately added. Samples were frozen at −20 °C overnight and subsequently centrifuged for 15 min at 3000 rpm. The supernatant was analyzed with an Agilent 1200 HPLC system with LC-MS/MS detection. The half-life of a test compound was determined from the concentration–time plot. From the half-life, the intrinsic clearances, the hepatic in vivo blood clearance (CL), and maximal oral bioavailability (*F*_max_) were calculated using the “well-stirred” liver model^53^ together with the additional parameters for liver blood flow, specific liver weight, and the amount of liver cells in vivo and in vitro. The following parameter values were used: liver blood flow—4.2 L/(h kg); specific liver weight—32 g/kg body weight; liver cells in vivo—1.1 × 10^8^ cells/g liver; and liver cells in vitro—1.0 × 10^6^/mL.

#### CYP Inhibition Assay

The ability of substances to inhibit CYP1A2, CYP2C8, CYP2C9, CYP2D6, and CYP3A4 in humans was investigated with pooled human liver microsomes as enzyme sources in the presence of standard substrates (see below), which form CYP-isoform-specific metabolites. The inhibitory effects were investigated with six different concentrations of the test compounds (0.6, 1.3, 2.5, 5, 10, and 20 μM), compared with the extent of the CYP-isoform-specific metabolite formation of the standard substrates in the absence of the test compounds, and the corresponding IC_50_ values were calculated. IC_50_ determination for CYP3A4 activity was additionally determined after 30 min preincubation of the enzyme in the presence of NADP to determine the potential for time-dependent inhibition. A standard inhibitor, which specifically inhibits a single CYP isoform, served as a control for all results obtained. Procedure: Phenacetin, amodiaquine, diclofenac, dextromethorphan, or midazolam were incubated with human liver microsomes in the presence of six different concentrations of a test compound (as a potential inhibitor) in 96-well plate format. Standard incubation mixtures comprised of 1.0 mM NADP, 1.0 mM EDTA, 5.0 mM glucose 6-phosphate, glucose 6-phosphate dehydrogenase (1.5 U), and 50 mM phosphate buffer (pH 7.4) in a total volume of 200 μL. Test compounds were preferably dissolved in acetonitrile. Then, 96-well plates were incubated with the enzyme preparation at 37 °C for a defined time. The reactions were stopped by adding 100 μL of acetonitrile in which stable isotope-labeled internal standards are always present. Precipitated proteins were removed by centrifugation, and the supernatants were combined and analyzed by LC-MS/MS.

### Physicochemical Assays

#### Stability of Compounds in Solution

Solution stability was determined by HPLC-UV or LC-MS. A stock solution of the test compound in an organic solvent was created and mixed with the respective buffer. Injections were made immediately after mixing for time zero injection and then again after different time points. The degradation rate (recovery in %) was calculated by relating peak areas after different time points to the time zero injection.

#### Aqueous Solubility of Compounds

Test compounds were applied as DMSO solutions or directly from powder. After the addition of the buffer, solutions were shaken at rt for 24 h. The undissolved material was removed by filtration or centrifugation. The compound dissolved in the supernatant was quantified by HPLC-UV or HPLC-MS/MS.

#### log *D* Measurement

log *D* values at pH 7.5 were recorded using an indirect method for determining hydrophobicity constants by reverse-phase HPLC. A series of compounds with well-known log *D* values were used for calibration. Test compounds were injected into the same HPLC system. The lipophilicity of compounds was then assessed by comparison to the calibration curve.

## Supporting Information Available

Molecular formula strings (CSV)
USP selectivity panel; biochemical characterization of compound **11**; selectivity data for BAY-805 (**21**) and BAY-728 (**22**); experimental procedures, NMR spectra and HPLC chromatograms for compounds **1**–**22** (PDF)

## Abbreviations Used

AML: aminoluciferin
CETSA: cellular thermal shift assay
Cpd: compound
DUB: deubiquitinating enzyme
ER: efflux ratio
*K*_d_: equilibrium constant
k_off_: dissociation constant
*k*_on_: association constant
LLE: lipophilic ligand efficiency
PBS: phosphate-buffered saline
RU: resonance unit
SAR: structure–activity relationship
SEM: standard error of mean
SGC: Structural Genomics Consortium
SPR: surface plasmon resonance
TPSA: topological polar surface area
TSA: thermal shift assay
TR-FRET: time-resolved fluorescence resonance energy transfer
Ub: ubiquitin
USP: ubiquitin-specific protease
