# Supplementary material for: Discovery and Characterization of BAY-805, a Potent and Selective Inhibitor of Ubiquitin-Specific Protease USP21
Source: J Med Chem. 2023 Feb 20;66(5):3431–47. doi: 10.1021/acs.jmedchem.2c01933 (PMC10009755; doi:10.1021/acs.jmedchem.2c01933)
Supplement: Supplementary file 2 — jm2c01933_si_002.pdf [file jm2c01933_si_002.pdf]

Supporting Information

Discovery and Characterization of BAY-805, a Potent and Selective Inhibitor of Ubiquitin Specific Protease USP21

Fabian Göricke,*†[1] Victoria Vu,†[4] Leanna Smith,[4] Ulrike Scheib,[3] Raphael Böhm,[3] Namik Akkilic,[3] Gerd Wohlfahrt,[2] Jörg Weiske,[3] Ulf Bömer,[3] Krzysztof Brzezinka,[3] Niels Lindner,[1] Philip Lienau,[2] Stefan Gradl,[2] Hartmut Beck,[1] Peter J. Brown,[4] Vijayaratnam Santhakumar,[4] Masoud Vedadi,[4,5] Dalia Barsyte-Lovejoy,[4] Cheryl H. Arrowsmith,*[4] Norbert Schmees,*§[3] Kirstin Petersen*§[2]

[1] Bayer AG, Research & Development, Pharmaceuticals, 42096 Wuppertal, Germany.

[2] Bayer AG, Research & Development, Pharmaceuticals, 13353 Berlin, Germany.

[3] Nuvisan Innovation Campus Berlin, 13353 Berlin, Germany.

[4] Structural Genomics Consortium, University of Toronto, Toronto, Ontario M5G 1L7, Canada

[5] Department of Pharmacology and Toxicology, University of Toronto, Toronto, Ontario M5S 1A8, Canadaa

†F.G. and V.V. contributed equally to this work.

§N.S. and K.P. contributed equally.

*** For correspondence:** fabian.goericke@bayer.com, norbert.schmees@nuvisan.com, kirstin.petersen@bayer.com, cheryl.arrowsmith@uhnresearch.ca

Table of Contents

**USP Selectivity Panel**	S3
**Biochemical Characterization of Compound 11**	S4
IC_{50} Determination in hUSP21 HTRF Assay	S4
IC_{50} Determination in hUSP21 Ub-Rhodamine Assay	S5
**Selectivity Data for BAY-805 and BAY-728**	S6
USP Selectivity Screen (SGC)	S6
DUB Selectivity Screen in DUB*profiler*™ (Ubiquigent)	S8
Off-target Safety Screen (Eurofins Panlabs)	S18
Kinase Selectivity Screen (Eurofins Panlabs)	S26
**Chemistry**	S51
General Methods and Materials	S51
Experimental Procedures	S53
NMR Spectra	S91
HPLC Chromatograms	S121

USP Selectivity Panel

Table S1. Construct and expression info, purity, and concentration in assays for USPs on USP selectivity panel.

DUB	Amino acid range (start - end)	Host/ strain	Construct ID	Purity (approximate, %)	Concentration (nM)
USP21	209 - 563	*E.coli*, BL21(DE3)CodonPlusRIL	USP21:SDC122-G08	95	2
USP02	251 - 605	*E.coli*, BL21(DE3)CodonPlusRIL	USP02:SDC054-H01	90	2
USP04	1-932	*Sf9*	USP04:YTC039-H06	95	0.3
USP30	57 - 517	*Sf9*	USP30:YTC042-C10	90	15
USP07	1 - 1102	*Sf9*	USP7:YTC009-B12	90	1
USP08	734 - 1110	*E.coli*, BL21(DE3)CodonPlusRIL	USP08:SDC064-H08	90	1
USP15	1 - 952	*Sf9*	USP15:SDC227-B03	90	1
USP22	1 - 525	*Sf9*	USP22: MVC012B09	90	1000
USP9X	1551 - 1970	*E.coli*, BL21(DE3)CodonPlusRIL	USP09X:YTC014-F08	90	125

USP05	1-835	*Sf9*	USP05:SDC227-A05	95	1
ATAXIN3	1-370	*E.coli*, BL21(DE3)CodonPlusRIL	ataxin3:SDC068-D12	90	1000

Biochemical Characterization of Compound 11

IC_{50} Determination in hUSP21 HTRF Assay:

Figure S1: Dose response curve to determine IC_{50} value for compound **11** in hUSP21 HTRF assay. Values represent arithmetic mean of at least two independent experiments. Efficacy of compound **11** is < 70 %.

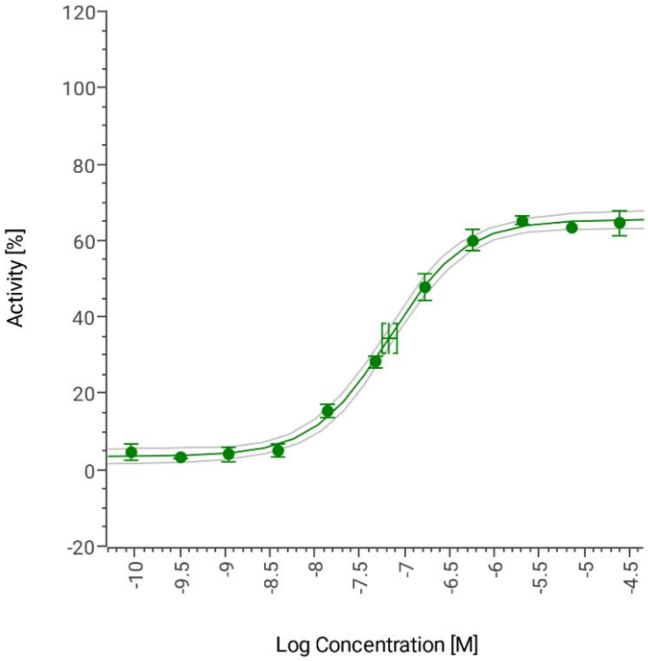

IC$_{50}$ Determination in hUSP21 Ub-Rhodamine Assay:

Figure S2: Dose response curve to determine IC$_{50}$ value for compound **11** in hUSP21 Ub-Rhodamine assay. Values represent arithmetic mean of at least two independent experiments. Efficacy of compound **11** is < 30 %.

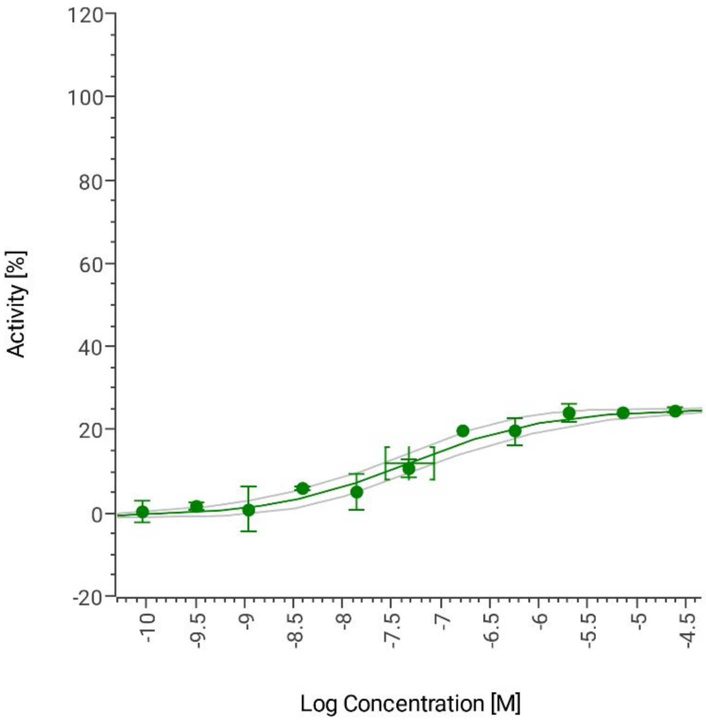

Selectivity Data for BAY-805 and BAY-728

USP Selectivity Screen (SGC):

Table S2. SGC USP selectivity screen for BAY-805 (**21**) was performed at 50 µM and 10 µM compound concentrations. Biochemical assay results are presented as mean % activity (relative to the positive control enzyme signal), e.g. 100% activity = 0% inhibition of the enzyme; 60 % activity = 40% inhibition. BAY-805 (**21**) shows no significant autofluorescent properties in the assay. Significant response (< 50% activity) at 10 µM compound concentration was noted for USP21 (9% activity = 91% inhibition), USP10 (50% activity = 50% inhibition), and USP22 (47% activity = 53% inhibition).

DUB Target	Activity (% Control*) BAY-805 50 µM	Activity (% Control*) BAY-805 10 µM
USP2	73	79
USP4	93	98
USP5	80	97
USP7	84	87

USP8	71	83
USP9X	97	95
USP10	45	50
USP21	6	6
USP22	43	47
USP30	65	69

Table S3. SGC USP selectivity screen for BAY-728 (**22**) performed at 50 µM and 10 µM compound concentration. Biochemical assay results are presented as mean % activity (relative to the positive control enzyme signal), e.g. 100% activity = 0% inhibition of the enzyme; 60% activity = 40% inhibition. BAY-728 (**22**) shows no significant autofluorescent properties in the assay. Significant response (< 50% activity) at 10 µM compound concentration was noted for USP10 (48% activity = 52% inhibition) and USP22 (41% activity = 59% inhibition).

**DUB Target**	**Activity (% Control*)**	**Activity (% Control*)**
	**BAY-728 50 µM**	**BAY-728 10 µM**

USP2	60	79
USP4	91	98
USP5	78	94
USP7	81	82
USP8	69	77
USP9X	97	97
USP10	32	48
USP21	54	56
USP22	35	41
USP30	65	74

DUB Selectivity Screen in DUB*profiler*™ (Ubiquigent):

Table S4. DUB*profiler*™ (Ubiquigent) for BAY-805 (**21**) performed at 10 μM compound concentration. Biochemical assay results are presented as mean % activity (relative to the positive control enzyme signal), e.g. 100% activity = 0% inhibition of the enzyme; 60% activity = 40% inhibition. BAY-805 (**21**) shows no significant autofluorescent properties in the assay. Significant response (< 50% activity) at 10 μM compound concentration was noted for USP21 (9% activity = 91% inhibition).

DUB Target	Conc.	Activity (% Control*)
USP1/UAF1	10 μM	111
USP2	10 μM	102
USP4	10 μM	103
USP5	10 μM	110
USP5 (+Ubiquitin @ Kd)	10 μM	110
USP5 (+Ubiquitin @ Bmax)	10 μM	101
USP6	10 μM	102

USP7	10 µM	100
USP8	10 µM	96
USP9x	10 µM	108
USP11	10 µM	115
USP12/UAF1/WDR20	10 µM	91
USP14 (Proteasome-VS @ Kd)	10 µM	98
USP15	10 µM	113
USP16	10 µM	98
USP17	10 µM	97
USP19	10 µM	101
USP20	10 µM	87
USP21	10 µM	9
USP25	10 µM	116
USP27x	10 µM	108
USP28	10 µM	102
USP30	10 µM	97

USP35	10 µM	105
USP36	10 µM	101
USP45	10 µM	96
USP46/UAF1/WDR20	10 µM	97
CYLD	10 µM	102
UCHL1	10 µM	114
UCHL3	10 µM	101
UCHL5	10 µM	98
BAP1	10 µM	106
OTU1	10 µM	104
OTUB2	10 µM	95
OTUD1	10 µM	99
OTUD3	10 µM	101
OTUD5 (p117S)	10 µM	90
OTUD6A	10 µM	99
OTUD6B	10 µM	92

Cezanne	10 µM	99
VCPIP	10 µM	114
AMSH-LP	10 µM	101
AMSH-LP (+Zinc)	10 µM	93
Ataxin3	10 µM	106
Ataxin3L	10 µM	111
JOSD1	10 µM	101
JOSD2	10 µM	113

*% Control = [(sample – mean no enzyme)/(mean plus enzyme – mean no enzyme)]□100. Km is greater than substrate concentration except for USP1/UAF1, USP11, USP15, USP20, UCHL1, UCHL3 and OTUD3. For these DUBs the potency of competitive inhibitors may be underestimated.

Table S5. DUB*profiler*™ (Ubiquigent) for BAY-805 (**21**) performed at 1 µM compound concentration. Biochemical assay results are presented as mean % activity (relative to the positive control enzyme signal), e.g. 100% activity = 0% inhibition of the enzyme; 60% activity = 40% inhibition. BAY-805 (**21**) shows no significant autofluorescent properties in the assay. Significant

response (< 50% activity) at 1 μM compound concentration was noted for USP21 (12% activity = 88% inhibition).

DUB Target	Conc.	Activity (% Control*)
USP1/UAF1	1 μM	117
USP2	1 μM	93
USP4	1 μM	102
USP5	1 μM	107
USP5 (+Ubiquitin @ Kd)	1 μM	105
USP5 (+Ubiquitin @ Bmax)	1 μM	93
USP6	1 μM	106
USP7	1 μM	101
USP8	1 μM	94
USP9x	1 μM	97
USP11	1 μM	111
USP12/UAF1/WDR20	1 μM	96

USP14 (Proteasome-VS @ Kd)	1 µM	103
USP15	1 µM	118
USP16	1 µM	100
USP17	1 µM	93
USP19	1 µM	100
USP20	1 µM	88.5
USP21	1 µM	12
USP25	1 µM	104
USP27x	1 µM	105
USP28	1 µM	123
USP30	1 µM	102
USP35	1 µM	109
USP36	1 µM	110
USP45	1 µM	121
USP46/UAF1/WDR20	1 µM	92
CYLD	1 µM	105

UCHL1	1 µM	103
UCHL3	1 µM	113
UCHL5	1 µM	108
BAP1	1 µM	111
OTU1	1 µM	104
OTUB2	1 µM	106
OTUD1	1 µM	102
OTUD3	1 µM	102
OTUD5 (p117S)	1 µM	102
OTUD6A	1 µM	103
OTUD6B	1 µM	104
Cezanne	1 µM	102
VCPIP	1 µM	106
AMSH-LP	1 µM	96
AMSH-LP (+Zinc)	1 µM	99
Ataxin3	1 µM	109

Ataxin3L	1 µM	109
JOSD1	1 µM	108
JOSD2	1 µM	113

*% Control = [(sample – mean no enzyme)/(mean plus enzyme – mean no enzyme)]□100. Km is greater than substrate concentration except for USP1/UAF1, USP11, USP15, USP20, UCHL1, UCHL3 and OTUD3. For these DUBs the potency of competitive inhibitors may be underestimated.

Table S6. DUB*profiler*™ (Ubiquigent) for BAY-728 (**22**) performed at 10 µM compound concentration. Biochemical assay results are presented as mean % activity (relative to the positive control enzyme signal), e.g. 100% activity = 0% inhibition of the enzyme; 60% activity = 40% inhibition. BAY-728 (**22**) shows no significant autofluorescent properties in the assay. Significant response (< 50% activity) at 10 µM compound concentration was noted for USP21 (40% activity = 60% inhibition).

**DUB Target**	**Conc.**	**Activity (% Control*)**
USP1/UAF1	10 µM	97
USP2	10 µM	82

USP4	10 μM	94
USP5	10 μM	105
USP5 (+Ubiquitin @ Kd)	10 μM	90
USP5 (+Ubiquitin @ Bmax)	10 μM	101
USP6	10 μM	105
USP7	10 μM	87
USP8	10 μM	109
USP9x	10 μM	99
USP11	10 μM	102
USP12/UAF1/WDR20	10 μM	106
USP14 (Proteasome-VS @ Kd)	10 μM	112
USP15	10 μM	105
USP16	10 μM	107
USP17	10 μM	88
USP19	10 μM	96
USP20	10 μM	113

USP21	10 µM	40
USP25	10 µM	105
USP27x	10 µM	96
USP28	10 µM	106
USP30	10 µM	103
USP35	10 µM	110
USP36	10 µM	95
USP45	10 µM	98
USP46/UAF1/WDR20	10 µM	100
CYLD	10 µM	100
UCHL1	10 µM	103
UCHL3	10 µM	106
UCHL5	10 µM	147
BAP1	10 µM	92.4
OTU1	10 µM	113
OTUB2	10 µM	101

OTUD1	10 µM	104
OTUD3	10 µM	100
OTUD5 (p117S)	10 µM	121
OTUD6A	10 µM	105
OTUD6B	10 µM	100
Cezanne	10 µM	93
VCPIP	10 µM	115
AMSH-LP	10 µM	88
AMSH-LP (+Zinc)	10 µM	107
Ataxin3	10 µM	90
Ataxin3L	10 µM	106
JOSD1	10 µM	99
JOSD2	10 µM	110

*% Control = [(sample – mean no enzyme)/(mean plus enzyme – mean no enzyme)]□100. Km is greater than substrate concentration except for USP1/UAF1, USP11, USP15, USP20, UCHL1, UCHL3 and OTUD3. For these DUBs the potency of competitive inhibitors may be underestimated.

Table S7. DUB*profiler*™ (Ubiquigent) for BAY-728 (**22**) performed at 1 µM compound concentration. Biochemical assay results are presented as mean % activity (relative to the positive control enzyme signal), e.g. 100% activity = 0% inhibition of the enzyme; 60% activity = 40% inhibition. BAY-728 (**22**) shows no significant autofluorescent properties in the assay.

DUB Target	Conc.	Activity (% Control*)
USP1/UAF1	1 µM	115
USP2	1 µM	100
USP4	1 µM	98
USP5	1 µM	104
USP5 (+Ubiquitin @ Kd)	1 µM	108
USP5 (+Ubiquitin @ Bmax)	1 µM	96
USP6	1 µM	108
USP7	1 µM	85
USP8	1 µM	93
USP9x	1 µM	101

USP11	1 µM	99
USP12/UAF1/WDR20	1 µM	96
USP14 (Proteasome-VS @ Kd)	1 µM	100
USP15	1 µM	101
USP16	1 µM	95
USP17	1 µM	90
USP19	1 µM	93
USP20	1 µM	109
USP21	1 µM	73
USP25	1 µM	105
USP27x	1 µM	103
USP28	1 µM	99
USP30	1 µM	98
USP35	1 µM	109
USP36	1 µM	100
USP45	1 µM	94

USP46/UAF1/WDR20	1 μM	101
CYLD	1 μM	96
UCHL1	1 μM	100
UCHL3	1 μM	105
UCHL5	1 μM	100
BAP1	1 μM	100
OTU1	1 μM	103
OTUB2	1 μM	107
OTUD1	1 μM	101
OTUD3	1 μM	102
OTUD5 (p117S)	1 μM	96
OTUD6A	1 μM	99
OTUD6B	1 μM	94
Cezanne	1 μM	104
VCPIP	1 μM	103
AMSH-LP	1 μM	105

AMSH-LP (+Zinc)	1 µM	103
Ataxin3	1 µM	90
Ataxin3L	1 µM	91
JOSD1	1 µM	100
JOSD2	1 µM	98

*% Control = [(sample – mean no enzyme)/(mean plus enzyme – mean no enzyme)]□100. Km is greater than substrate concentration except for USP1/UAF1, USP11, USP15, USP20, UCHL1, UCHL3 and OTUD3. For these DUBs the potency of competitive inhibitors may be underestimated.

Off-target Safety Screen (Eurofins Panlabs):

Table S8. Eurofins Panlabs safety screen for BAY-805 (**21**) performed at 10 µM compound concentration. Biochemical assay results are presented as the percent inhibition of specific binding or activity. Significant responses (≥ 50% inhibition) at 10 µM compound concentration were noted for acetyl cholinesterase (72% inhibition) and adenosine transporter (62% inhibition).

Target	Conc.	% Inh.
Aldose Reductase	10 µM	0
ATPase, Na^+/K^+, Heart, Pig	10 µM	-1
Carbonic Anhydrase II	10 µM	3
Cholinesterase, Acetyl, ACES	10 µM	72
Cyclooxygenase COX-1	10 µM	-2
Cyclooxygenase COX-2	10 µM	2
HMG-CoA Reductase	10 µM	16
Leukotriene LTC4 Synthase	10 µM	35
Lipoxygenase 15-LO	10 µM	-3
Monoamine Oxidase MAO-A	10 µM	3
Monoamine Oxidase MAO-B	10 µM	7
Nitric Oxide Synthase, Neuronal (nNOS)	10 µM	-15
Nitric Oxide Synthetase, Inducible (iNOS)	10 µM	-1
Peptidase, Angiotensin Converting Enzyme	10 µM	-33

Phosphodiesterase PDE3	10 μM	3
Phosphodiesterase PDE4	10 μM	1
Phosphodiesterase PDE5	10 μM	13
Thromboxane Synthase	10 μM	-1
Adenosine A1	10 μM	4
Adenosine A_{2A}	10 μM	2
Adenosine A_3	10 μM	12
Adrenergic α_{1A}	10 μM	1
Adrenergic α_{2A}	10 μM	14
Adrenergic α_{2B}	10 μM	4
Adrenergic α_{2C}	10 μM	6
Adrenergic β_1	10 μM	3
Adrenergic β_2	10 μM	3
Adrenergic β_3	10 μM	10
Androgen (Testosterone)	10 μM	4
Angiotensin AT_1	10 μM	14

Angiotensin AT_2	10 μM	-3
Bradykinin B_1	10 μM	6
Bradykinin B_2	10 μM	-7
Cannabinoid CB_1	10 μM	8
Cannabinoid $_{CB2}$	10 μM	1
Dopamine D_1	10 μM	-3
Dopamine D_{2L}	10 μM	8
Dopamine D_{2S}	10 μM	24
Dopamine D_3	10 μM	9
Endothelin ET_A	10 μM	-7
Endothelin ET_B	10 μM	4
Estrogen Erα	10 μM	-6
$GABA_A$, Chloride Channel, TBOB	10 μM	9
$GABA_A$, Flunitrazepam, Central	10 μM	-7
$GABA_B$, Non-Selective	10 μM	-2
Glucocorticoid	10 μM	14

Glutamate, AMPA	10 μM	2
Glutamate, Kainate	10 μM	-7
Glutamate, NMDA, Agonism	10 μM	7
Glutamate, NMDA, Glycine	10 μM	6
Growth Hormone Secretagogue (GHS, Ghrelin)	10 μM	20
Histamine H_1	10 μM	-1
Histamine H_2	10 μM	-16
Histamine H_3	10 μM	-2
Insulin	10 μM	10
Motilin	10 μM	10
Muscarinic M_1	10 μM	18
Muscarinic M_2	10 μM	2
Muscarinic M_3	10 μM	7
Muscarinic M_4	10 μM	-4
Nicotinic Acetylcholine α3β4	10 μM	-3
Opiate δ_1 (OP1, DOP)	10 μM	-5

Opiate κ (OP2, KOP)	10 μM	6
Opiate μ (OP3, MOP)	10 μM	-1
Progesterone PR-B	10 μM	5
Purinergic P2X	10 μM	-4
Purinergic P2Y, Non-Selective	10 μM	5
Serotonin (5-Hydroxytryptamine) 5-HT_{1A}	10 μM	2
Serotonin (5-Hydroxytryptamine) 5-HT_{2A}	10 μM	4
Serotonin (5-Hydroxytryptamine) 5-HT_{2B}	10 μM	3
Serotonin (5-Hydroxytryptamine) 5-HT_{2C}	10 μM	-5
Transporter, Adenosine	10 μM	62
Transporter, Dopamine (DAT)	10 μM	9
Transporter, GABA	10 μM	14
Transporter, Norepinephrine (NET)	10 μM	10
Transporter, Serotonin (5-Hydroxytryptamine) (SERT)	10 μM	13
Vasopressin V_{1A}	10 μM	2

Table S9. In vitro IC_{50} determination of BAY-805 (**21**) on Cholinesterase, Acetyl, ACES at Eurofins Panlabs.

Target	Conc.	% Inh.	IC_{50}
Cholinesterase, Acetyl, ACES	30 µM	74	7.61 µM
	10 µM	74	
	3 µM	4	
	1 µM	11	
	0.3 µM	16	
	0.1 µM	15	

Table S10. Eurofins Panlabs safety screen for BAY-728 (**22**) performed at 10 µM compound concentration. Biochemical assay results are presented as the percent inhibition of specific binding or activity. Significant responses (≥ 50% inhibition) at 10 µM compound concentration were noted for acetyl cholinesterase (75% inhibition).

Target	Conc.	% Inh.
Aldose Reductase	10 µM	2
ATPase, Na^+/K^+, Heart, Pig	10 µM	-3
Carbonic Anhydrase II	10 µM	1
Cholinesterase, Acetyl, ACES	10 µM	75
Cyclooxygenase COX-1	10 µM	3
Cyclooxygenase COX-2	10 µM	5
HMG-CoA Reductase	10 µM	1
Leukotriene LTC4 Synthase	10 µM	5
Lipoxygenase 15-LO	10 µM	-3
Monoamine Oxidase MAO-A	10 µM	5
Monoamine Oxidase MAO-B	10 µM	5
Nitric Oxide Synthase, Neuronal (nNOS)	10 µM	-5
Nitric Oxide Synthetase, Inducible (iNOS)	10 µM	0
Peptidase, Angiotensin Converting Enzyme	10 µM	-28

Phosphodiesterase PDE3	10 μM	4
Phosphodiesterase PDE4	10 μM	1
Phosphodiesterase PDE5	10 μM	27
Thromboxane Synthase	10 μM	15
Adenosine A1	10 μM	-2
Adenosine A_{2A}	10 μM	3
Adenosine A_3	10 μM	24
Adrenergic α_{1A}	10 μM	13
Adrenergic α_{2A}	10 μM	13
Adrenergic α_{2B}	10 μM	1
Adrenergic α_{2C}	10 μM	12
Adrenergic β_1	10 μM	4
Adrenergic β_2	10 μM	6
Adrenergic β_3	10 μM	8
Androgen (Testosterone)	10 μM	7
Angiotensin AT_1	10 μM	27

Angiotensin AT_2	10 µM	-2
Bradykinin B_1	10 µM	23
Bradykinin B_2	10 µM	-7
Cannabinoid CB_1	10 µM	3
Cannabinoid $_{CB2}$	10 µM	4
Dopamine D_1	10 µM	2
Dopamine D_{2L}	10 µM	17
Dopamine D_{2S}	10 µM	25
Dopamine D_3	10 µM	2
Endothelin ET_A	10 µM	7
Endothelin ET_B	10 µM	29
Estrogen Erα	10 µM	-15
$GABA_A$, Chloride Channel, TBOB	10 µM	14
$GABA_A$, Flunitrazepam, Central	10 µM	-10
$GABA_B$, Non-Selective	10 µM	-1
Glucocorticoid	10 µM	43

Glutamate, AMPA	10 µM	-21
Glutamate, Kainate	10 µM	-5
Glutamate, NMDA, Agonism	10 µM	5
Glutamate, NMDA, Glycine	10 µM	19
Growth Hormone Secretagogue (GHS, Ghrelin)	10 µM	8
Histamine H_1	10 µM	5
Histamine H_2	10 µM	0
Histamine H_3	10 µM	2
Insulin	10 µM	-5
Motilin	10 µM	4
Muscarinic M_1	10 µM	36
Muscarinic M_2	10 µM	24
Muscarinic M_3	10 µM	4
Muscarinic M_4	10 µM	-8
Nicotinic Acetylcholine α3β4	10 µM	-1
Opiate δ_1 (OP1, DOP)	10 µM	5

Opiate κ (OP2, KOP)	10 μM	12
Opiate μ (OP3, MOP)	10 μM	-3
Progesterone PR-B	10 μM	16
Purinergic P2X	10 μM	3
Purinergic P2Y, Non-Selective	10 μM	22
Serotonin (5-Hydroxytryptamine) 5-HT_{1A}	10 μM	5
Serotonin (5-Hydroxytryptamine) 5-HT_{2A}	10 μM	2
Serotonin (5-Hydroxytryptamine) 5-HT_{2B}	10 μM	2
Serotonin (5-Hydroxytryptamine) 5-HT_{2C}	10 μM	14
Transporter, Adenosine	10 μM	43
Transporter, Dopamine (DAT)	10 μM	41
Transporter, GABA	10 μM	23
Transporter, Norepinephrine (NET)	10 μM	37
Transporter, Serotonin (5-Hydroxytryptamine) (SERT)	10 μM	11
Vasopressin V_{1A}	10 μM	5

Table S11. In vitro IC_{50} determination of BAY-728 (**22**) on Cholinesterase, Acetyl, ACES at Eurofins Panlabs.

Target	Conc.	% Inh.	IC_{50}
Cholinesterase, Acetyl, ACES	30 µM	78	6.87 µM
	10 µM	76	
	3 µM	8	
	1 µM	19	
	0.3 µM	4	
	0.1 µM	18	

Kinase Selectivity Panel (Eurofins Panlabs):

Table S12. Kinase Selectivity Panel (Eurofins) for BAY-805 (**21**) performed at 10 µM compound concentration. Significant response (> 50% inhibition) at 10 µM compound concentration was noted for PRAK(h) (58% inhibition) and TrkA(h) (57% inhibition).

Kinase	ATP Conc. [mol/L]	Concentration [mol/L]	Inhibition [%]	Enzyme Conc. [mol/L]
AAK1(h)	1.0E-05	1.0E-05	4.09	1.3E-08

Abl(h)	1.0E-05	1.0E-05	5.25	7.7E-10
ACK1(h)	1.0E-05	1.0E-05	-2.92	1.7E-08
ACTR2(h)	1.0E-05	1.0E-05	6.68	1.8E-08
ALK(h)	1.0E-05	1.0E-05	9.29	7.5E-08
ALK1(h)	1.0E-05	1.0E-05	2.39	2.8E-08
ALK2(h)	1.0E-05	1.0E-05	4.53	6.2E-08
ALK4(h)	1.0E-05	1.0E-05	3.05	1.6E-08
ALK6(h)	1.0E-05	1.0E-05	14.58	2.0E-08
AMPKalpha1(h)	1.0E-05	1.0E-05	11.21	5.3E-10
AMPKalpha2(h)	1.0E-05	1.0E-05	8.04	1.6E-09
A-Raf(h)	1.0E-05	1.0E-05	2.98	6.9E-09
Arg(h)	1.0E-05	1.0E-05	-7.9	2.1E-09
ARK5(h)	1.0E-05	1.0E-05	-0.18	1.8E-08
ASK1(h)	1.0E-05	1.0E-05	3.07	2.1E-07
Aurora-A(h)	1.0E-05	1.0E-05	21.23	1.1E-08
Aurora-B(h)	1.0E-05	1.0E-05	-14.46	3.2E-09
Aurora-C(h)	1.0E-05	1.0E-05	-2.87	2.9E-09
Axl(h)	1.0E-05	1.0E-05	-0.59	1.3E-07
BIKe(h)	1.0E-05	1.0E-05	7.79	1.2E-08
Blk(h)	1.0E-05	1.0E-05	-2.02	1.5E-08
BMPR2(h)	1.0E-05	1.0E-05	2.11	7.2E-08
Bmx(h)	1.0E-05	1.0E-05	22.94	2.5E-08
B-Raf(h)	1.0E-05	1.0E-05	-7.19	8.6E-11
BRK(h)	1.0E-05	1.0E-05	4.3	3.7E-08
BrSK1(h)	1.0E-05	1.0E-05	-2.96	6.1E-09
BrSK2(h)	1.0E-05	1.0E-05	-0.32	2.3E-08
BTK(h)	1.0E-05	1.0E-05	7.26	1.6E-08
CaMKI(h)	1.0E-05	1.0E-05	0.22	1.5E-08
CaMKIbeta(h)	1.0E-05	1.0E-05	1.26	8.0E-09
CaMKIdelta(h)	1.0E-05	1.0E-05	-9.34	4.4E-10
CaMKIgamma(h)	1.0E-05	1.0E-05	10.46	1.5E-09
CaMKIIalpha(h)	1.0E-05	1.0E-05	-4.14	3.5E-10

CaMKIIbeta(h)	1.0E-05	1.0E-05	7.19	3.0E-10
CaMKIIdelta(h)	1.0E-05	1.0E-05	-6.77	2.2E-10
CaMKIIgamma(h)	1.0E-05	1.0E-05	5.77	1.6E-10
CaMKIV(h)	1.0E-05	1.0E-05	-9.78	5.3E-09
CaMKK1(h)	1.0E-05	1.0E-05	-4.16	9.7E-08
CaMKK2(h)	1.0E-05	1.0E-05	3.81	3.5E-08
Cdc7/cyclinB1(h)	1.0E-05	1.0E-05	6.32	5.7E-08
CDK1/cyclinB(h)	1.0E-05	1.0E-05	1.6	3.5E-09
CDK12/cyclinK(h)	1.0E-05	1.0E-05	-5.64	3.2E-09
CDK13/cyclinK(h)	1.0E-05	1.0E-05	2.39	1.0E-08
CDK14/cyclinY(h)	1.0E-05	1.0E-05	0.61	1.2E-08
CDK16/cyclinY(h)	1.0E-05	1.0E-05	2.36	3.6E-10
CDK17/cyclinY(h)	1.0E-05	1.0E-05	9.31	7.7E-10
CDK18/cyclinY(h)	1.0E-05	1.0E-05	3.5	3.5E-08
CDK2/cyclinA(h)	1.0E-05	1.0E-05	-20.11	6.5E-09
CDK2/cyclinE(h)	1.0E-05	1.0E-05	-7.88	5.6E-09
CDK3/cyclinE(h)	1.0E-05	1.0E-05	-8.85	3.7E-08
CDK4/cyclinD3(h)	1.0E-05	1.0E-05	3.86	9.8E-08
CDK5/p25(h)	1.0E-05	1.0E-05	-2.47	6.2E-10
CDK5/p35(h)	1.0E-05	1.0E-05	-0.85	4.5E-09
CDK6/cyclinD3(h)	1.0E-05	1.0E-05	-12.45	1.4E-07
CDK7/cyclinH/MAT1(h)	1.0E-05	1.0E-05	4.08	4.2E-08
CDK9/cyclin T1(h)	1.0E-05	1.0E-05	-4.38	3.6E-08
CDKL1(h)	1.0E-05	1.0E-05	10.13	1.6E-07
CDKL2(h)	1.0E-05	1.0E-05	8.38	9.7E-08
CDKL3(h)	1.0E-05	1.0E-05	-1.25	6.3E-08
CDKL4(h)	1.0E-05	1.0E-05	0.71	4.1E-08
ChaK1(h)	1.0E-05	1.0E-05	-12.95	2.8E-09
CHK1(h)	1.0E-05	1.0E-05	-13.86	2.1E-08
CHK2(h)	1.0E-05	1.0E-05	4.79	4.5E-09
CK1alpha(h)	1.0E-05	1.0E-05	7.91	6.0E-10
CK1delta(h)	1.0E-05	1.0E-05	9.09	1.2E-09

CK1epsilon(h)	1.0E-05	1.0E-05	9.53	5.1E-10
CK1gamma1(h)	1.0E-05	1.0E-05	8.24	2.8E-10
CK1gamma2(h)	1.0E-05	1.0E-05	9.63	5.3E-10
CK1gamma3(h)	1.0E-05	1.0E-05	8	3.0E-10
CK2(h)	1.0E-05	1.0E-05	1.69	8.2E-10
CK2alpha1(h)	1.0E-05	1.0E-05	7.38	6.5E-10
CK2alpha2(h)	1.0E-05	1.0E-05	-3	1.5E-09
cKit(h)	1.0E-05	1.0E-05	-0.55	1.0E-06
CLIK1(h)	1.0E-05	1.0E-05	6.36	1.6E-07
CLK1(h)	1.0E-05	1.0E-05	8.38	2.9E-08
CLK2(h)	1.0E-05	1.0E-05	-2.03	2.8E-09
CLK3(h)	1.0E-05	1.0E-05	-15.65	8.2E-09
CLK4(h)	1.0E-05	1.0E-05	0.91	6.5E-08
c-RAF(h)	1.0E-05	1.0E-05	-9.81	2.5E-10
CRIK(h)	1.0E-05	1.0E-05	5.6	3.7E-09
CSK(h)	1.0E-05	1.0E-05	-13.91	2.1E-08
cSRC(h)	1.0E-05	1.0E-05	17.77	9.1E-09
DAPK1(h)	1.0E-05	1.0E-05	0.57	1.5E-09
DAPK2(h)	1.0E-05	1.0E-05	1.79	1.3E-08
DCAMKL1(h)	1.0E-05	1.0E-05	6.25	1.9E-08
DCAMKL2(h)	1.0E-05	1.0E-05	-0.9	1.7E-09
DCAMKL3(h)	1.0E-05	1.0E-05	4.88	2.3E-09
DDR1(h)	1.0E-05	1.0E-05	-6.82	2.6E-08
DDR2(h)	1.0E-05	1.0E-05	-4.1	2.9E-07
DMPK(h)	1.0E-05	1.0E-05	4.5	2.5E-07
DRAK1(h)	1.0E-05	1.0E-05	-23.38	4.1E-08
DRAK2(h)	1.0E-05	1.0E-05	17.58	2.2E-08
DYRK1A(h)	1.0E-05	1.0E-05	-2.65	1.6E-09
DYRK1B(h)	1.0E-05	1.0E-05	2.8	1.4E-09
DYRK2(h)	1.0E-05	1.0E-05	4.51	7.8E-09
DYRK3(h)	1.0E-05	1.0E-05	-6.78	1.4E-09
eEF-2K(h)	1.0E-05	1.0E-05	-5.77	2.8E-09

EGFR(h)	1.0E-05	1.0E-05	-15.57	1.4E-08
EphA1(h)	1.0E-05	1.0E-05	4.63	2.0E-07
EphA2(h)	1.0E-05	1.0E-05	7.27	2.7E-08
EphA3(h)	1.0E-05	1.0E-05	-9.71	1.2E-07
EphA4(h)	1.0E-05	1.0E-05	5.39	6.6E-08
EphA5(h)	1.0E-05	1.0E-05	6.76	8.7E-09
EphA7(h)	1.0E-05	1.0E-05	-1.14	1.6E-07
EphA8(h)	1.0E-05	1.0E-05	1.36	1.1E-07
EphB1(h)	1.0E-05	1.0E-05	-31.48	1.9E-08
EphB2(h)	1.0E-05	1.0E-05	-5.06	2.8E-08
EphB3(h)	1.0E-05	1.0E-05	-3.42	2.0E-07
EphB4(h)	1.0E-05	1.0E-05	-25.63	1.5E-08
ErbB2(h)	1.0E-05	1.0E-05	-7.69	1.3E-08
ErbB4(h)	1.0E-05	1.0E-05	-3.07	7.3E-09
FAK(h)	1.0E-05	1.0E-05	-2.96	1.2E-07
Fer(h)	1.0E-05	1.0E-05	14.05	7.1E-08
Fes(h)	1.0E-05	1.0E-05	8.18	1.2E-08
FGFR1(h)	1.0E-05	1.0E-05	-3.5	2.2E-08
FGFR2(h)	1.0E-05	1.0E-05	-17.39	1.5E-09
FGFR3(h)	1.0E-05	1.0E-05	19.42	6.1E-09
FGFR4(h)	1.0E-05	1.0E-05	-10.45	1.3E-08
Fgr(h)	1.0E-05	1.0E-05	-3.78	2.4E-09
Flt1(h)	1.0E-05	1.0E-05	-5.51	8.7E-08
Flt3(h)	1.0E-05	1.0E-05	0.65	3.0E-08
Flt4(h)	1.0E-05	1.0E-05	-10.5	8.2E-08
Fms(h)	1.0E-05	1.0E-05	3.41	1.3E-07
Fyn(h)	1.0E-05	1.0E-05	-2.08	5.2E-09
GCK (h)	1.0E-05	1.0E-05	11.02	4.7E-09
GCN2(h)	1.0E-05	1.0E-05	1.83	1.1E-08
GRK1(h)	1.0E-05	1.0E-05	-7.18	1.4E-08
GRK2(h)	1.0E-05	1.0E-05	5.48	1.4E-08
GRK3(h)	1.0E-05	1.0E-05	1.85	1.9E-08

GRK5(h)	1.0E-05	1.0E-05	-6.49	1.4E-08
GRK6(h)	1.0E-05	1.0E-05	4.94	4.3E-08
GRK7(h)	1.0E-05	1.0E-05	-0.84	1.4E-08
GSK3alpha(h)	1.0E-05	1.0E-05	10.99	8.4E-09
GSK3beta(h)	1.0E-05	1.0E-05	6.52	6.2E-09
Haspin(h)	1.0E-05	1.0E-05	9.87	1.7E-08
Hck(h)	1.0E-05	1.0E-05	15.31	1.9E-08
Hck(h) activated	1.0E-05	1.0E-05	-0.71	1.6E-10
HIPK1(h)	1.0E-05	1.0E-05	8.51	5.5E-09
HIPK2(h)	1.0E-05	1.0E-05	11.48	8.5E-10
HIPK3(h)	1.0E-05	1.0E-05	2.03	3.3E-09
HIPK4(h)	1.0E-05	1.0E-05	7.73	2.5E-09
HPK1(h)	1.0E-05	1.0E-05	10.96	6.5E-09
HRI(h)	1.0E-05	1.0E-05	-14.51	9.0E-08
ICK(h)	1.0E-05	1.0E-05	29.69	3.1E-08
IGF-1R(h)	1.0E-05	1.0E-05	-23.65	3.8E-07
IGF-1R(h), activated	1.0E-05	1.0E-05	-2.37	3.0E-09
IKKalpha(h)	1.0E-05	1.0E-05	-10.41	1.5E-08
IKKbeta(h)	1.0E-05	1.0E-05	-4.66	3.3E-07
IKKepsilon(h)	1.0E-05	1.0E-05	-4.62	2.6E-09
IR(h)	1.0E-05	1.0E-05	-5.67	5.2E-07
IR(h), activated	1.0E-05	1.0E-05	12.89	1.8E-09
IRAK1(h)	1.0E-05	1.0E-05	-2.62	8.8E-09
IRAK4(h)	1.0E-05	1.0E-05	-5.95	1.2E-08
IRE1(h)	1.0E-05	1.0E-05	-8.46	1.2E-08
IRR(h)	1.0E-05	1.0E-05	-2.02	5.2E-07
Itk(h)	1.0E-05	1.0E-05	-2.46	1.3E-07
JAK1(h)	1.0E-05	1.0E-05	5.61	2.0E-08
JAK2(h)	1.0E-05	1.0E-05	-3.89	4.8E-09
JAK3(h)	1.0E-05	1.0E-05	4.23	3.7E-09
JNK1alpha1(h)	1.0E-05	1.0E-05	9.47	1.7E-07
JNK2alpha2(h)	1.0E-05	1.0E-05	4.4	1.4E-07

JNK3(h)	1.0E-05	1.0E-05	8.85	2.6E-08
KDR(h)	1.0E-05	1.0E-05	15.88	3.2E-08
LATS1(h)	1.0E-05	1.0E-05	21.93	2.2E-08
LATS2(h)	1.0E-05	1.0E-05	15.24	8.5E-09
Lck(h)	1.0E-05	1.0E-05	3.09	3.0E-08
Lck(h) activated	1.0E-05	1.0E-05	0.11	3.0E-09
LIMK1(h)	1.0E-05	1.0E-05	-2.38	1.4E-08
LIMK2(h)	1.0E-05	1.0E-05	2.4	4.9E-08
LKB1(h)	1.0E-05	1.0E-05	0.83	7.5E-08
LOK(h)	1.0E-05	1.0E-05	-0.83	2.5E-08
LRRK2(h)	1.0E-05	1.0E-05	-3.23	1.9E-08
LTK(h)	1.0E-05	1.0E-05	-1.54	5.9E-08
Lyn(h)	1.0E-05	1.0E-05	-1.62	5.0E-09
MAK(h)	1.0E-05	1.0E-05	2.05	4.5E-08
MAP4K3(h)	1.0E-05	1.0E-05	1.35	5.0E-09
MAP4K4(h)	1.0E-05	1.0E-05	8.2	6.1E-09
MAP4K5(h)	1.0E-05	1.0E-05	-2.39	2.0E-09
MAPK1(h)	1.0E-05	1.0E-05	6	9.3E-09
MAPK2(h)	1.0E-05	1.0E-05	7.68	1.2E-08
MAPKAP-K2(h)	1.0E-05	1.0E-05	5.92	5.4E-09
MAPKAP-K3(h)	1.0E-05	1.0E-05	12.63	3.6E-09
MARK1(h)	1.0E-05	1.0E-05	12.61	1.4E-09
MARK3(h)	1.0E-05	1.0E-05	0.57	1.9E-09
MARK4(h)	1.0E-05	1.0E-05	9.89	2.2E-09
MEK1(h)	1.0E-05	1.0E-05	-0.02	2.3E-09
MEK2(h)	1.0E-05	1.0E-05	5.61	8.7E-10
MEKK2(h)	1.0E-05	1.0E-05	4.74	3.4E-08
MEKK3(h)	1.0E-05	1.0E-05	1.98	1.9E-08
MELK(h)	1.0E-05	1.0E-05	8.28	2.5E-09
Mer(h)	1.0E-05	1.0E-05	5.4	5.4E-10
Met(h)	1.0E-05	1.0E-05	12.58	8.7E-09
MINK(h)	1.0E-05	1.0E-05	1.61	2.4E-08

MKK3(h)	1.0E-05	1.0E-05	4.95	6.5E-09
MKK6(h)	1.0E-05	1.0E-05	9.24	9.3E-09
MLCK(h)	1.0E-05	1.0E-05	-4.41	2.7E-08
MLK1(h)	1.0E-05	1.0E-05	1.96	1.7E-08
MLK2(h)	1.0E-05	1.0E-05	10.22	1.6E-08
MLK3(h)	1.0E-05	1.0E-05	-2.48	1.2E-08
MLK4(h)	1.0E-05	1.0E-05	5.22	7.8E-09
Mnk2(h)	1.0E-05	1.0E-05	10.09	8.8E-08
MOK(h)	1.0E-05	1.0E-05	7.14	4.6E-09
MRCKalpha(h)	1.0E-05	1.0E-05	-0.05	1.1E-08
MRCKbeta(h)	1.0E-05	1.0E-05	6.66	1.9E-08
MRCKgamma(h)	1.0E-05	1.0E-05	5.33	3.8E-09
MSK1(h)	1.0E-05	1.0E-05	6.32	3.0E-08
MSK2(h)	1.0E-05	1.0E-05	15.15	7.5E-08
MSSK1(h)	1.0E-05	1.0E-05	8.63	1.8E-08
MST1(h)	1.0E-05	1.0E-05	-6.64	4.5E-09
MST2(h)	1.0E-05	1.0E-05	4.72	5.1E-09
MST3(h)	1.0E-05	1.0E-05	5.19	3.2E-08
MST4(h)	1.0E-05	1.0E-05	6.75	2.0E-08
mTOR FKBP12(h)	1.0E-05	1.0E-05	2.46	4.9E-07
mTOR(h)	1.0E-05	1.0E-05	0.96	1.0E-08
MuSK(h)	1.0E-05	1.0E-05	6.14	1.5E-06
MYLK2(h)	1.0E-05	1.0E-05	4.38	2.4E-09
MYO3B(h)	1.0E-05	1.0E-05	2.08	1.3E-08
NDR1(h)	1.0E-05	1.0E-05	5.83	3.3E-09
NDR2(h)	1.0E-05	1.0E-05	10.8	9.7E-08
NEK1(h)	1.0E-05	1.0E-05	13.01	2.6E-08
NEK11(h)	1.0E-05	1.0E-05	-2.03	2.9E-08
NEK2(h)	1.0E-05	1.0E-05	-5.96	4.3E-08
NEK3(h)	1.0E-05	1.0E-05	14.31	1.2E-07
NEK4(h)	1.0E-05	1.0E-05	3.5	1.8E-08
NEK6(h)	1.0E-05	1.0E-05	-4.86	1.6E-07

NEK7(h)	1.0E-05	1.0E-05	0.69	2.9E-07
NEK9(h)	1.0E-05	1.0E-05	2.31	1.9E-09
NIM1(h)	1.0E-05	1.0E-05	2.28	5.3E-09
NLK (h)	1.0E-05	1.0E-05	7.16	3.4E-07
NUAK2(h)	1.0E-05	1.0E-05	41.02	2.9E-07
OSR1(h)	1.0E-05	1.0E-05	-11.17	3.6E-09
p70S6K(h)	1.0E-05	1.0E-05	2.52	2.1E-08
PAK1(h)	1.0E-05	1.0E-05	-1.95	9.4E-11
PAK2(h)	1.0E-05	1.0E-05	2.68	2.3E-09
PAK3(h)	1.0E-05	1.0E-05	-2.47	3.9E-09
PAK4(h)	1.0E-05	1.0E-05	-8.93	1.5E-07
PAK5(h)	1.0E-05	1.0E-05	8.53	8.7E-10
PAK6(h)	1.0E-05	1.0E-05	6.95	1.2E-09
PAR-1Balpha(h)	1.0E-05	1.0E-05	-8.98	1.2E-09
PASK(h)	1.0E-05	1.0E-05	15.37	5.2E-09
PDGFRalpha(h)	1.0E-05	1.0E-05	16.46	2.9E-07
PDGFRbeta(h)	1.0E-05	1.0E-05	6.52	3.3E-07
PDHK2(h)	1.0E-05	1.0E-05	10.33	1.3E-07
PDHK4(h)	1.0E-05	1.0E-05	6.24	8.4E-08
PDK1(h)	1.0E-05	1.0E-05	11.39	2.3E-08
PEK(h)	1.0E-05	1.0E-05	-0.12	8.0E-09
PhKgamma1(h)	1.0E-05	1.0E-05	10.46	1.2E-08
PhKgamma2(h)	1.0E-05	1.0E-05	5.66	3.7E-08
Pim-1(h)	1.0E-05	1.0E-05	15.07	4.5E-09
Pim-2(h)	1.0E-05	1.0E-05	5.57	6.5E-09
Pim-3(h)	1.0E-05	1.0E-05	2.4	3.4E-09
PKA(h)	1.0E-05	1.0E-05	2.87	5.5E-10
PKACbeta(h)	1.0E-05	1.0E-05	15.05	7.9E-10
PKBalpha(h)	1.0E-05	1.0E-05	10.69	1.1E-08
PKBbeta(h)	1.0E-05	1.0E-05	6.13	1.5E-07
PKBgamma(h)	1.0E-05	1.0E-05	-0.64	1.4E-09
PKCalpha(h)	1.0E-05	1.0E-05	-0.68	1.6E-09

PKCbetaI(h)	1.0E-05	1.0E-05	-0.63	6.0E-10
PKCbetaII(h)	1.0E-05	1.0E-05	0.32	4.4E-10
PKCdelta(h)	1.0E-05	1.0E-05	7.71	5.5E-09
PKCepsilon(h)	1.0E-05	1.0E-05	21.11	4.3E-09
PKCeta(h)	1.0E-05	1.0E-05	19.36	8.7E-09
PKCgamma(h)	1.0E-05	1.0E-05	-0.1	1.4E-08
PKCiota(h)	1.0E-05	1.0E-05	0.81	2.8E-09
PKCmu(h)	1.0E-05	1.0E-05	0.41	5.3E-09
PKCtheta(h)	1.0E-05	1.0E-05	-12.64	4.0E-08
PKCzeta(h)	1.0E-05	1.0E-05	6.74	7.8E-09
PKD2(h)	1.0E-05	1.0E-05	8.25	2.0E-08
PKD3(h)	1.0E-05	1.0E-05	-7.09	7.3E-09
PKG1alpha(h)	1.0E-05	1.0E-05	14.66	7.5E-11
PKG1beta(h)	1.0E-05	1.0E-05	0.39	1.7E-09
PKR(h)	1.0E-05	1.0E-05	6.22	4.9E-09
Plk1(h)	1.0E-05	1.0E-05	1.06	2.2E-08
Plk3(h)	1.0E-05	1.0E-05	-8.23	1.2E-08
Plk4(h)	1.0E-05	1.0E-05	-10.75	1.6E-08
PRAK(h)	1.0E-05	1.0E-05	58.25	2.4E-08
PRK1(h)	1.0E-05	1.0E-05	2.46	3.0E-08
PRK2(h)	1.0E-05	1.0E-05	4.47	1.7E-08
PRKG2(h)	1.0E-05	1.0E-05	-1.61	1.7E-09
PrKX(h)	1.0E-05	1.0E-05	22.58	2.1E-08
PRP4(h)	1.0E-05	1.0E-05	8.18	1.2E-07
PTK5(h)	1.0E-05	1.0E-05	-5.66	1.5E-08
Pyk2(h)	1.0E-05	1.0E-05	9.9	1.6E-07
Ret(h)	1.0E-05	1.0E-05	-7.7	4.3E-09
RIPK1(h)	1.0E-05	1.0E-05	-2.93	2.6E-07
RIPK2(h)	1.0E-05	1.0E-05	0.2	2.1E-08
ROCK-I(h)	1.0E-05	1.0E-05	3.01	5.5E-08
ROCK-II(h)	1.0E-05	1.0E-05	2.99	4.1E-09
Ron(h)	1.0E-05	1.0E-05	2.31	2.2E-08

Ros(h)	1.0E-05	1.0E-05	0.5	2.3E-08
Rse(h)	1.0E-05	1.0E-05	13.68	1.5E-08
Rsk1(h)	1.0E-05	1.0E-05	-5.69	1.2E-09
Rsk2(h)	1.0E-05	1.0E-05	-2.95	2.3E-09
Rsk3(h)	1.0E-05	1.0E-05	-1.16	1.3E-08
Rsk4(h)	1.0E-05	1.0E-05	9.03	4.0E-09
SAPK2a(h)	1.0E-05	1.0E-05	2.13	1.7E-07
SAPK2b(h)	1.0E-05	1.0E-05	8.08	7.0E-09
SAPK3(h)	1.0E-05	1.0E-05	4.27	4.2E-08
SAPK4(h)	1.0E-05	1.0E-05	-2.62	7.0E-09
SBK1(h)	1.0E-05	1.0E-05	17.34	1.5E-08
SGK(h)	1.0E-05	1.0E-05	-0.2	3.3E-09
SGK2(h)	1.0E-05	1.0E-05	6.44	1.7E-07
SGK3(h)	1.0E-05	1.0E-05	6.43	3.4E-08
SIK(h)	1.0E-05	1.0E-05	9.31	6.5E-08
SIK2(h)	1.0E-05	1.0E-05	7.78	2.0E-08
SIK3(h)	1.0E-05	1.0E-05	9.94	6.3E-09
SLK(h)	1.0E-05	1.0E-05	6.3	2.7E-10
Snk(h)	1.0E-05	1.0E-05	-1.49	1.8E-07
SNRK(h)	1.0E-05	1.0E-05	8.39	4.8E-08
SRMS(h)	1.0E-05	1.0E-05	20.04	6.3E-09
SRPK1(h)	1.0E-05	1.0E-05	-23.25	8.5E-10
SRPK2(h)	1.0E-05	1.0E-05	9.6	5.2E-10
STK16(h)	1.0E-05	1.0E-05	-9.06	4.9E-08
STK25(h)	1.0E-05	1.0E-05	12.25	1.0E-08
STK32A(h)	1.0E-05	1.0E-05	1.52	3.2E-08
STK32B(h)	1.0E-05	1.0E-05	10.52	3.0E-09
STK32C(h)	1.0E-05	1.0E-05	2.45	4.7E-08
STK33(h)	1.0E-05	1.0E-05	1.36	1.9E-07
STK39(h)	1.0E-05	1.0E-05	-2.66	1.8E-09
Syk(h)	1.0E-05	1.0E-05	21.09	1.7E-08
TAF1L(h)	1.0E-05	1.0E-05	8.62	3.7E-08

TAK1(h)	1.0E-05	1.0E-05	-6.84	3.2E-08
TAO1(h)	1.0E-05	1.0E-05	4.17	4.0E-09
TAO2(h)	1.0E-05	1.0E-05	-3.58	4.7E-09
TAO3(h)	1.0E-05	1.0E-05	7.12	6.9E-09
TBK1(h)	1.0E-05	1.0E-05	-1.33	1.9E-08
Tec(h) activated	1.0E-05	1.0E-05	12.65	4.0E-09
TGFBR1(h)	1.0E-05	1.0E-05	7.47	8.4E-09
TGFBR2(h)	1.0E-05	1.0E-05	2.48	3.1E-08
Tie2(h)	1.0E-05	1.0E-05	-3.39	1.9E-07
TLK1(h)	1.0E-05	1.0E-05	4.27	1.5E-09
TLK2(h)	1.0E-05	1.0E-05	8.77	6.8E-09
TNIK(h)	1.0E-05	1.0E-05	18.38	8.7E-09
TRB2(h)	1.0E-05	1.0E-05	3.59	5.9E-08
TrkA(h)	1.0E-05	1.0E-05	56.88	6.3E-08
TrkB(h)	1.0E-05	1.0E-05	10.15	1.7E-07
TrkC(h)	1.0E-05	1.0E-05	9.76	7.3E-08
TSSK1(h)	1.0E-05	1.0E-05	4.75	8.6E-09
TSSK2(h)	1.0E-05	1.0E-05	15.11	6.1E-09
TSSK3(h)	1.0E-05	1.0E-05	24.48	1.4E-08
TSSK4(h)	1.0E-05	1.0E-05	27.76	1.8E-08
TTBK1(h)	1.0E-05	1.0E-05	-12.77	3.0E-09
TTBK2(h)	1.0E-05	1.0E-05	-10.08	3.1E-10
TTK(h)	1.0E-05	1.0E-05	5.09	4.5E-08
Txk(h)	1.0E-05	1.0E-05	-39.18	1.1E-08
TYK2(h)	1.0E-05	1.0E-05	7.17	1.6E-09
ULK1(h)	1.0E-05	1.0E-05	6.45	2.3E-09
ULK2(h)	1.0E-05	1.0E-05	8.08	8.7E-09
ULK3(h)	1.0E-05	1.0E-05	5.11	6.5E-09
VRK1(h)	1.0E-05	1.0E-05	3.92	1.4E-07
VRK2(h)	1.0E-05	1.0E-05	6.47	1.4E-07
WEE1(h)	1.0E-05	1.0E-05	-6.82	2.5E-08
Wee1B(h)	1.0E-05	1.0E-05	7.44	7.5E-08

WNK1(h)	1.0E-05	1.0E-05	6.4	2.5E-08
WNK2(h)	1.0E-05	1.0E-05	-5.83	1.7E-07
WNK3(h)	1.0E-05	1.0E-05	-17.58	2.5E-07
WNK4(h)	1.0E-05	1.0E-05	9.12	1.7E-07
Yes(h)	1.0E-05	1.0E-05	12.45	8.8E-10
ZAK(h)	1.0E-05	1.0E-05	-1.56	4.2E-09
ZAP-70(h)	1.0E-05	1.0E-05	6.36	2.9E-08
ZIPK(h)	1.0E-05	1.0E-05	-0.39	1.9E-08
ATM(h)	1.0E-05	1.0E-05	1.1	2.13E-09
ATR/ATRIP(h)	1.0E-05	1.0E-05	-8.31	2.56E-09
DNA-PK(h)	1.0E-05	1.0E-05	-12.43	1.7E-10
PI3 Kinase (p110a/p85a)(h)	1.0E-05	1.0E-05	5.48	1.91E-10
PI3 Kinase (p110b/p85a)(h)	1.0E-05	1.0E-05	7.43	4.26E-10
PI3 Kinase (p110d/p85a)(h)	1.0E-05	1.0E-05	13.07	7.91E-10
PI3 Kinase (p120g)(h)	1.0E-05	1.0E-05	2.58	2.08E-09
PI3KC2a(h)	1.0E-05	1.0E-05	7.47	0.000000023
PI3KC2g(h)	1.0E-05	1.0E-05	-10.72	3.56E-08
PIP4K2a(h)	1.0E-05	1.0E-05	2.88	2.13E-08
PIP5K1a(h)	1.0E-05	1.0E-05	0.26	3.9E-09
PIP5K1g(h)	1.0E-05	1.0E-05	2.56	1.17E-08
ATM(h)	1.0E-05	1.0E-05	0.43	2.13E-09
ATR/ATRIP(h)	1.0E-05	1.0E-05	-1.09	2.56E-09
DNA-PK(h)	1.0E-05	1.0E-05	-5.83	1.7E-10
PI3 Kinase (p110a/p85a)(h)	1.0E-05	1.0E-05	1.56	1.91E-10
PI3 Kinase (p110b/p85a)(h)	1.0E-05	1.0E-05	15.38	4.26E-10
PI3 Kinase (p110d/p85a)(h)	1.0E-05	1.0E-05	21	7.91E-10
PI3 Kinase (p120g)(h)	1.0E-05	1.0E-05	3.07	2.08E-09
PI3KC2a(h)	1.0E-05	1.0E-05	5.4	0.000000023

PI3KC2g(h)	1.0E-05	1.0E-05	1.22	3.56E-08
PIP4K2a(h)	1.0E-05	1.0E-05	5.9	2.13E-08
PIP5K1a(h)	1.0E-05	1.0E-05	-1.73	3.9E-09
PIP5K1g(h)	1.0E-05	1.0E-05	5.15	1.17E-08

Table S13. Kinase Selectivity Panel (Eurofins) for BAY-728 (**22**) performed at 10 µM compound concentration. No significant response (> 50% inhibition) at 10 µM compound concentration was noted.

Kinase	ATP Conc. [mol/L]	Concentration [mol/L]	Inhibition [%]	Enzyme Conc. [mol/L]
AAK1(h)	1.0E-05	1.0E-05	3.69	1.3E-08
Abl(h)	1.0E-05	1.0E-05	-14.7	7.7E-10
ACK1(h)	1.0E-05	1.0E-05	-3.45	1.7E-08
ACTR2(h)	1.0E-05	1.0E-05	6.16	1.8E-08
ALK(h)	1.0E-05	1.0E-05	11.49	7.5E-08
ALK1(h)	1.0E-05	1.0E-05	-3.75	2.8E-08
ALK2(h)	1.0E-05	1.0E-05	5.05	6.2E-08
ALK4(h)	1.0E-05	1.0E-05	3.46	1.6E-08
ALK6(h)	1.0E-05	1.0E-05	26.77	2.0E-08
AMPKalpha1(h)	1.0E-05	1.0E-05	7.32	5.3E-10
AMPKalpha2(h)	1.0E-05	1.0E-05	-8.56	1.6E-09
A-Raf(h)	1.0E-05	1.0E-05	7.65	6.9E-09
Arg(h)	1.0E-05	1.0E-05	-13.7	2.1E-09
ARK5(h)	1.0E-05	1.0E-05	10.7	1.8E-08
ASK1(h)	1.0E-05	1.0E-05	2.31	2.1E-07
Aurora-A(h)	1.0E-05	1.0E-05	21.14	1.1E-08
Aurora-B(h)	1.0E-05	1.0E-05	-17.19	3.2E-09
Aurora-C(h)	1.0E-05	1.0E-05	-2.91	2.9E-09

Axl(h)	1.0E-05	1.0E-05	5.28	1.3E-07
BIKe(h)	1.0E-05	1.0E-05	-13.91	1.2E-08
Blk(h)	1.0E-05	1.0E-05	3.04	1.5E-08
BMPR2(h)	1.0E-05	1.0E-05	1.41	7.2E-08
Bmx(h)	1.0E-05	1.0E-05	16.06	2.5E-08
B-Raf(h)	1.0E-05	1.0E-05	-3.24	8.6E-11
BRK(h)	1.0E-05	1.0E-05	7.88	3.7E-08
BrSK1(h)	1.0E-05	1.0E-05	0.09	6.1E-09
BrSK2(h)	1.0E-05	1.0E-05	-5.3	2.3E-08
BTK(h)	1.0E-05	1.0E-05	0.23	1.6E-08
CaMKI(h)	1.0E-05	1.0E-05	-2.64	1.5E-08
CaMKIbeta(h)	1.0E-05	1.0E-05	-1.47	8.0E-09
CaMKIdelta(h)	1.0E-05	1.0E-05	-10.46	4.4E-10
CaMKIgamma(h)	1.0E-05	1.0E-05	10.94	1.5E-09
CaMKIIalpha(h)	1.0E-05	1.0E-05	0.18	3.5E-10
CaMKIIbeta(h)	1.0E-05	1.0E-05	1.16	3.0E-10
CaMKIIdelta(h)	1.0E-05	1.0E-05	-3.4	2.2E-10
CaMKIIgamma(h)	1.0E-05	1.0E-05	2.22	1.6E-10
CaMKIV(h)	1.0E-05	1.0E-05	-9.6	5.3E-09
CaMKK1(h)	1.0E-05	1.0E-05	-7.24	9.7E-08
CaMKK2(h)	1.0E-05	1.0E-05	5.63	3.5E-08
Cdc7/cyclinB1(h)	1.0E-05	1.0E-05	3.54	5.7E-08
CDK1/cyclinB(h)	1.0E-05	1.0E-05	-0.33	3.5E-09
CDK12/cyclinK(h)	1.0E-05	1.0E-05	9.09	3.2E-09
CDK13/cyclinK(h)	1.0E-05	1.0E-05	3.1	1.0E-08
CDK14/cyclinY(h)	1.0E-05	1.0E-05	0.44	1.2E-08
CDK16/cyclinY(h)	1.0E-05	1.0E-05	20.83	3.6E-10
CDK17/cyclinY(h)	1.0E-05	1.0E-05	6.25	7.7E-10
CDK18/cyclinY(h)	1.0E-05	1.0E-05	2.07	3.5E-08
CDK2/cyclinA(h)	1.0E-05	1.0E-05	-15.33	6.5E-09
CDK2/cyclinE(h)	1.0E-05	1.0E-05	-6.08	5.6E-09
CDK3/cyclinE(h)	1.0E-05	1.0E-05	-14.69	3.7E-08

CDK4/cyclinD3(h)	1.0E-05	1.0E-05	0.72	9.8E-08
CDK5/p25(h)	1.0E-05	1.0E-05	1.49	6.2E-10
CDK5/p35(h)	1.0E-05	1.0E-05	-11.6	4.5E-09
CDK6/cyclinD3(h)	1.0E-05	1.0E-05	-16.77	1.4E-07
CDK7/cyclinH/MAT1(h)	1.0E-05	1.0E-05	-2.13	4.2E-08
CDK9/cyclin T1(h)	1.0E-05	1.0E-05	-1.35	3.6E-08
CDKL1(h)	1.0E-05	1.0E-05	13.45	1.6E-07
CDKL2(h)	1.0E-05	1.0E-05	10.41	9.7E-08
CDKL3(h)	1.0E-05	1.0E-05	0.86	6.3E-08
CDKL4(h)	1.0E-05	1.0E-05	-4.09	4.1E-08
ChaK1(h)	1.0E-05	1.0E-05	-14.62	2.8E-09
CHK1(h)	1.0E-05	1.0E-05	-6.25	2.1E-08
CHK2(h)	1.0E-05	1.0E-05	-1.61	4.5E-09
CK1alpha(h)	1.0E-05	1.0E-05	5.47	6.0E-10
CK1delta(h)	1.0E-05	1.0E-05	-2.63	1.2E-09
CK1epsilon(h)	1.0E-05	1.0E-05	3.02	5.1E-10
CK1gamma1(h)	1.0E-05	1.0E-05	-1.52	2.8E-10
CK1gamma2(h)	1.0E-05	1.0E-05	5.69	5.3E-10
CK1gamma3(h)	1.0E-05	1.0E-05	2.37	3.0E-10
CK2(h)	1.0E-05	1.0E-05	-2.03	8.2E-10
CK2alpha1(h)	1.0E-05	1.0E-05	4.27	6.5E-10
CK2alpha2(h)	1.0E-05	1.0E-05	0.25	1.5E-09
cKit(h)	1.0E-05	1.0E-05	-4.4	1.0E-06
CLIK1(h)	1.0E-05	1.0E-05	-3.36	1.6E-07
CLK1(h)	1.0E-05	1.0E-05	1.92	2.9E-08
CLK2(h)	1.0E-05	1.0E-05	-11.52	2.8E-09
CLK3(h)	1.0E-05	1.0E-05	-16.15	8.2E-09
CLK4(h)	1.0E-05	1.0E-05	-7.17	6.5E-08
c-RAF(h)	1.0E-05	1.0E-05	-10.51	2.5E-10
CRIK(h)	1.0E-05	1.0E-05	6.31	3.7E-09
CSK(h)	1.0E-05	1.0E-05	-17.21	2.1E-08
cSRC(h)	1.0E-05	1.0E-05	18.04	9.1E-09

DAPK1(h)	1.0E-05	1.0E-05	0.15	1.5E-09
DAPK2(h)	1.0E-05	1.0E-05	25.2	1.3E-08
DCAMKL1(h)	1.0E-05	1.0E-05	-0.87	1.9E-08
DCAMKL2(h)	1.0E-05	1.0E-05	-15.08	1.7E-09
DCAMKL3(h)	1.0E-05	1.0E-05	4.5	2.3E-09
DDR1(h)	1.0E-05	1.0E-05	-7.03	2.6E-08
DDR2(h)	1.0E-05	1.0E-05	-7.41	2.9E-07
DMPK(h)	1.0E-05	1.0E-05	-0.97	2.5E-07
DRAK1(h)	1.0E-05	1.0E-05	-21.92	4.1E-08
DRAK2(h)	1.0E-05	1.0E-05	6.43	2.2E-08
DYRK1A(h)	1.0E-05	1.0E-05	-5.31	1.6E-09
DYRK1B(h)	1.0E-05	1.0E-05	-3.27	1.4E-09
DYRK2(h)	1.0E-05	1.0E-05	-1.89	7.8E-09
DYRK3(h)	1.0E-05	1.0E-05	-6.72	1.4E-09
eEF-2K(h)	1.0E-05	1.0E-05	1.23	2.8E-09
EGFR(h)	1.0E-05	1.0E-05	-21.6	1.4E-08
EphA1(h)	1.0E-05	1.0E-05	-8.87	2.0E-07
EphA2(h)	1.0E-05	1.0E-05	-2.44	2.7E-08
EphA3(h)	1.0E-05	1.0E-05	-27.21	1.2E-07
EphA4(h)	1.0E-05	1.0E-05	9.3	6.6E-08
EphA5(h)	1.0E-05	1.0E-05	-2.12	8.7E-09
EphA7(h)	1.0E-05	1.0E-05	4.64	1.6E-07
EphA8(h)	1.0E-05	1.0E-05	-0.72	1.1E-07
EphB1(h)	1.0E-05	1.0E-05	4.2	1.9E-08
EphB2(h)	1.0E-05	1.0E-05	-7.93	2.8E-08
EphB3(h)	1.0E-05	1.0E-05	-2.88	2.0E-07
EphB4(h)	1.0E-05	1.0E-05	-37.79	1.5E-08
ErbB2(h)	1.0E-05	1.0E-05	-8.06	1.3E-08
ErbB4(h)	1.0E-05	1.0E-05	-3.69	7.3E-09
FAK(h)	1.0E-05	1.0E-05	-23.59	1.2E-07
Fer(h)	1.0E-05	1.0E-05	0.43	7.1E-08
Fes(h)	1.0E-05	1.0E-05	1.46	1.2E-08

FGFR1(h)	1.0E-05	1.0E-05	2.15	2.2E-08
FGFR2(h)	1.0E-05	1.0E-05	-9.39	1.5E-09
FGFR3(h)	1.0E-05	1.0E-05	21.17	6.1E-09
FGFR4(h)	1.0E-05	1.0E-05	-10.5	1.3E-08
Fgr(h)	1.0E-05	1.0E-05	10.86	2.4E-09
Flt1(h)	1.0E-05	1.0E-05	-10.66	8.7E-08
Flt3(h)	1.0E-05	1.0E-05	8.17	3.0E-08
Flt4(h)	1.0E-05	1.0E-05	-8.37	8.2E-08
Fms(h)	1.0E-05	1.0E-05	0.62	1.3E-07
Fyn(h)	1.0E-05	1.0E-05	1.56	5.2E-09
GCK (h)	1.0E-05	1.0E-05	8.31	4.7E-09
GCN2(h)	1.0E-05	1.0E-05	4.09	1.1E-08
GRK1(h)	1.0E-05	1.0E-05	-1.62	1.4E-08
GRK2(h)	1.0E-05	1.0E-05	-0.15	1.4E-08
GRK3(h)	1.0E-05	1.0E-05	3.53	1.9E-08
GRK5(h)	1.0E-05	1.0E-05	-1.71	1.4E-08
GRK6(h)	1.0E-05	1.0E-05	6.11	4.3E-08
GRK7(h)	1.0E-05	1.0E-05	-4.53	1.4E-08
GSK3alpha(h)	1.0E-05	1.0E-05	-3.55	8.4E-09
GSK3beta(h)	1.0E-05	1.0E-05	3.68	6.2E-09
Haspin(h)	1.0E-05	1.0E-05	10.96	1.7E-08
Hck(h)	1.0E-05	1.0E-05	4.11	1.9E-08
Hck(h) activated	1.0E-05	1.0E-05	7.68	1.6E-10
HIPK1(h)	1.0E-05	1.0E-05	11.91	5.5E-09
HIPK2(h)	1.0E-05	1.0E-05	0.7	8.5E-10
HIPK3(h)	1.0E-05	1.0E-05	-2.65	3.3E-09
HIPK4(h)	1.0E-05	1.0E-05	6.43	2.5E-09
HPK1(h)	1.0E-05	1.0E-05	0.4	6.5E-09
HRI(h)	1.0E-05	1.0E-05	-10.68	9.0E-08
ICK(h)	1.0E-05	1.0E-05	6.32	3.1E-08
IGF-1R(h)	1.0E-05	1.0E-05	-24.39	3.8E-07
IGF-1R(h), activated	1.0E-05	1.0E-05	3.24	3.0E-09

IKKalpha(h)	1.0E-05	1.0E-05	-8.97	1.5E-08
IKKbeta(h)	1.0E-05	1.0E-05	6.11	3.3E-07
IKKepsilon(h)	1.0E-05	1.0E-05	-10.56	2.6E-09
IR(h)	1.0E-05	1.0E-05	-7.1	5.2E-07
IR(h), activated	1.0E-05	1.0E-05	-1.5	1.8E-09
IRAK1(h)	1.0E-05	1.0E-05	-2.41	8.8E-09
IRAK4(h)	1.0E-05	1.0E-05	-10.37	1.2E-08
IRE1(h)	1.0E-05	1.0E-05	-7.86	1.2E-08
IRR(h)	1.0E-05	1.0E-05	3.88	5.2E-07
Itk(h)	1.0E-05	1.0E-05	-6.13	1.3E-07
JAK1(h)	1.0E-05	1.0E-05	1.11	2.0E-08
JAK2(h)	1.0E-05	1.0E-05	-2.9	4.8E-09
JAK3(h)	1.0E-05	1.0E-05	3.97	3.7E-09
JNK1alpha1(h)	1.0E-05	1.0E-05	-0.15	1.7E-07
JNK2alpha2(h)	1.0E-05	1.0E-05	6.67	1.4E-07
JNK3(h)	1.0E-05	1.0E-05	-3.97	2.6E-08
KDR(h)	1.0E-05	1.0E-05	11.88	3.2E-08
LATS1(h)	1.0E-05	1.0E-05	11.91	2.2E-08
LATS2(h)	1.0E-05	1.0E-05	4.04	8.5E-09
Lck(h)	1.0E-05	1.0E-05	-8.47	3.0E-08
Lck(h) activated	1.0E-05	1.0E-05	3.42	3.0E-09
LIMK1(h)	1.0E-05	1.0E-05	0.51	1.4E-08
LIMK2(h)	1.0E-05	1.0E-05	-0.4	4.9E-08
LKB1(h)	1.0E-05	1.0E-05	3.08	7.5E-08
LOK(h)	1.0E-05	1.0E-05	-13.12	2.5E-08
LRRK2(h)	1.0E-05	1.0E-05	-2.52	1.9E-08
LTK(h)	1.0E-05	1.0E-05	-4.59	5.9E-08
Lyn(h)	1.0E-05	1.0E-05	3.48	5.0E-09
MAK(h)	1.0E-05	1.0E-05	-2.88	4.5E-08
MAP4K3(h)	1.0E-05	1.0E-05	-1.13	5.0E-09
MAP4K4(h)	1.0E-05	1.0E-05	8.87	6.1E-09
MAP4K5(h)	1.0E-05	1.0E-05	-3.65	2.0E-09

MAPK1(h)	1.0E-05	1.0E-05	-0.7	9.3E-09
MAPK2(h)	1.0E-05	1.0E-05	4.81	1.2E-08
MAPKAP-K2(h)	1.0E-05	1.0E-05	-6.72	5.4E-09
MAPKAP-K3(h)	1.0E-05	1.0E-05	5.63	3.6E-09
MARK1(h)	1.0E-05	1.0E-05	8.84	1.4E-09
MARK3(h)	1.0E-05	1.0E-05	2.14	1.9E-09
MARK4(h)	1.0E-05	1.0E-05	3.52	2.2E-09
MEK1(h)	1.0E-05	1.0E-05	1.44	2.3E-09
MEK2(h)	1.0E-05	1.0E-05	4.53	8.7E-10
MEKK2(h)	1.0E-05	1.0E-05	-0.26	3.4E-08
MEKK3(h)	1.0E-05	1.0E-05	4.03	1.9E-08
MELK(h)	1.0E-05	1.0E-05	4.43	2.5E-09
Mer(h)	1.0E-05	1.0E-05	-0.22	5.4E-10
Met(h)	1.0E-05	1.0E-05	7.84	8.7E-09
MINK(h)	1.0E-05	1.0E-05	-12.35	2.4E-08
MKK3(h)	1.0E-05	1.0E-05	1.89	6.5E-09
MKK6(h)	1.0E-05	1.0E-05	-8.15	9.3E-09
MLCK(h)	1.0E-05	1.0E-05	-3.85	2.7E-08
MLK1(h)	1.0E-05	1.0E-05	-1.43	1.7E-08
MLK2(h)	1.0E-05	1.0E-05	8.77	1.6E-08
MLK3(h)	1.0E-05	1.0E-05	0	1.2E-08
MLK4(h)	1.0E-05	1.0E-05	-1.94	7.8E-09
Mnk2(h)	1.0E-05	1.0E-05	28.68	8.8E-08
MOK(h)	1.0E-05	1.0E-05	14.95	4.6E-09
MRCKalpha(h)	1.0E-05	1.0E-05	8.27	1.1E-08
MRCKbeta(h)	1.0E-05	1.0E-05	0.48	1.9E-08
MRCKgamma(h)	1.0E-05	1.0E-05	-2.61	3.8E-09
MSK1(h)	1.0E-05	1.0E-05	6.97	3.0E-08
MSK2(h)	1.0E-05	1.0E-05	3.68	7.5E-08
MSSK1(h)	1.0E-05	1.0E-05	-2.83	1.8E-08
MST1(h)	1.0E-05	1.0E-05	-4.69	4.5E-09
MST2(h)	1.0E-05	1.0E-05	-10.27	5.1E-09

MST3(h)	1.0E-05	1.0E-05	4.23	3.2E-08
MST4(h)	1.0E-05	1.0E-05	3.88	2.0E-08
mTOR FKBP12(h)	1.0E-05	1.0E-05	3	4.9E-07
mTOR(h)	1.0E-05	1.0E-05	-5.19	1.0E-08
MuSK(h)	1.0E-05	1.0E-05	1.15	1.5E-06
MYLK2(h)	1.0E-05	1.0E-05	2.95	2.4E-09
MYO3B(h)	1.0E-05	1.0E-05	3.59	1.3E-08
NDR1(h)	1.0E-05	1.0E-05	3.89	3.3E-09
NDR2(h)	1.0E-05	1.0E-05	-2.71	9.7E-08
NEK1(h)	1.0E-05	1.0E-05	13	2.6E-08
NEK11(h)	1.0E-05	1.0E-05	3.19	2.9E-08
NEK2(h)	1.0E-05	1.0E-05	-2.19	4.3E-08
NEK3(h)	1.0E-05	1.0E-05	9.85	1.2E-07
NEK4(h)	1.0E-05	1.0E-05	0.22	1.8E-08
NEK6(h)	1.0E-05	1.0E-05	-8.5	1.6E-07
NEK7(h)	1.0E-05	1.0E-05	-1.75	2.9E-07
NEK9(h)	1.0E-05	1.0E-05	-1.19	1.9E-09
NIM1(h)	1.0E-05	1.0E-05	1.06	5.3E-09
NLK (h)	1.0E-05	1.0E-05	-1.46	3.4E-07
NUAK2(h)	1.0E-05	1.0E-05	17.95	2.9E-07
OSR1(h)	1.0E-05	1.0E-05	-14.9	3.6E-09
p70S6K(h)	1.0E-05	1.0E-05	-3.21	2.1E-08
PAK1(h)	1.0E-05	1.0E-05	11.08	9.4E-11
PAK2(h)	1.0E-05	1.0E-05	0.75	2.3E-09
PAK3(h)	1.0E-05	1.0E-05	-0.66	3.9E-09
PAK4(h)	1.0E-05	1.0E-05	0.07	1.5E-07
PAK5(h)	1.0E-05	1.0E-05	12.83	8.7E-10
PAK6(h)	1.0E-05	1.0E-05	3.73	1.2E-09
PAR-1Balpha(h)	1.0E-05	1.0E-05	2.65	1.2E-09
PASK(h)	1.0E-05	1.0E-05	11.33	5.2E-09
PDGFRalpha(h)	1.0E-05	1.0E-05	29.9	2.9E-07
PDGFRbeta(h)	1.0E-05	1.0E-05	-3.59	3.3E-07

PDHK2(h)	1.0E-05	1.0E-05	4.73	1.3E-07
PDHK4(h)	1.0E-05	1.0E-05	-5.39	8.4E-08
PDK1(h)	1.0E-05	1.0E-05	9.31	2.3E-08
PEK(h)	1.0E-05	1.0E-05	1.95	8.0E-09
PhKgamma1(h)	1.0E-05	1.0E-05	6.48	1.2E-08
PhKgamma2(h)	1.0E-05	1.0E-05	9.2	3.7E-08
Pim-1(h)	1.0E-05	1.0E-05	12.59	4.5E-09
Pim-2(h)	1.0E-05	1.0E-05	-2.68	6.5E-09
Pim-3(h)	1.0E-05	1.0E-05	5.7	3.4E-09
PKA(h)	1.0E-05	1.0E-05	4.33	5.5E-10
PKACbeta(h)	1.0E-05	1.0E-05	8.25	7.9E-10
PKBalpha(h)	1.0E-05	1.0E-05	4.46	1.1E-08
PKBbeta(h)	1.0E-05	1.0E-05	9.96	1.5E-07
PKBgamma(h)	1.0E-05	1.0E-05	-0.27	1.4E-09
PKCalpha(h)	1.0E-05	1.0E-05	5.89	1.6E-09
PKCbetaI(h)	1.0E-05	1.0E-05	-1.7	6.0E-10
PKCbetaII(h)	1.0E-05	1.0E-05	0.91	4.4E-10
PKCdelta(h)	1.0E-05	1.0E-05	5.78	5.5E-09
PKCepsilon(h)	1.0E-05	1.0E-05	-0.61	4.3E-09
PKCeta(h)	1.0E-05	1.0E-05	23.68	8.7E-09
PKCgamma(h)	1.0E-05	1.0E-05	-2.02	1.4E-08
PKCiota(h)	1.0E-05	1.0E-05	2.1	2.8E-09
PKCmu(h)	1.0E-05	1.0E-05	-7.74	5.3E-09
PKCtheta(h)	1.0E-05	1.0E-05	-8.12	4.0E-08
PKCzeta(h)	1.0E-05	1.0E-05	-0.09	7.8E-09
PKD2(h)	1.0E-05	1.0E-05	0.72	2.0E-08
PKD3(h)	1.0E-05	1.0E-05	7.79	7.3E-09
PKG1alpha(h)	1.0E-05	1.0E-05	7.5	7.5E-11
PKG1beta(h)	1.0E-05	1.0E-05	3.46	1.7E-09
PKR(h)	1.0E-05	1.0E-05	3.56	4.9E-09
Plk1(h)	1.0E-05	1.0E-05	5.71	2.2E-08
Plk3(h)	1.0E-05	1.0E-05	-17.43	1.2E-08

Plk4(h)	1.0E-05	1.0E-05	-23.19	1.6E-08
PRAK(h)	1.0E-05	1.0E-05	39.11	2.4E-08
PRK1(h)	1.0E-05	1.0E-05	5.48	3.0E-08
PRK2(h)	1.0E-05	1.0E-05	9.51	1.7E-08
PRKG2(h)	1.0E-05	1.0E-05	3.67	1.7E-09
PrKX(h)	1.0E-05	1.0E-05	10.45	2.1E-08
PRP4(h)	1.0E-05	1.0E-05	-7.38	1.2E-07
PTK5(h)	1.0E-05	1.0E-05	-6.75	1.5E-08
Pyk2(h)	1.0E-05	1.0E-05	-5.98	1.6E-07
Ret(h)	1.0E-05	1.0E-05	1.67	4.3E-09
RIPK1(h)	1.0E-05	1.0E-05	0.89	2.6E-07
RIPK2(h)	1.0E-05	1.0E-05	6.19	2.1E-08
ROCK-I(h)	1.0E-05	1.0E-05	15.18	5.5E-08
ROCK-II(h)	1.0E-05	1.0E-05	-4.94	4.1E-09
Ron(h)	1.0E-05	1.0E-05	-5.2	2.2E-08
Ros(h)	1.0E-05	1.0E-05	2.33	2.3E-08
Rse(h)	1.0E-05	1.0E-05	13.56	1.5E-08
Rsk1(h)	1.0E-05	1.0E-05	-1.98	1.2E-09
Rsk2(h)	1.0E-05	1.0E-05	6.09	2.3E-09
Rsk3(h)	1.0E-05	1.0E-05	0.77	1.3E-08
Rsk4(h)	1.0E-05	1.0E-05	4.56	4.0E-09
SAPK2a(h)	1.0E-05	1.0E-05	4.9	1.7E-07
SAPK2b(h)	1.0E-05	1.0E-05	-6.41	7.0E-09
SAPK3(h)	1.0E-05	1.0E-05	-2.05	4.2E-08
SAPK4(h)	1.0E-05	1.0E-05	0.28	7.0E-09
SBK1(h)	1.0E-05	1.0E-05	9.66	1.5E-08
SGK(h)	1.0E-05	1.0E-05	-7.07	3.3E-09
SGK2(h)	1.0E-05	1.0E-05	-5.61	1.7E-07
SGK3(h)	1.0E-05	1.0E-05	-2.22	3.4E-08
SIK(h)	1.0E-05	1.0E-05	6.07	6.5E-08
SIK2(h)	1.0E-05	1.0E-05	1.8	2.0E-08
SIK3(h)	1.0E-05	1.0E-05	1.46	6.3E-09

SLK(h)	1.0E-05	1.0E-05	1.74	2.7E-10
Snk(h)	1.0E-05	1.0E-05	-2.57	1.8E-07
SNRK(h)	1.0E-05	1.0E-05	0.06	4.8E-08
SRMS(h)	1.0E-05	1.0E-05	10.23	6.3E-09
SRPK1(h)	1.0E-05	1.0E-05	-6.69	8.5E-10
SRPK2(h)	1.0E-05	1.0E-05	0.9	5.2E-10
STK16(h)	1.0E-05	1.0E-05	-9.98	4.9E-08
STK25(h)	1.0E-05	1.0E-05	19.97	1.0E-08
STK32A(h)	1.0E-05	1.0E-05	0.29	3.2E-08
STK32B(h)	1.0E-05	1.0E-05	10.7	3.0E-09
STK32C(h)	1.0E-05	1.0E-05	7.05	4.7E-08
STK33(h)	1.0E-05	1.0E-05	2.39	1.9E-07
STK39(h)	1.0E-05	1.0E-05	-9.43	1.8E-09
Syk(h)	1.0E-05	1.0E-05	8.25	1.7E-08
TAF1L(h)	1.0E-05	1.0E-05	-1.18	3.7E-08
TAK1(h)	1.0E-05	1.0E-05	-8.54	3.2E-08
TAO1(h)	1.0E-05	1.0E-05	-1.18	4.0E-09
TAO2(h)	1.0E-05	1.0E-05	2.21	4.7E-09
TAO3(h)	1.0E-05	1.0E-05	3.93	6.9E-09
TBK1(h)	1.0E-05	1.0E-05	-1.29	1.9E-08
Tec(h) activated	1.0E-05	1.0E-05	7.04	4.0E-09
TGFBR1(h)	1.0E-05	1.0E-05	-2.34	8.4E-09
TGFBR2(h)	1.0E-05	1.0E-05	-0.85	3.1E-08
Tie2(h)	1.0E-05	1.0E-05	-3.43	1.9E-07
TLK1(h)	1.0E-05	1.0E-05	3.93	1.5E-09
TLK2(h)	1.0E-05	1.0E-05	-2.92	6.8E-09
TNIK(h)	1.0E-05	1.0E-05	8.51	8.7E-09
TRB2(h)	1.0E-05	1.0E-05	-4.47	5.9E-08
TrkA(h)	1.0E-05	1.0E-05	11.1	6.3E-08
TrkB(h)	1.0E-05	1.0E-05	7.83	1.7E-07
TrkC(h)	1.0E-05	1.0E-05	4.31	7.3E-08
TSSK1(h)	1.0E-05	1.0E-05	5.25	8.6E-09

TSSK2(h)	1.0E-05	1.0E-05	16.44	6.1E-09
TSSK3(h)	1.0E-05	1.0E-05	15.47	1.4E-08
TSSK4(h)	1.0E-05	1.0E-05	9.24	1.8E-08
TTBK1(h)	1.0E-05	1.0E-05	-12.31	3.0E-09
TTBK2(h)	1.0E-05	1.0E-05	-1.36	3.1E-10
TTK(h)	1.0E-05	1.0E-05	2.5	4.5E-08
Txk(h)	1.0E-05	1.0E-05	-7.12	1.1E-08
TYK2(h)	1.0E-05	1.0E-05	10.66	1.6E-09
ULK1(h)	1.0E-05	1.0E-05	-0.03	2.3E-09
ULK2(h)	1.0E-05	1.0E-05	7.46	8.7E-09
ULK3(h)	1.0E-05	1.0E-05	-3.51	6.5E-09
VRK1(h)	1.0E-05	1.0E-05	-1.15	1.4E-07
VRK2(h)	1.0E-05	1.0E-05	-0.65	1.4E-07
WEE1(h)	1.0E-05	1.0E-05	-10.87	2.5E-08
Wee1B(h)	1.0E-05	1.0E-05	11.74	7.5E-08
WNK1(h)	1.0E-05	1.0E-05	3.59	2.5E-08
WNK2(h)	1.0E-05	1.0E-05	2.53	1.7E-07
WNK3(h)	1.0E-05	1.0E-05	-4.01	2.5E-07
WNK4(h)	1.0E-05	1.0E-05	9.62	1.7E-07
Yes(h)	1.0E-05	1.0E-05	13.21	8.8E-10
ZAK(h)	1.0E-05	1.0E-05	-5.15	4.2E-09
ZAP-70(h)	1.0E-05	1.0E-05	-15.29	2.9E-08
ZIPK(h)	1.0E-05	1.0E-05	1.95	1.9E-08

Chemistry

General Methods and Materials

Commercially available reagents were used as provided by the supplier without further purification. Solvents for synthesis, extraction, and chromatography were of reagent grade and used as received. Moisture-sensitive reactions were carried out under an atmosphere of argon, and anhydrous solvents were used as provided by the commercial supplier. Reaction progress was monitored by TLC and/or LC/MS with an Agilent MS Quad 6150 instrument and Agilent 1290 HPLC; column: Waters Acquity UPLC HSS T3 1.8 µm 50 x 2.1 mm; eluent A: 1 l Water + 0.25 ml 99% Formic acid , eluent B: 1 l Acetonitrile + 0.25 ml 99% Formic acid; gradient: 0.0 min 90% A → 0.3 min 90% A → 1.7 min 5% A → 3.0 min 5% A Oven: 50°C; flow: 1,20 ml/min; UV-detection: 205 – 305 nm. Crude products were purified using preparative reversed-phase HPLC methodology with UV detection or flash chromatography using Isolera chromatography system with prepacked Biotage silica cartridges. The fractions obtained were concentrated in vacuo to remove organic volatiles. Unless otherwise indicated, all compounds have greater than 95% purity as determined by LC-MS. ^{1}H NMR and ^{13}C NMR spectra were recorded in solvents indicated below at RT with Bruker Avance spectrometers operating at 400, 500 or 600 MHz for ^{1}H NMR; at 126 MHz for ^{13}C NMR. Chemical shifts are reported in ppm relative to tetramethylsilane (TMS) as an internal standard. The descriptions of the coupling patterns of ^{1}H NMR signals are based on the optical appearance of the signals and do not necessarily reflect the physically correct interpretation. In general, the chemical shift information refers to the center of the signal. In the case of multiplets, intervals are given. Spin multiplicities are reported as s = singlet, br s = broad singlet, d = doublet, dd = doublet of doublets, t = triplet, q = quartet, m = multiplet. High-resolution mass spectra (electrospray ionization, ESI) were obtained via UHPLC-MS. Method A: system MS:

Thermo Scientific FT-MS; system UHPLC+: Thermo Scientific UltiMate 3000; Column: Waters, HSST3, 2.1 x 75 mm, C18 1.8 µm; Eluent A: 1 l Water + 0.01% Formic acid; Eluent B: 1 l Acetonitrile + 0.01% Formic acid; gradient: 0.0 min 10% B → 2.5 min 95% B → 3.5 min 95% B; oven: 50°C; flow: 0.90 ml/min; UV-Detection: 210 nm/ Optimum Integration Path 210-300 nm. Method B: system MS: Thermo Scientific FT-MS, system UHPLC+: Thermo Scientific Vanquish; column: Waters, HSST3, 2.1 x 75 mm, C18 1.8 µm; eluent A: 1 l Water + 0.01% Formic acid; eluent B: 1 l Acetonitrile + 0.01% Formic acid; Gradient: 0.0 min 10% B → 2.5 min 95% B → 3.5 min 95% B; oven: 50°C; flow: 0.90 ml/min; UV-Detection: 210 nm. Preparative HPLC was carried out with a Waters Prep LC/MS System; column: Phenomenex Kinetex C18 5µm 100x30 mm; eluent A: water, eluent B: Acetonitrile, eluent C: 2% Formic acid in Water, eluent D: Acetonitrile/Water (80Vol.%/20Vol%); flow: 80 ml/min , room temperature, UV detection: 200-400 nm, At-Column Injection (total injection); gradient: eluent A: 0 → 2 min 55 ml, eluent B: 0 → 2min 15 ml, eluent A: 2 → 10 min with 55 ml → 31 ml and Eluent B: 15 ml → 39 ml, 10 → 12 min 0 ml Eluent A and 70 ml Eluent B. Eluent C and Eluent D with constant flow of 5 ml/min each. Optical rotations were recorded on an Anton Polarimeter MCP200 with parameters (solvent, wavelength, temperature) as indicated.

Experimental Procedures

The experimental procedures for BAY-805 (**21**) can be found in the Experimental Section of the main manuscript.

Synthesis of BAY-728

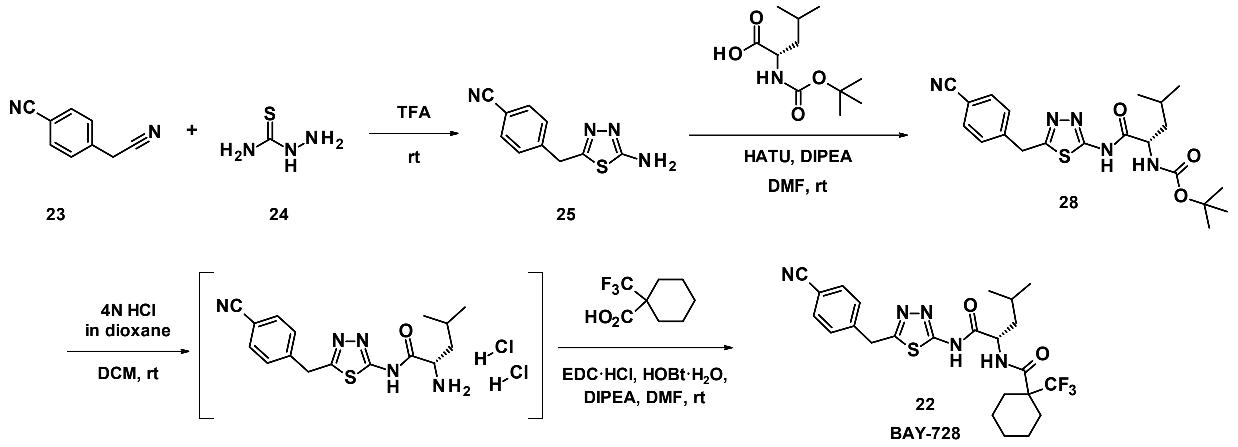

tert-butyl N-[(1S)-1-[[5-[(4-cyanophenyl)methyl]-1,3,4-thiadiazol-2-yl]carbamoyl]-3-methyl-butyl]carbamate (**28**):

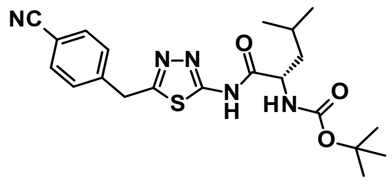

Boc-L-Leucine (802 mg, 3.47 mmol, 1.5 eq.) and HATU (1.32 g, 3.47 mmol, 1.5 eq.) were dissolved in DMF (6.0 mL) and DIPEA (896 mg, 1.21 mL, 6.94 mmol, 3.0 eq.) was added. After stirring for 30 min at room temperature, 4-[(5-amino-1,3,4-thiadiazol-2-yl)methyl]benzonitrile (compound **25**, 500 mg, 2.31 mmol, 1.0 eq.) was added and the reaction mixture was stirred at room temperature overnight. The reaction mixture was diluted with water and extracted with

EtOAc (3x). The organic phases were dried over Na_2SO_4, filtered and concentrated. The crude product was purified by column chromatography (silica gel, eluent: cyclohexane / ethyl acetate 9:1 to 0:1) to yield the desired compound **28** (890 mg, 93% purity, 83% yield). **LC-MS** (Method B): R_t = 1.99 min; **HRMS:** m/z $[M+H]^+$ calcd for $C_{21}H_{28}N_5O_3S$: 430.1912, found 430.1908. **^{1}H NMR** (600 MHz, DMSO-d_6) δ ppm 0.87 (t, *J*=6.14 Hz, 6 H), 1.36 (s, 9H), 1.38 - 1.42 (m, 1 H), 1.48 - 1.56 (m, 1 H), 1.58 - 1.68 (m, 1 H), 4.19 - 4.30 (m, 1 H), 4.48 (s, 2 H), 7.18 - 7.27 (m, 1 H), 7.55 (d, *J*=8.25 Hz, 2 H), 7.81 (s, 2 H), 12.63 (s, 1 H).

N-[(1S)-1-[[5-[(4-cyanophenyl)methyl]-1,3,4-thiadiazol-2-yl]carbamoyl]-3-methyl-butyl]-1-(trifluoromethyl)cyclohexanecarboxamide (**22**):

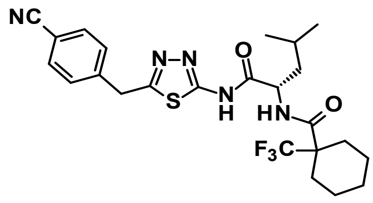

Step 1:

Tert-butyl N-[(1S)-1-[[5-[(4-cyanophenyl)methyl]-1,3,4-thiadiazol-2-yl]carbamoyl]-3-methyl-butyl]carbamate (compound **28**, 93% purity, 890 mg, 1.92 mmol, 1.0 eq.) was stirred in 4 N HCl in dioxane (4.80 mL, 19.2 mmol, 10.0 eq.) for 6 h at room temperature. The reaction mixture was concentrated and dried *in vacuo* to yield (2S)-2-amino-N-[5-[(4-cyanophenyl)methyl]-1,3,4-thiadiazol-2-yl]-4-methyl-pentanamide hydrochloride (710 mg, 100 % purity, quant). **LC-MS** (Method A): R_t = 0.85 min; **HRMS:** m/z $[M+H-HCl]^+$ calcd for $C_{16}H_{20}N_5OS$: 330.1388, found 330.1383.

Step 2:

1-(Trifluoromethyl)cyclohexanecarboxylic acid (519 mg, 2.65 mmol, 1.5 eq.), EDC·HCl (507 mg, 2.65 mmol, 1.5 eq.) and HOBt Hydrate (405 mg, 2.65 mmol, 1.5 eq.) were dissolved in DMF (8.0

mL). DIPEA (1.14 g, 1.54 mL, 8.82 mmol, 5.0 eq.) and (2S)-2-amino-N-[5-[(4-cyanophenyl) methyl]-1,3,4-thiadiazol-2-yl]-4-methyl-pentanamide dihydrochloride (710 mg, 1.76 mmol, 1.0 eq.) were added and the reaction mixture was stirred at room temperature over night. The crude mixture was purified using preparative HPLC (water/acetonitrile gradient) to yield the desired compound **22** (648 mg, 100 % purity, 72 % yield). **LC-MS** (Method B): R_t = 2.20 min; **HRMS:** m/z $[M+H]^+$ calcd for $C_{24}H_{29}F_3N_5O_2S$: 508.1994, found 508.1989. **^{1}H NMR** (600 MHz, DMSO-d_6) δ ppm 0.84 (d, *J*=6.65 Hz, 3 H), 0.89 (d, *J*=6.65 Hz, 3 H), 1.09 - 1.20 (m, 2 H), 1.29 - 1.48 (m, 4 H), 1.52 - 1.70 (m, 4 H), 1.74 - 1.83 (m, 1 H), 2.32 - 2.37 (m, 1 H), 2.44 - 2.48 (m, 1 H), 4.47 (s, 2 H), 4.61 - 4.69 (m, 1 H), 7.55 (d, *J*=8.22 Hz, 2 H), 7.81 (d, *J*=8.22 Hz, 2 H), 8.13 - 8.23 (m, 1 H), 12.71 (br s, 1 H). **^{13}C NMR** (126 MHz, DMSO-d_6) δ ppm 20.9, 22.2, 22.2, 23.6, 24.8, 24.9, 27.0, 27.3, 35.2, 52.0, 52.2, 52.6, 110.4, 119.2, 125.9, 128.1, 130.5, 133.1, 143.9, 159.4, 162.6, 167.0, 172.0. **$[\alpha]_D^{20}$** = -52.13 (c = 0.188 in MeOH)

Synthesis of Screening Hit 1

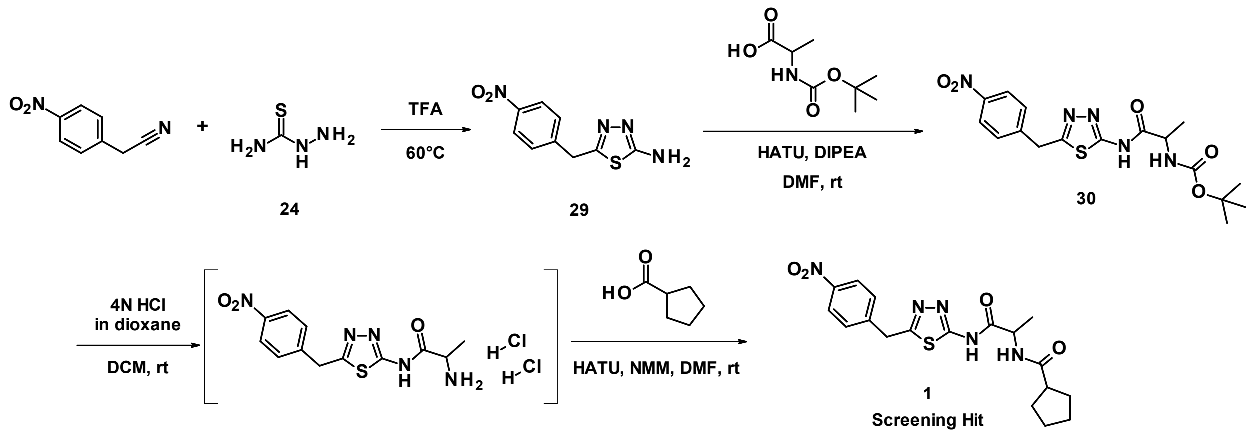

5-[(4-nitrophenyl)methyl]-1,3,4-thiadiazol-2-amine (**29**):

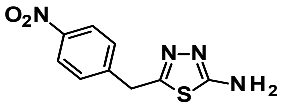

2-(4-nitrophenyl)acetonitrile (9.73 g, 60.0 mmol, 1.0 eq.) and thiosemcarbazide (compound **24**, 8.20 g, 90.0 mmol, 1.5 eq.) were dissolved in TFA (30 mL) and stirred at 60°C overnight. The reaction mixture was poured on ice water (300 mL) and basified with aq. NH_4OH. The filtered precipitate was washed with water (200 mL), EtOH (100 mL) and Et_2O (100 mL) and was dried in vacuo to yield the desired compound **29** (13.6 g, 100% purity, 96 % yield). **LC-MS** (Method A): R_t = 0.99 min; **HRMS:** m/z [M+H]+ calcd for $C_9H_9N_4O_2S$: 237.0446, found 237.0441. **^{1}H NMR** (600 MHz, DMSO-d_6) δ ppm 4.34 (s, 2 H), 7.11 (s, 2 H), 7.57 (d, *J*=8.62 Hz, 2 H), 8.20 (d, *J*=8.62 Hz, 2 H).

tert-butyl N-[1-methyl-2-[[5-[(4-nitrophenyl)methyl]-1,3,4-thiadiazol-2-yl]amino]-2-oxo-ethyl] carbamate (**30**):

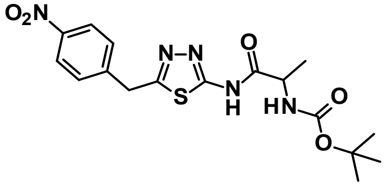

5-[(4-nitrophenyl)methyl]-1,3,4-thiadiazol-2-amine (compound **29**, 150 mg, 635 µmol, 1.0 eq.), N-(tert-butoxycarbonyl)alanine (144 mg, 762 µmol, 1.2 eq.), HATU (290 mg, 762 µmol, 1.2 eq.) and DIPEA (164 mg, 221 µL, 1.27 mmol, 2.0 eq.) were dissolved in DMF (1.5 mL) and stirred at room temperature over night. A few drops of water were added and the reaction mixture was purified using preparative HPLC (water/acetonitrile gradient) to yield the desired compound **30**

(199 mg, 98 % purity, 75 % yield). **LC-MS** (Method B): R_t = 1.72 min; **HRMS:** m/z $[M+H]^+$ calcd for $C_{17}H_{22}N_5O_5S$: 408.1341, found 4081336. **^{1}H NMR** (600 MHz, DMSO-d_6) δ ppm 1.23 - 1.26 (m, 3 H), 1.36 (s, 9 H), 4.16 - 4.26 (m, 1 H), 4.54 (s, 2 H), 7.23 - 7.32 (m, 1 H), 7.62 (d, *J*=8.61 Hz, 2 H), 8.21 (d, *J*=8.61 Hz, 2 H), 12.55 (br s, 1 H).

N-[1-methyl-2-[[5-[(4-nitrophenyl)methyl]-1,3,4-thiadiazol-2-yl]amino]-2-oxo-ethyl]cyclopentanecarboxamide (**1**):

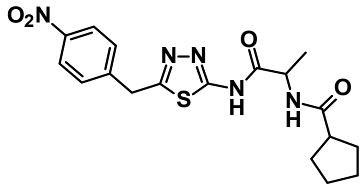

Step 1:

tert-butyl N-[1-methyl-2-[[5-[(4-nitrophenyl)methyl]-1,3,4-thiadiazol-2-yl]amino]-2-oxo-ethyl] carbamate (compound **30**, 98% purity, 196 mg, 471 µmol, 1.0 eq.) was dissolved in DCM (3.9 mL) and 4 N HCl in dioxane (1.18 mL, 4.71 mmol, 10.0 eq.) was added. After stirring for 3 h at room temperature, the reaction mixture was concentrated. The residue was repeatedly suspended in DCM, concentrated under reduced pressure and finally dried *in vacuo* to yield 2-amino-N-[5-[(4-nitrophenyl)methyl]-1,3,4-thiadiazol-2-yl]propanamide dihydrochloride (179 mg, 100 % purity, 100 % yield). **LC-MS** (Method B): R_t = 0.73 min; **HRMS:** m/z $[M+H-2HCl]^+$ calcd for $C_{12}H_{14}N_5O_3S$: 308.0817, found 308.0812.

Step 2:

2-amino-N-[5-[(4-nitrophenyl)methyl]-1,3,4-thiadiazol-2-yl]propanamide dihydrochloride (176 mg, 463 µmol, 1.0 eq.), cyclopentanecarboxylic acid (79.2 mg, 694 µmol, 1.5 eq.) and HATU (264 mg, 694 µmol, 1.5 eq.) were dissolved in DMF (1.0 mL) and 4-methylmorpholine (234 mg, 254

µL, 2.31 mmol, 5.0 eq.) was added. The reaction mixture was stirred at room temperature over night. A few drops of water were added and the reaction mixture was purified using preparative HPLC (water/acetonitrile gradient) to yield the desired compound **1** (148 mg, 100 % purity, 79 % yield). **LC-MS** (Method A): R_t = 1.57 min; **HRMS:** m/z $[M+H]^+$ calcd for $C_{18}H_{22}N_5O_4S$: 404.1392, found 404.1387. **^{1}H NMR** (500 MHz, DMSO-d_6) δ ppm 1.28 (d, *J*=7.17 Hz, 3 H), 1.43 - 1.51 (m, 2 H), 1.53 - 1.62 (m, 4 H), 1.68 - 1.77 (m, 2 H), 2.59 - 2.67 (m, 1 H), 4.43 (m, 1 H), 4.54 (s, 2 H), 7.59 - 7.65 (m, 2 H), 8.17 (d, *J*=6.41 Hz, 1 H), 8.19 - 8.23 (m, 2 H), 12.59 (br s, 1 H). **^{13}C NMR** (126 MHz, DMSO-d_6) δ ppm 17.73, 26.14, 26.22, 30.30, 30.41, 34.87, 44.00, 49.01, 124.32, 130.68, 146.08, 147.02, 159.43, 162.37, 172.63, 176.08.

Synthesis of compound 2

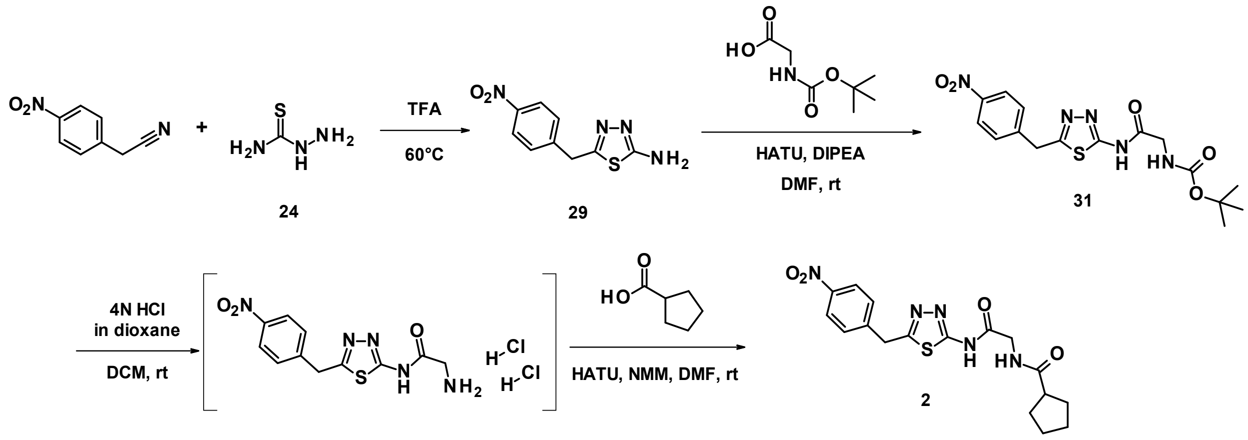

tert-butyl N-[2-[[5-[(4-nitrophenyl)methyl]-1,3,4-thiadiazol-2-yl]amino]-2-oxo-ethyl]carbamate **(31)**:

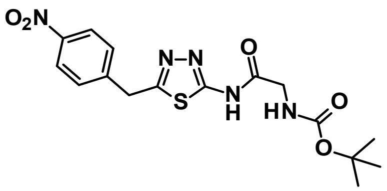

5-[(4-nitrophenyl)methyl]-1,3,4-thiadiazol-2-amine (compound **29**, 150 mg, 635 µmol, 1.0 eq.), N-(tert-butoxycarbonyl)glycine (144 mg, 762 µmol, 1.2 eq.), HATU (290 mg, 762 µmol, 1.2 eq.) and DIPEA (164 mg, 221 µL, 1.27 mmol, 2.0 eq.) were dissolved in DMF (1.5 mL) and stirred at room temperature over night. A few drops of water were added and the reaction mixture was purified using preparative HPLC (water/acetonitrile gradient) to yield the desired compound **31** (122 mg, 100 % purity, 49 % yield). **LC-MS** (Method A): R_t = 1.56 min; **HRMS:** m/z $[M+H]^+$ calcd for $C_{16}H_{20}N_5O_5S$: 394.1185, found 394.1180. **^{1}H NMR** (600 MHz, DMSO-*d*$_6$) δ ppm 1.37 (s, 9 H), 3.84 (d, *J*=6.06 Hz, 2 H), 4.54 (s, 2 H), 7.18 (t, *J*=5.97 Hz, 1 H), 7.62 (d, *J*=8.61 Hz, 2 H), 8.21 (d, *J*=8.80 Hz, 2 H), 12.52 (br s, 1 H).

N-[2-[[5-[(4-nitrophenyl)methyl]-1,3,4-thiadiazol-2-yl]amino]-2-oxo-ethyl]cyclopentane-carboxamide (**2**):

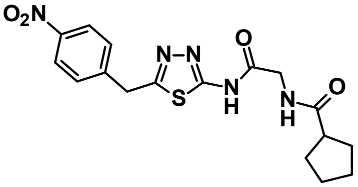

Step 1:

tert-butyl N-[2-[[5-[(4-nitrophenyl)methyl]-1,3,4-thiadiazol-2-yl]amino]-2-oxo-ethyl]carbamate (compound **31**, 100% purity, 120 mg, 305 µmol, 1.0 eq.) was dissolved in DCM (2.5 mL) and 4 N HCl in dioxane (763 µL, 3.05 mmol, 10.0 eq.) was added. After stirring for 5 h at room temperature, the reaction mixture was concentrated. The residue was repeatedly suspended in DCM, concentrated under reduced pressure and finally dried *in vacuo* to yield 2-amino-N-[5-[(4-nitrophenyl)methyl]-1,3,4-thiadiazol-2-yl]acetamide dihydrochloride (109 mg, 94 % purity, 92 %

yield). **LC-MS** (Method B): R_t = 0.66 min; **HRMS:** m/z $[M+H-2HCl]^+$ calcd for $C_{11}H_{12}N_5O_3S$: 294.0660, found 294.0656.

Step 2:

2-amino-N-[5-[(4-nitrophenyl)methyl]-1,3,4-thiadiazol-2-yl]acetamide dihydrochloride (107 mg, 94 % purity, 275 µmol, 1.0 eq.), cyclopentanecarboxylic acid (47.0 mg, 412 µmol, 1.5 eq.) and HATU (157 mg, 412 µmol, 1.5 eq.) were dissolved in DMF (1.0 mL) and 4-methylmorpholine (139 mg, 151 µL, 1.37 mmol, 5.0 eq.) was added. The reaction mixture was stirred at room temperature overnight. A few drops of water were added and the reaction mixture was purified using preparative HPLC (water/acetonitrile gradient) to yield the desired compound **2** (63.3 mg, 100 % purity, 57 % yield). **LC-MS** (Method A): R_t = 1.46 min; **HRMS:** m/z $[M+H]^+$ calcd for $C_{17}H_{20}N_5O_4S$: 390.1236, found 390.1231. **^{1}H NMR** (500 MHz, DMSO-d_6) δ ppm 1.43 - 1.53 (m, 2 H), 1.56 - 1.65 (m, 4 H), 1.70 - 1.78 (m, 2 H), 2.59 - 2.68 (m, 1 H), 3.97 (d, *J*=5.95 Hz, 2 H), 4.54 (s, 2 H), 7.62 (d, *J*=8.70 Hz, 2 H), 8.16 - 8.24 (m, 3 H), 12.56 (br s, 1 H). **^{13}C NMR** (126 MHz, DMSO-d_6) δ ppm 25.5, 29.8, 34.3, 41.9, 43.7, 123.7, 130.1, 145.5, 146.5, 158.7, 161.7, 168.6, 175.9.

Synthesis of compound 3

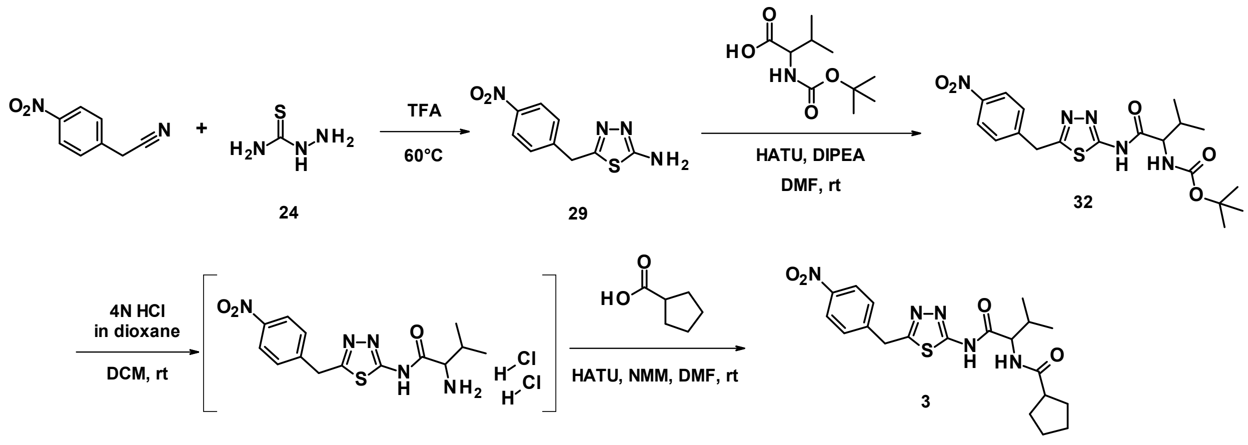

tert-butyl N-[2-methyl-1-[[5-[(4-nitrophenyl)methyl]-1,3,4-thiadiazol-2-yl]carbamoyl] propyl]carbamate (**32**):

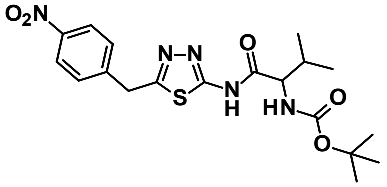

5-[(4-nitrophenyl)methyl]-1,3,4-thiadiazol-2-amine (compound **29**, 150 mg, 635 µmol, 1.0 eq.), N-(tert-butoxycarbonyl)valine (166 mg, 762 µmol, 1.2 eq.), HATU (290 mg, 762 µmol, 1.2 eq.) and DIPEA (164 mg, 221 µL, 1.27 mmol, 2.0 eq.) were dissolved in DMF (1.5 mL) and stirred at room temperature over night. A few drops of water were added and the reaction mixture was purified using preparative HPLC (water/acetonitrile gradient) to yield the desired compound **32** (215 mg, 91 % purity, 71 % yield). **LC-MS** (Method B): R_t = 1.93 min; **HRMS:** m/z $[M+H]^+$ calcd for $C_{19}H_{26}N_5O_5S$: 436.1654, found 436.1649. **^{1}H NMR** (600 MHz, DMSO-d_6) δ ppm 0.83 - 0.89 (m, 6 H), 1.36 (s, 9 H), 1.90 - 2.06 (m, 1 H), 4.05 (br t, *J*=7.43 Hz, 1 H), 4.54 (s, 2 H), 7.13 (br d, *J*=7.83 Hz, 1 H), 7.63 (d, *J*=8.61 Hz, 2 H), 8.18 - 8.24 (m, 2 H), 12.58 (s, 1 H).

N-[2-methyl-1-[[5-[(4-nitrophenyl)methyl]-1,3,4-thiadiazol-2-yl]carbamoyl]propyl]cyclo-pentanecarboxamide (**3**):

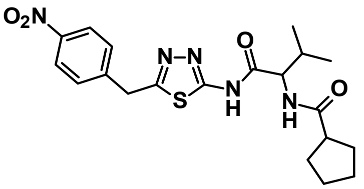

Step 1:

tert-butyl N-[2-methyl-1-[[5-[(4-nitrophenyl)methyl]-1,3,4-thiadiazol-2-yl]carbamoyl]propyl] carbamate (compound **32**, 91% purity, 213 mg, 445 µmol, 1.0 eq.) was dissolved in DCM (3.7 mL) and 4 N HCl in dioxane (1.11 mL, 4.45 mmol, 10.0 eq.) was added. After stirring for 5 h at room temperature, the reaction mixture was concentrated. The residue was repeatedly suspended in DCM, concentrated under reduced pressure and finally dried *in vacuo* to yield 2-amino-3-methyl-N-[5-[(4-nitrophenyl)methyl]-1,3,4-thiadiazol-2-yl]butanamide dihydrochloride (200 mg, 90 % purity, 99 % yield). **LC-MS** (Method B): R_t = 0.89 min; **HRMS:** m/z $[M+H-2HCl]^+$ calcd for $C_{14}H_{18}N_5O_3S$: 336.1130, found 336.1125.

Step 2:

2-amino-3-methyl-N-[5-[(4-nitrophenyl)methyl]-1,3,4-thiadiazol-2-yl]butanamide dihydrochloride (197 mg, 90% purity, 434 µmol, 1.0 eq.), cyclopentanecarboxylic acid (74.3 mg, 651 µmol, 1.5 eq.) and HATU (248 mg, 651 µmol, 1.5 eq.) were dissolved in DMF (1.0 mL) and 4-methylmorpholine (220 mg, 239 µL, 2.17 mmol, 5.0 eq.) was added. The reaction mixture was stirred at room temperature over night. A few drops of water were added and the reaction mixture was purified using preparative HPLC (water/acetonitrile gradient) to yield the desired compound

3 (126,8 mg, 100 % purity, 68 % yield). **LC-MS** (Method A): $R_t = 1.78$ min; **HRMS:** m/z $[M+H]^+$ calcd for $C_{20}H_{26}N_5O_4S$: 432.1705, found 432.1700. **^{1}H NMR** (400 MHz, DMSO-d_6) δ ppm 0.87 (m, 6 H), 1.40 - 1.52 (m, 2 H), 1.53 - 1.62 (m, 4 H), 1.65 - 1.81 (m, 2 H), 1.96 - 2.09 (m, 1 H), 2.66 - 2.79 (m, 1 H), 4.36 (t, *J*=7.56 Hz, 1 H), 4.54 (s, 2 H), 7.62 (d, *J*=8.81 Hz, 2 H), 8.01 (d, *J*=7.76 Hz, 1 H), 8.19 - 8.24 (m, 2 H), 12.64 (s, 1 H). **^{13}C NMR** (126 MHz, DMSO-d_6) δ ppm 18.32, 18.93, 25.56, 25.67, 29.51, 29.98, 30.31, 34.30, 43.46, 58.07, 123.75, 130.15, 145.47, 146.46, 158.52, 161.84, 170.98, 175.82.

Synthesis of compound 4

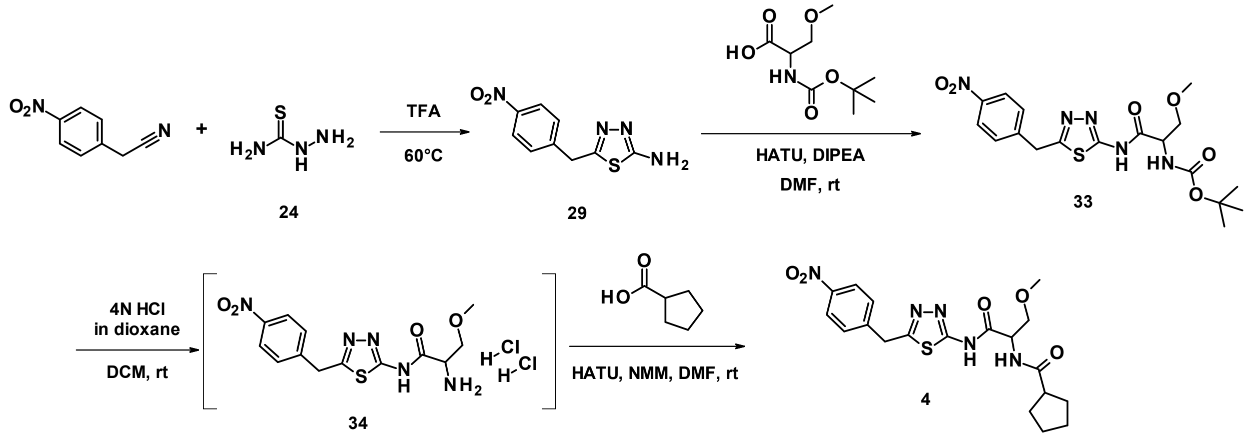

<u>*tert-butyl N-[1-(methoxymethyl)-2-[[5-[(4-nitrophenyl)methyl]-1,3,4-thiadiazol-2-yl]amino]-2-oxo-ethyl]carbamate* (**33**):</u>

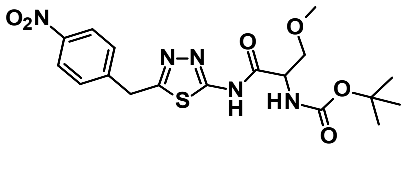

N-(tert-butoxycarbonyl)-O-methylserine (8.91 g, 40.6 mmol, 1.2 eq.) and HATU (15.5 g, 40.6 mmol, 1.2 eq.) were dissolved in DMF (76 mL) and DIPEA (8.75 g, 11.8 mL, 67.7 mmol, 2.0 eq.) was added. After stirring at room temperature for 40 min, 5-[(4-nitrophenyl) methyl]-1,3,4-

thiadiazol-2-amine (compound **29**, 8.00 g, 33.9 mmol, 1.0 eq.) was added and the reaction mixture was stirred for 3 d at room temperature. The reaction mixture was diluted with water and extracted with EtOAc. The organic phases were dried over Na_2SO_4, filtered and concentrated. The crude product was purified by column chromatography (silica gel, eluent: cyclohexane / ethyl acetate 7:1 to 1:9) to yield the desired compound **33** (13.4 g, 100% purity, 91% yield). **LC-MS** (Method A): R_t = 1.73 min; **HRMS:** m/z [M+H]+ calcd for $C_{18}H_{24}N_5O_6S$: 438.1447, found 438.1442. ^{1}**H NMR** (600 MHz, DMSO-d_6) δ ppm 1.37 (s, 9 H), 3.23 (s, 3 H), 3.47 - 3.62 (m, 2 H), 4.39 - 4.50 (m, 1 H), 4.55 (s, 2 H), 7.21 (br d, *J*=7.15 Hz, 1 H), 7.62 (d, *J*=8.80 Hz, 2 H), 8.21 (d, *J*=8.62 Hz, 2 H), 12.68 (s, 1 H).

2-amino-3-methoxy-N-[5-[(4-nitrophenyl)methyl]-1,3,4-thiadiazol-2-yl]propanamide hydrochloride (**34**):

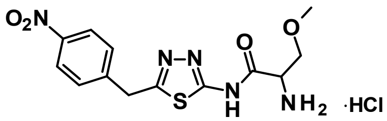

tert-butyl N-[1-(methoxymethyl)-2-[[5-[(4-nitrophenyl)methyl]-1,3,4-thiadiazol-2-yl]amino]-2-oxo-ethyl]carbamate (compound **33**, 13.4 g, 100 % purity, 30.7 mmol, 1.0 eq.) was added to 4N HCl in dioxane (76.8 mL, 307 mmol, 10.0 eq.) at 0 °C. After stirring at room temperature for 1h, all volatiles were removed *in vacuo*. The residue was repeatedly suspended in DCM, concentrated under reduced pressure, and finally dried *in vacuo* to give the desire compound **34** (13.1 g, 75% purity, 85% yield) with 25 % residual dioxane as impurity. **LC-MS** (Method A): R_t = 0.87 min; **HRMS:** m/z [M-Cl]+ calcd for $C_{13}H_{16}N_5O_4S$: 338.0923, found 338.0918. ^{1}**H NMR** (600 MHz,

DMSO-d_6) δ ppm 3.28 (s, 3 H), 3.80 (m, 1 H), 3.88 (m, 1 H), 4.29 - 4.41 (m, 1 H), 4.59 (s, 2 H), 7.63 (d, *J*=8.62 Hz, 2 H), 8.22 (d, *J*=8.62 Hz, 2 H), 8.70 (br s, 3 H).

N-[1-(methoxymethyl)-2-[[5-[(4-nitrophenyl)methyl]-1,3,4-thiadiazol-2-yl]amino]-2-oxo-ethyl]cyclopentanecarboxamide (**4**):

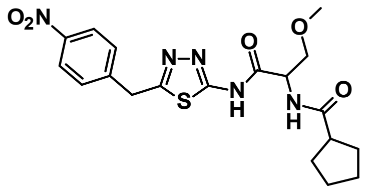

Cyclopentanecarboxylic acid (17.1 mg, 150 µmol, 1.5 eq.) and HATU (57.0 mg, 150 µmol, 1.5 eq.) were dissolved in DMF (1.1 mL) and 4-methylmorpholine (50.6 mg, 55 µL, 500 µmol, 5.0 eq.) was added. After stirring for 15 min at room temperature, 2-amino-3-methoxy-N-[5-[(4-nitrophenyl)methyl]-1,3,4-thiadiazol-2-yl]propanamide hydrochloride (compound **34**, 49.8 mg, 75 % purity, 100 µmol, 1.0 eq.) was added and the reaction mixture was stirred at room temperature overnight. A few drops of water were added, and the reaction mixture was purified using preparative HPLC (water/acetonitrile gradient) to yield the desired compound **4** (36.8 mg, 100% purity, 85 % yield). **LC-MS** (Method A): R_t = 1.60 min; **HRMS:** m/z [M+H]+ calcd for $C_{19}H_{24}N_5O_5S$: 434.1498, found 434.1493. **^{1}H NMR** (500 MHz, DMSO-d_6) δ ppm 1.43 - 1.52 (m, 2 H), 1.53 - 1.64 (m, 4 H), 1.68 - 1.78 (m, 2 H), 2.63 - 2.75 (m, 1 H), 3.25 (s, 3 H), 3.53 - 3.58 (m, 1 H), 3.58 - 3.63 (m, 1 H), 4.55 (s, 2 H), 4.67 - 4.74 (m, 1 H), 7.59 - 7.64 (m, 1 H), 8.17 - 8.23 (m, 3 H), 12.70 (s, 1 H). **^{13}C NMR** (126 MHz, DMSO-d_6) δ ppm 25.7, 29.8, 29.9, 34.3, 43.4, 52.7, 58.3, 123.7, 130.1, 145.5, 146.5, 161.9, 169.4, 175.8.

Synthesis of compounds 5 – 9

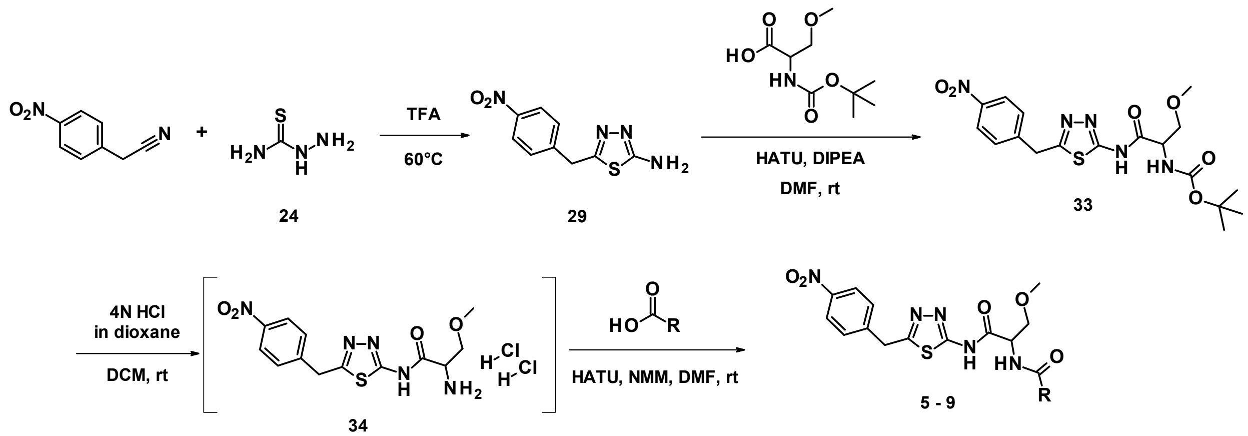

N-[1-(methoxymethyl)-2-[[5-[(4-nitrophenyl)methyl]-1,3,4-thiadiazol-2-yl]amino]-2-oxo-ethyl]benzamide (**5**):

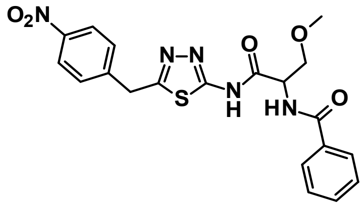

Benzoic acid (18.3 mg, 150 µmol, 1.5 eq.) and HATU (57.0 mg, 150 µmol, 1.5 eq.) were dissolved in DMF (0.8 mL) and 4-methylmorpholine (50.6 mg, 55 µL, 500 µmol, 5.0 eq.) was added. After stirring for 15 min at room temperature, 2-amino-3-methoxy-N-[5-[(4-nitrophenyl)methyl]-1,3,4-thiadiazol-2-yl]propanamide hydrochloride (compound **34**, 49.8 mg, 75 % purity, 100 µmol, 1.0 eq.) was added and the reaction mixture was stirred at room temperature overnight. A few drops of water were added, and the reaction mixture was purified using preparative HPLC (water/acetonitrile gradient) to yield the desired compound **5** (28.9 mg, 100% purity, 65 % yield).

LC-MS (Method B): $R_t = 1.65$ min; **HRMS:** m/z $[M+H]^+$ calcd for $C_{20}H_{20}N_5O_5S$: 442.1185, found 442.1180. **^{1}H NMR** (500 MHz, DMSO-d_6) δ ppm 3.30 (s, 3 H), 3.74 (m, 2 H), 4.55 (s, 2 H), 4.88 - 4.95 (m, 1 H), 7.46 - 7.50 (m, 2 H), 7.54 - 7.58 (m, 1 H), 7.60 - 7.64 (m, 2 H), 7.87 - 7.93 (m, 2 H), 8.18 - 8.23 (m, 2 H), 8.75 (d, *J*=6.71 Hz, 1 H), 12.83 (s, 1 H). **^{13}C NMR** (126 MHz, DMSO-

d_6) δ ppm 34.3, 53.6, 58.3, 70.8, 123.7, 127.6, 128.2, 130.1, 131.5, 133.3, 145.5, 146.5, 158.7, 161.9, 166.6, 169.4.

N-[1-(methoxymethyl)-2-[[5-[(4-nitrophenyl)methyl]-1,3,4-thiadiazol-2-yl]amino]-2-oxo-ethyl]-2-methyl-propanamide (**6**):

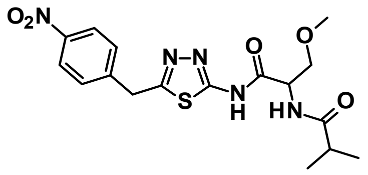

2-Methylpropanoic acid (13.2 mg, 150 µmol, 1.5 eq.) and HATU (57.0 mg, 150 µmol, 1.5 eq.) were dissolved in DMF (0.8 mL) and 4-methylmorpholine (50.6 mg, 55 µL, 500 µmol, 5.0 eq.) was added. After stirring for 15 min at room temperature, 2-amino-3-methoxy-N-[5-[(4-nitrophenyl)methyl]-1,3,4-thiadiazol-2-yl]propanamide hydrochloride (compound **34**, 49.8 mg, 75 % purity, 100 µmol, 1.0 eq.) was added and the reaction mixture was stirred at room temperature overnight. A few drops of water were added, and the reaction mixture was purified using preparative HPLC (water/acetonitrile gradient) to yield the desired compound **6** (35.8 mg, 100% purity, 88 % yield). **LC-MS** (Method A): R_t = 1.42 min; **HRMS:** m/z [M+H]+ calcd for $C_{17}H_{22}N_5O_5S$: 408.1341, found 408.1336. **^{1}H NMR** (600 MHz, DMSO-d_6) δ ppm 0.94 - 1.01 (m, 6 H), 2.51 - 2.53 (m, 1 H), 3.25 (s, 3 H), 3.53 - 3.57 (m, 1 H), 3.58 - 3.63 (m, 1 H), 4.54 (s, 2 H), 4.65 - 4.74 (m, 1 H), 7.60 - 7.64 (m, 2 H), 8.15 - 8.18 (m, 1 H), 8.19 - 8.23 (m, 2 H), 12.69 (s, 1 H). **^{13}C NMR** (126 MHz, DMSO-d_6) δ ppm 19.3, 19.4, 33.2, 34.3, 52.6, 58.3, 71.3, 123.7, 130.1, 145.5, 146.5, 161.9, 158.7, 169.3, 176.5.

N-[1-(methoxymethyl)-2-[[5-[(4-nitrophenyl)methyl]-1,3,4-thiadiazol-2-yl]amino]-2-oxo-ethyl]cyclohexanecarboxamide (**7**):

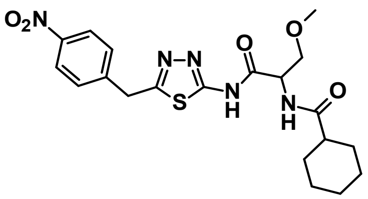

Cyclohexanecarboxylic acid (19.2 mg, 150 µmol, 1.5 eq.) and HATU (57.0 mg, 150 µmol, 1.5 eq.) were dissolved in DMF (0.8 mL) and 4-methylmorpholine (50.6 mg, 55 µL, 500 µmol, 5.0 eq.) was added. After stirring for 15 min at room temperature, 2-amino-3-methoxy-N-[5-[(4-nitrophenyl)methyl]-1,3,4-thiadiazol-2-yl]propanamide hydrochloride (compound **34**, 49.8 mg, 75 % purity, 100 µmol, 1.0 eq.) was added and the reaction mixture was stirred at room temperature overnight. A few drops of water were added, and the reaction mixture was purified using preparative HPLC (water/acetonitrile gradient) to yield the desired compound **7** (41.8 mg, 100% purity, 93 % yield). **LC-MS** (Method A): R_t = 1.42 min; **HRMS:** m/z [M+H]+ calcd for $C_{20}H_{26}N_5O_5S$: 448.1654, found 448.1649. **^{1}H NMR** (500 MHz, DMSO-d_6) δ ppm 1.08 - 1.35 (m, 5 H), 1.56 - 1.63 (m, 1 H), 1.63 - 1.73 (m, 4 H), 2.24 (m, 1 H), 3.24 (s, 3 H), 3.51 - 3.56 (m, 1 H), 3.57 - 3.62 (m, 1 H), 4.55 (s, 2 H), 4.62 - 4.76 (m, 1 H), 7.62 (d, *J*=8.85 Hz, 2 H), 8.10 (d, *J*=7.17 Hz, 1 H), 8.21 (d, *J*=8.85 Hz, 2 H), 12.68 (s, 1 H). **^{13}C NMR** (126 MHz, DMSO-d_6) δ ppm 25.1, 25.1, 25.3, 28.9, 29.1, 34.3, 43.2, 52.5, 58.3, 71.2, 123.7, 130.1, 145.5, 169.4, 175.6.

N-[1-(methoxymethyl)-2-[[5-[(4-nitrophenyl)methyl]-1,3,4-thiadiazol-2-yl]amino]-2-oxo-ethyl]tetrahydropyran-4-carboxamide (**8**):

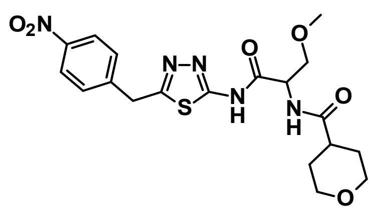

Tetrahydro-2H-pyran-4-carboxylic acid (19.5 mg, 150 µmol, 1.5 eq.) and HATU (57.0 mg, 150 µmol, 1.5 eq.) were dissolved in DMF (0.8 mL) and 4-methylmorpholine (50.6 mg, 55 µL, 500 µmol, 5.0 eq.) was added. After stirring for 15 min at room temperature, 2-amino-3-methoxy-N-[5-[(4-nitrophenyl)methyl]-1,3,4-thiadiazol-2-yl]propanamide hydrochloride (compound **34**, 49.8 mg, 75 % purity, 100 µmol, 1.0 eq.) was added and the reaction mixture was stirred at room temperature overnight. A few drops of water were added, and the reaction mixture was purified using preparative HPLC (water/acetonitrile gradient) to yield the desired compound **8** (39.2 mg, 100% purity, 87 % yield). **LC-MS** (Method A): R_t = 1.42 min; **HRMS:** m/z [M+H]+ calcd for $C_{19}H_{24}N_5O_6S$: 450.1447, found450.1442. **^{1}H NMR** (600 MHz, DMSO-d_6) δ ppm 1.46 - 1.62 (m, 4 H), 3.24 (s, 3 H), 3.28 (m, 3 H), 3.53 - 3.56 (m, 1 H), 3.58 - 3.63 (m, 1 H), 3.81 - 3.87 (m, 2 H), 4.54 (s, 2 H), 4.69 (m, 1 H), 7.62 (d, *J*=8.80 Hz, 2 H), 8.18 - 8.24 (m, 3 H), 12.70 (s, 1 H). **^{13}C NMR** (126 MHz, DMSO-d_6) δ ppm 28.7, 28.8, 34.3, 52.6, 58.3, 66.3, 71.2, 123.7, 130.1, 145.5, 146.5, 158.7, 161.9, 169.2, 174.4.

N-[1-(methoxymethyl)-2-[[5-[(4-nitrophenyl)methyl]-1,3,4-thiadiazol-2-yl]amino]-2-oxo-ethyl]-1-methyl-cyclohexanecarboxamide (**9**):

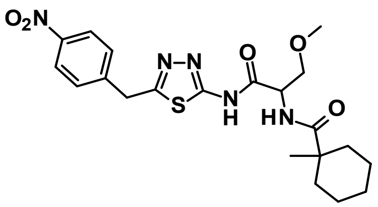

1-Methylcyclohexanecarboxylic acid (28.4 mg, 200 µmol, 2.0 eq.), HATU (53.2 mg, 140 µmol, 1.4 eq.) and 2-amino-3-methoxy-N-[5-[(4-nitrophenyl)methyl]-1,3,4-thiadiazol-2-yl]propanamide hydrochloride (compound **34**, 75 % purity, 49.8 mg, 100 µmol, 1.0 eq.) were dissolved in DMF (0.8 mL) and 4-methylmorpholine (46 mg, 50.0 µL, 455 µmol, 4.6 eq.) was added. The reaction mixture was stirred at room temperature overnight. A few drops of water were added, and the reaction mixture was purified using preparative HPLC (water/acetonitrile gradient) to yield the desired compound **9** (33.2 mg, 100% purity, 72 % yield). **LC-MS** (Method A): R_t = 1.85 min; **HRMS:** m/z $[M+H]^+$ calcd for $C_{21}H_{28}N_5O_5S$: 462.1811, found 462.1806. **^{1}H NMR** (500 MHz, DMSO-d_6) δ ppm 1.04 (s, 3 H), 1.12 - 1.48 (m, 9 H), 1.95 (br d, *J*=12.66 Hz, 2 H), 3.25 (s, 3 H), 3.57 - 3.68 (m, 2 H), 4.51 - 4.56 (m, 2 H), 4.72 (m, 1 H), 7.57 (d, *J*=7.02 Hz, 1 H), 7.60 - 7.65 (m, 2 H), 8.19 - 8.23 (m, 2 H), 12.66 (br s, 1 H). **^{13}C NMR** (126 MHz, DMSO-d_6) δ ppm 23.0, 23.1, 25.8, 26.8, 34.9, 35.49, 35.52, 42.5, 53.5, 58.8, 71.5, 124.3, 130.7, 146.0, 147.0, 159.3, 162.4, 170.1, 177.5.

Synthesis of compound 10

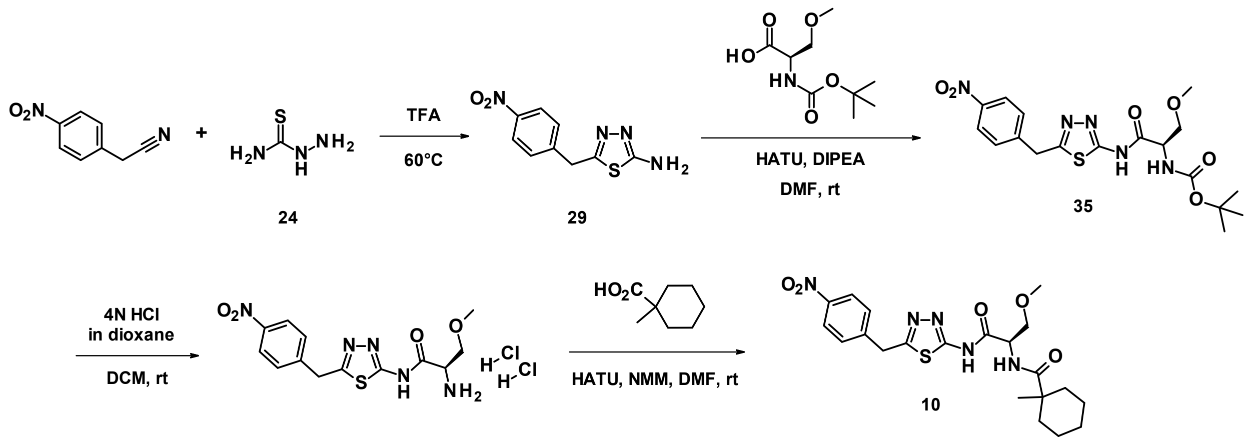

tert-butyl N-[(1R)-1-(methoxymethyl)-2-[[5-[(4-nitrophenyl)methyl]-1,3,4-thiadiazol-2-yl]amino]-2-oxo-ethyl]carbamate (**35**):

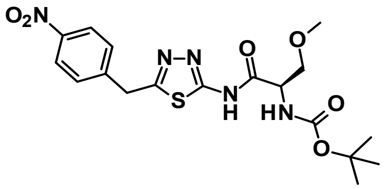

5-[(4-nitrophenyl) methyl]-1,3,4-thiadiazol-2-amine (compound **29**, 300 mg, 1.27 mmol, 1.0 eq.), HATU (724 mg, 1.91 mmol, 1.5 eq.) and N-(tert-butoxycarbonyl)-O-methyl-D-serine (373 mg, 97% purity, 1.65 mmol, 1.3 eq.) were dissolved in DMF (7.0 mL) and 4-methylmorpholine (385 mg, 419 µL, 3.81 mmol, 3.0 eq.) was added. The reaction mixture was stirred at room temperature overnight. A few drops of water were added, and the reaction mixture was purified using preparative HPLC (water/acetonitrile gradient) to yield the desired compound **35** (524 mg, 100% purity, 94 % yield). **LC-MS** (Method A): R_t = 1.70 min; **HRMS:** m/z $[M+H]^+$ calcd for $C_{18}H_{24}N_5O_6S$: 438.1447, found 438.1442. **^{1}H NMR** (600 MHz, DMSO-d_6) δ ppm 1.36 (s, 9 H), 3.23 (s, 3 H), 3.49 - 3.57 (m, 2 H), 4.42 - 4.48 (m, 1 H), 4.55 (s, 2 H), 7.21 (br d, *J*=7.04 Hz, 1 H), 7.62 (d, *J*=8.61 Hz, 2 H), 8.21 (d, *J*=8.61 Hz, 2 H), 12.68 (br s, 1 H).

N-[(1R)-1-(methoxymethyl)-2-[[5-[(4-nitrophenyl)methyl]-1,3,4-thiadiazol-2-yl]amino]-2-oxo-ethyl]-1-methyl-cyclohexanecarboxamide (**10**):

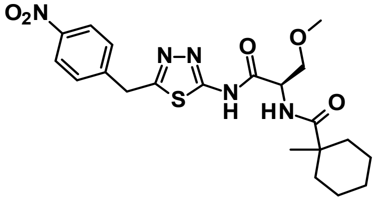

Step 1:

tert-butyl N-[(1R)-1-(methoxymethyl)-2-[[5-[(4-nitrophenyl)methyl]-1,3,4-thiadiazol-2-yl]amino]-2-oxo-ethyl]carbamate (compound **35**, 100% purity, 524 mg, 1.20 mmol, 1.0 eq.) was dissolved in DCM (8.0 mL) and 4 N HCl in dioxane (2.99 mL, 12.0 mmol, 10.0 eq.) was added. After stirring for 1.5 h at room temperature, the reaction mixture was concentrated. The residue was repeatedly suspended in DCM, concentrated under reduced pressure and finally dried *in vacuo* to yield (2R)-2-amino-3-methoxy-N-[5-[(4-nitrophenyl)methyl]-1,3,4-thiadiazol-2-yl]propanamide dihydrochloride (454 mg, 100 % purity, 92 % yield). **LC-MS** (Method A): R_t = 0.79 min; **HRMS:** m/z $[M+H-2HCl]^+$ calcd for $C_{13}H_{16}N_5O_4S$: 338.0923, found 338.0918.

Step 2:

1-Methylcyclohexanecarboxylic acid (42.7 mg, 300 µmol, 2.0 eq.), HATU (79.8 mg, 210 µmol, 1.4 eq.) and 2-amino-3-methoxy-N-[5-[(4-nitrophenyl)methyl]-1,3,4-thiadiazol-2-yl]propanamide dihydrochloride (100 % purity, 61.5 mg, 150 µmol, 1.0 eq.) were dissolved in DMF (0.8 mL) and 4-methylmorpholine (46 mg, 50.0 µL, 455 µmol, 3.0 eq.) was added. The reaction mixture was stirred at room temperature overnight. A few drops of water were added, and the reaction mixture was purified using preparative HPLC (water/acetonitrile gradient) to yield the desired compound **10** (39.5 mg, 100% purity, 57 % yield). **LC-MS** (Method A): R_t = 1.87 min; **HRMS:** m/z $[M+H]^+$

calcd for $C_{21}H_{28}N_5O_5S$: 462.1811, found 462.1806. **^{1}H NMR** (600 MHz, DMSO-d_6) δ ppm 1.04 (s, 3 H), 1.13 - 1.35 (m, 5 H), 1.38 - 1.48 (m, 3 H), 1.95 (br d, J=12.52 Hz, 2 H), 3.25 (s, 3 H), 3.57 - 3.69 (m, 2 H), 4.54 (s, 2 H), 4.72 (m, 1 H), 7.57 (d, J=7.04 Hz, 1 H), 7.62 (d, J=8.41 Hz, 2 H), 8.21 (d, J=8.41 Hz, 2 H), 12.66 (s, 1 H). **^{13}C NMR** (126 MHz, DMSO-d_6) δ ppm 22.5, 22.5, 25.3, 26.2, 34.4, 34.9, 35.0, 41.9, 53.0, 58.2, 70.9, 123.8, 130.1, 145.5, 146.5, 158.7, 161.9, 169.5, 176.9. **$[\alpha]_D^{20}$** = +78.24 (c = 0.190 in MeOH)

Synthesis of compound 11 and 14 – 17

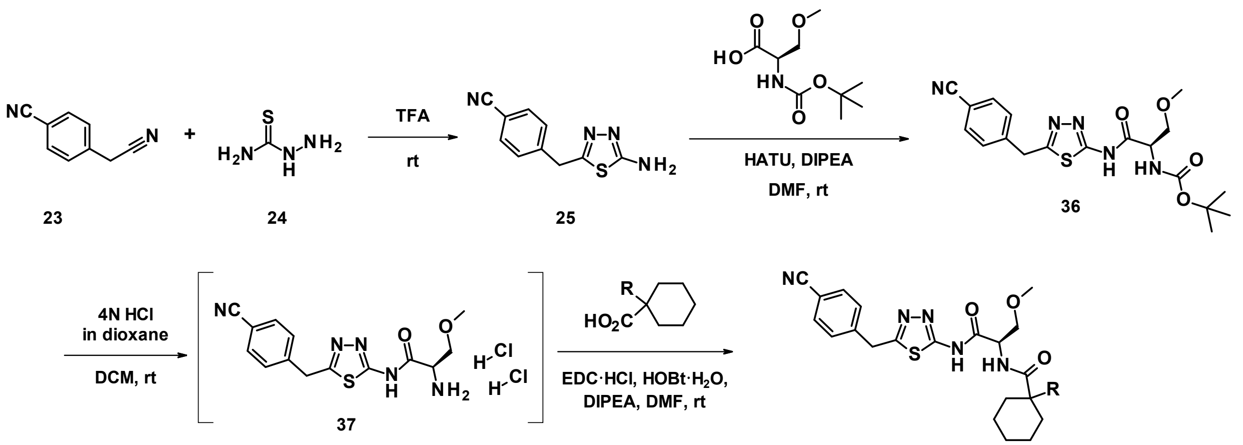

tert-butyl N-[(1R)-2-[[5-[(4-cyanophenyl)methyl]-1,3,4-thiadiazol-2-yl]amino]-1-(methoxymethyl)-2-oxo-ethyl]carbamate (**36**)*:*

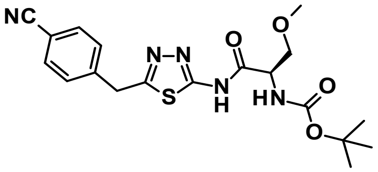

(2R)-2-(tert-butoxycarbonylamino)-3-methoxy-propanoic acid (837 mg, 3.70 mmol, 1.3 eq.), 4-[(5-amino-1,3,4-thiadiazol-2-yl)methyl]benzonitrile (compound **25**, 616 mg, 2.85 mmol, 1.0 eq.) and HATU (1.62 g, 4.27 mmol, 1.5 eq.) were dissolved in DMF (12.0 mL). 4-Methylmorpholine

(864 mg, 0.939 mL, 8.55 mmol, 3.0 eq.) was added and the reaction mixture was stirred overnight at room temperature. The crude mixture was purified using preparative HPLC (water/acetonitrile gradient) to yield the desired compound **36** (1.05 g, 100% purity, 88 % yield). **LC-MS** (Method A): R_t = 1.61 min; **HRMS:** m/z $[M+H]^+$ calcd for $C_{19}H_{24}N_5O_4S$: 418.1549, found 418.1544. **^{1}H NMR** (600 MHz, DMSO-d_6) δ ppm 1.37 (s, 9 H), 3.23 (s, 3 H), 3.50 - 3.55 (m, 2 H), 4.42 - 4.47 (m, 1 H), 4.48 (s, 2 H), 7.16 - 7.25 (m, 1 H), 7.55 (d, *J*=8.22 Hz, 2 H), 7.82 (d, *J*=8.22 Hz, 2 H), 12.66 (br s, 1 H).

*(2R)-2-amino-N-[5-[(4-cyanophenyl)methyl]-1,3,4-thiadiazol-2-yl]-3-methoxy-propanamide dihydrochloride (**37**):*

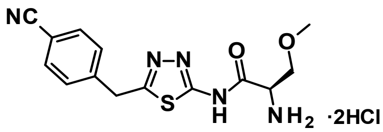

tert-butyl N-[(1R)-2-[[5-[(4-cyanophenyl)methyl]-1,3,4-thiadiazol-2-yl]amino]-1-(methoxy-methyl)-2-oxo-ethyl]carbamate (compound **36**, 1.05 g, 2.52 mmol, 1.0 eq.) was dissolved in DCM (15 mL) and 4 N HCl in dioxane (6.29 mL, 25.2 mmol, 10.0 eq.) was added. After stirring for 1.5 h at room temperature, the reaction mixture was concentrated and dried *in vacuo* to yield (2R)-2-amino-N-[5-[(4-cyanophenyl)methyl]-1,3,4-thiadiazol-2-yl]-3-methoxy-propanamide dihydrochloride (917 mg, 100 % purity, 93 % yield). **LC-MS** (Method A): R_t = 0.73 min; **HRMS:** m/z $[M+H-2HCl]^+$ calcd for $C_{14}H_{16}N_5O_2S$: 318.1024, found 318.1019. **^{1}H NMR** (600 MHz, DMSO-d_6) δ ppm 3.28 (s, 3 H), 3.77 - 3.84 (m, 1 H), 3.85 - 3.90 (m, 1 H), 4.34 - 4.38 (m, 1 H), 4.52 (s, 2 H), 7.53 - 7.60 (m, 2 H), 7.82 (d, *J*=8.22 Hz, 2 H), 8.57 - 8.82 (m, 3 H).

N-[(1R)-2-[[5-[(4-cyanophenyl)methyl]-1,3,4-thiadiazol-2-yl]amino]-1-(methoxymethyl)-2-oxo-ethyl]-1-methyl-cyclohexanecarboxamide (**11**):

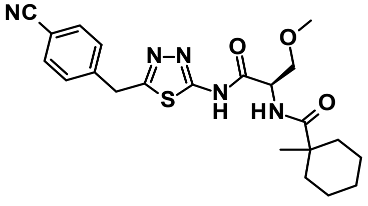

(2R)-2-amino-N-[5-[(4-cyanophenyl)methyl]-1,3,4-thiadiazol-2-yl]-3-methoxy-propanamide dihydrochloride (compound **37**, 58.5 mg, 150 μmol, 1.0 eq.), 1-methylcyclohexanecarboxylic acid (42.7 mg, 300 μmol, 2.0 eq.) and HATU (79.8 mg, 210 μmol, 1.4 eq.) were dissolved in DMF (0.80 mL) and 4-methylmorpholine (46.0 mg, 40 μL, 455 μmol, 3.0 eq.) was added. The reaction mixture was stirred at room temperature overnight and then passed through a syringe filter. The filtrate was purified using preparative HPLC (water/acetonitrile gradient) to yield the desired compound **11** (35.7 mg, 100% purity, 54 % yield). **LC-MS** (Method A): R_t = 1.78 min; **HRMS:** m/z $[M+H]^+$ calcd for $C_{22}H_{28}N_5O_3S$: 442.1912, found 442.1908. **^{1}H NMR** (600 MHz, DMSO-*d*$_6$) δ ppm 1.05 (s, 3 H), 1.13 - 1.35 (m, 5 H), 1.38 - 1.52 (m, 3 H), 1.88 - 2.03 (m, 2 H), 3.25 (s, 3 H), 3.56 - 3.69 (m, 2 H), 4.48 (s, 2 H), 4.68 - 4.76 (m, 1 H), 7.52 - 7.55 (m, 2 H), 7.57 (d, *J*=7.04 Hz, 1 H), 7.81 (d, *J*=8.22 Hz, 2 H), 12.60 - 12.69 (m, 1 H). **^{13}C NMR** (126 MHz, DMSO-*d*$_6$) δ ppm 22.6, 22.5, 25.3, 26.2, 34.6, 34.9, 34.9, 41.9, 52.9, 58.2, 70.9, 109.8, 118.6, 129.9, 132.5, 143.4, 158.7, 162.0, 169.5, 176.9. **$[\alpha]_D^{20}$** = +42.08 (c = 0.254 in MeOH).

N-[(1R)-2-[[5-[(4-cyanophenyl)methyl]-1,3,4-thiadiazol-2-yl]amino]-1-(methoxymethyl)-2-oxo-ethyl]-1-ethyl-cyclohexanecarboxamide (**14**):

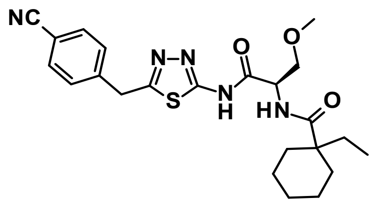

1-Ethylcyclohexanecarboxylic acid (46.8 mg, 300 µmol, 1.5 eq.), EDC·HCl (57.5 mg, 300 µmol, 1.5 eq.) and HOBt Hydrate (45.9 mg, 300 µmol, 1.5 eq.) were dissolved in DMF (0.90 mL). DIPEA (129 mg, 174 µL, 1.00 mmol, 5.0 eq.) and (2R)-2-amino-N-[5-[(4-cyanophenyl)methyl]-1,3,4-thiadiazol-2-yl]-3-methoxy-propanamide dihydrochloride (compound **37**, 78.0 mg, 200 µmol, 1.0 eq.) were added and the reaction mixture was stirred for three days at room temperature. A few drops of water were added, and the reaction mixture was purified using preparative HPLC (water/acetonitrile gradient) to yield the desired compound **14** (52.3 mg, 100% purity, 57 % yield).

LC-MS (Method A): R_t = 1.88 min; **HRMS:** m/z $[M+H]^+$ calcd for $C_{23}H_{30}N_5O_3S$: 456.2069, found 456.2064. **^{1}H NMR** (600 MHz, DMSO-*d*$_6$) δ ppm 0.68 (t, *J*=7.52 Hz, 3 H), 1.06 - 1.35 (m, 5 H), 1.37 - 1.49 (m, 5 H), 1.99 (br d, *J*=13.20 Hz, 2 H), 3.25 (s, 3 H), 3.58 - 3.67 (m, 2 H), 4.47 (s, 2 H), 4.73 - 4.79 (m, 1 H), 7.53 - 7.58 (m, 3 H), 7.81 (d, *J*=8.44 Hz, 2 H), 12.65 (s, 1 H). **^{13}C NMR** (126 MHz, DMSO-*d*$_6$) δ ppm 8.07, 22.51, 22.56, 25.59, 32.68, 33.12, 33.33, 34.59, 45.77, 52.82, 58.13, 70.87, 109.79, 118.65, 129.88, 132.54, 143.35, 158.62, 162.04, 169.50, 175.51. **$[\alpha]_D^{20}$** = +11.71 (c = 0.205 in MeOH).

N-[(1R)-2-[[5-[(4-cyanophenyl)methyl]-1,3,4-thiadiazol-2-yl]amino]-1-(methoxymethyl)-2-oxo-ethyl]-1-methoxy-cyclohexanecarboxamide (**15**):

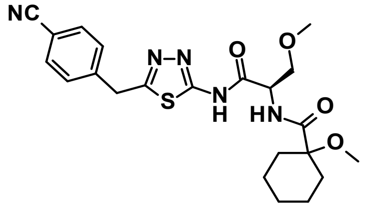

1-Methoxycyclohexanecarboxylic acid (47.4 mg, 300 µmol, 1.5 eq.), EDC·HCl (57.5 mg, 300 µmol, 1.5 eq.) and HOBt Hydrate (45.9 mg, 300 µmol, 1.5 eq.) were dissolved in DMF (0.90 mL). DIPEA (129 mg, 174 µL, 1.00 mmol, 5.0 eq.) and (2R)-2-amino-N-[5-[(4-cyanophenyl)methyl]-1,3,4-thiadiazol-2-yl]-3-methoxy-propanamide dihydrochloride (compound **37**, 78.0 mg, 200 µmol, 1.0 eq.) were added and the reaction mixture was stirred for three days at room temperature. A few drops of water were added, and the reaction mixture was purified using preparative HPLC (water/acetonitrile gradient) to yield the desired compound **15** (51.2 mg, 100% purity, 56 % yield).

LC-MS (Method B): R_t = 1.81 min; **HRMS:** m/z $[M+H]^+$ calcd for $C_{22}H_{28}N_5O_4S$: 458.1862, found 458.1857. **^{1}H NMR** (600 MHz, DMSO-d_6) δ ppm 1.15 - 1.24 (m, 1 H), 1.39 - 1.50 (m, 4 H), 1.52 - 1.62 (m, 3 H), 1.72 (br d, *J*=13.57 Hz, 2 H), 3.13 (s, 3 H), 3.24 (s, 3 H), 3.62 (m, 1 H), 3.67 - 3.72 (m, 1 H), 4.48 (s, 2 H), 4.68 - 4.75 (m, 1 H), 7.54 (d, *J*=8.25 Hz, 2 H), 7.81 (d, *J*=8.07 Hz, 3 H), 12.71 (s, 1 H). **^{13}C NMR** (126 MHz, DMSO-d_6) δ ppm 20.71, 20.78, 24.71, 30.56, 31.21, 34.59, 50.69, 52.51, 58.26, 70.98, 78.94, 109.78, 118.64, 129.87, 132.55, 143.33, 158.57, 162.20, 168.93, 174.33. **$[\alpha]_D^{20}$** = +15.05 (c = 0.202 in MeOH).

N-[(1R)-2-[[5-[(4-cyanophenyl)methyl]-1,3,4-thiadiazol-2-yl]amino]-1-(methoxymethyl)-2-oxo-ethyl]-1-(difluoromethyl)cyclohexanecarboxamide (**16**):

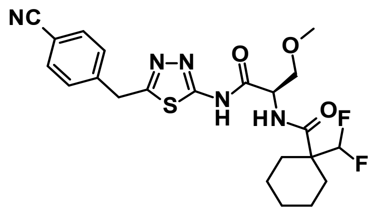

1-(Difluoromethyl)cyclohexanecarboxylic acid (53.4 mg, 300 µmol, 1.5 eq.), EDC·HCl (57.5 mg, 300 µmol, 1.5 eq.) and HOBt Hydrate (45.9 mg, 300 µmol, 1.5 eq.) were dissolved in DMF (0.90 mL). DIPEA (129 mg, 174 µL, 1.00 mmol, 5.0 eq.) and (2R)-2-amino-N-[5-[(4-cyanophenyl)methyl]-1,3,4-thiadiazol-2-yl]-3-methoxy-propanamide dihydrochloride (compound **37**, 78.0 mg, 200 µmol, 1.0 eq.) were added and the reaction mixture was stirred for three days at room temperature. A few drops of water were added, and the reaction mixture was purified using preparative HPLC (water/acetonitrile gradient) to yield the desired compound **16** (67.8 mg, 100% purity, 71 % yield). **LC-MS** (Method B): R_t = 1.84 min; **HRMS:** m/z $[M+H]^+$ calcd for $C_{22}H_{26}F_2N_5O_3S$: 478.1724, found 478.1719. **^{1}H NMR** (600 MHz, DMSO-d_6) δ ppm 1.07 - 1.16 (m, 1 H), 1.21 - 1.34 (m, 4 H), 1.52 - 1.58 (m, 3 H), 2.09 - 2.21 (m, 2 H), 3.25 (s, 3 H), 3.60 - 3.64 (m, 1 H), 3.65 - 3.71 (m, 1 H), 4.48 (s, 2 H) 4.80 (m, 1 H), 5.79 - 6.06 (m, 1 H), 7.55 (d, *J*=8.07 Hz, 2 H), 7.81 (d, *J*=8.07 Hz, 2 H), 7.92 (br d, *J*=6.79 Hz, 1 H), 12.72 (s, 1 H). **^{13}C NMR** (126 MHz, DMSO-d_6) δ ppm 21.55, 24.90, 26.36 - 26.69 (m) 34.58, 50.34 (t, $J_{C\text{-}F}$ = 17.92 Hz) 53.18, 58.19, 70.68, 109.78, 118.06, 118.65 (t, $J_{C\text{-}F}$ = 245 Hz), 129.87, 132.55, 143.33, 158.58, 162.14, 168.81, 169.33. **$[\alpha]_D^{20}$** = +15.13 (c = 0.185 in MeOH)

N-[(1R)-2-[[5-[(4-cyanophenyl)methyl]-1,3,4-thiadiazol-2-yl]amino]-1-(methoxymethyl)-2-oxo-ethyl]-1-(trifluoromethyl)cyclohexanecarboxamide (**17**):

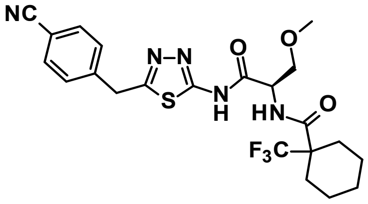

1-(Trifluoromethyl)cyclohexanecarboxylic acid (58.8 mg, 300 µmol, 1.5 eq.), EDC·HCl (57.5 mg, 300 µmol, 1.5 eq.) and HOBt Hydrate (45.9 mg, 300 µmol, 1.5 eq.) were dissolved in DMF (0.89 mL). DIPEA (129 mg, 174 µL, 1.00 mmol, 5.0 eq.) and (2R)-2-amino-N-[5-[(4-cyanophenyl) methyl]-1,3,4-thiadiazol-2-yl]-3-methoxy-propanamide dihydrochloride (compound **37**, 78.0 mg, 200 µmol, 1.0 eq.) were added and the reaction mixture was stirred for three days at room temperature. The crude mixture was purified using preparative HPLC (water/acetonitrile gradient) to yield the desired compound **17** (71.2 mg, 100 % purity, 72 % yield). **LC-MS** (Method B): R_t = 1.95 min; **HRMS:** m/z $[M+H]^+$ calcd for $C_{22}H_{25}F_3N_5O_3S$: 496.1630, found 496.1625. **^{1}H NMR** (600 MHz, DMSO-*d*$_6$) δ ppm 1.08 - 1.24 (m, 2 H), 1.28 - 1.48 (m, 3 H), 1.53 - 1.68 (m, 3 H), 2.31 - 2.47 (m, 2 H), 3.26 (s, 3 H), 3.60 - 3.73 (m, 2 H), 4.48 (s, 2 H), 4.78 - 4.87 (m, 1 H), 7.55 (d, *J*=8.44 Hz, 2 H), 7.81 (d, *J*=8.25 Hz, 2 H), 8.18 (d, *J*=6.79 Hz, 1 H), 12.77 (s, 1 H). **^{13}C NMR** (126 MHz, DMSO-*d*$_6$) δ ppm 21.5, 21.5, 24.3, 26.6, 26.7, 34.6, 51.5, 51.6, 53.5, 58.1, 70.3, 109.8, 118.6, 125.2, 127.5, 129.9, 132.5, 143.3, 158.6, 162.1, 166.5, 168.6. **$[\alpha]_D^{20}$** = +13.97 (c = 0.251 in MeOH).

Synthesis of compound 12

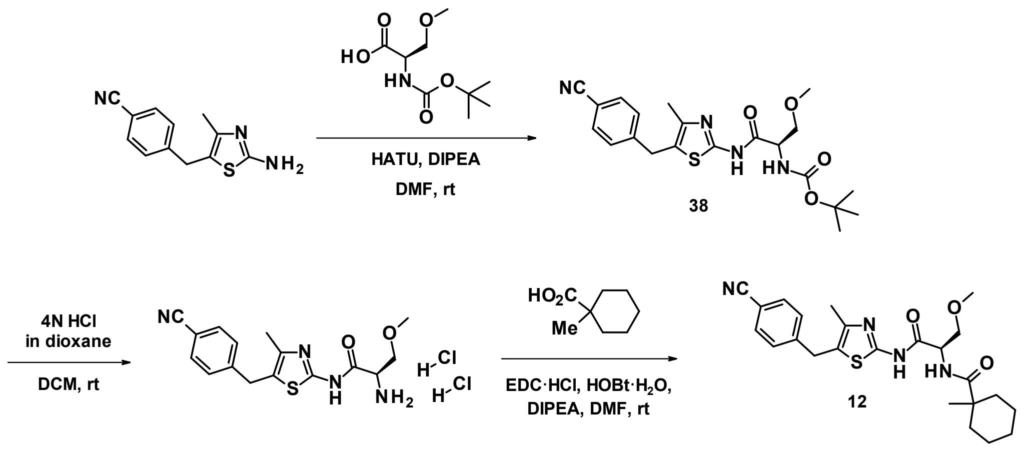

tert-butyl N-[(1R)-2-[[5-[(4-cyanophenyl)methyl]-4-methyl-thiazol-2-yl]amino]-1-(methoxymethyl)-2-oxo-ethyl]carbamate (**38**):

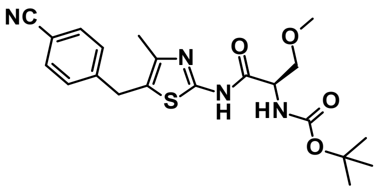

4-[(2-amino-4-methyl-thiazol-5-yl)methyl]benzonitrile (250 mg, 1.09 mmol, 1.0 eq.), HATU (622 mg, 1.64 mmol, 1.5 eq.) and N-(tert-butoxycarbonyl)-O-methyl-D-serine (320 mg, 97% purity, 1.42 mmol, 1.3 eq.) were dissolved in DMF (5.0 mL) and 4-methylmorpholine (331 mg, 360 µL, 3.27 mmol, 3.0 eq.) was added. The reaction mixture was stirred at room temperature overnight. The reaction mixture was purified using preparative HPLC (water + 0.05% TFA/acetonitrile gradient) to yield the desired compound **38** (492 mg, 100% purity, quant.). **LC-MS** (Method A): R_t = 1.84 min; **HRMS:** m/z $[M+H]^+$ calcd for $C_{21}H_{27}N_4O_4S$: 431.1753, found 431.1748. **^{1}H NMR** (600 MHz, DMSO-d_6) δ ppm 1.37 (s, 9 H), 2.23 (s, 3 H), 3.23 (s, 3 H), 3.45 - 3.55 (m, 2 H), 4.13

(s, 2 H), 4.37 - 4.43 (m, 1 H), 6.94 - 7.21 (m, 1 H), 7.42 (d, *J*=8.22 Hz, 2 H), 7.77 (d, *J*=8.02 Hz, 2 H), 12.06 (br s, 1 H).

N-[(1R)-2-[[5-[(4-cyanophenyl)methyl]-4-methyl-thiazol-2-yl]amino]-1-(methoxymethyl)-2-oxo-ethyl]-1-methyl-cyclohexanecarboxamide (**12**):

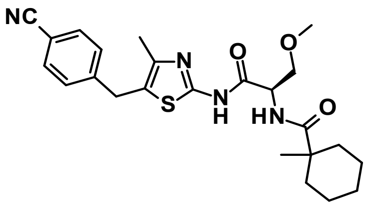

Step 1:

tert-butyl N-[(1R)-2-[[5-[(4-cyanophenyl)methyl]-4-methyl-thiazol-2-yl]amino]-1-(methoxy-methyl)-2-oxo-ethyl]carbamate (compound **38**, 100% purity, 492 mg, 1.14 mmol, 1.0 eq.) was dissolved in DCM (10 mL) and 4 N HCl in dioxane (2.86 mL, 11.4 mmol, 10.0 eq.) was added. After stirring for 1.5 h at room temperature, the reaction mixture was concentrated. The residue was repeatedly suspended in DCM, concentrated under reduced pressure and finally dried *in vacuo* to yield (2R)-2-amino-N-[5-[(4-cyanophenyl)methyl]-4-methyl-thiazol-2-yl]-3-methoxy-propan-amide dihydrochloride (473 mg, 100 % purity, quant.). **LC-MS** (Method A): R_t = 0.94 min; **HRMS:** m/z $[M+H-2HCl]^+$ calcd for $C_{16}H_{19}N_4O_2S$: 331.1229, found 331.1223.

Step 2:

1-Methylcyclohexanecarboxylic acid (42.7 mg, 300 µmol, 2.0 eq.), HATU (79.8 mg, 210 µmol, 1.4 eq.) and (2R)-2-amino-N-[5-[(4-cyanophenyl)methyl]-4-methyl-thiazol-2-yl]-3-methoxy-propanamide dihydrochloride (100 % purity, 60.5 mg, 150 µmol, 1.0 eq.) were dissolved in DMF (0.8 mL) and 4-methylmorpholine (46 mg, 50.0 µL, 455 µmol, 3.0 eq.) was added. The reaction mixture was stirred at room temperature overnight. A few drops of water were added, and the

reaction mixture was purified using preparative HPLC (water/acetonitrile gradient) to yield the desired compound **12** (33.6 mg, 97% purity, 48 % yield). **LC-MS** (Method A): R_t = 1.99 min; **HRMS:** m/z $[M+H]^+$ calcd for $C_{24}H_{31}N_4O_3S$: 455.2117, found 455.2112. **^{1}H NMR** (600 MHz, DMSO-d_6) δ ppm 1.05 (s, 3 H), 1.13 - 1.37 (m, 5 H), 1.38 - 1.50 (m, 3 H), 1.87 - 1.99 (m, 2 H), 2.23 (s, 3 H), 3.24 (s, 3 H), 3.52 - 3.66 (m, 2 H), 4.13 (s, 2 H), 4.69 (m, 1 H), 7.42 (d, *J*=8.22 Hz, 2 H), 7.46 (d, *J*=7.43 Hz, 1 H), 7.77 (d, *J*=8.22 Hz, 2 H), 12.05 (br s, 1 H). **$[\alpha]_D^{20}$** = +27.14 (c = 0.210 in $CHCl_3$)

Synthesis of compound 13

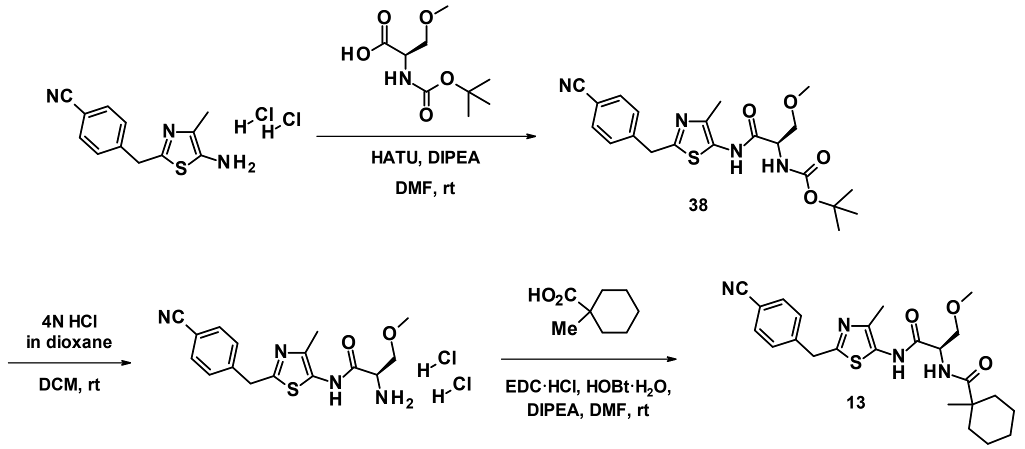

<u>*N-[(1R)-2-[[2-[(4-cyanophenyl)methyl]-4-methyl-thiazol-5-yl]amino]-1-(methoxymethyl)-2-oxo-ethyl]-1-methyl-cyclohexanecarboxamide* (**13**):</u>

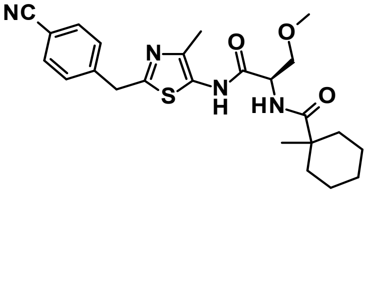

<u>*Step 1:*</u>

Sequentially, N-(tert-butoxycarbonyl)-O-methyl-D-serine (87.7 mg, 400 µmol, 2.0 eq.) dissolved in DMF (0.4 mL), HATU (106 mg, 280 µmol, 1.4 eq.) dissolved in DMF (0.4 mL) and 4-methylmorpholine (100 µL, 92.0 mg, 910 mmol, 4.55 eq.) were added to 4-[(5-amino-4-methyl-thiazol-2-yl)methyl]benzonitrile dihydrochloride (60.4 mg, 0.2 mmol, 1.0 eq.). The reaction mixture was stirred at room temperature overnight and filtered. Purification with preparative HPLC (water/acetonitrile gradient) yielded tert-butyl N-[(1R)-2-[[2-[(4-cyanophenyl)methyl]-4-methyl-thiazol-5-yl]amino]-1-(methoxy-methyl)-2-oxo-ethyl]carbamate (compound **38**).

Step 2:

tert-butyl N-[(1R)-2-[[2-[(4-cyanophenyl)methyl]-4-methyl-thiazol-5-yl]amino]-1-(methoxy-methyl)-2-oxo-ethyl]carbamate (compound **38**) was dissolved in DCM (0.5 mL). 4 N HCl in dioxane (0.500 mL, 2.00 mmol, 10.0 eq.) was added, stirred overnight at room temperature and concentrated under reduced pressure.

Step 3:

1-Methyl-cyclohexanecarboxylic acid (56.9 mg, 0.400 mmol, 2.0 eq.) dissolved in DMF (0.4 mL), HATU (107 mg, 0.280 mmol, 1.4 eq.) dissolved in DMF (0.4 mL) and 4-methylmorpholine (100 µL, 92.0 mg, 910 mmol, 4.55 eq.) were added. The reaction mixture was stirred at room temperature overnight and purified using preparative HPLC (water/acetonitrile gradient) to yield the desired compound **13** (14.4 mg, 100% purity, 8 % yield over all three steps). **LC-MS** (Method A): R_t = 1.86 min; **HRMS:** m/z $[M+H]^+$ calcd for $C_{24}H_{31}N_4O_3S$: 455.2116, found 455.2112. **^{1}H NMR** (500 MHz, DMSO-d_6) δ ppm 1.05 (s, 3 H), 1.13 - 1.36 (m, 5 H), 1.38 - 1.50 (m, 3 H), 1.92 - 2.00 (m, 2 H), 2.29 (s, 3 H), 3.26 (s, 3 H), 3.58 (d, *J*=6.41 Hz, 2 H), 4.28 (s, 2 H), 4.74 - 4.83 (m, 1 H), 7.47 - 7.53 (m, 3 H), 7.77 - 7.81 (m, 2 H), 10.42 (s, 1 H). **^{13}C NMR** (126 MHz, DMSO-d_6)

δ ppm 15.0, 23.1, 25.8, 26.9, 35.5, 35.6, 38.5, 40.9, 42.5, 53.0, 58.8, 71.9, 110.1, 119.3, 128.1, 130.4, 133.0, 137.7, 144.8, 159.4, 168.6, 177.3. $[\alpha]_D^{20}$ = +19.01 (c = 0.235 in $CHCl_3$).

Synthesis of compound 18

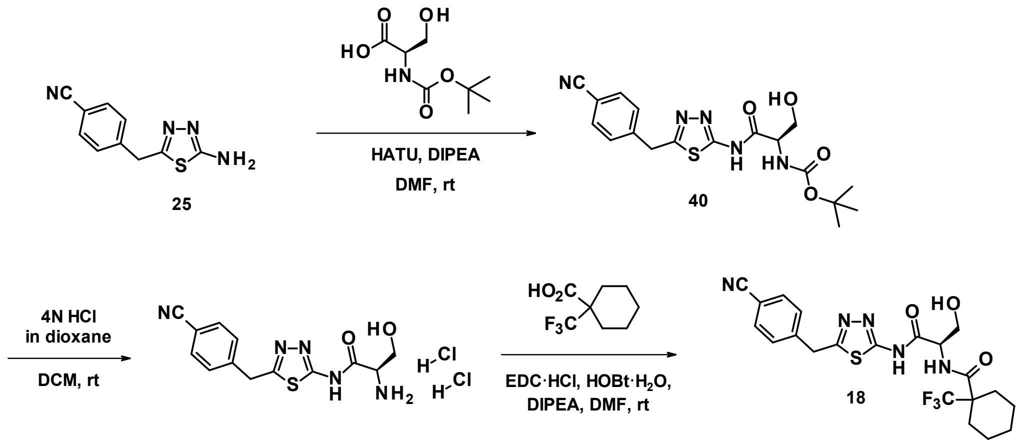

tert-butyl N-[(1R)-2-[[5-[(4-cyanophenyl)methyl]-1,3,4-thiadiazol-2-yl]amino]-1-(hydroxy-methyl)-2-oxo-ethyl]carbamate (**40**):

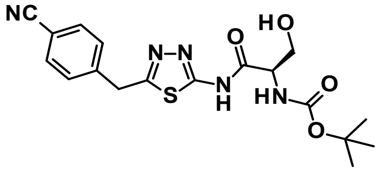

(2R)-2-(tert-butoxycarbonylamino)-3-hydroxy-propanoic acid (91.1 mg, 444 µmol, 1.2 eq.) and HATU (211 mg, 555 µmol, 1.5 eq.) were dissolved in DMF (0.87 mL) and DIPEA (95.6 mg, 129 µL, 740 µmol, 2.0 eq.) was added. After stirring for 15 min at room temperature, 4-[(5-amino-1,3,4-thiadiazol-2-yl)methyl]benzonitrile (compound **25**, 80 mg, 370 µmol, 1.0 eq.) was added and

the reaction mixture was stirred at room temperature for 3 days. A few drops of water were added, and the reaction mixture was purified using preparative HPLC (water/acetonitrile gradient) to yield the desired compound **40** (75.3 mg, 100% purity, 50 % yield). **LC-MS** (Method A): R_t = 1.39 min; **HRMS:** m/z $[M+H]^+$ calcd for $C_{18}H_{22}N_5O_4S$: 404.1392, found 404.1387. **^{1}H NMR** (600 MHz, DMSO-d_6) δ ppm 1.37 (s, 9 H), 3.56 - 3.69 (m, 2 H), 4.21 - 4.32 (m, 1 H), 4.48 (s, 2 H), 4.89 - 5.05 (m, 1 H), 6.86 - 6.96 (m, 1 H), 7.54 (d, *J*=8.02 Hz, 2 H), 7.81 (d, *J*=8.02 Hz, 2 H), 12.56 (br s, 1 H).

N-[(1R)-2-[[5-[(4-cyanophenyl)methyl]-1,3,4-thiadiazol-2-yl]amino]-1-(hydroxymethyl)-2-oxo-ethyl]-1-(trifluoromethyl)cyclohexanecarboxamide (**18**):

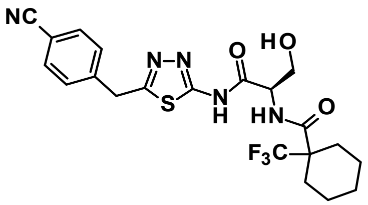

Step 1:

tert-butyl N-[(1R)-2-[[5-[(4-cyanophenyl)methyl]-1,3,4-thiadiazol-2-yl]amino]-1-(hydroxy-methyl)-2-oxo-ethyl]carbamate (compound **40**, 100 % purity, 73.0 mg, 181 µmol, 1.0 eq.) was dissolved in DCM (1.5 mL) and 4 N HCl in dioxane (452 µL, 1.81 mmol, 10.0 eq.) was added. After stirring for 3 h at room temperature, the reaction mixture was concentrated and dried *in vacuo* to yield (2R)-2-amino-N-[5-[(4-cyanophenyl)methyl]-1,3,4-thiadiazol-2-yl]-3-hydroxy-propan-amide dihydrochloride (67.3 mg, 100 % purity, 99 % yield). **LC-MS** (Method A): R_t = 0.59 min; **HRMS:** m/z $[M+H-2HCl]^+$ calcd for $C_{13}H_{14}N_5O_2S$: 304.0868, found 304.0863.

Step 2:

1-(Trifluoromethyl)cyclohexanecarboxylic acid (52.4 mg, 267 µmol, 1.5 eq.), EDC·HCl (51.2 mg, 267 µmol, 1.5 eq.) and HOBt Hydrat (40.9 mg, 267 µmol, 1.5 eq.) were dissolved in DMF (0.80 mL). DIPEA (115 mg, 155 µL, 890 µmol, 5.0 eq.) and (2R)-2-amino-N-[5-[(4-cyano-phenyl)methyl]-1,3,4-thiadiazol-2-yl]-3-hydroxy-propanamide dihydrochloride (67.0 mg, 178 µmol, 1.0 eq.) were added and the reaction mixture was stirred overnight at room temperature. The crude mixture was purified using preparative HPLC (water/acetonitrile gradient) to yield the desired compound **18** (41.6 mg, 100 % purity, 49 % yield). **LC-MS** (Method A): R_t = 1.67 min; **HRMS:** m/z $[M+H]^+$ calcd for $C_{21}H_{23}F_3N_5O_3S$: 482.1473, found 482.1468. **^{1}H NMR** (600 MHz, DMSO-d_6) δ ppm 1.09 - 1.26 (m, 2 H), 1.34 - 1.51 (m, 3 H), 1.53 - 1.68 (m, 3 H), 2.27 - 2.41 (m, 2 H), 3.67 - 3.84 (m, 2 H), 4.48 (s, 2 H), 4.55 - 4.65 (m, 1 H), 5.10 (br s, 1 H), 7.54 (d, *J*=8.41 Hz, 2 H), 7.81 (d, *J*=8.41 Hz, 2 H), 7.95 (d, *J*=6.65 Hz, 1 H), 12.66 (br s, 1 H). **^{13}C NMR** (126 MHz, DMSO-d_6) δ ppm 21.40, 21.52, 24.31, 26.58, 26.84, 34.58, 51.24, 51.43, 51.61, 56.22, 60.76, 109.76, 118.65, 125.21, 127.46, 129.85, 132.54, 143.38, 158.73, 161.97, 166.34, 168.89. **$[\alpha]_D^{20}$** = +52.05 (c = 0.244 in MeOH)

Synthesis of compound 19

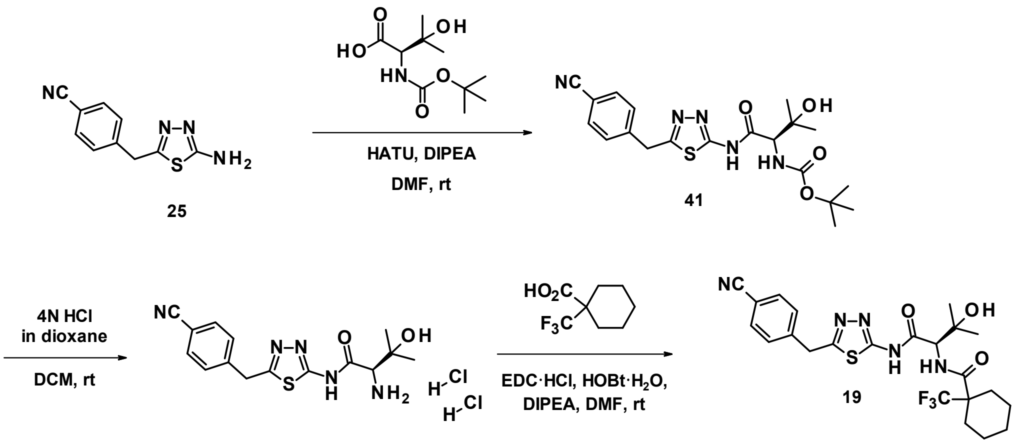

tert-butyl N-[(1R)-1-[[5-[(4-cyanophenyl)methyl]-1,3,4-thiadiazol-2-yl]carbamoyl]-2-hydroxy-2-methyl-propyl]carbamate (**41**):

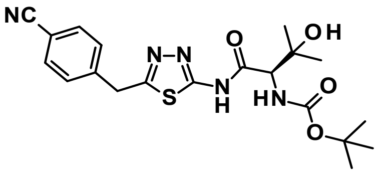

N-(tert-butoxycarbonyl)-3-hydroxy-D-valine (104 mg, 444 µmol, 1.2 eq.) and HATU (211 mg, 555 µmol, 1.5 eq.) were dissolved in DMF (0.87 mL) and DIPEA (95.6 mg, 129 µL, 740 µmol, 2.0 eq.) was added. After stirring for 15 min at room temperature, 4-[(5-amino-1,3,4-thiadiazol-2-yl)methyl]benzonitrile (compound **25**, 80 mg, 370 µmol, 1.0 eq.) was added and the reaction mixture was stirred at room temperature for 3 days. A few drops of water were added, and the reaction mixture was purified using preparative HPLC (water/acetonitrile gradient) to yield the desired compound **41** (76.7 mg, 100% purity, 48 % yield). **LC-MS** (Method A): R_t = 1.56 min; **HRMS:** m/z $[M+H]^+$ calcd for $C_{20}H_{26}N_5O_4S$: 432.1705, found 432.1700. **^{1}H NMR** (600 MHz, DMSO-d_6) δ ppm 1.16 (s, 3 H), 1.19 (s, 3 H), 1.41 (s, 9 H), 4.24 (br d, *J*=8.22 Hz, 1 H), 4.51 (s, 2

H), 4.86 - 4.96 (m, 1 H), 6.84 (br d, *J*=8.41 Hz, 1 H), 7.58 (d, *J*=8.02 Hz, 2 H), 7.85 (d, *J*=8.02 Hz, 2 H), 12.26 - 12.38 (m, 1 H).

N-[(1R)-1-[[5-[(4-cyanophenyl)methyl]-1,3,4-thiadiazol-2-yl]carbamoyl]-2-hydroxy-2-methyl-propyl]-1-(trifluoromethyl)cyclohexanecarboxamide (**19**):

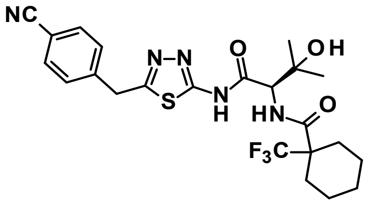

Step 1:

tert-butyl N-[(1R)-1-[[5-[(4-cyanophenyl)methyl]-1,3,4-thiadiazol-2-yl]carbamoyl]-2-hydroxy-2-methyl-propyl]carbamate (compound **41**, 100 % purity, 75.0 mg, 174 µmol, 1.0 eq.) was dissolved in DCM (1.32 mL) and 4 N HCl in dioxane (435 µL, 1.74 mmol, 10.0 eq.) was added. After stirring for 3 h at room temperature, the reaction mixture was concentrated and dried *in vacuo* to yield (2R)-2-amino-N-[5-[(4-cyanophenyl)methyl]-1,3,4-thiadiazol-2-yl]-3-hydroxy-3-methyl-butan-amide dihydrochloride (69.1 mg, 100 % purity, 98 % yield). **LC-MS** (Method A): R_t = 0.71 min; **HRMS:** m/z $[M+H-2HCl]^+$ calcd for $C_{15}H_{18}N_5O_2S$: 332.1181, found 332.1176.

Step 2:

1-(Trifluoromethyl)cyclohexanecarboxylic acid (49.9 mg, 254 µmol, 1.5 eq.), EDC·HCl (48.7 mg, 254 µmol, 1.5 eq.) and HOBt Hydrate (38.9 mg, 254 µmol, 1.5 eq.) were dissolved in DMF (0.76 mL). DIPEA (109 mg, 148 µL, 847 µmol, 5.0 eq.) and (2R)-2-amino-N-[5-[(4-cyanophenyl)methyl]-1,3,4-thiadiazol-2-yl]-3-hydroxy-3-methyl-butanamide dihydrochloride (68.5 mg, 169 µmol, 1.0 eq.) were added and the reaction mixture was stirred overnight at room temperature. A few drops of water were added and the reaction mixture was purified using

preparative HPLC (water/acetonitrile gradient) to yield the desired compound **19** (57.3 mg, 100 % purity, 66 % yield). **LC-MS** (Method A): R_t = 1.84 min; **HRMS:** m/z $[M+H]^+$ calcd for $C_{23}H_{27}F_3N_5O_3S$: 510.1786, found 510.1781. **^{1}H NMR** (600 MHz, DMSO-d_6) δ ppm 1.13 - 1.21 (m, 8 H), 1.24 - 1.32 (m, 1 H), 1.40 - 1.58 (m, 3 H), 1.64 (m, 2 H), 2.29 - 2.41 (m, 2 H), 4.48 (s, 2 H), 4.62 (d, *J*=8.02 Hz, 1 H), 5.19 (s, 1 H), 7.56 (d, *J*=8.22 Hz, 3 H), 7.82 (d, *J*=8.22 Hz, 2 H), 12.51 (s, 1 H). **^{13}C NMR** (126 MHz, DMSO-d_6) δ ppm 21.44, 21.6, 24.2, 26.7, 26.8, 26.95, 26.97, 34.6, 51.6, 51.8, 61.3, 70.3, 109.8, 118.6, 125.2, 127.5, 129.9, 132.5, 143.3, 158.3, 162.1, 166.2, 168.3. **$[\alpha]_D^{20}$** = +46.41 (c = 0.260 in MeOH).

Synthesis of compound 20

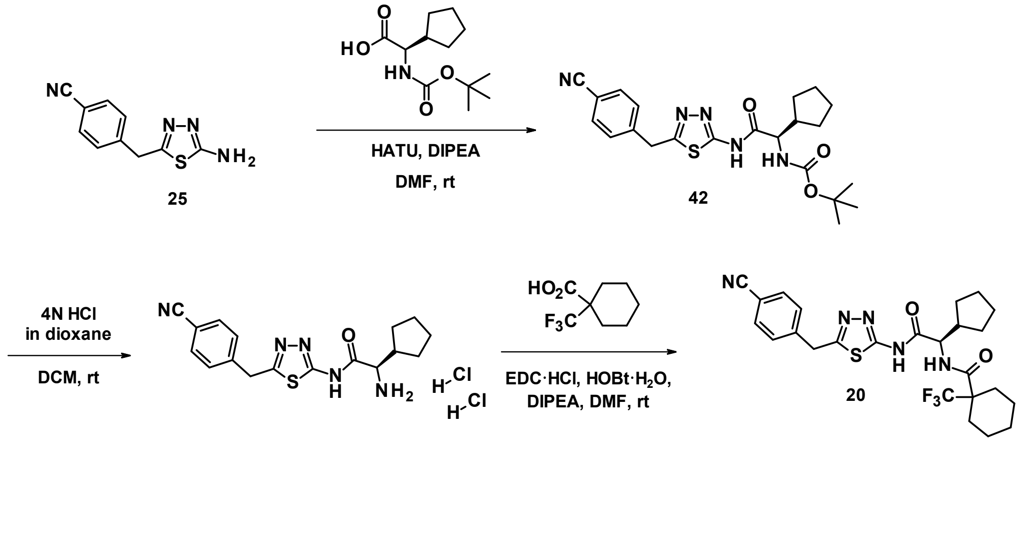

tert-butyl N-[(1R)-2-[[5-[(4-cyanophenyl)methyl]-1,3,4-thiadiazol-2-yl]amino]-1-cyclopentyl-2-oxo-ethyl]carbamate (**42**):

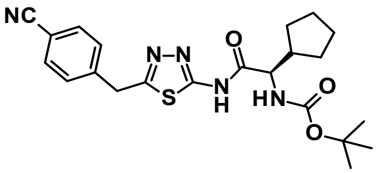

(2R)-2-(tert-butoxycarbonylamino)-2-cyclopentyl-acetic acid (135 mg, 554 µmol, 1.2 eq.) and HATU (211 mg, 554 µmol, 1.2 eq.) were dissolved in DMF (1.1 mL) and DIPEA (120 mg, 161 µL, 925 µmol, 2.0 eq.) was added. After stirring for 15 min at room temperature, 4-[(5-amino-1,3,4-thiadiazol-2-yl)methyl]benzonitrile (compound **25**, 100 mg, 462 µmol, 1.0 eq.) was added and the reaction mixture was stirred at room temperature overnight. A few drops of water were added, and the reaction mixture was purified using preparative HPLC (water/acetonitrile gradient) to yield the desired compound **42** (166 mg, 100% purity, 81 % yield). **LC-MS** (Method A): R_t = 1.93 min; **HRMS:** m/z $[M+H]^+$ calcd for $C_{22}H_{28}N_5O_3S$: 442.1912, found 4442.1908. **^{1}H NMR** (600 MHz, DMSO-d_6) δ ppm 1.15 - 1.24 (m, 2 H), 1.36 (s, 9 H), 1.41 - 1.49 (m, 3 H), 1.51 - 1.63 (m, 2 H), 1.73 (m, 1 H), 1.95 - 2.15 (m, 1 H), 4.00 - 4.07 (m, 1 H), 4.48 (s, 2 H), 7.16 - 7.26 (m, 1 H), 7.55 (d, *J*=8.25 Hz, 2 H), 7.82 (d, *J*=8.07 Hz, 2 H), 12.56 (s, 1 H).

N-[(1R)-2-[[5-[(4-cyanophenyl)methyl]-1,3,4-thiadiazol-2-yl]amino]-1-cyclopentyl-2-oxo-ethyl]-1-(trifluoromethyl)cyclohexanecarboxamide (**20**):

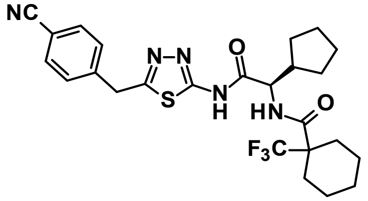

Step 1:

tert-butyl N-[(1R)-2-[[5-[(4-cyanophenyl)methyl]-1,3,4-thiadiazol-2-yl]amino]-1-cyclopentyl-2-oxo-ethyl]carbamate (compound **42**, 232 mg, 525 µmol, 1.0 eq.) was dissolved in DCM (4.36 mL) and 4 N HCl in dioxane (1.31 mL, 5.25 mmol, 10.0 eq.) was added. After stirring for 5 h at room temperature, the reaction mixture was concentrated and dried *in vacuo* to yield (2R)-2-amino-N-[5-[(4-cyanophenyl)methyl]-1,3,4-thiadiazol-2-yl]-2-cyclopentyl-acetamide dihydrochloride (213 mg, 95 % purity, 93 % yield). **LC-MS** (Method B): R_t = 0.93 min; **HRMS:** m/z $[M+H-2HCl]^+$ calcd for $C_{17}H_{20}N_5OS$: 342.1388, found 342.1383.

Step 2:

1-(Trifluoromethyl)cyclohexanecarboxylic acid (142 mg, 722 µmol, 1.5 eq.), EDC·HCl (138 mg, 722 µmol, 1.5 eq.) and HOBt Hydrate (111 mg, 722 µmol, 1.5 eq.) were dissolved in DMF (2.4 mL). DIPEA (311 mg, 419 µL, 2.41 mmol, 5.0 eq.) and (2R)-2-amino-N-[5-[(4-cyanophenyl) methyl]-1,3,4-thiadiazol-2-yl]-2-cyclopentyl-acetamide dihydrochloride (210 mg, 95% purity, 481 µmol, 1.0 eq.) were added and the reaction mixture was stirred overnight at room temperature. The crude mixture was purified using preparative HPLC (water/acetonitrile gradient) to yield the desired compound **20** (146 mg, 100 % purity, 58 % yield). **LC-MS** (Method B): R_t = 2.23 min; **HRMS:** m/z $[M+H]^+$ calcd for $C_{25}H_{29}F_3N_5O_2S$: 520.1994, found 520.1989. **^{1}H NMR** (500 MHz, DMSO-d_6) δ ppm 1.10 - 1.22 (m, 4 H), 1.35 - 1.64 (m, 11 H), 1.79 - 1.88 (m, 1 H), 2.25 - 2.33 (m, 1 H), 2.35 - 2.41 (m, 1 H), 2.41 - 2.48 (m, 1 H), 4.37 - 4.43 (m, 1 H), 4.48 (s, 2 H), 7.54 - 7.59 (m, 2 H), 7.79 - 7.86 (m, 2 H), 8.13 (d, *J*=7.32 Hz, 1 H), 12.67 (s, 1 H). **^{13}C NMR** (126 MHz, DMSO-d_6) δ ppm 21.6, 21.7, 24.1, 24.3, 24.7, 26.5, 26.6, 28.4, 29.9, 34.6, 40.8, 51.5, 51.6, 58.1, 109.8, 118.7, 125.3, 127.5, 129.9, 132.6, 143.3, 158.4, 162.2, 166.3, 170.79. **$[\alpha]_D^{20}$** = +38.28 (c = 0.256 in MeOH)

NMR Spectra

^{1}H-NMR of compound **1**:

Frequency (MHz)	500.1331	Nucleus	1H
Number of Transients	32	Origin	Bruker BioSpin GmbH
Original Points Count	16384	Owner	x
Points Count	65536	Pulse Sequence	zg30
SW(cyclical) (Hz)	9999.85	Solvent	DMSO-d6
Spectrum Offset (Hz)	3086.8579	Spectrum Type	standard
Sweep Width (Hz)	9999.70	Temperature (degree C)	25.000

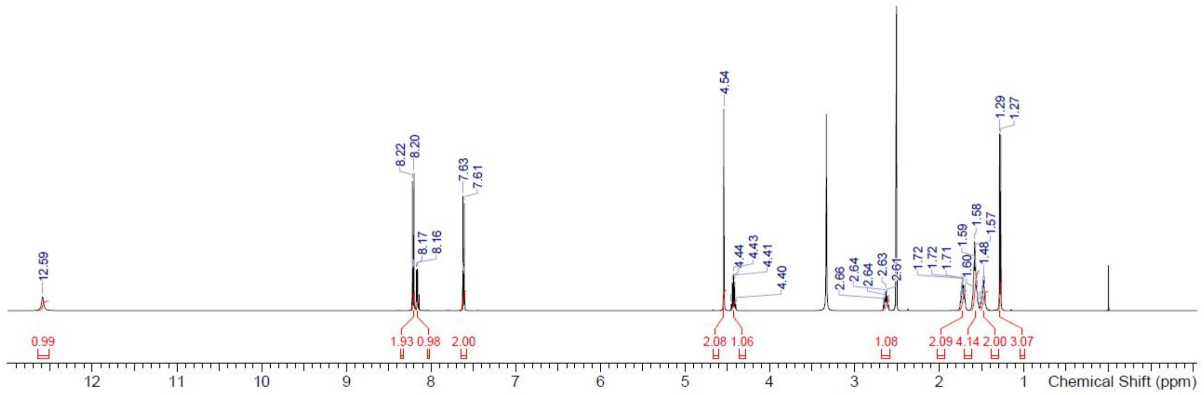

^{13}C-NMR of compound **1**:

Frequency (MHz)	125.7704	Nucleus	13C
Number of Transients	384	Origin	Bruker BioSpin GmbH
Original Points Count	32768	Owner	x
Points Count	65536	Pulse Sequence	zgpg
SW(cyclical) (Hz)	30120.02	Solvent	DMSO-d6
Spectrum Offset (Hz)	12578.7803	Spectrum Type	standard
Sweep Width (Hz)	30119.56	Temperature (degree C)	25.000

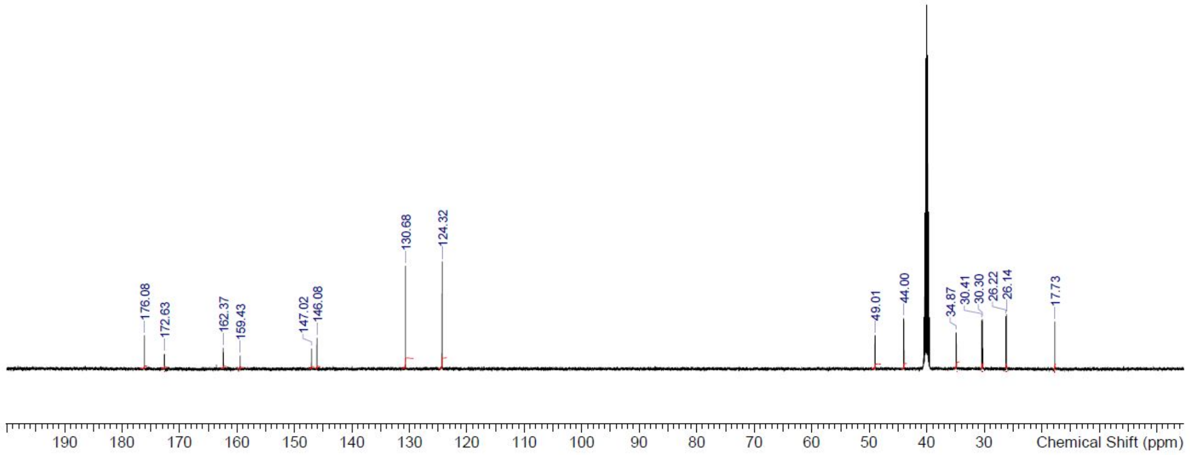

^{1}H-NMR of compound **2**:

Frequency (MHz)	500.1331	Nucleus	1H
Number of Transients	32	Origin	Bruker BioSpin GmbH
Original Points Count	16384	Owner	x
Points Count	65536	Pulse Sequence	zg30
SW(cyclical) (Hz)	9999.85	Solvent	DMSO-d6
Spectrum Offset (Hz)	3086.0884	Spectrum Type	standard
Sweep Width (Hz)	9999.70	Temperature (degree C)	24.995

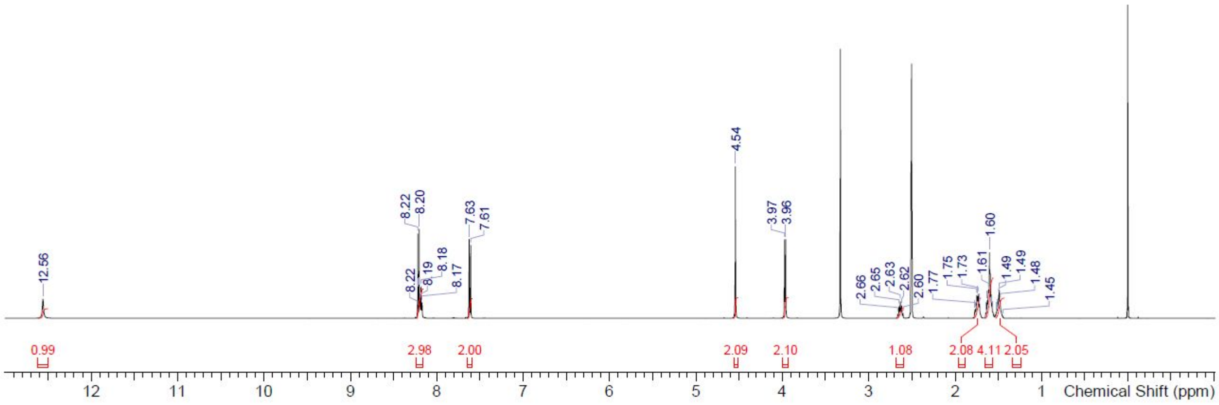

^{13}C-NMR of compound **2**:

Frequency (MHz)	125.7704	Nucleus	13C
Number of Transients	640	Origin	Bruker BioSpin GmbH
Original Points Count	32768	Owner	x
Points Count	65536	Pulse Sequence	zgpg
SW(cyclical) (Hz)	30120.02	Solvent	DMSO-d6
Spectrum Offset (Hz)	12506.0361	Spectrum Type	standard
Sweep Width (Hz)	30119.56	Temperature (degree C)	25.000

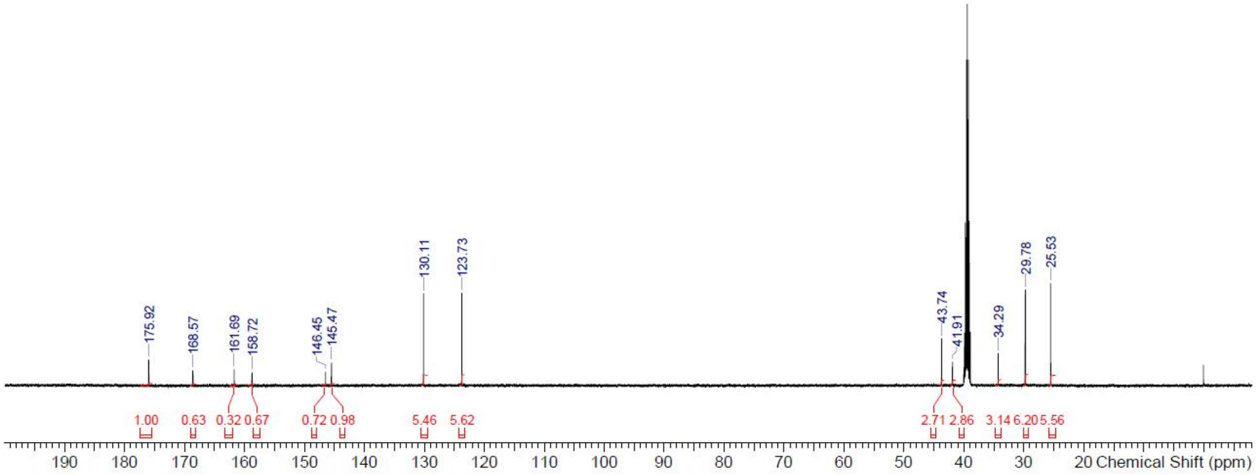

<u>^{1}H-NMR of compound **3**:</u>

Frequency (MHz)	400.1425	Nucleus	1H
Number of Transients	16	Origin	Bruker BioSpin GmbH
Original Points Count	32768	Owner	x
Points Count	65536	Pulse Sequence	zg30
SW(cyclical) (Hz)	8620.56	Solvent	DMSO-d6
Spectrum Offset (Hz)	2470.4507	Spectrum Type	standard
Sweep Width (Hz)	8620.43	Temperature (degree C)	24.998

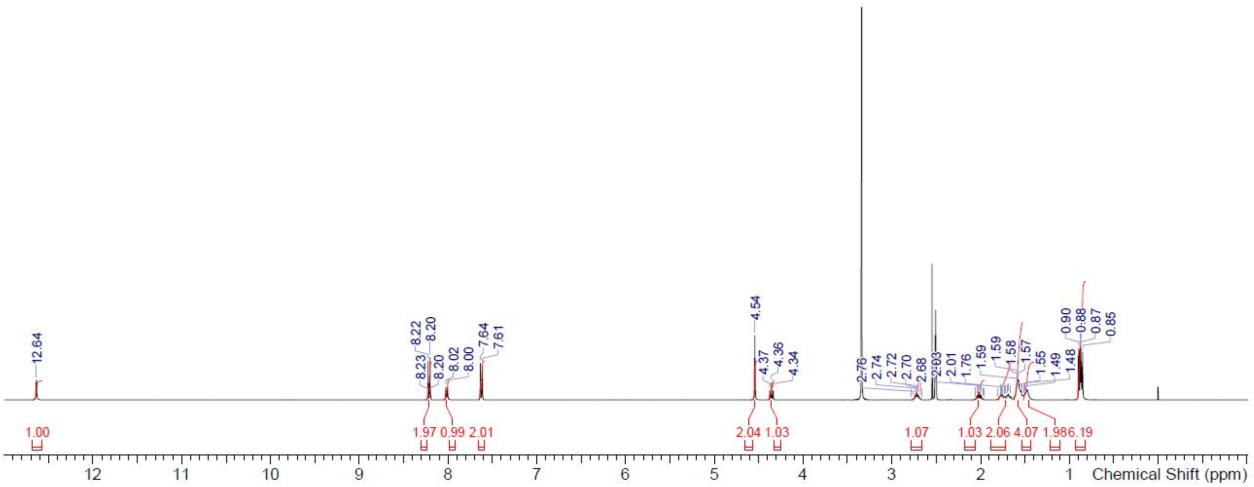

<u>^{13}C-NMR of compound **3**:</u>

Frequency (MHz)	125.7704	Nucleus	13C
Number of Transients	256	Origin	Bruker BioSpin GmbH
Original Points Count	32768	Owner	x
Points Count	65536	Pulse Sequence	zgpg
SW(cyclical) (Hz)	30120.02	Solvent	DMSO-d6
Spectrum Offset (Hz)	12507.7979	Spectrum Type	standard
Sweep Width (Hz)	30119.56	Temperature (degree C)	24.999

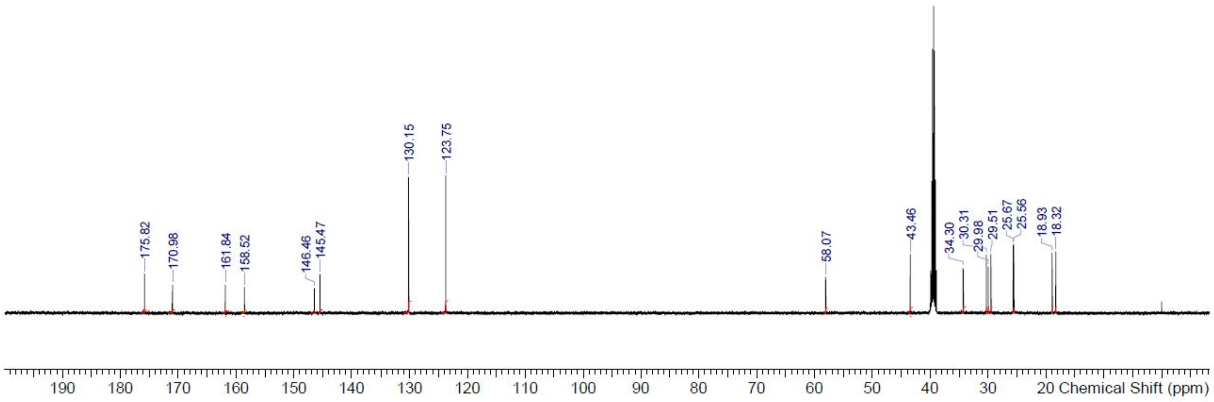

^{1}H-NMR of compound **4**:

Frequency (MHz)	500.1331	*Nucleus*	1H
Number of Transients	32	*Origin*	Bruker BioSpin GmbH
Original Points Count	16384	*Owner*	x
Points Count	65536	*Pulse Sequence*	zg30
SW(cyclical) (Hz)	9999.85	*Solvent*	DMSO-d6
Spectrum Offset (Hz)	3087.5981	*Spectrum Type*	standard
Sweep Width (Hz)	9999.70	*Temperature (degree C)*	24.989

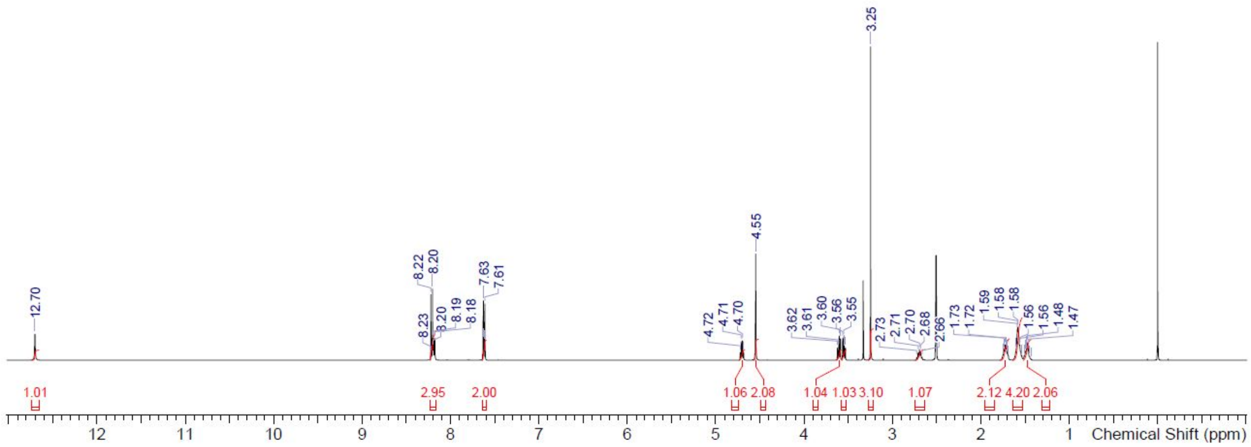

^{13}C-NMR of compound **4**:

Frequency (MHz)	125.7704	*Nucleus*	13C
Number of Transients	256	*Origin*	Bruker BioSpin GmbH
Original Points Count	32768	*Owner*	x
Points Count	65536	*Pulse Sequence*	zgpg
SW(cyclical) (Hz)	30120.02	*Solvent*	DMSO-d6
Spectrum Offset (Hz)	12507.3447	*Spectrum Type*	standard
Sweep Width (Hz)	30119.56	*Temperature (degree C)*	25.003

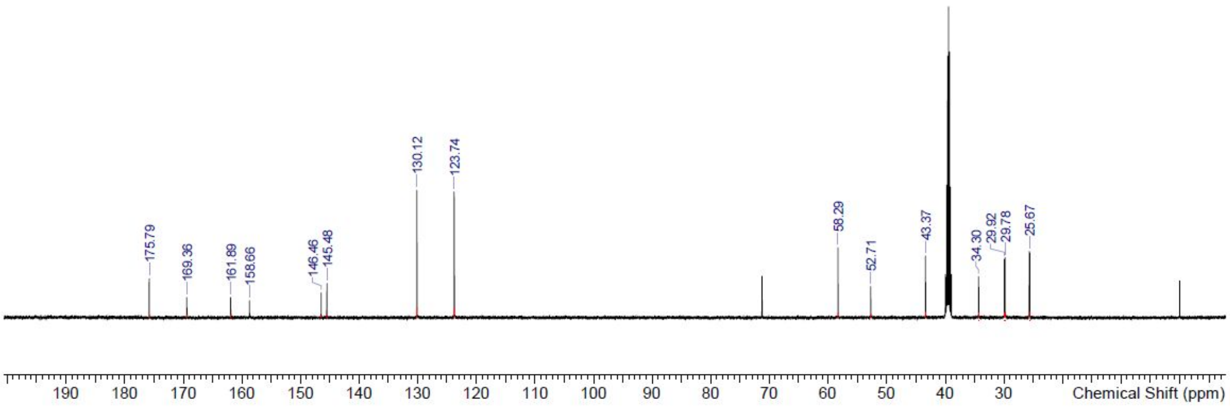

^{1}H-NMR of compound **5**:

Frequency (MHz)	500.1331	*Nucleus*	1H
Number of Transients	32	*Origin*	Bruker BioSpin GmbH
Original Points Count	16384	*Owner*	x
Points Count	65536	*Pulse Sequence*	zg30
SW(cyclical) (Hz)	9999.85	*Solvent*	DMSO-d6
Spectrum Offset (Hz)	3087.4180	*Spectrum Type*	standard
Sweep Width (Hz)	9999.70	*Temperature (degree C)*	24.999

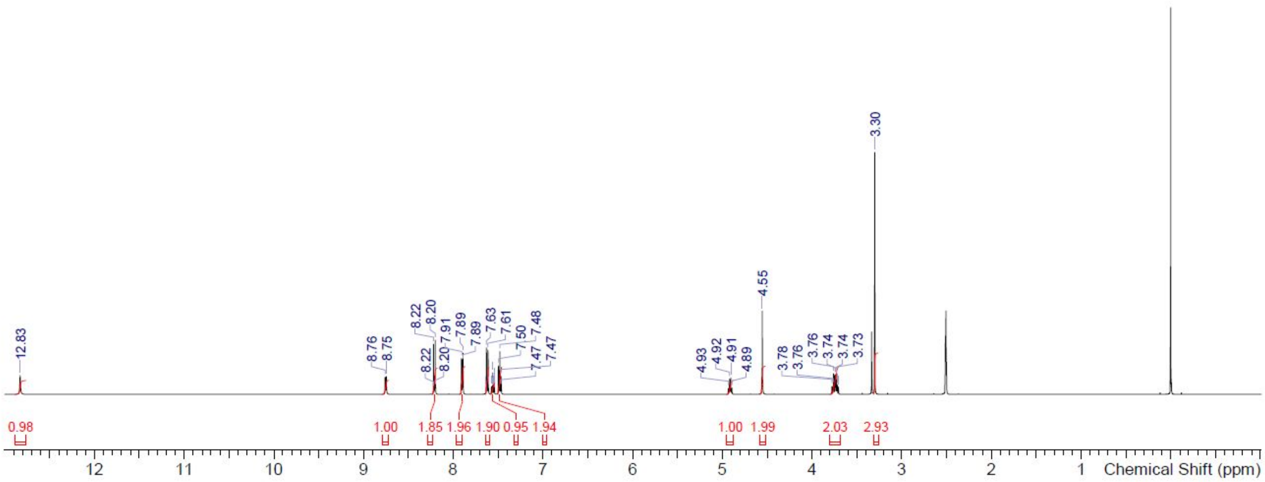

^{13}C-NMR of compound **5**:

Frequency (MHz)	125.7704	*Nucleus*	13C
Number of Transients	256	*Origin*	Bruker BioSpin GmbH
Original Points Count	32768	*Owner*	x
Points Count	65536	*Pulse Sequence*	zgpg
SW(cyclical) (Hz)	30120.02	*Solvent*	DMSO-d6
Spectrum Offset (Hz)	12506.9170	*Spectrum Type*	standard
Sweep Width (Hz)	30119.56	*Temperature (degree C)*	25.036

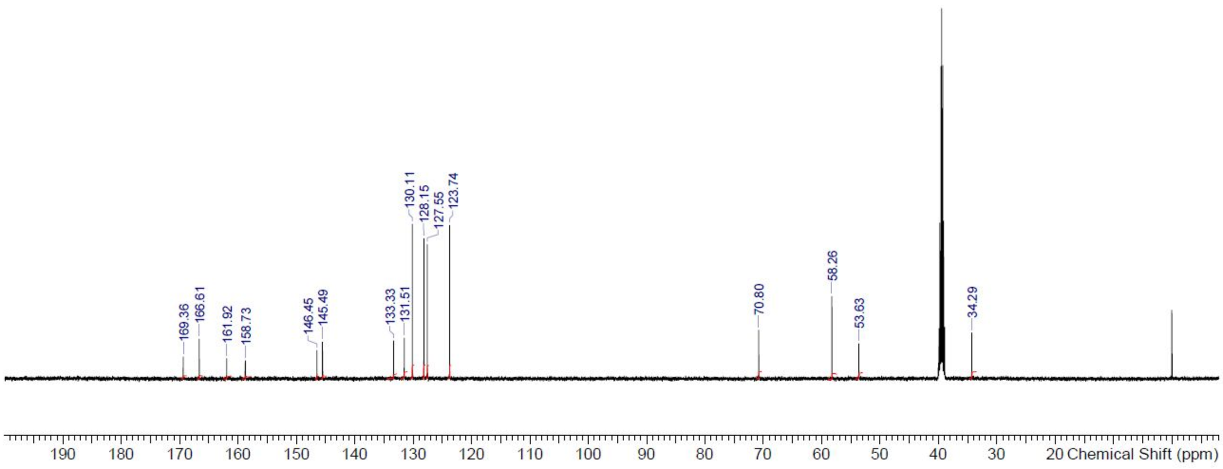

^{1}H-NMR of compound **6**:

Frequency (MHz)	600.2845	*Nucleus*	1H
Number of Transients	16	*Origin*	Bruker BioSpin GmbH
Original Points Count	32768	*Owner*	x
Points Count	65536	*Pulse Sequence*	zg30
SW(cyclical) (Hz)	12820.32	*Solvent*	DMSO-d6
Spectrum Offset (Hz)	4481.1392	*Spectrum Type*	standard
Sweep Width (Hz)	12820.12	*Temperature (degree C)*	25.320

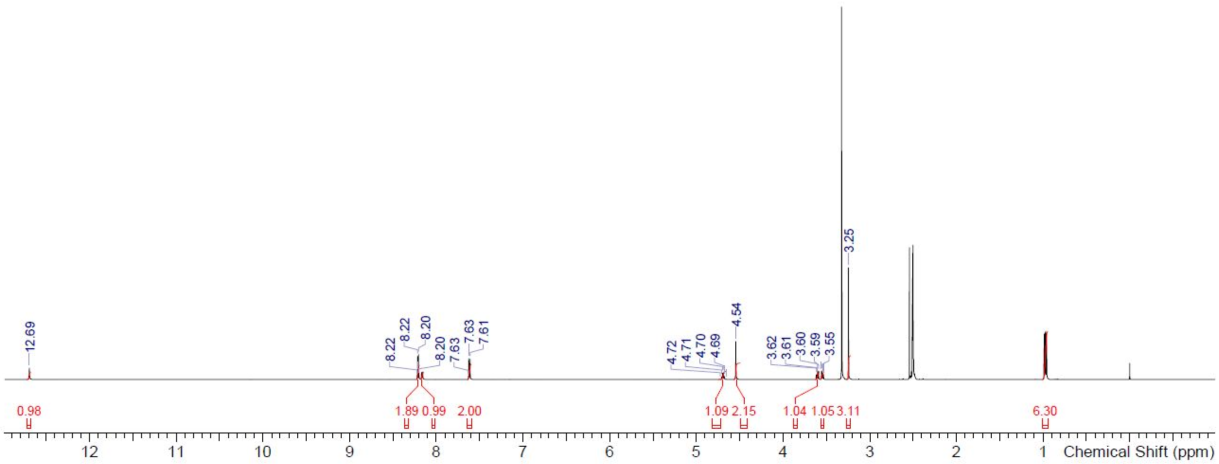

^{13}C-NMR of compound **6**:

Frequency (MHz)	125.7704	*Nucleus*	13C
Number of Transients	384	*Origin*	Bruker BioSpin GmbH
Original Points Count	32768	*Owner*	x
Points Count	65536	*Pulse Sequence*	zgpg
SW(cyclical) (Hz)	30120.02	*Solvent*	DMSO-d6
Spectrum Offset (Hz)	12506.5771	*Spectrum Type*	standard
Sweep Width (Hz)	30119.56	*Temperature (degree C)*	24.996

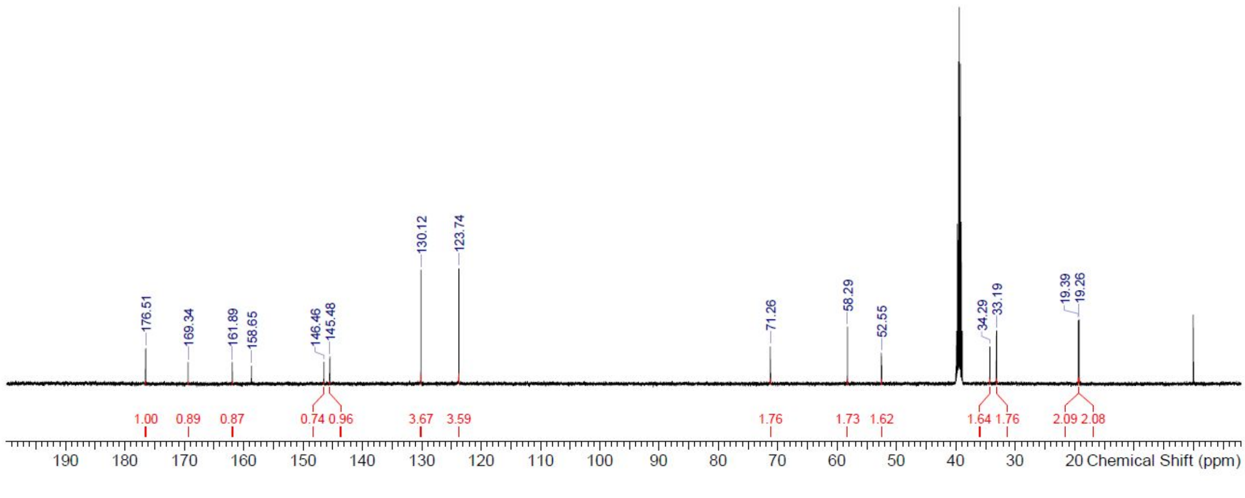

^{1}H-NMR of compound **7**:

Frequency (MHz)	500.1331	*Nucleus*	1H
Number of Transients	32	*Origin*	Bruker BioSpin GmbH
Original Points Count	16384	*Owner*	x
Points Count	65536	*Pulse Sequence*	zg30
SW(cyclical) (Hz)	9999.85	*Solvent*	DMSO-d6
Spectrum Offset (Hz)	3086.6528	*Spectrum Type*	standard
Sweep Width (Hz)	9999.70	*Temperature (degree C)*	24.997

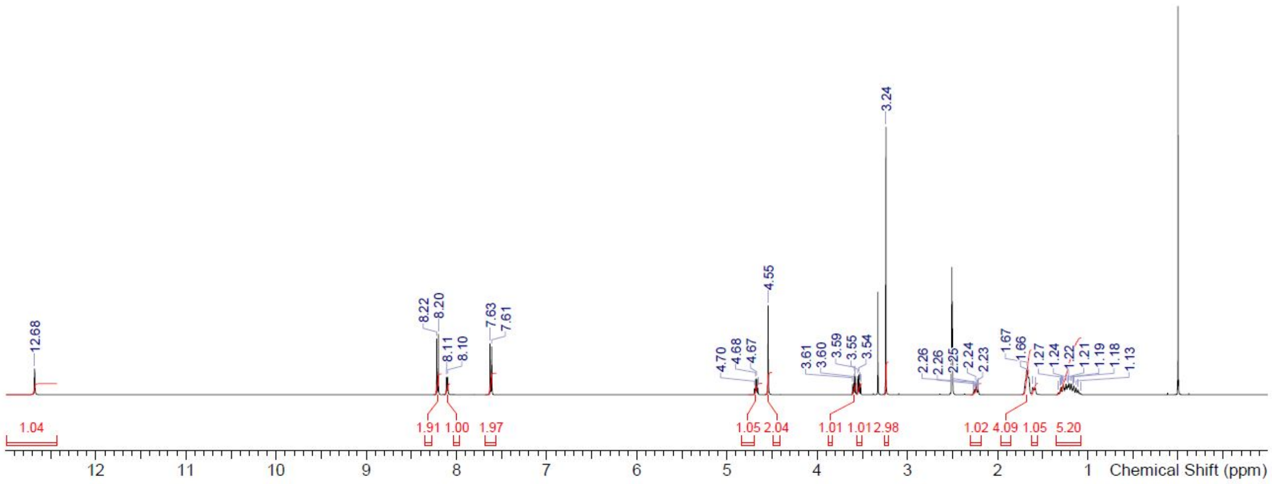

^{13}C-NMR of compound **7**:

Frequency (MHz)	125.7704	*Nucleus*	13C
Number of Transients	384	*Origin*	Bruker BioSpin GmbH
Original Points Count	32768	*Owner*	x
Points Count	65536	*Pulse Sequence*	zgpg
SW(cyclical) (Hz)	30120.02	*Solvent*	DMSO-d6
Spectrum Offset (Hz)	12506.7646	*Spectrum Type*	standard
Sweep Width (Hz)	30119.56	*Temperature (degree C)*	24.987

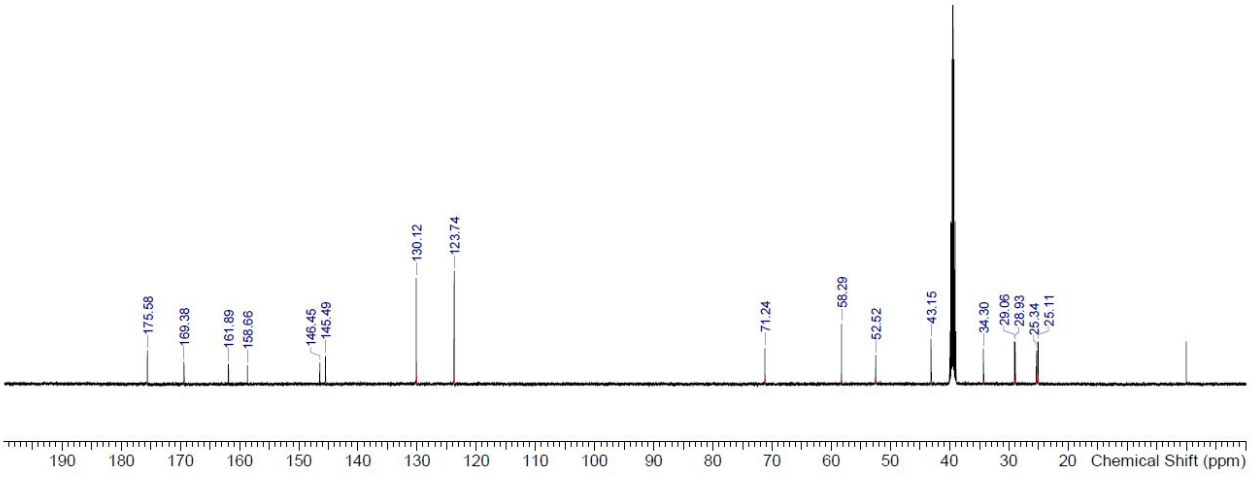

^{1}H-NMR of compound **8**:

Frequency (MHz)	600.2845	*Nucleus*	1H
Number of Transients	16	*Origin*	Bruker BioSpin GmbH
Original Points Count	32768	*Owner*	x
Points Count	65536	*Pulse Sequence*	zg30
SW(cyclical) (Hz)	12820.32	*Solvent*	DMSO-d6
Spectrum Offset (Hz)	4481.1021	*Spectrum Type*	standard
Sweep Width (Hz)	12820.12	*Temperature (degree C)*	25.319

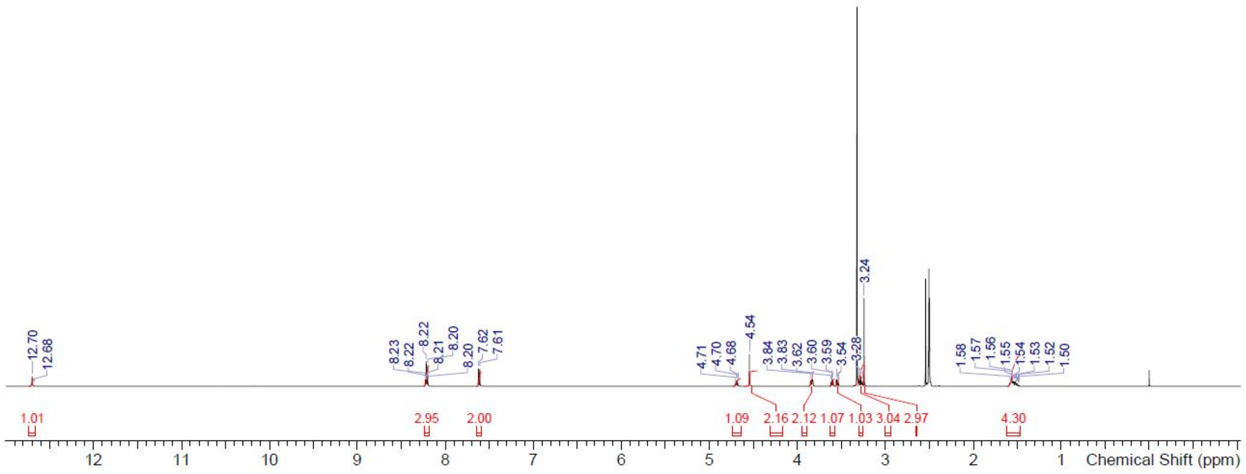

^{13}C-NMR of compound **8**:

Frequency (MHz)	125.7704	*Nucleus*	13C
Number of Transients	384	*Origin*	Bruker BioSpin GmbH
Original Points Count	32768	*Owner*	x
Points Count	65536	*Pulse Sequence*	zgpg
SW(cyclical) (Hz)	30120.02	*Solvent*	DMSO-d6
Spectrum Offset (Hz)	12506.3506	*Spectrum Type*	standard
Sweep Width (Hz)	30119.56	*Temperature (degree C)*	24.993

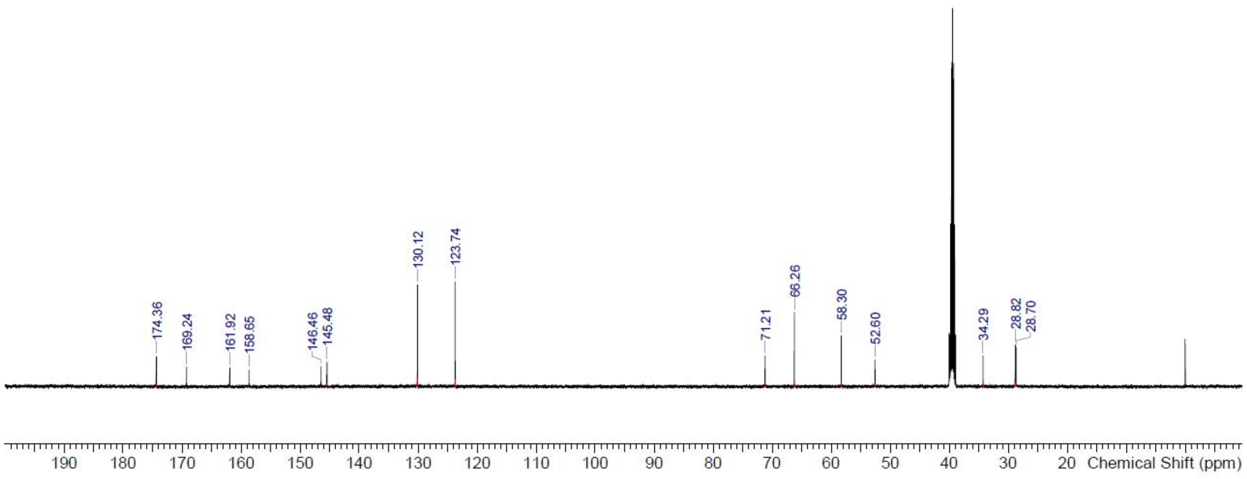

^{1}H-NMR of compound **9**:

Frequency (MHz)	500.1331	Nucleus	1H
Number of Transients	32	Origin	Bruker BioSpin GmbH
Original Points Count	16384	Owner	x
Points Count	65536	Pulse Sequence	zg30
SW(cyclical) (Hz)	9999.85	Solvent	DMSO-d6
Spectrum Offset (Hz)	3085.1030	Spectrum Type	standard
Sweep Width (Hz)	9999.70	Temperature (degree C)	24.999

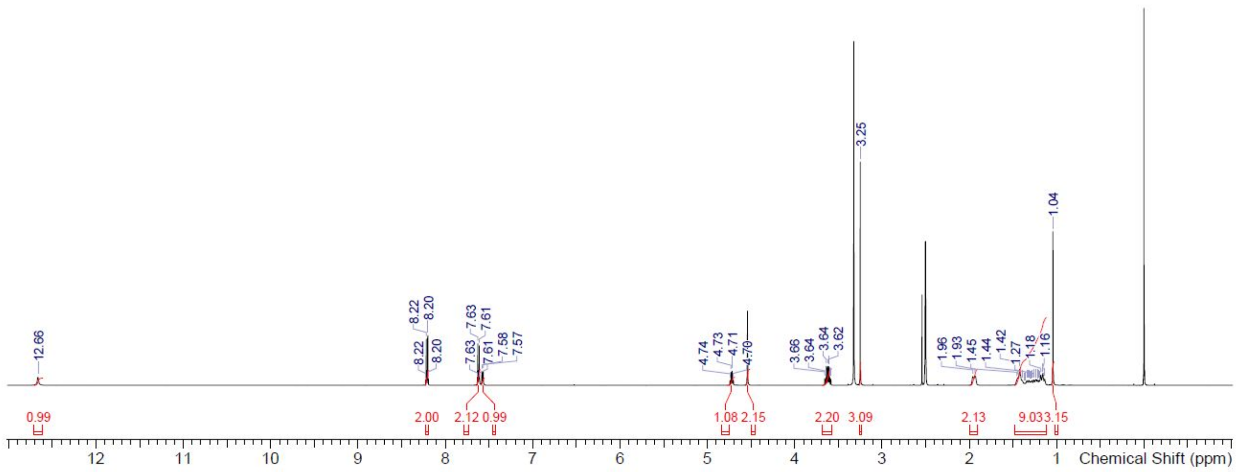

^{13}C-NMR of compound **9**:

Frequency (MHz)	125.7704	Nucleus	13C
Number of Transients	256	Origin	Bruker BioSpin GmbH
Original Points Count	32768	Owner	x
Points Count	65536	Pulse Sequence	zgpg
SW(cyclical) (Hz)	30120.02	Solvent	DMSO-d6
Spectrum Offset (Hz)	12578.7803	Spectrum Type	standard
Sweep Width (Hz)	30119.56	Temperature (degree C)	25.000

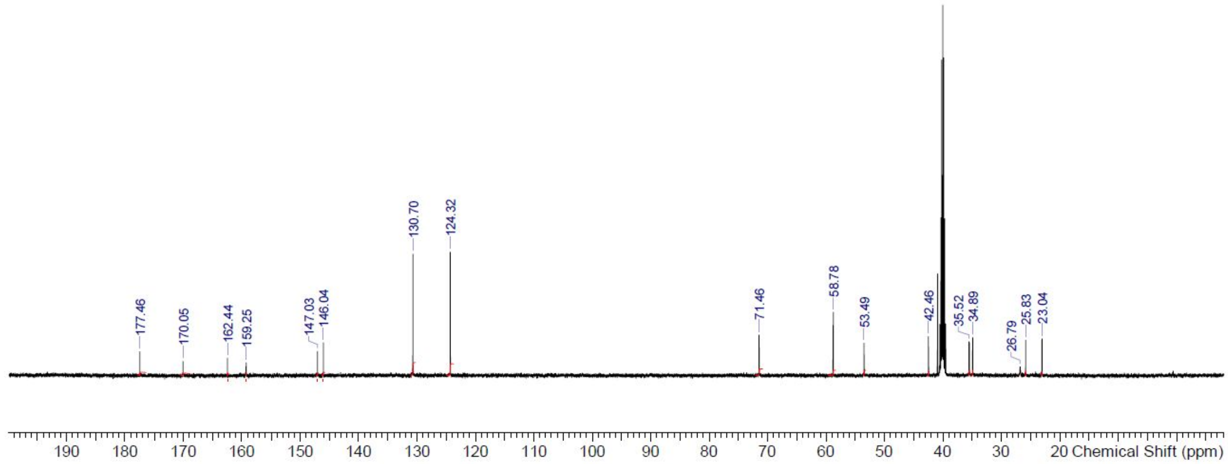

^{1}H-NMR of compound **10**:

Frequency (MHz)	600.2845	Nucleus	1H
Number of Transients	16	Origin	Bruker BioSpin GmbH
Original Points Count	32768	Owner	x
Points Count	65536	Pulse Sequence	zg30
SW(cyclical) (Hz)	12820.32	Solvent	DMSO-d6
Spectrum Offset (Hz)	4482.6753	Spectrum Type	standard
Sweep Width (Hz)	12820.12	Temperature (degree C)	25.001

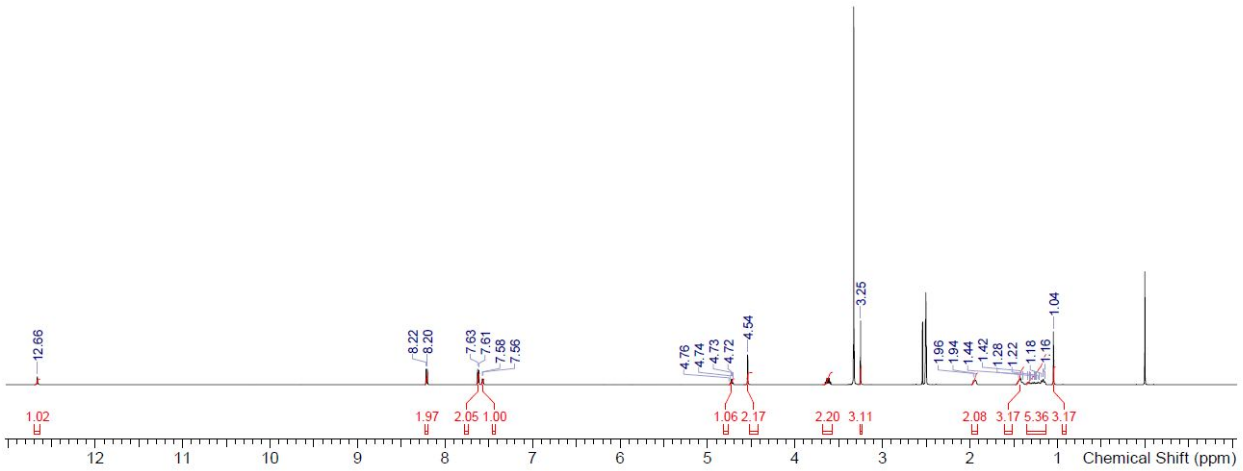

^{13}C-NMR of compound **10**:

Frequency (MHz)	125.7704	Nucleus	13C
Number of Transients	128	Origin	Bruker BioSpin GmbH
Original Points Count	32768	Owner	x
Points Count	65536	Pulse Sequence	zgpg
SW(cyclical) (Hz)	30120.02	Solvent	DMSO-d6
Spectrum Offset (Hz)	12509.3428	Spectrum Type	standard
Sweep Width (Hz)	30119.56	Temperature (degree C)	25.002

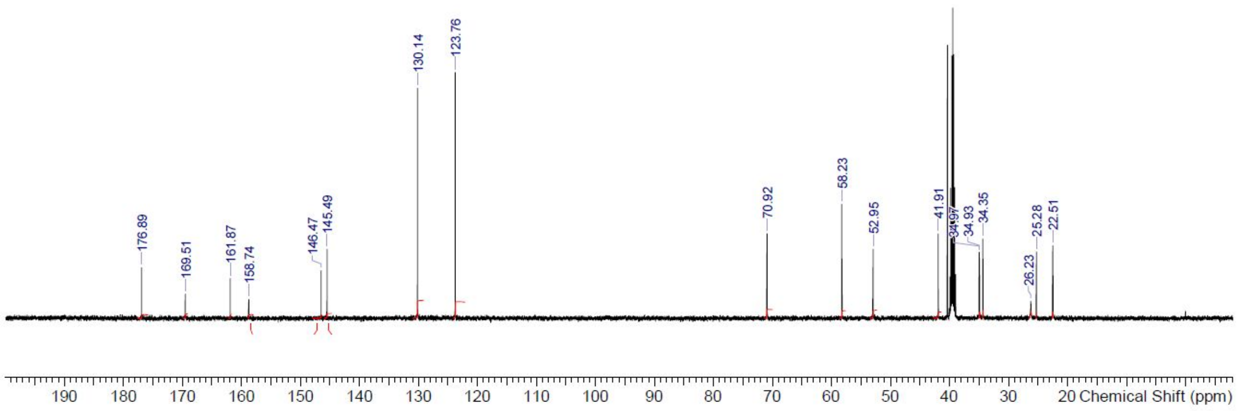

^{1}H-NMR of compound **11**:

Frequency (MHz)	500.1331	Nucleus	1H
Number of Transients	32	Origin	Bruker BioSpin GmbH
Original Points Count	16384	Owner	x
Points Count	65536	Pulse Sequence	zg30
SW(cyclical) (Hz)	9999.85	Solvent	DMSO-d6
Spectrum Offset (Hz)	3085.3022	Spectrum Type	standard
Sweep Width (Hz)	9999.70	Temperature (degree C)	25.002

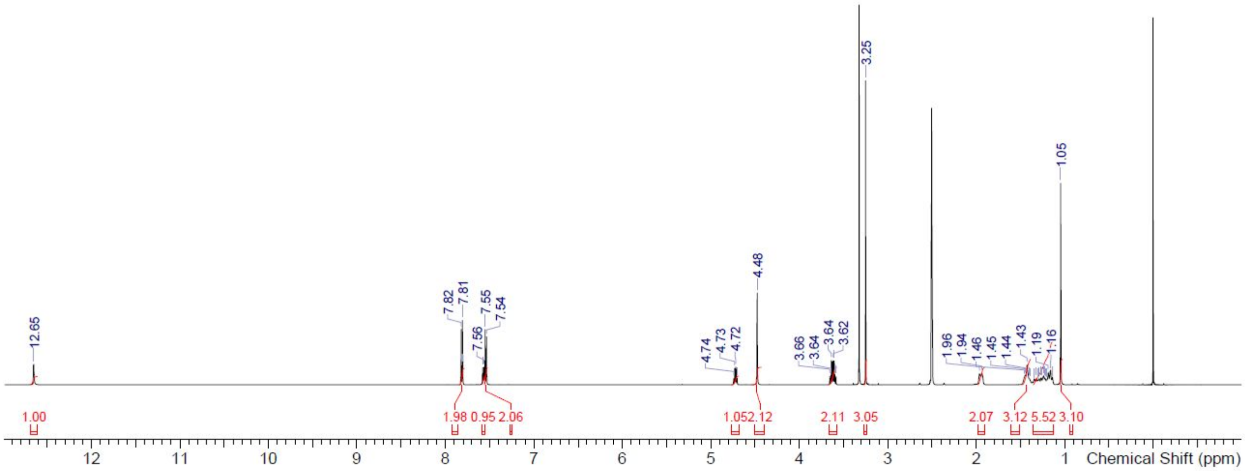

^{13}C-NMR of compound **11**:

Frequency (MHz)	125.7704	Nucleus	13C
Number of Transients	1024	Origin	Bruker BioSpin GmbH
Original Points Count	32768	Owner	x
Points Count	65536	Pulse Sequence	zgpg
SW(cyclical) (Hz)	30120.02	Solvent	DMSO-d6
Spectrum Offset (Hz)	12505.5186	Spectrum Type	standard
Sweep Width (Hz)	30119.56	Temperature (degree C)	25.000

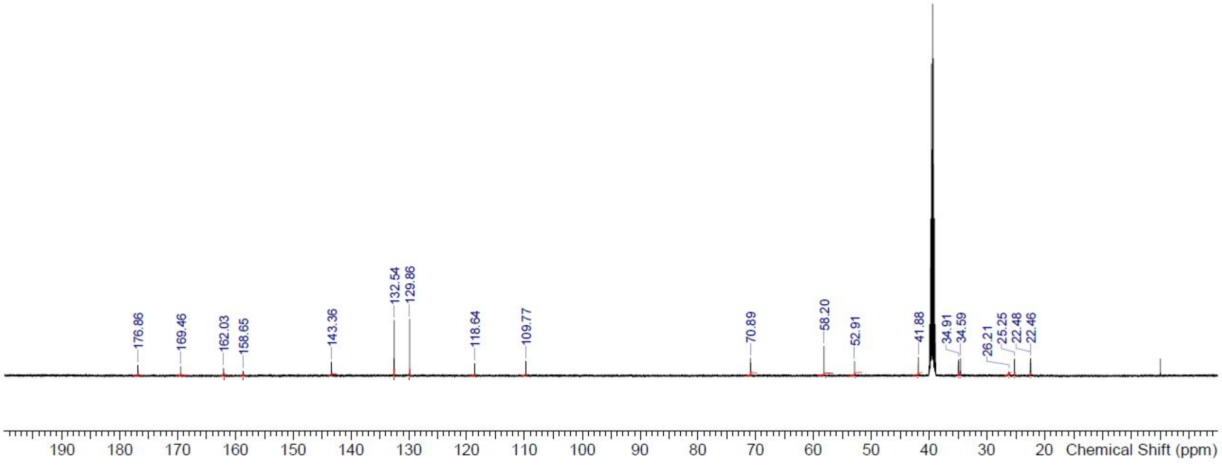

^{1}H-NMR of compound **12**:

Frequency (MHz)	600.2845	Nucleus	1H
Number of Transients	16	Origin	Bruker BioSpin GmbH
Original Points Count	32768	Owner	x
Points Count	65536	Pulse Sequence	zg30
SW(cyclical) (Hz)	12820.32	Solvent	DMSO-d6
Spectrum Offset (Hz)	4481.8589	Spectrum Type	standard
Sweep Width (Hz)	12820.12	Temperature (degree C)	25.001

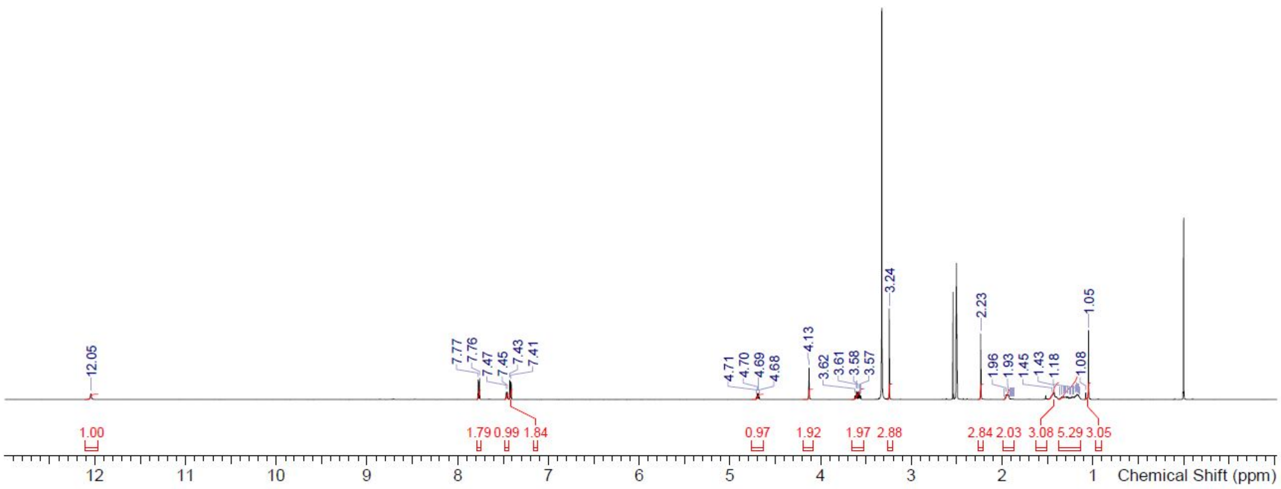

^{1}H-NMR of compound **13**:

Frequency (MHz)	500.1331	Nucleus	1H
Number of Transients	32	Origin	Bruker BioSpin GmbH
Original Points Count	16384	Owner	x
Points Count	65536	Pulse Sequence	zg30
SW(cyclical) (Hz)	9999.85	Solvent	DMSO-d6
Spectrum Offset (Hz)	3085.7783	Spectrum Type	standard
Sweep Width (Hz)	9999.70	Temperature (degree C)	24.998

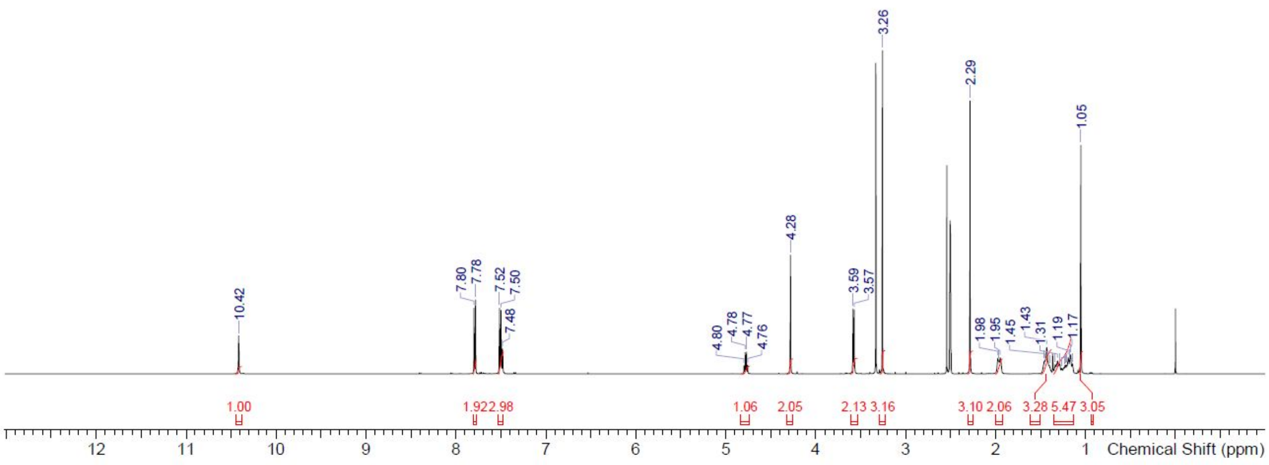

^{13}C-NMR of compound **13**:

Frequency (MHz)	125.7704	Nucleus	13C
Number of Transients	384	Origin	Bruker BioSpin GmbH
Original Points Count	32768	Owner	x
Points Count	65536	Pulse Sequence	zgpg
SW(cyclical) (Hz)	30120.02	Solvent	DMSO-d6
Spectrum Offset (Hz)	12578.7803	Spectrum Type	standard
Sweep Width (Hz)	30119.56	Temperature (degree C)	24.999

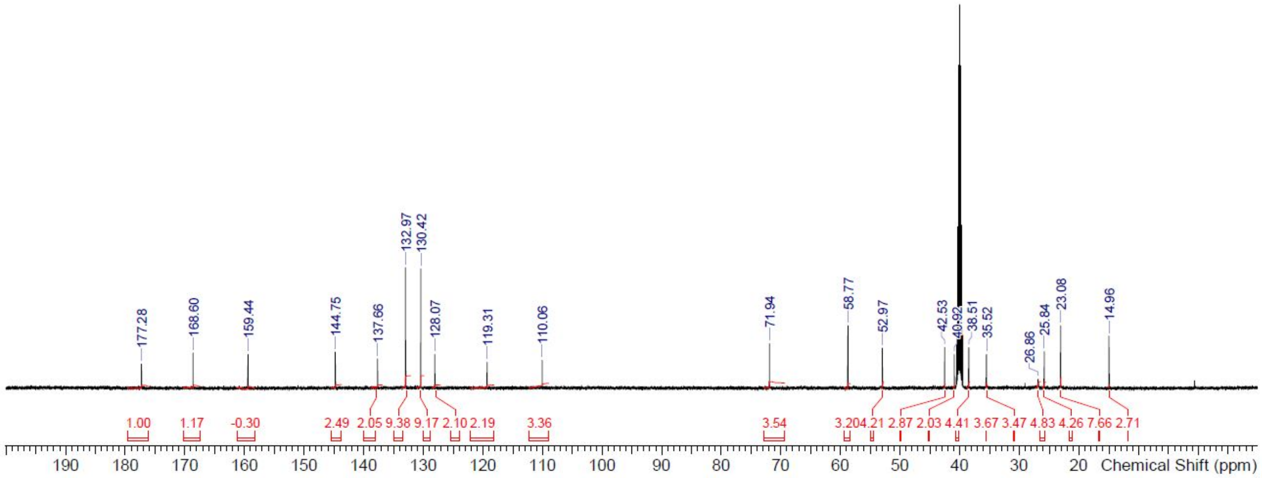

^{1}H-NMR of compound **14**:

Frequency (MHz)	600.2845	Nucleus	1H
Number of Transients	16	Origin	Bruker BioSpin GmbH
Original Points Count	32768	Owner	x
Points Count	65536	Pulse Sequence	zg30
SW(cyclical) (Hz)	12019.05	Solvent	DMSO-d6
Spectrum Offset (Hz)	4481.8218	Spectrum Type	standard
Sweep Width (Hz)	12018.86	Temperature (degree C)	25.000

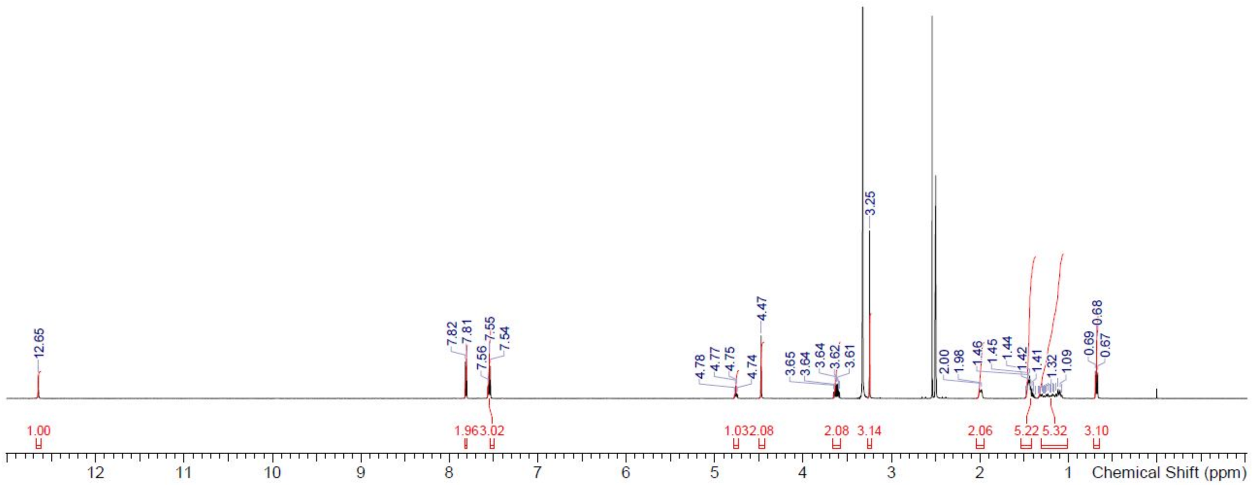

^{13}C-NMR of compound **14**:

Frequency (MHz)	125.7704	*Nucleus*	13C
Number of Transients	256	*Origin*	Bruker BioSpin GmbH
Original Points Count	32768	*Owner*	x
Points Count	65536	*Pulse Sequence*	zgpg
SW(cyclical) (Hz)	30120.02	*Solvent*	DMSO-d6
Spectrum Offset (Hz)	12507.3311	*Spectrum Type*	standard
Sweep Width (Hz)	30119.56	*Temperature (degree C)*	24.997

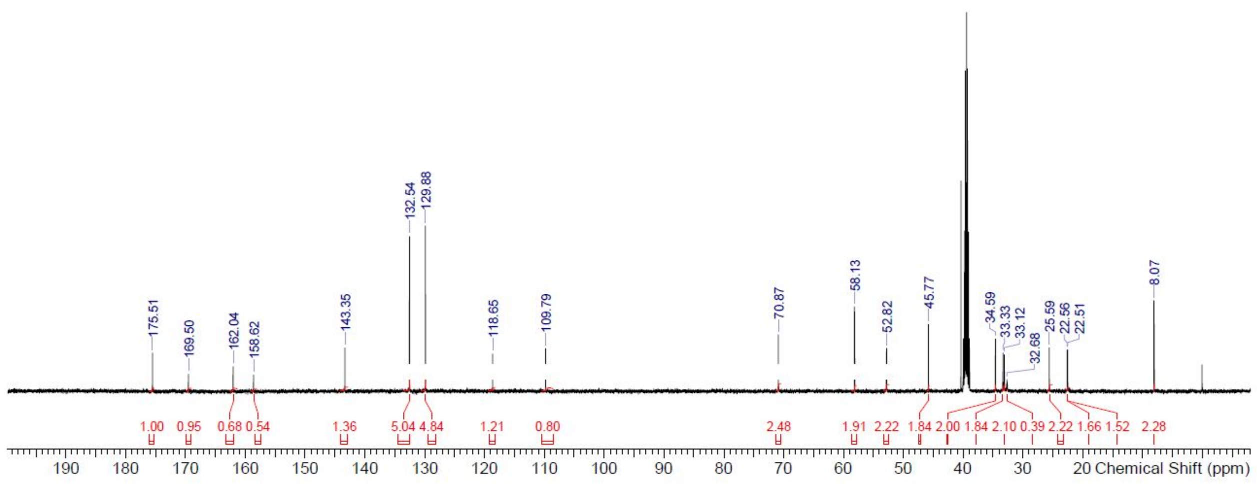

^{1}H-NMR of compound **15**:

Frequency (MHz)	600.2845	*Nucleus*	1H
Number of Transients	16	*Origin*	Bruker BioSpin GmbH
Original Points Count	32768	*Owner*	x
Points Count	65536	*Pulse Sequence*	zg30
SW(cyclical) (Hz)	12019.05	*Solvent*	DMSO-d6
Spectrum Offset (Hz)	4482.1284	*Spectrum Type*	standard
Sweep Width (Hz)	12018.86	*Temperature (degree C)*	25.000

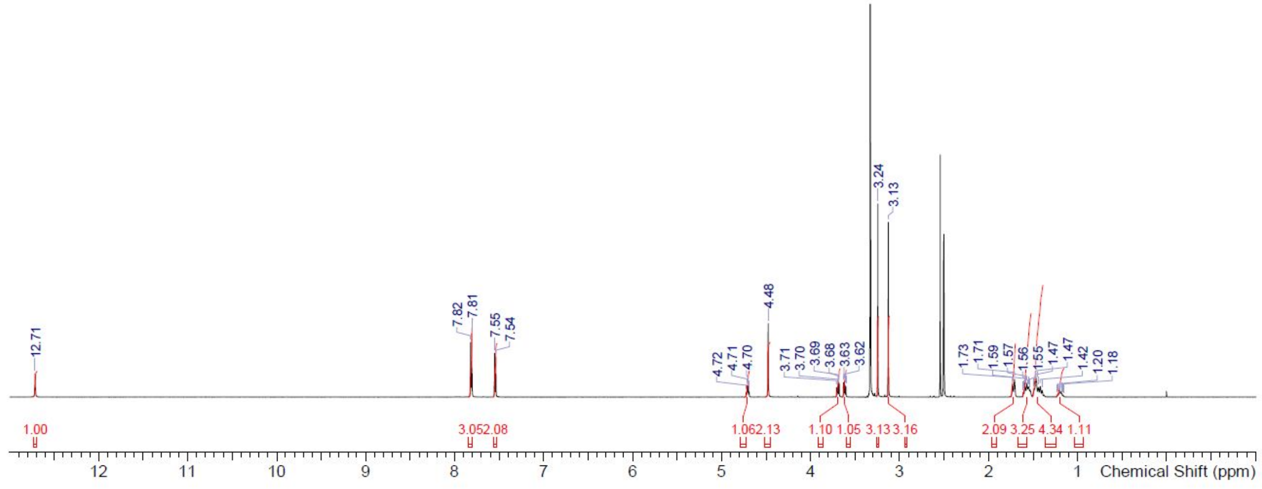

^{13}C-NMR of compound **15**:

Frequency (MHz)	125.7704	*Nucleus*	13C
Number of Transients	1024	*Origin*	Bruker BioSpin GmbH
Original Points Count	32768	*Owner*	x
Points Count	65536	*Pulse Sequence*	zgpg
SW(cyclical) (Hz)	30120.02	*Solvent*	DMSO-d6
Spectrum Offset (Hz)	12505.4326	*Spectrum Type*	standard
Sweep Width (Hz)	30119.56	*Temperature (degree C)*	25.008

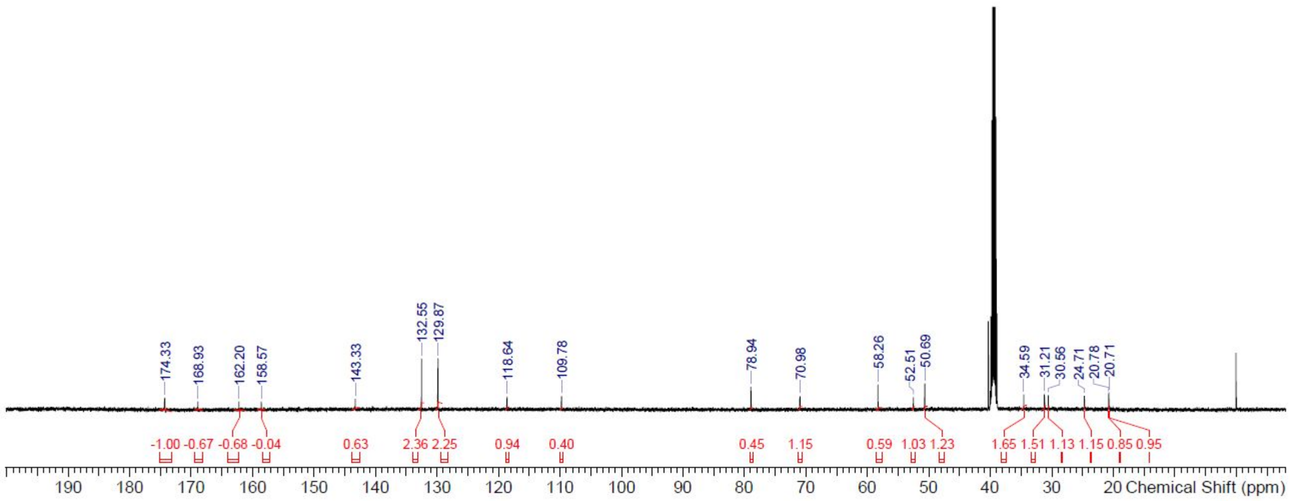

^{1}H-NMR of compound **16**:

Frequency (MHz)	600.2845	*Nucleus*	1H
Number of Transients	16	*Origin*	Bruker BioSpin GmbH
Original Points Count	32768	*Owner*	x
Points Count	65536	*Pulse Sequence*	zg30
SW(cyclical) (Hz)	12019.05	*Solvent*	DMSO-d6
Spectrum Offset (Hz)	4481.9116	*Spectrum Type*	standard
Sweep Width (Hz)	12018.86	*Temperature (degree C)*	25.001

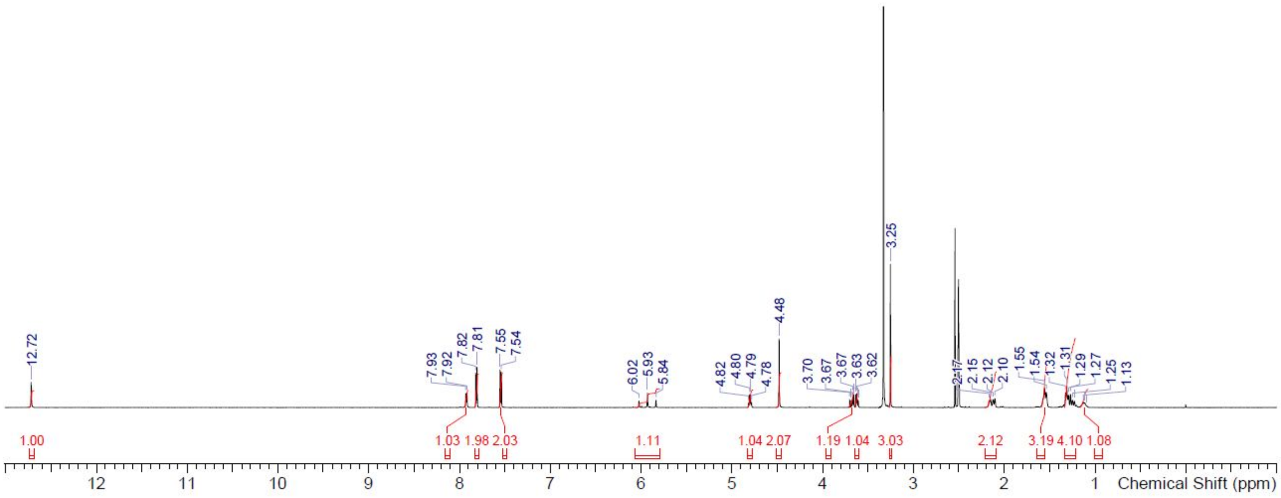

^{13}C-NMR of compound **16**:

File Name	C:\Users\GMILY\AppData\Local\Temp\CLJ\SpectraDownloads\dsdp372-1-2.323397.13c-nmr.477359.0.dx		
Frequency (MHz)	125.7704	Nucleus	13C
Number of Transients	640	Origin	Bruker BioSpin GmbH
Original Points Count	32768	Owner	x
Points Count	65536	Pulse Sequence	zgpg
SW(cyclical) (Hz)	30120.02	Solvent	DMSO-d6
Spectrum Offset (Hz)	12506.2119	Spectrum Type	standard
Sweep Width (Hz)	30119.56	Temperature (degree C)	24.997

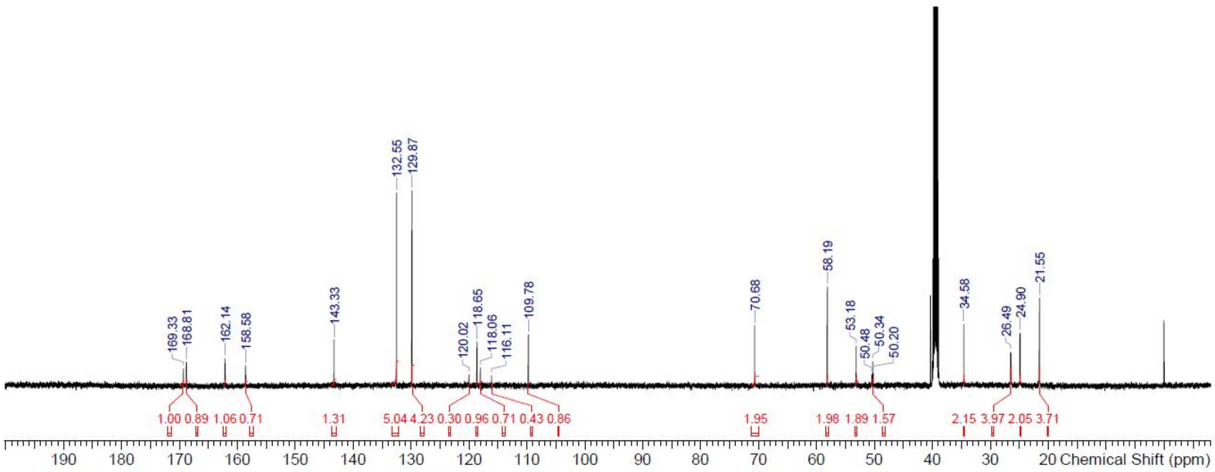

^{1}H-NMR of compound **17**:

Frequency (MHz)	500.1331	Nucleus	1H
Number of Transients	32	Origin	Bruker BioSpin GmbH
Original Points Count	16384	Owner	x
Points Count	65536	Pulse Sequence	zg30
SW(cyclical) (Hz)	9999.85	Solvent	DMSO-d6
Spectrum Offset (Hz)	3084.8872	Spectrum Type	standard
Sweep Width (Hz)	9999.70	Temperature (degree C)	24.994

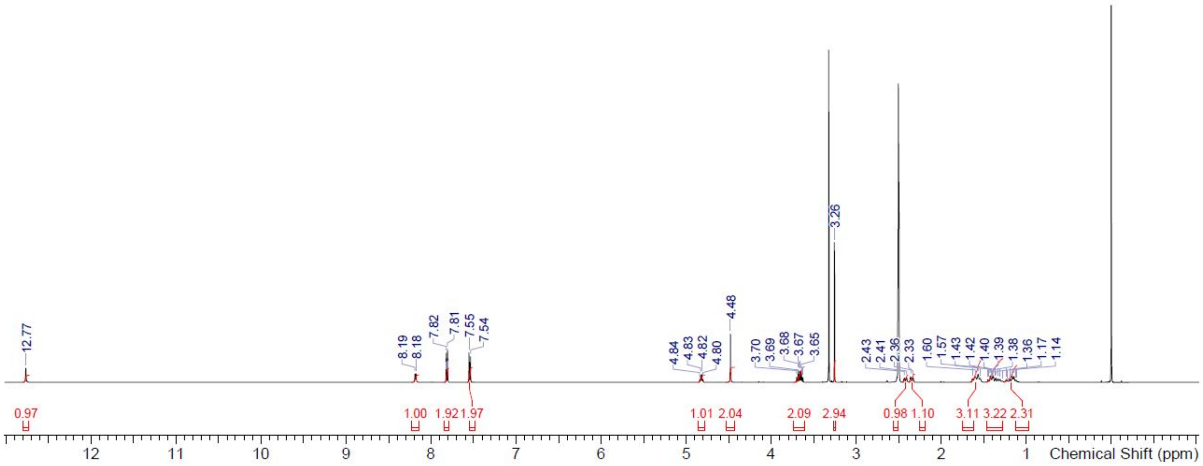

^{13}C-NMR of compound **17**:

Frequency (MHz)	125.7704	*Nucleus*	13C
Number of Transients	640	*Origin*	Bruker BioSpin GmbH
Original Points Count	32768	*Owner*	x
Points Count	65536	*Pulse Sequence*	zgpg
SW(cyclical) (Hz)	30120.02	*Solvent*	DMSO-d6
Spectrum Offset (Hz)	12505.3701	*Spectrum Type*	standard
Sweep Width (Hz)	30119.56	*Temperature (degree C)*	25.014

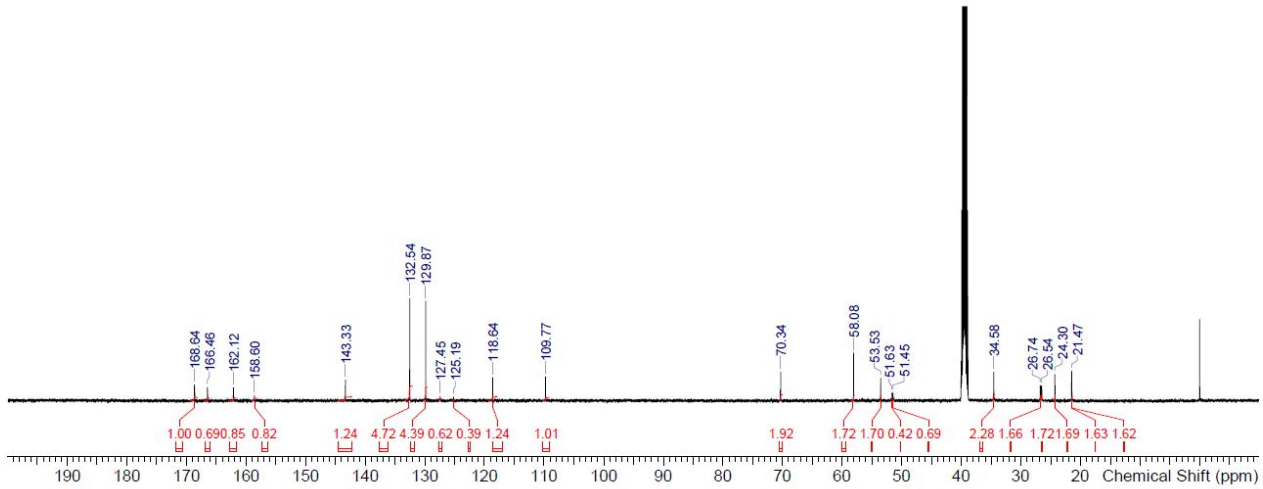

^{1}H-NMR of compound **18**:

Frequency (MHz)	600.2845	*Nucleus*	1H
Number of Transients	16	*Origin*	Bruker BioSpin GmbH
Original Points Count	32768	*Owner*	x
Points Count	65536	*Pulse Sequence*	zg30
SW(cyclical) (Hz)	12820.32	*Solvent*	DMSO-d6
Spectrum Offset (Hz)	4480.7192	*Spectrum Type*	standard
Sweep Width (Hz)	12820.12	*Temperature (degree C)*	25.000

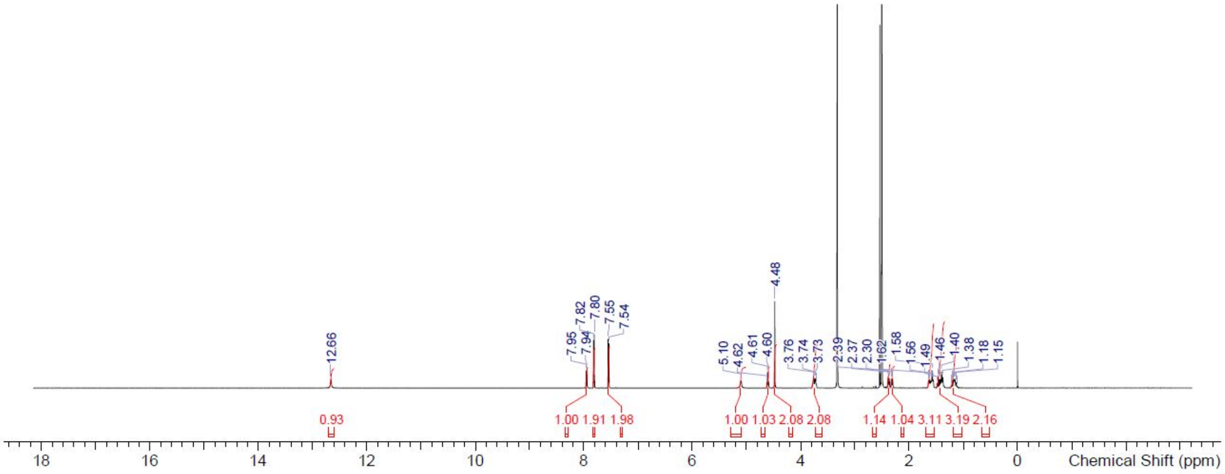

^{13}C-NMR of compound **18**:

Frequency (MHz)	125.7704	Nucleus	13C
Number of Transients	512	Origin	Bruker BioSpin GmbH
Original Points Count	32768	Owner	x
Points Count	65536	Pulse Sequence	zgpg
SW(cyclical) (Hz)	30120.02	Solvent	DMSO-d6
Spectrum Offset (Hz)	12505.3447	Spectrum Type	standard
Sweep Width (Hz)	30119.56	Temperature (degree C)	25.000

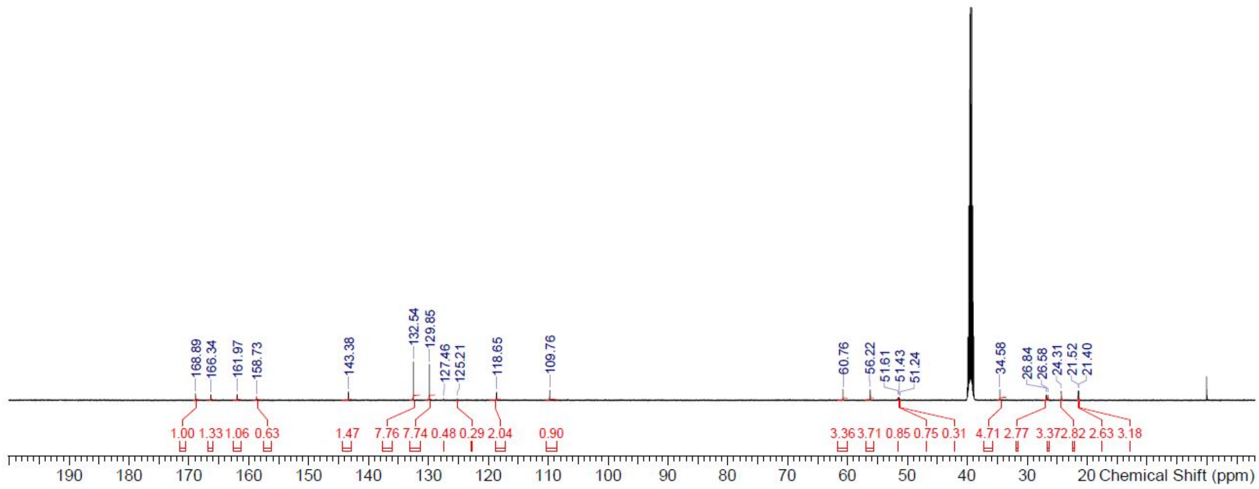

^{1}H-NMR of compound **19**:

Frequency (MHz)	600.2845	Nucleus	1H
Number of Transients	16	Origin	Bruker BioSpin GmbH
Original Points Count	32768	Owner	x
Points Count	65536	Pulse Sequence	zg30
SW(cyclical) (Hz)	12820.32	Solvent	DMSO-d6
Spectrum Offset (Hz)	4480.9224	Spectrum Type	standard
Sweep Width (Hz)	12820.12	Temperature (degree C)	24.998

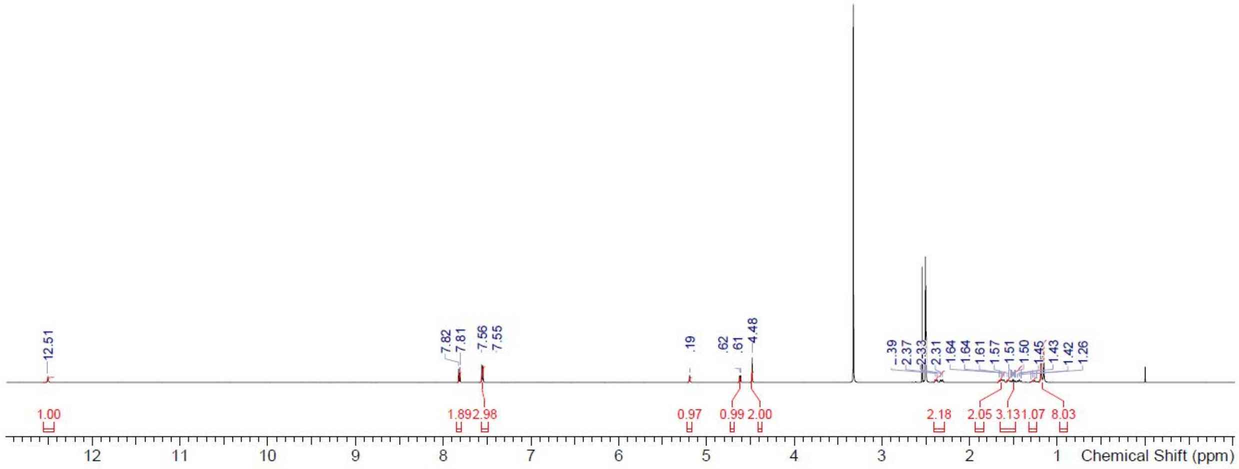

^{13}C-NMR of compound **19**:

Frequency (MHz)	125.7704	*Nucleus*	13C
Number of Transients	384	*Origin*	Bruker BioSpin GmbH
Original Points Count	32768	*Owner*	x
Points Count	65536	*Pulse Sequence*	zgpg
SW(cyclical) (Hz)	30120.02	*Solvent*	DMSO-d6
Spectrum Offset (Hz)	12505.0166	*Spectrum Type*	standard
Sweep Width (Hz)	30119.56	*Temperature (degree C)*	25.002

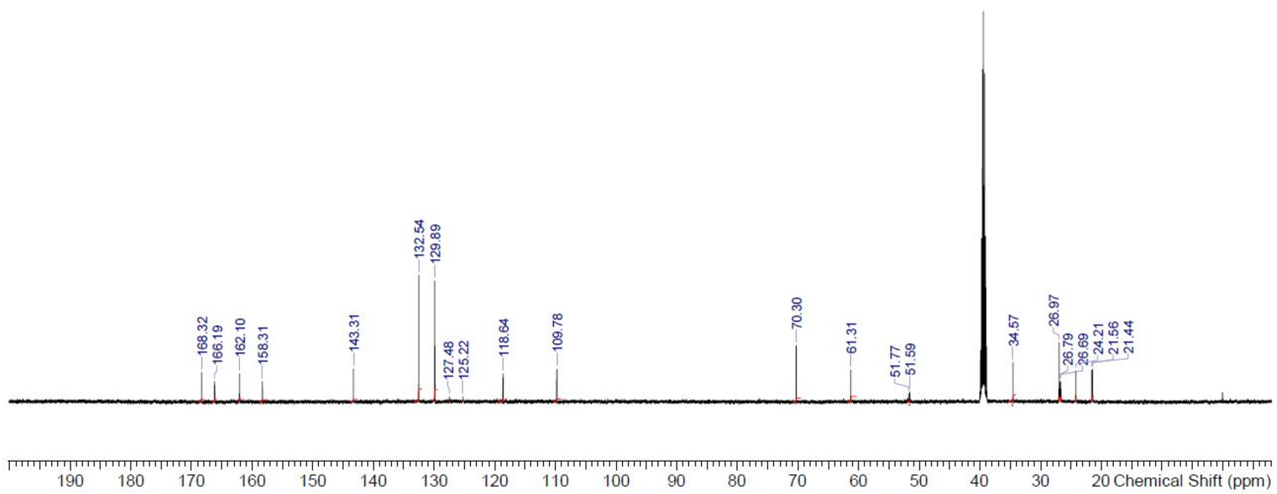

^{1}H-NMR of compound **20**:

Frequency (MHz)	500.1331	*Nucleus*	1H
Number of Transients	32	*Origin*	Bruker BioSpin GmbH
Original Points Count	16384	*Owner*	x
Points Count	65536	*Pulse Sequence*	zg30
SW(cyclical) (Hz)	9999.85	*Solvent*	DMSO-d6
Spectrum Offset (Hz)	3087.4380	*Spectrum Type*	standard
Sweep Width (Hz)	9999.70	*Temperature (degree C)*	25.009

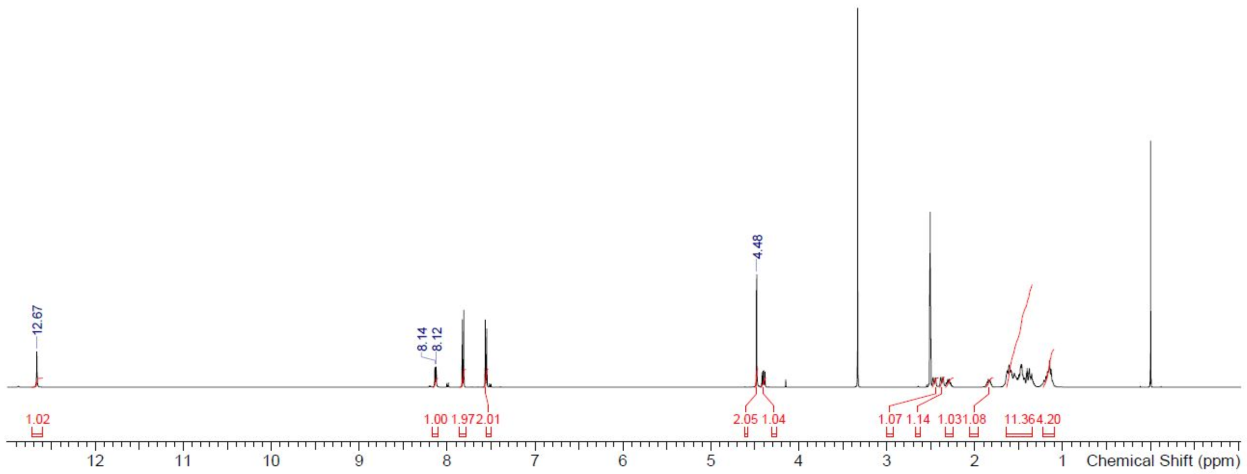

^{13}C-NMR of compound **20**:

Frequency (MHz)	125.7704	*Nucleus*	13C
Number of Transients	384	*Origin*	Bruker BioSpin GmbH
Original Points Count	32768	*Owner*	x
Points Count	65536	*Pulse Sequence*	zgpg
SW(cyclical) (Hz)	30120.02	*Solvent*	DMSO-d6
Spectrum Offset (Hz)	12507.4580	*Spectrum Type*	standard
Sweep Width (Hz)	30119.56	*Temperature (degree C)*	24.997

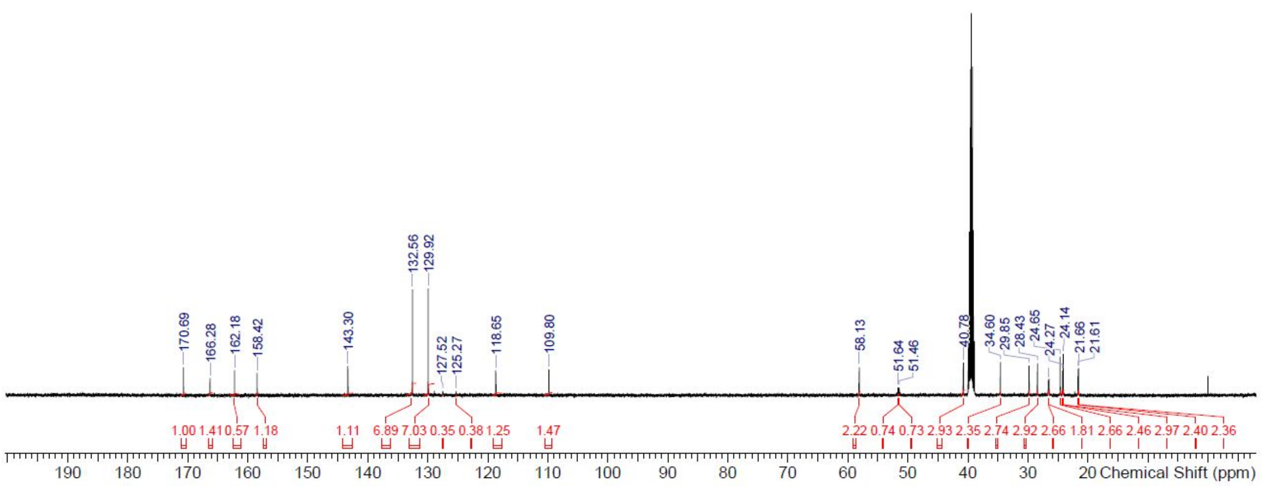

^{1}H-NMR of compound **21** (BAY-805)

Frequency (MHz)	600.2845	*Nucleus*	1H
Number of Transients	16	*Origin*	Bruker BioSpin GmbH
Original Points Count	32768	*Owner*	x
Points Count	65536	*Pulse Sequence*	zg30
SW(cyclical) (Hz)	12820.32	*Solvent*	DMSO-d6
Spectrum Offset (Hz)	4481.8296	*Spectrum Type*	standard
Sweep Width (Hz)	12820.12	*Temperature (degree C)*	25.002

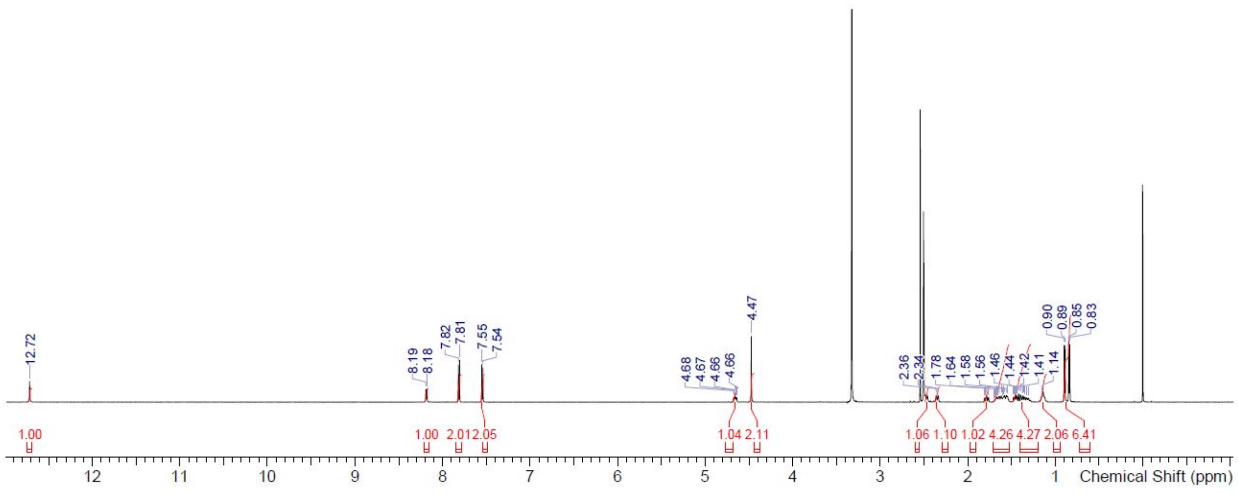

^{13}C-NMR of BAY-805 (**21**):

Frequency (MHz)	125.7704	*Nucleus*	13C
Number of Transients	256	*Origin*	Bruker BioSpin GmbH
Original Points Count	32768	*Owner*	x
Points Count	65536	*Pulse Sequence*	zgpg
SW(cyclical) (Hz)	30120.02	*Solvent*	DMSO-d6
Spectrum Offset (Hz)	12578.7803	*Spectrum Type*	standard
Sweep Width (Hz)	30119.56	*Temperature (degree C)*	24.999

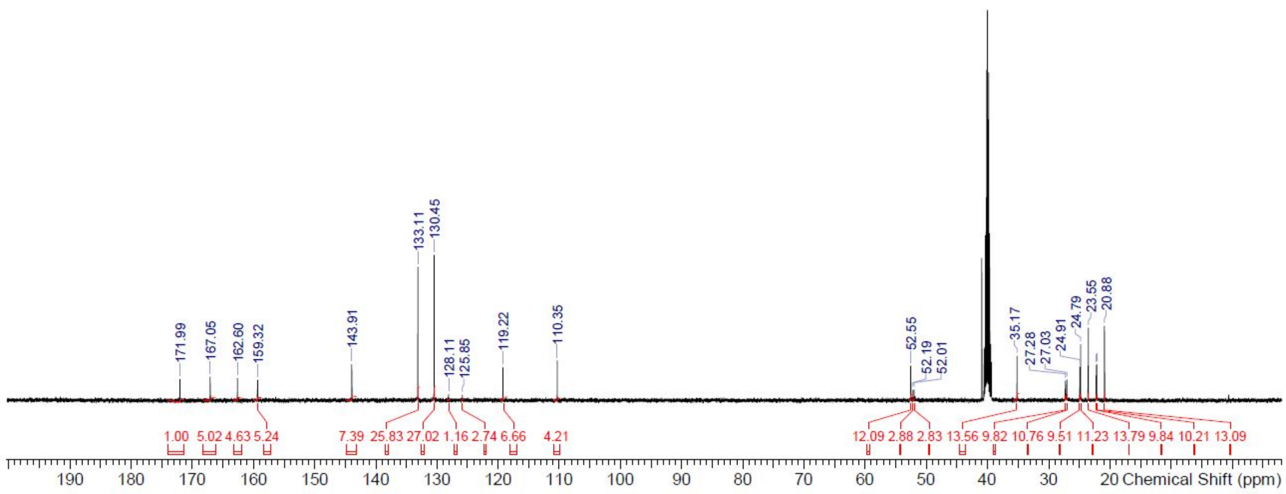

^{1}H-NMR of compound **22**:

Frequency (MHz)	600.2845	*Nucleus*	1H
Number of Transients	16	*Origin*	Bruker BioSpin GmbH
Original Points Count	32768	*Owner*	x
Points Count	65536	*Pulse Sequence*	zg30
SW(cyclical) (Hz)	12820.32	*Solvent*	DMSO-d6
Spectrum Offset (Hz)	4481.7515	*Spectrum Type*	standard
Sweep Width (Hz)	12820.12	*Temperature (degree C)*	25.002

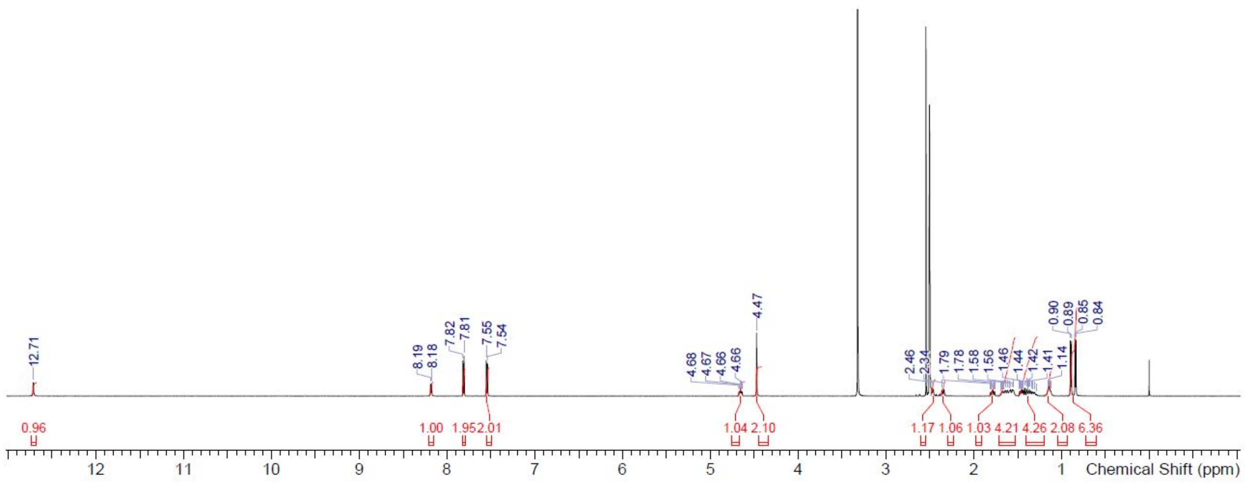

^{13}C-NMR of compound **22**:

Frequency (MHz)	125.7704	Nucleus	13C
Number of Transients	256	Origin	Bruker BioSpin GmbH
Original Points Count	32768	Owner	x
Points Count	65536	Pulse Sequence	zgpg
SW(cyclical) (Hz)	30120.02	Solvent	DMSO-d6
Spectrum Offset (Hz)	12578.7803	Spectrum Type	standard
Sweep Width (Hz)	30119.56	Temperature (degree C)	25.001

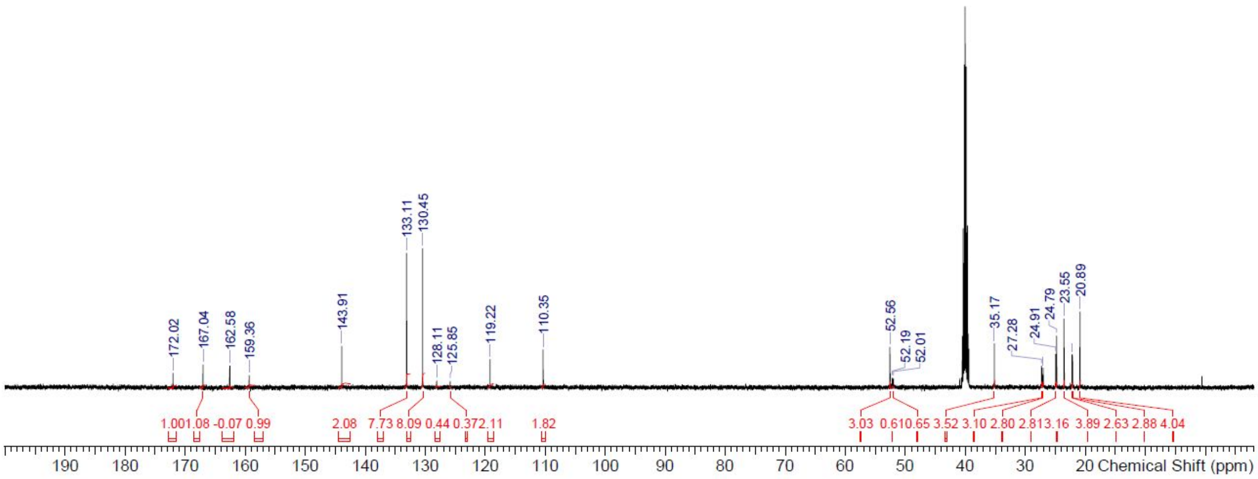

^{1}H-NMR of compound **25**:

Frequency (MHz)	600.2845	Nucleus	1H
Number of Transients	16	Origin	Bruker BioSpin GmbH
Original Points Count	32768	Owner	x
Points Count	65536	Pulse Sequence	zg30
SW(cyclical) (Hz)	12820.32	Solvent	DMSO-d6
Spectrum Offset (Hz)	4482.2495	Spectrum Type	standard
Sweep Width (Hz)	12820.12	Temperature (degree C)	24.998

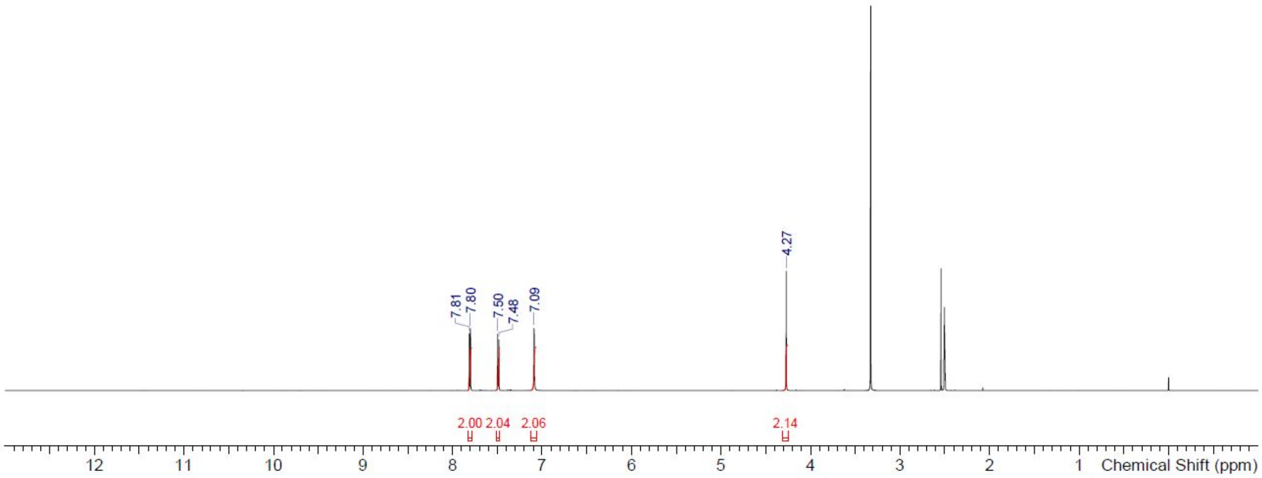

^{1}H-NMR of compound **27**:

Frequency (MHz)	600.2845	Nucleus	1H
Number of Transients	16	Origin	Bruker BioSpin GmbH
Original Points Count	32768	Owner	x
Points Count	65536	Pulse Sequence	zg30
SW(cyclical) (Hz)	12820.32	Solvent	DMSO-d6
Spectrum Offset (Hz)	4481.9019	Spectrum Type	standard
Sweep Width (Hz)	12820.12	Temperature (degree C)	24.999

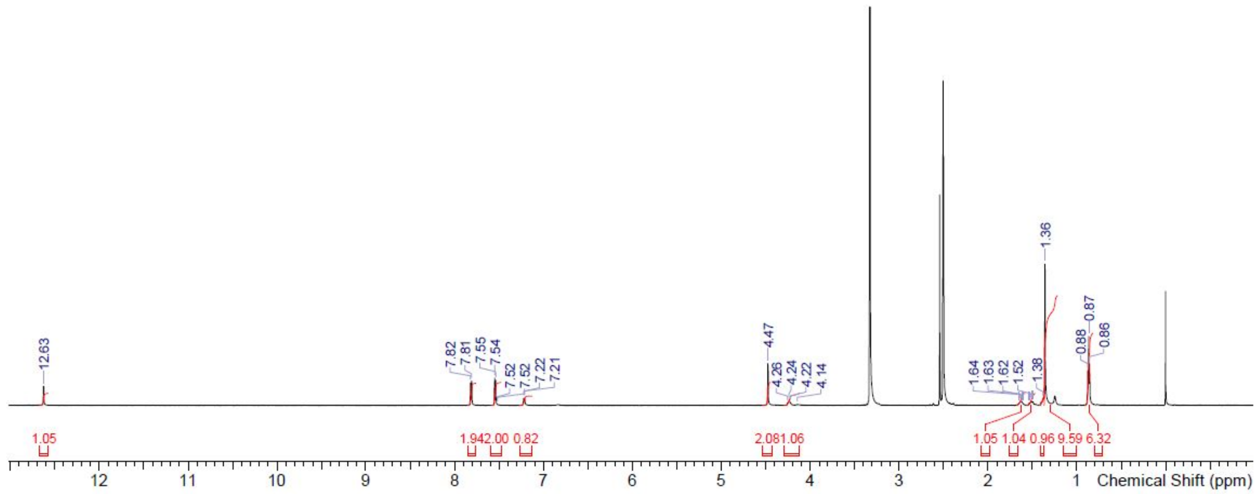

^{1}H-NMR of compound **28**:

Frequency (MHz)	600.2845	Nucleus	1H
Number of Transients	16	Origin	Bruker BioSpin GmbH
Original Points Count	32768	Owner	x
Points Count	65536	Pulse Sequence	zg30
SW(cyclical) (Hz)	12019.05	Solvent	DMSO-d6
Spectrum Offset (Hz)	4483.0952	Spectrum Type	standard
Sweep Width (Hz)	12018.86	Temperature (degree C)	25.000

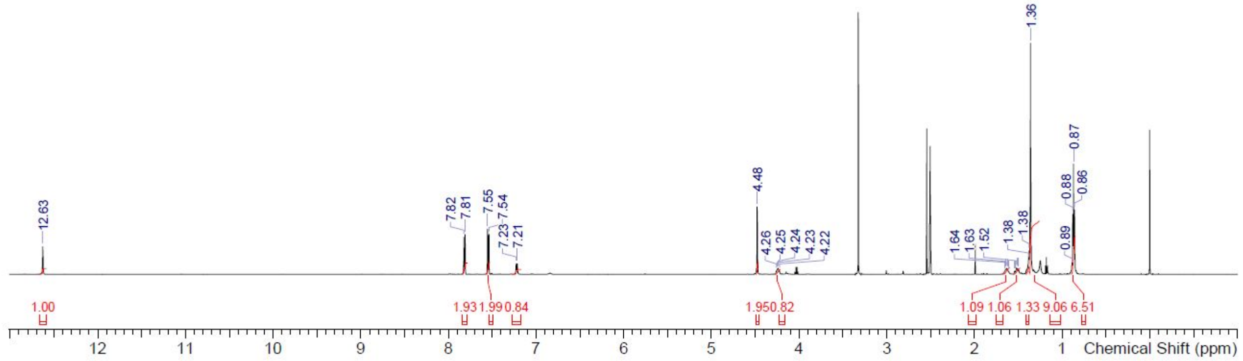

^{1}H-NMR of compound **29**:

Frequency (MHz)	600.2845	Nucleus	1H
Number of Transients	16	Origin	Bruker BioSpin GmbH
Original Points Count	32768	Owner	x
Points Count	65536	Pulse Sequence	zg30
SW(cyclical) (Hz)	12019.05	Solvent	DMSO-d6
Spectrum Offset (Hz)	4483.4487	Spectrum Type	standard
Sweep Width (Hz)	12018.86	Temperature (degree C)	25.000

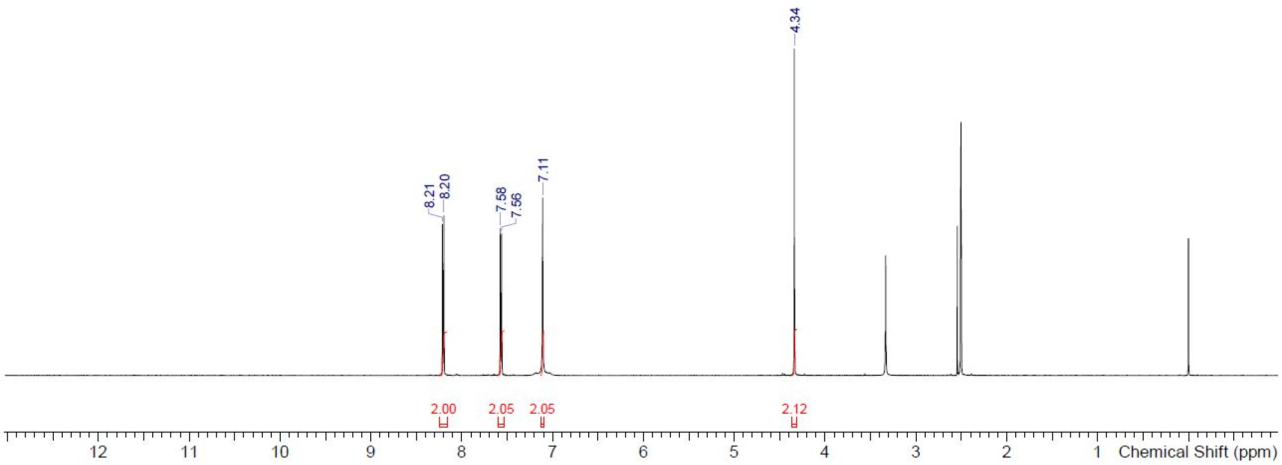

^{1}H-NMR of compound **30**:

Frequency (MHz)	600.2845	Nucleus	1H
Number of Transients	16	Origin	Bruker BioSpin GmbH
Original Points Count	32768	Owner	x
Points Count	65536	Pulse Sequence	zg30
SW(cyclical) (Hz)	12820.32	Solvent	DMSO-d6
Spectrum Offset (Hz)	4481.7329	Spectrum Type	standard
Sweep Width (Hz)	12820.12	Temperature (degree C)	25.318

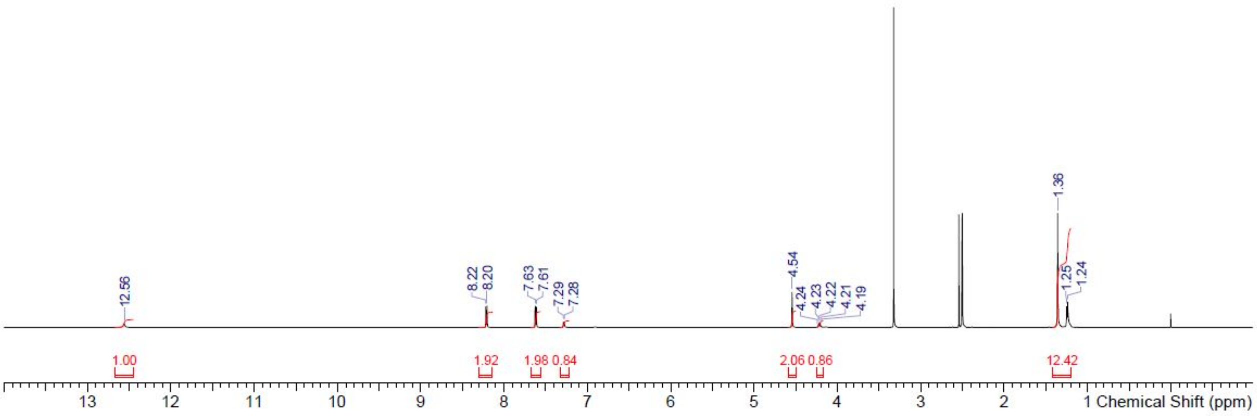

^{1}H-NMR of compound **31**:

Frequency (MHz)	600.2845	Nucleus	1H
Number of Transients	16	Origin	Bruker BioSpin GmbH
Original Points Count	32768	Owner	x
Points Count	65536	Pulse Sequence	zg30
SW(cyclical) (Hz)	12820.32	Solvent	DMSO-d6
Spectrum Offset (Hz)	4481.3560	Spectrum Type	standard
Sweep Width (Hz)	12820.12	Temperature (degree C)	25.321

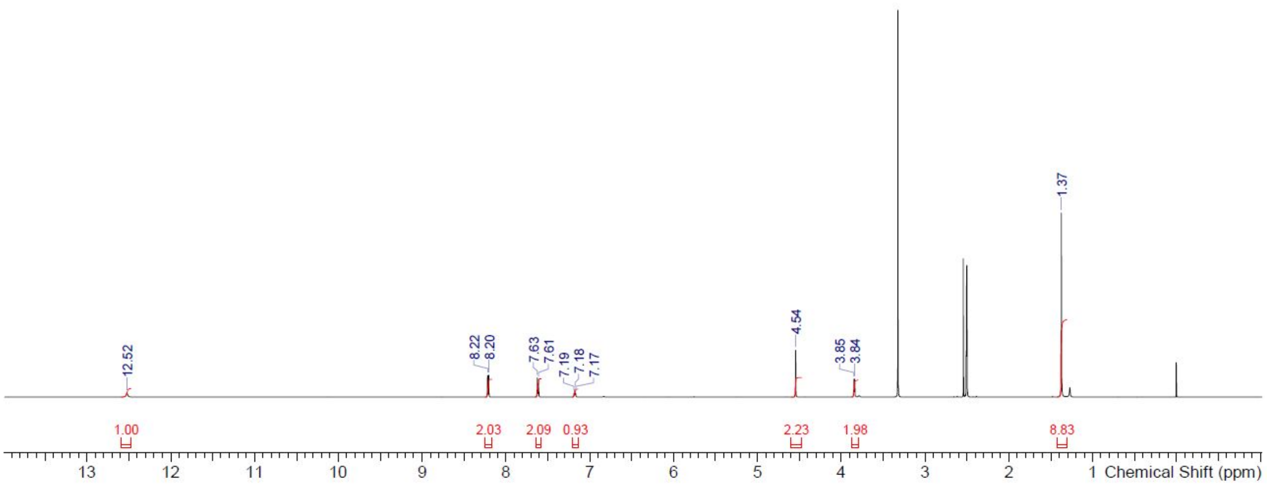

^{1}H-NMR of compound **32**:

Frequency (MHz)	600.2845	Nucleus	1H
Number of Transients	16	Origin	Bruker BioSpin GmbH
Original Points Count	32768	Owner	x
Points Count	65536	Pulse Sequence	zg30
SW(cyclical) (Hz)	12820.32	Solvent	DMSO-d6
Spectrum Offset (Hz)	4481.3003	Spectrum Type	standard
Sweep Width (Hz)	12820.12	Temperature (degree C)	25.327

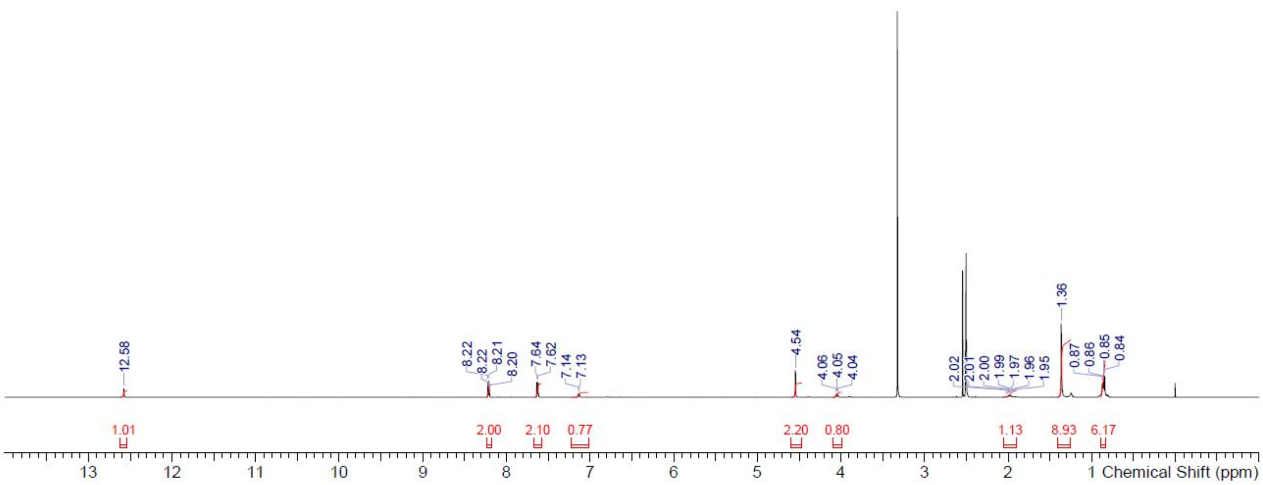

^{1}H-NMR of compound **33**:

Frequency (MHz)	600.2845	Nucleus	1H
Number of Transients	16	Origin	Bruker BioSpin GmbH
Original Points Count	32768	Owner	x
Points Count	65536	Pulse Sequence	zg30
SW(cyclical) (Hz)	12019.05	Solvent	DMSO-d6
Spectrum Offset (Hz)	4483.9536	Spectrum Type	standard
Sweep Width (Hz)	12018.86	Temperature (degree C)	24.997

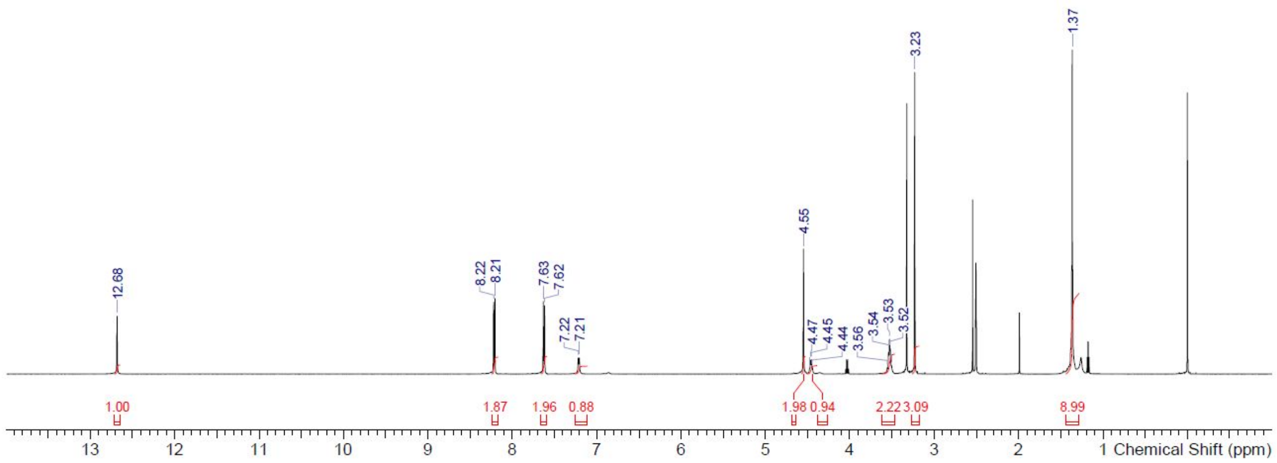

^{1}H-NMR of compound **34**:

Frequency (MHz)	600.2845	Nucleus	1H
Number of Transients	16	Origin	Bruker BioSpin GmbH
Original Points Count	32768	Owner	x
Points Count	65536	Pulse Sequence	zg30
SW(cyclical) (Hz)	12019.05	Solvent	DMSO-d6
Spectrum Offset (Hz)	4485.6646	Spectrum Type	standard
Sweep Width (Hz)	12018.86	Temperature (degree C)	24.999

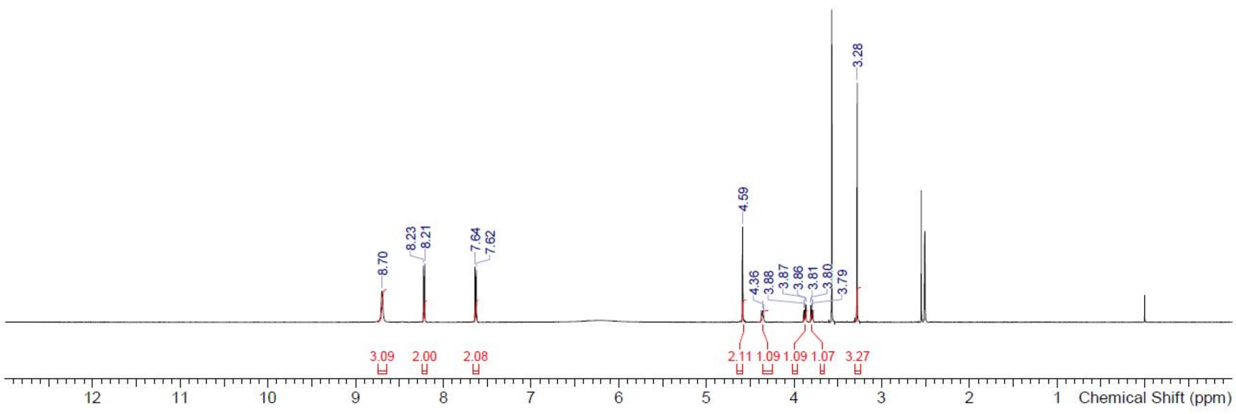

^{1}H-NMR of compound **35**:

Frequency (MHz)	600.2845	*Nucleus*	1H
Number of Transients	16	*Origin*	Bruker BioSpin GmbH
Original Points Count	32768	*Owner*	x
Points Count	65536	*Pulse Sequence*	zg30
SW(cyclical) (Hz)	12820.32	*Solvent*	DMSO-d6
Spectrum Offset (Hz)	4482.7056	*Spectrum Type*	standard
Sweep Width (Hz)	12820.12	*Temperature (degree C)*	25.002

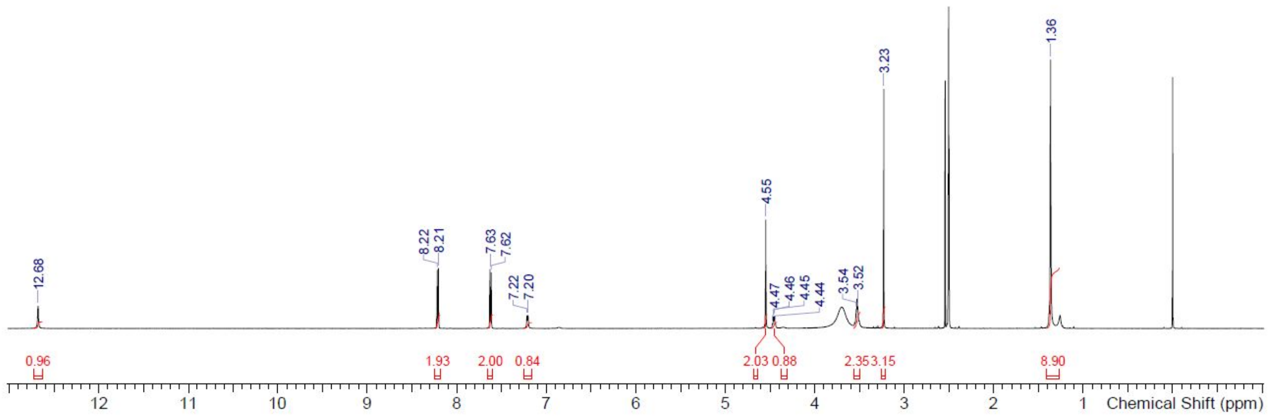

^{1}H-NMR of compound **36**:

Frequency (MHz)	600.2845	*Nucleus*	1H
Number of Transients	16	*Origin*	Bruker BioSpin GmbH
Original Points Count	32768	*Owner*	x
Points Count	65536	*Pulse Sequence*	zg30
SW(cyclical) (Hz)	12820.32	*Solvent*	DMSO-d6
Spectrum Offset (Hz)	4482.6274	*Spectrum Type*	standard
Sweep Width (Hz)	12820.12	*Temperature (degree C)*	25.000

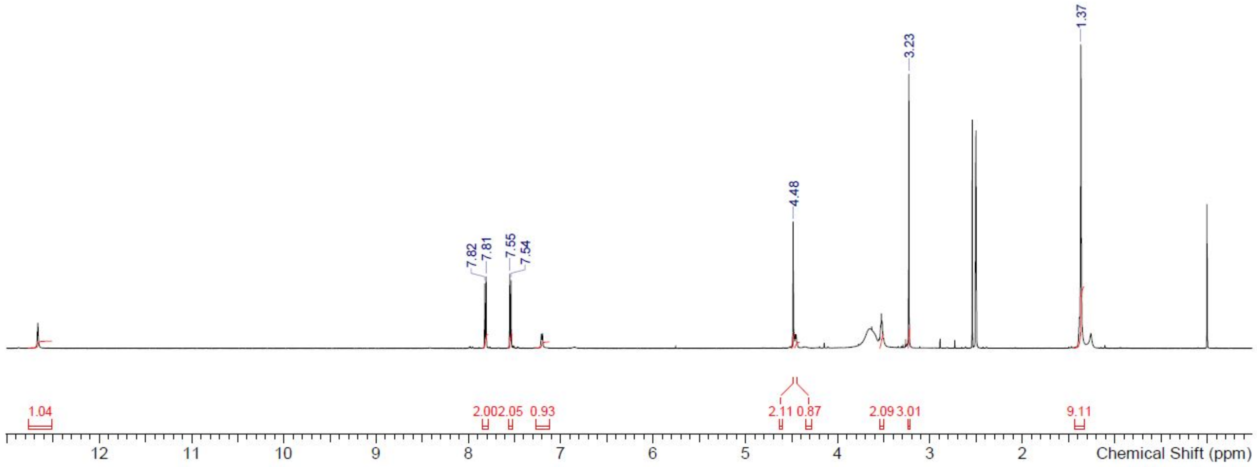

^{1}H-NMR of compound **37**:

File Name	C:\Users\GMILY\AppData\Local\Temp\CLJ\SpectraDownloads\lind7120-1-2.78853102.1h-nmr.2.1.dx		
Frequency (MHz)	600.2845	Nucleus	1H
Number of Transients	16	Origin	Bruker BioSpin GmbH
Original Points Count	32768	Owner	x
Points Count	65536	Pulse Sequence	zg30
SW(cyclical) (Hz)	12820.32	Solvent	DMSO-d6
Spectrum Offset (Hz)	4486.2056	Spectrum Type	standard
Sweep Width (Hz)	12820.12	Temperature (degree C)	24.999

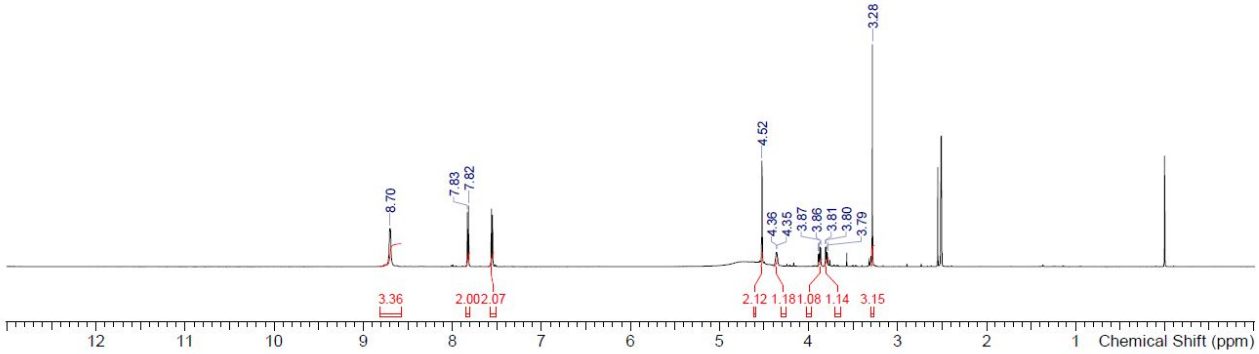

^{1}H-NMR of compound **38**:

Frequency (MHz)	600.2845	Nucleus	1H
Number of Transients	16	Origin	Bruker BioSpin GmbH
Original Points Count	32768	Owner	x
Points Count	65536	Pulse Sequence	zg30
SW(cyclical) (Hz)	12820.32	Solvent	DMSO-d6
Spectrum Offset (Hz)	4483.0298	Spectrum Type	standard
Sweep Width (Hz)	12820.12	Temperature (degree C)	24.996

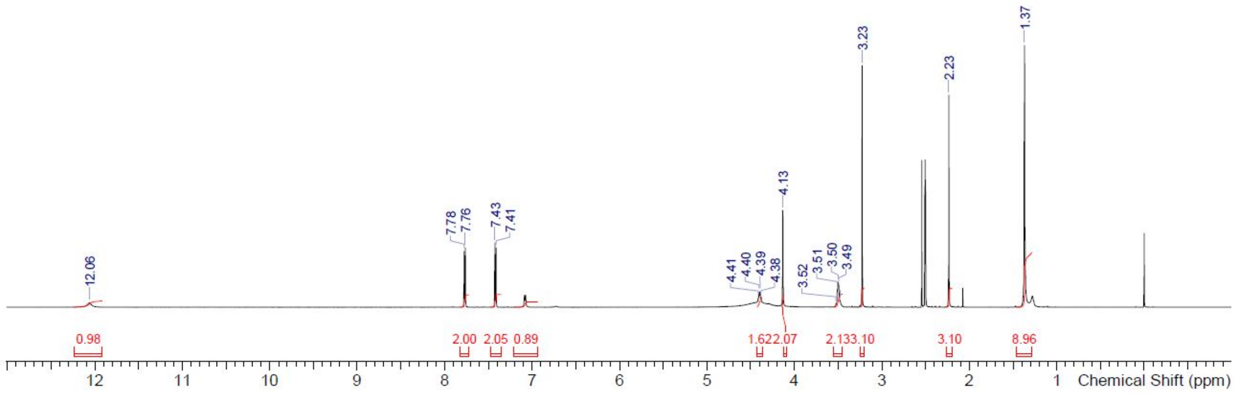

^{1}H-NMR of compound **40**:

Frequency (MHz)	600.2845	Nucleus	1H
Number of Transients	16	Origin	Bruker BioSpin GmbH
Original Points Count	32768	Owner	x
Points Count	65536	Pulse Sequence	zg30
SW(cyclical) (Hz)	12820.32	Solvent	DMSO-d6
Spectrum Offset (Hz)	4481.1147	Spectrum Type	standard
Sweep Width (Hz)	12820.12	Temperature (degree C)	24.995

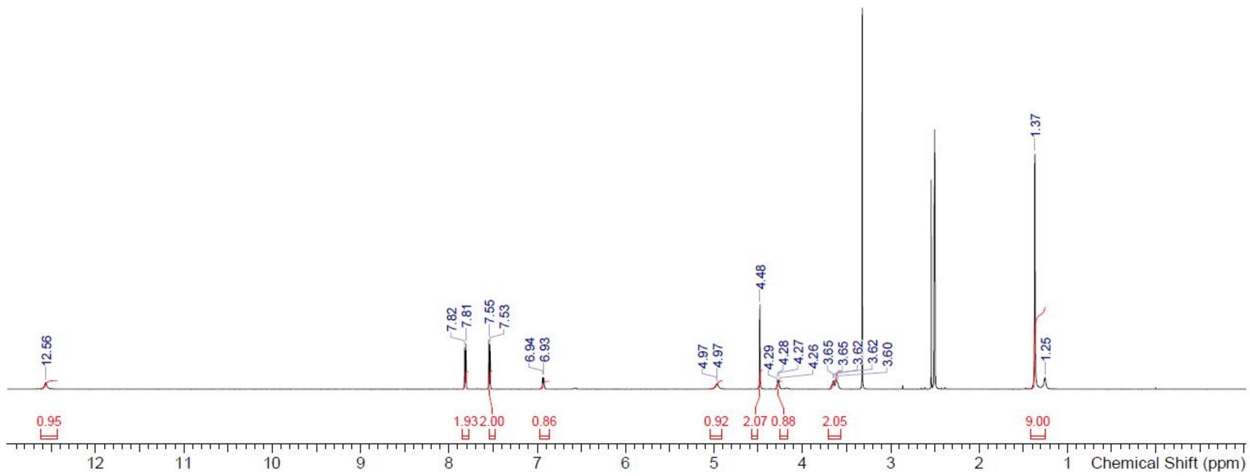

^{1}H-NMR of compound **41**:

Frequency (MHz)	600.2845	Nucleus	1H
Number of Transients	16	Origin	Bruker BioSpin GmbH
Original Points Count	32768	Owner	x
Points Count	65536	Pulse Sequence	zg30
SW(cyclical) (Hz)	12820.32	Solvent	DMSO-d6
Spectrum Offset (Hz)	4502.2808	Spectrum Type	standard
Sweep Width (Hz)	12820.12	Temperature (degree C)	24.994

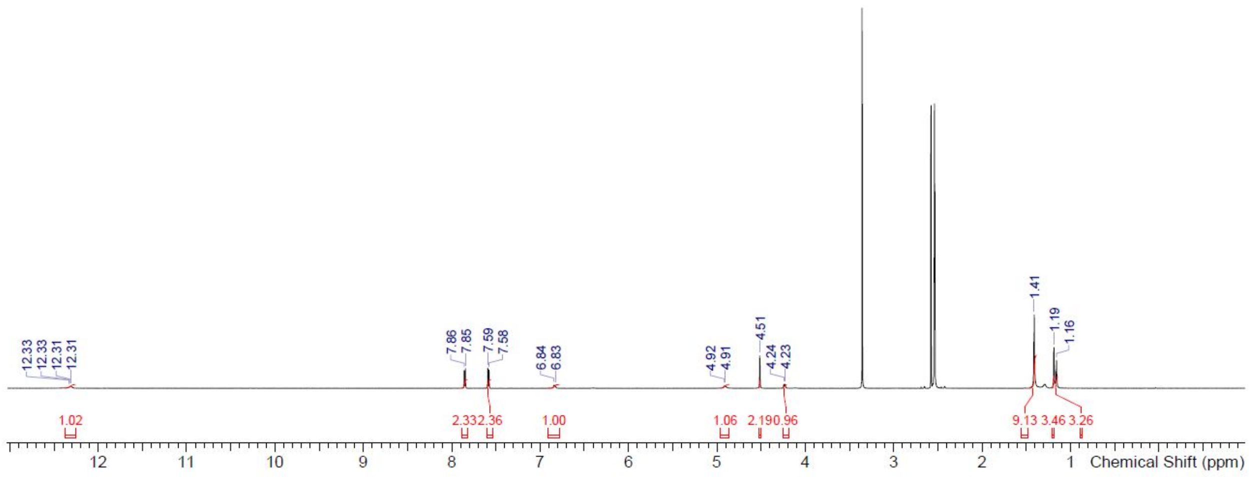

^{1}H-NMR of compound **42**:

Frequency (MHz)	600.2845	*Nucleus*	1H
Number of Transients	16	*Origin*	Bruker BioSpin GmbH
Original Points Count	32768	*Owner*	x
Points Count	65536	*Pulse Sequence*	zg30
SW(cyclical) (Hz)	12019.05	*Solvent*	DMSO-d6
Spectrum Offset (Hz)	4482.3745	*Spectrum Type*	standard
Sweep Width (Hz)	12018.86	*Temperature (degree C)*	25.002

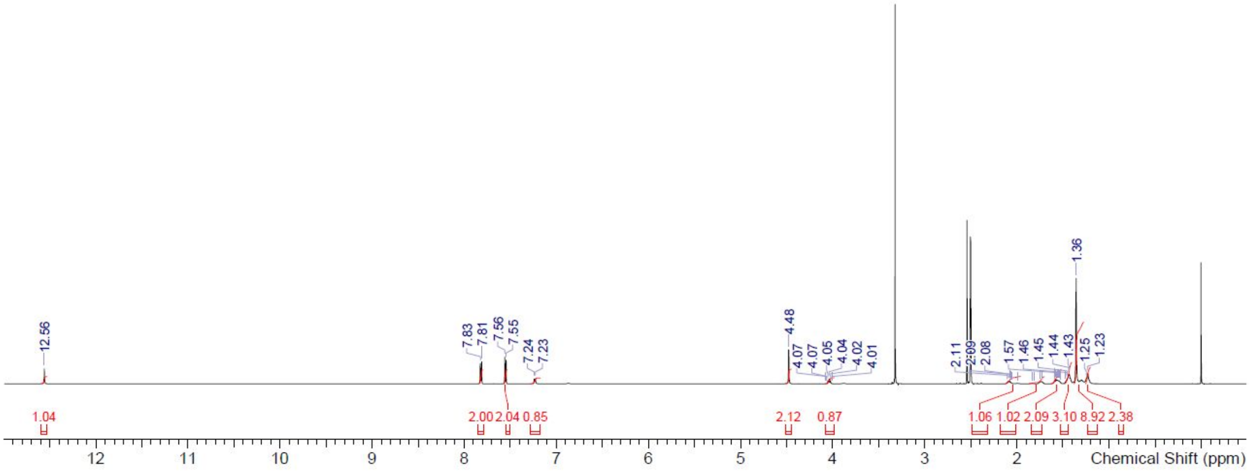

HPLC Chromatograms

LC/MS of compound **1**:

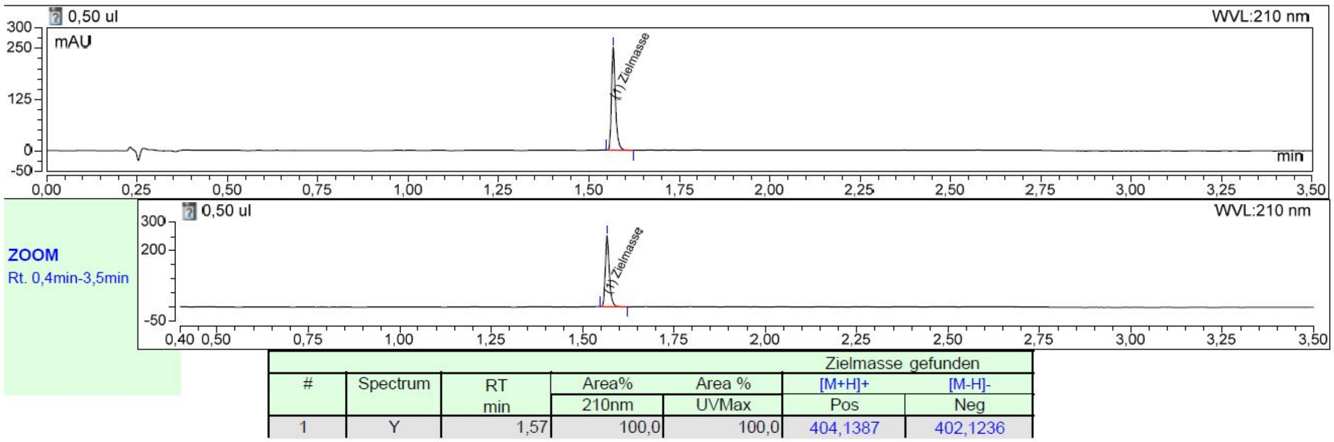

#	Spectrum	RT min	Area% 210nm	Area % UVMax	Zielmasse gefunden [M+H]+ Pos	[M-H]- Neg
1	Y	1,57	100,0	100,0	404,1387	402,1236

LC/MS of compound **2**:

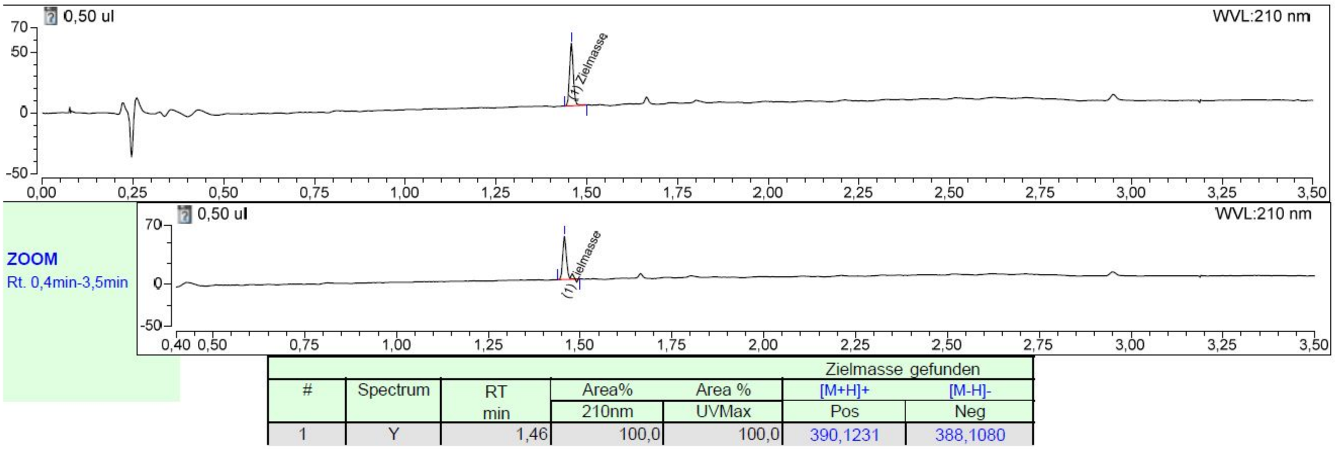

#	Spectrum	RT min	Area% 210nm	Area % UVMax	Zielmasse gefunden [M+H]+ Pos	[M-H]- Neg
1	Y	1,46	100,0	100,0	390,1231	388,1080

LC/MS of compound **3**:

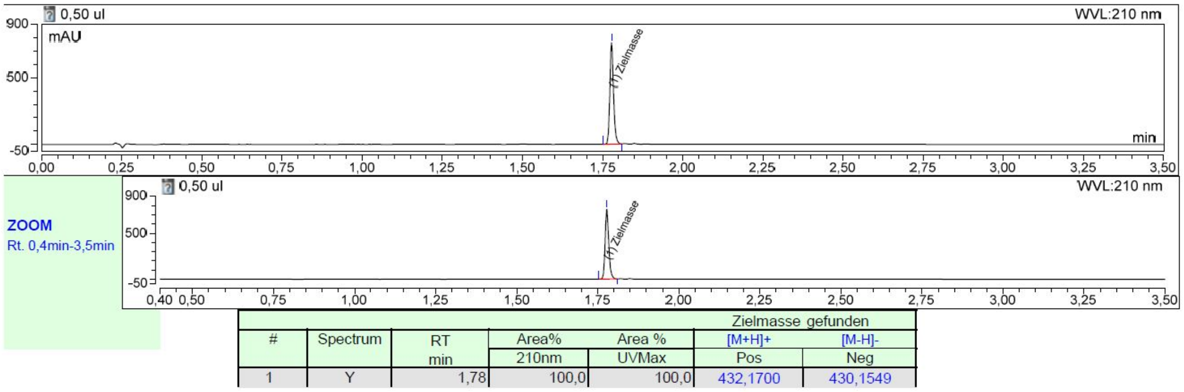

					Zielmasse gefunden	
#	Spectrum	RT	Area%	Area %	[M+H]+	[M-H]-
		min	210nm	UVMax	Pos	Neg
1	Y	1,78	100,0	100,0	432,1700	430,1549

LC/MS of compound **4**:

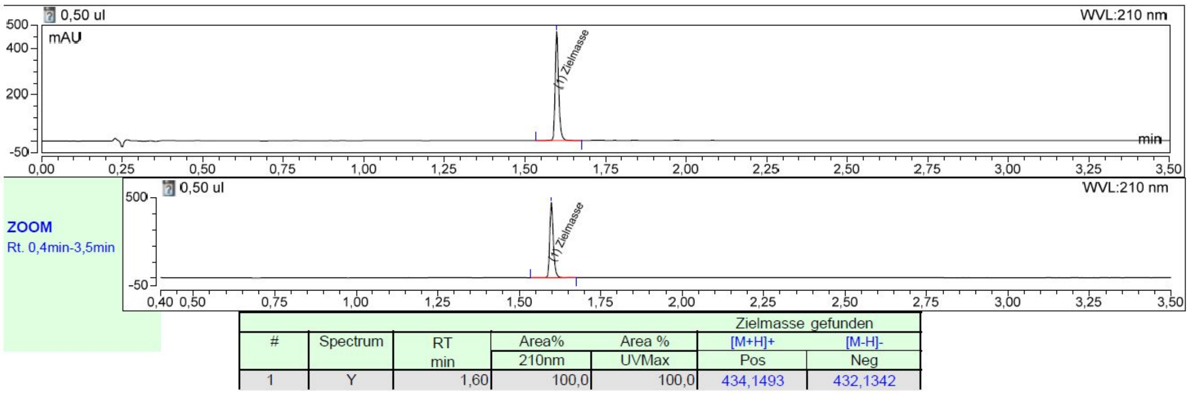

					Zielmasse gefunden	
#	Spectrum	RT	Area%	Area %	[M+H]+	[M-H]-
		min	210nm	UVMax	Pos	Neg
1	Y	1,60	100,0	100,0	434,1493	432,1342

LC/MS of compound **5**:

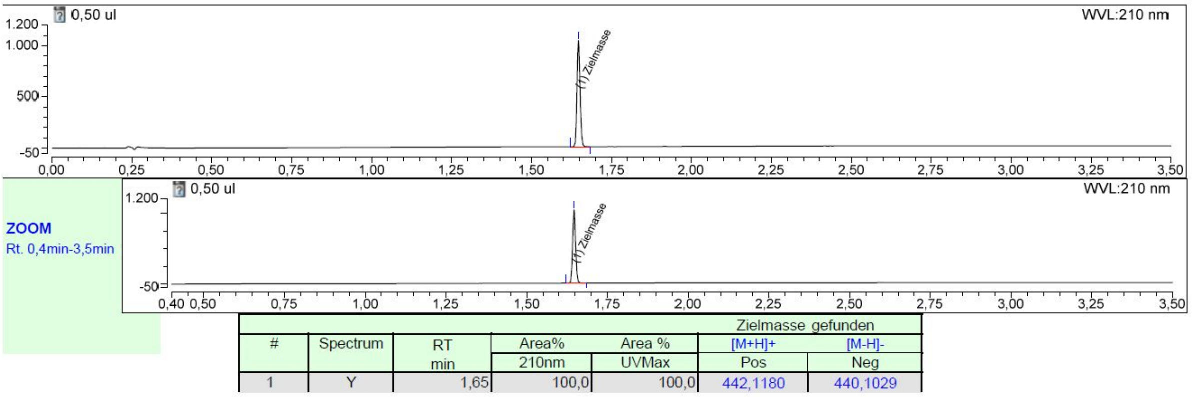

					Zielmasse gefunden	
#	Spectrum	RT	Area%	Area %	[M+H]+	[M-H]-
		min	210nm	UVMax	Pos	Neg
1	Y	1,65	100,0	100,0	442,1180	440,1029

<u>LC/MS of compound **6**:</u>

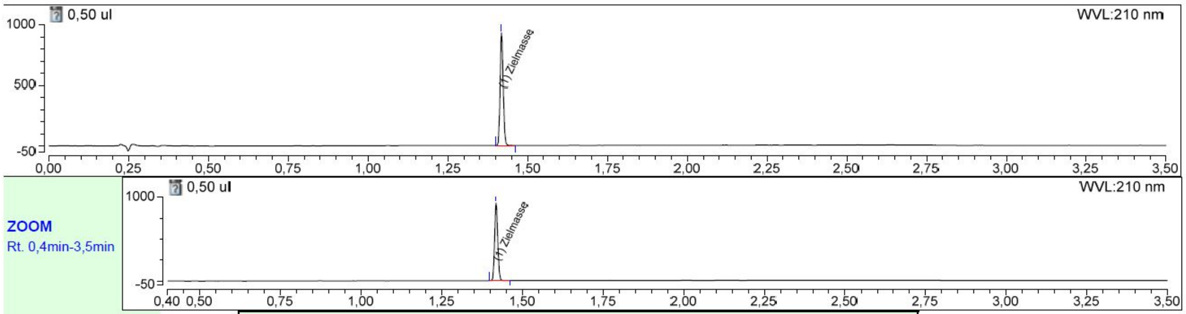

#	Spectrum	RT min	Area% 210nm	Area % UVMax	Zielmasse gefunden [M+H]+ Pos	Zielmasse gefunden [M-H]- Neg
1	Y	1,42	100,0	100,0	408,1336	406,1185

<u>LC/MS of compound **7**:</u>

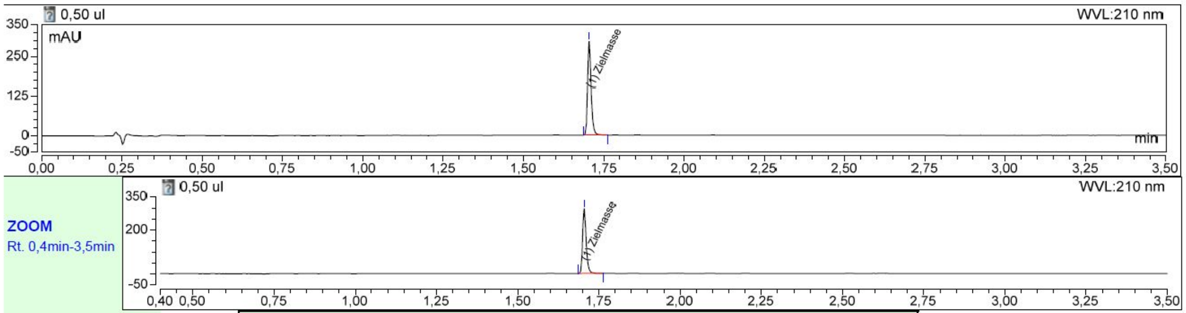

#	Spectrum	RT min	Area% 210nm	Area % UVMax	Zielmasse gefunden [M+H]+ Pos	Zielmasse gefunden [M-H]- Neg
1	Y	1,70	100,0	100,0	448,1649	446,1498

<u>LC/MS of compound **8**:</u>

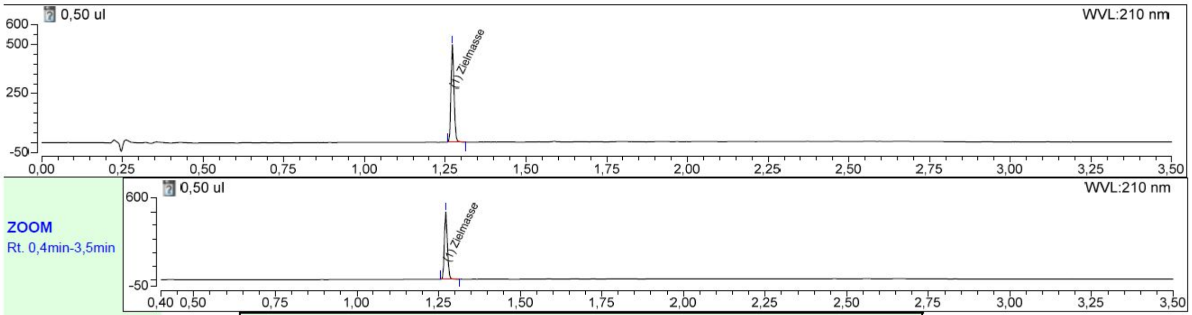

#	Spectrum	RT min	Area% 210nm	Area % UVMax	Zielmasse gefunden [M+H]+ Pos	Zielmasse gefunden [M-H]- Neg
1	Y	1,27	100,0	100,0	450,1442	448,1291

LC/MS of compound **9**:

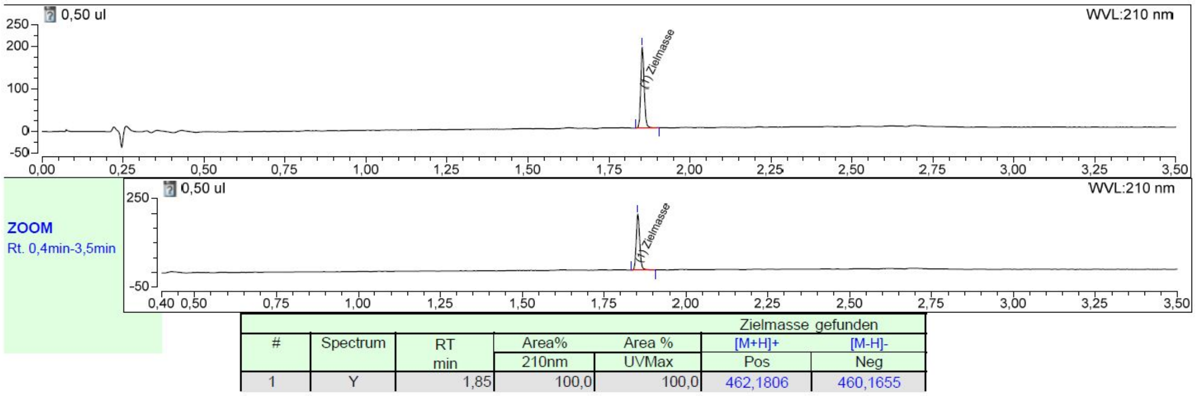

#	Spectrum	RT min	Area% 210nm	Area % UVMax	Zielmasse gefunden [M+H]+ Pos	[M-H]- Neg
1	Y	1,85	100,0	100,0	462,1806	460,1655

LC/MS of compound **10**:

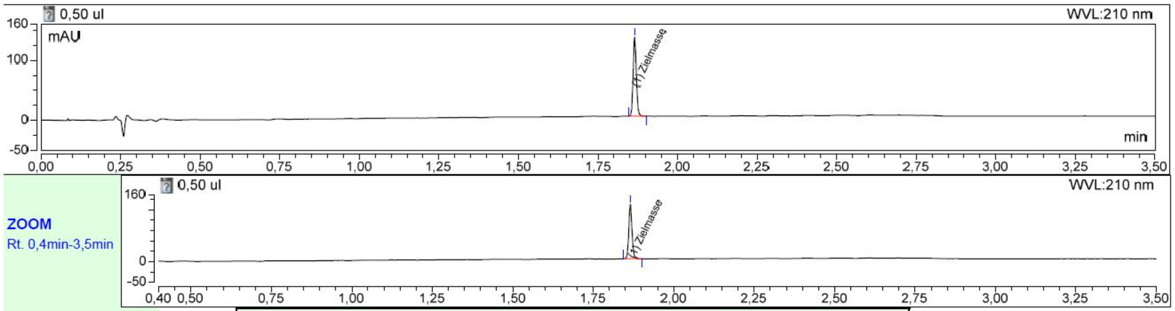

#	Spectrum	RT min	Area% 210nm	Area % UVMax	Zielmasse gefunden [M+H]+ Pos	[M-H]- Neg
1	Y	1,87	100,0	100,0	462,1806	460,1655

LC/MS of compound **11**:

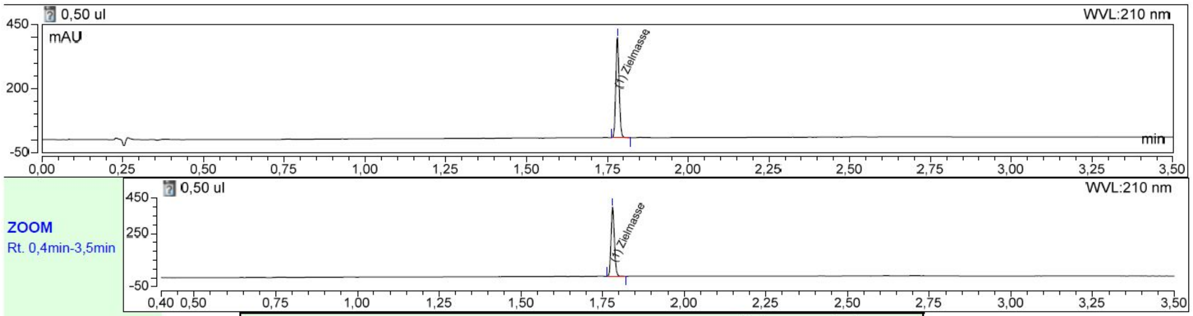

#	Spectrum	RT min	Area% 210nm	Area % UVMax	Zielmasse gefunden [M+H]+ Pos	[M-H]- Neg
1	Y	1,78	100,0	100,0	442,1908	440,1757

LC/MS of compound **12**:

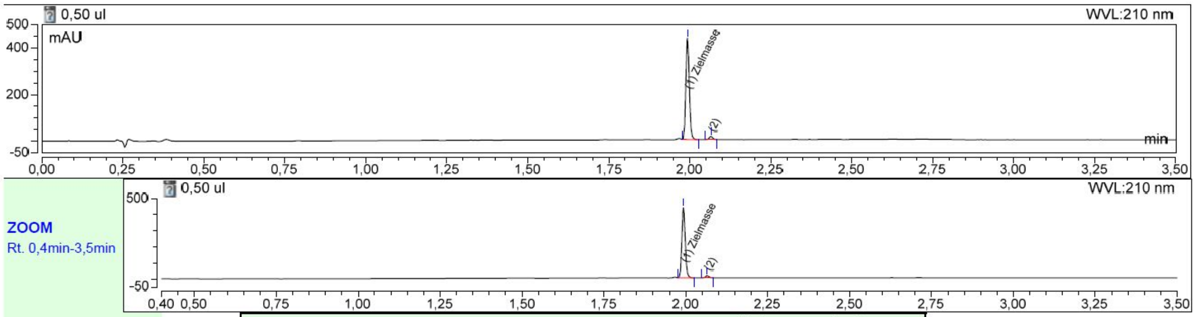

#	Spectrum	RT	Area%	Area %	Zielmasse gefunden	
					[M+H]+	[M-H]-
		min	210nm	UVMax	Pos	Neg
1	Y	1,99	96,9	97,1	455,2112	453,1961
2	Y	2,07	3,1	2,9		

LC/MS of compound **13**:

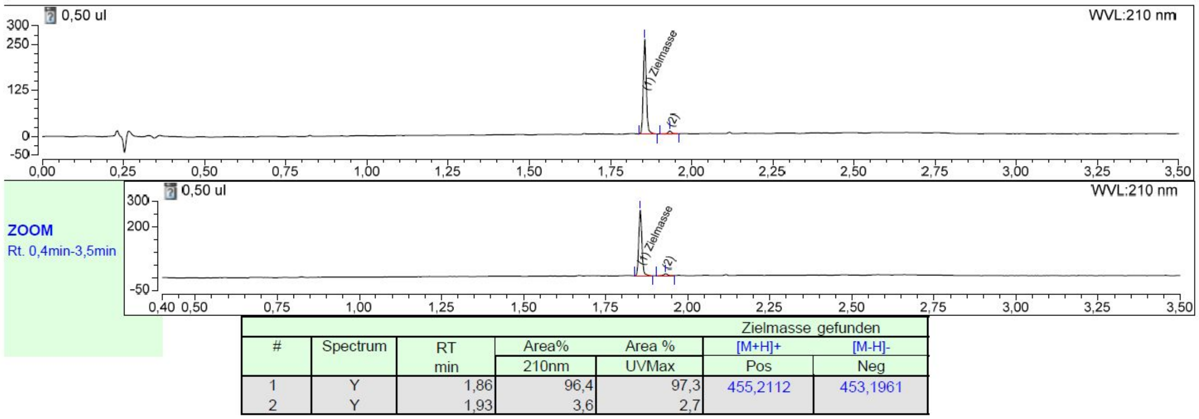

#	Spectrum	RT	Area%	Area %	Zielmasse gefunden	
					[M+H]+	[M-H]-
		min	210nm	UVMax	Pos	Neg
1	Y	1,86	96,4	97,3	455,2112	453,1961
2	Y	1,93	3,6	2,7		

LC/MS of compound **14**:

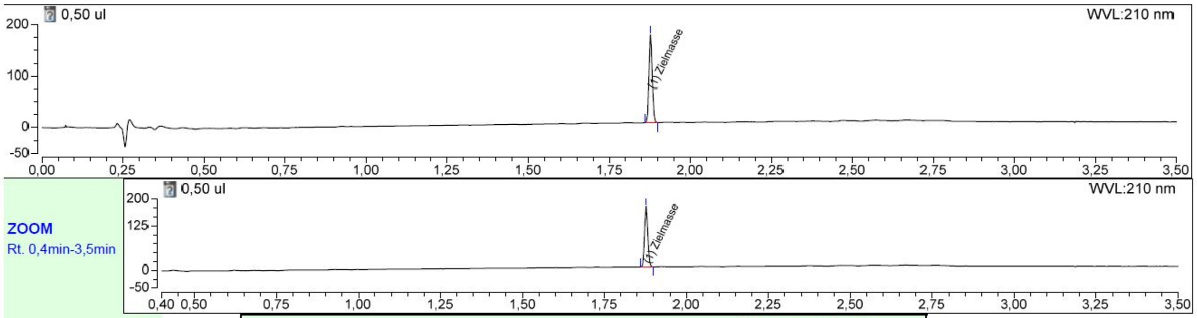

#	Spectrum	RT	Area%	Area %	Zielmasse gefunden	
					[M+H]+	[M-H]-
		min	210nm	UVMax	Pos	Neg
1	Y	1,88	100,0	100,0	456,2064	454,1913

LC/MS of compound **15**:

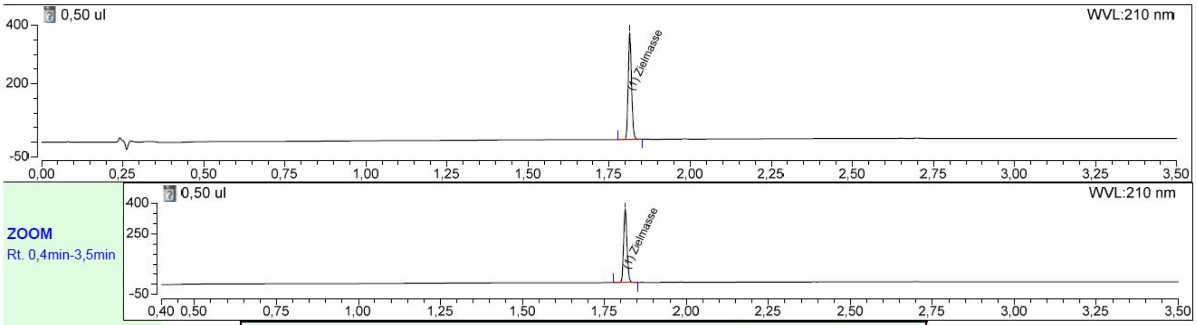

#	Spectrum	RT min	Area% 210nm	Area % UVMax	Zielmasse gefunden [M+H]+ Pos	[M-H]- Neg
1	Y	1,81	100,0	100,0	458,1857	456,1706

LC/MS of compound **16**:

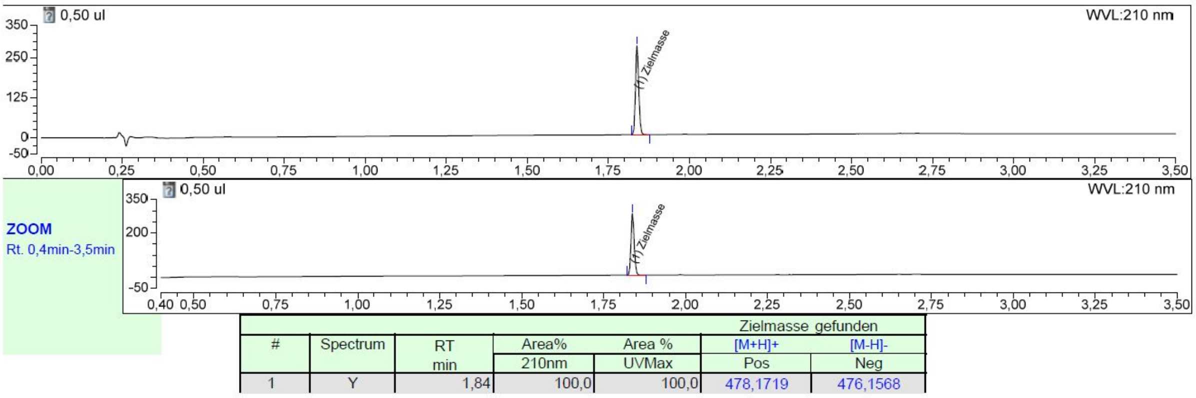

#	Spectrum	RT min	Area% 210nm	Area % UVMax	Zielmasse gefunden [M+H]+ Pos	[M-H]- Neg
1	Y	1,84	100,0	100,0	478,1719	476,1568

LC/MS of compound **17**:

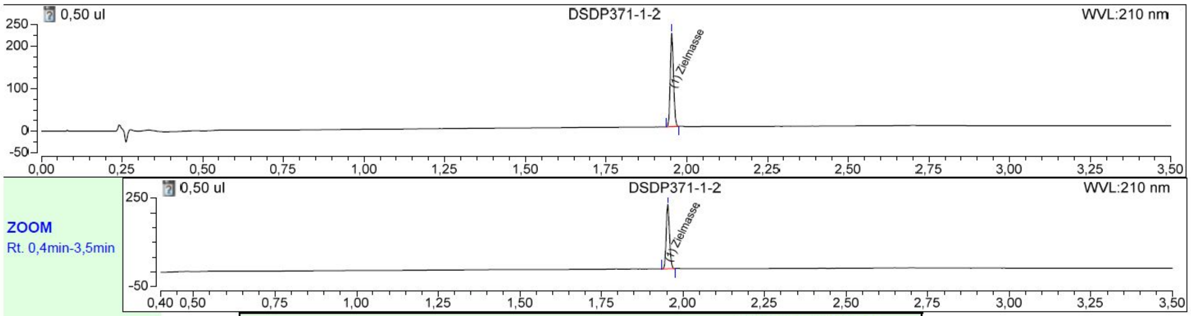

#	Spectrum	RT min	Area% 210nm	Area % UVMax	Zielmasse gefunden [M+H]+ Pos	[M-H]- Neg
1	Y	1,95	100,0	100,0	496,1625	494,1474

LC/MS of compound **18**:

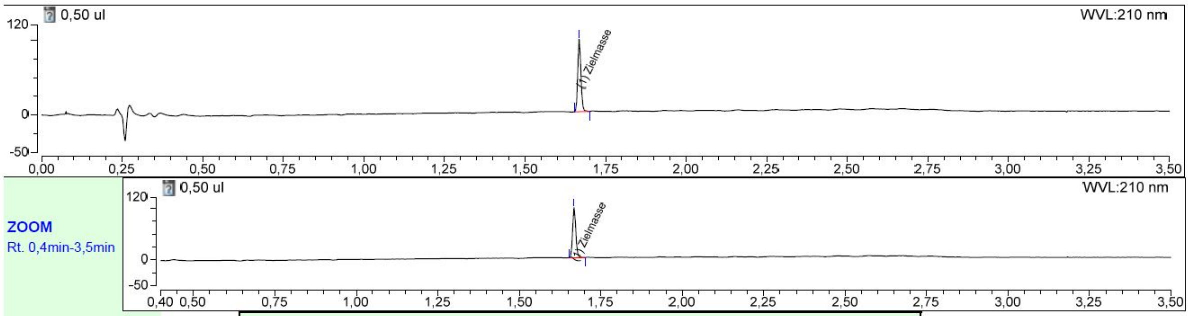

#	Spectrum	RT	Area%	Area %	Zielmasse gefunden	
					[M+H]+	[M-H]-
		min	210nm	UVMax	Pos	Neg
1	Y	1,67	100,0	100,0	482,1468	480,1317

LC/MS of compound **19**:

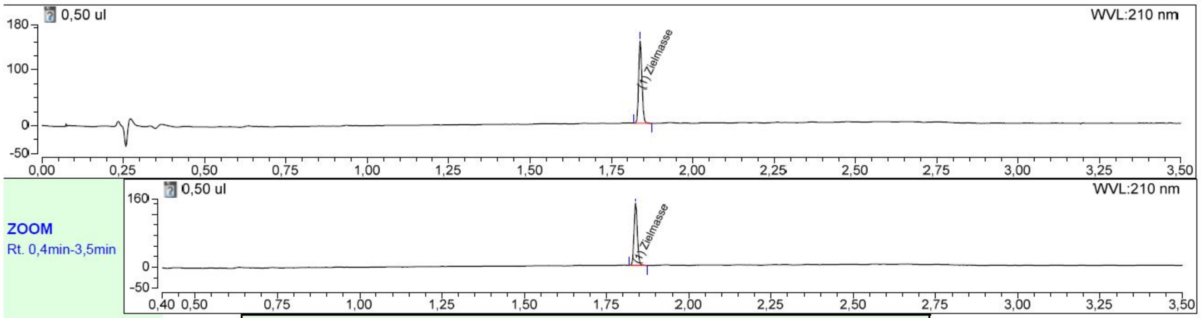

#	Spectrum	RT	Area%	Area %	Zielmasse gefunden	
					[M+H]+	[M-H]-
		min	210nm	UVMax	Pos	Neg
1	Y	1,84	100,0	100,0	510,1781	508,1630

LC/MS of compound **20**:

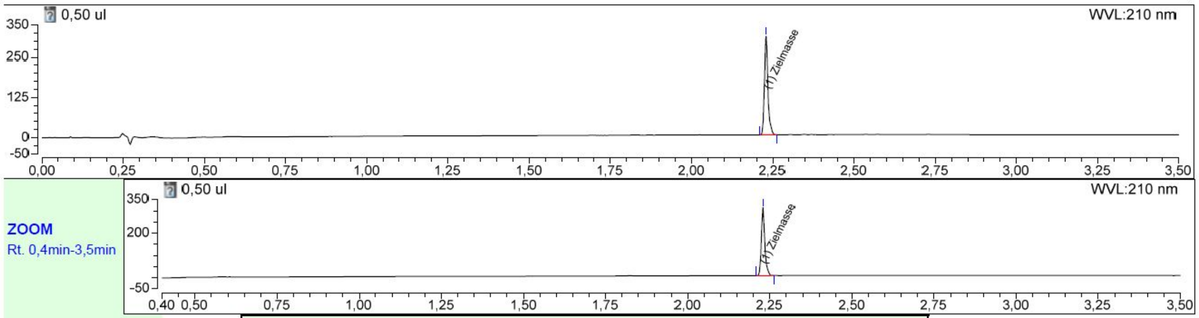

#	Spectrum	RT	Area%	Area %	Zielmasse gefunden	
					[M+H]+	[M-H]-
		min	210nm	UVMax	Pos	Neg
1	Y	2,23	100,0	100,0	520,1989	518,1838

LC/MS of compound **21** (BAY-805):

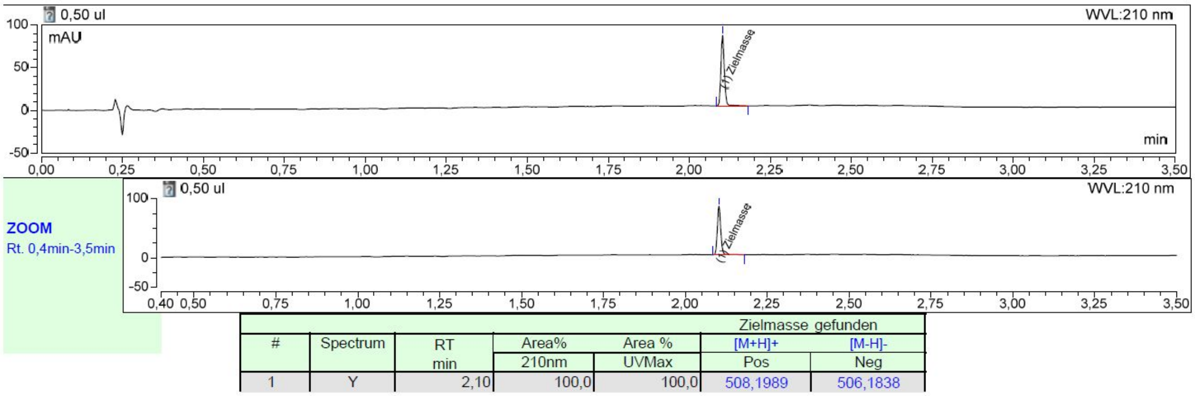

#	Spectrum	RT	Area%	Area %	Zielmasse gefunden	
					[M+H]+	[M-H]-
		min	210nm	UVMax	Pos	Neg
1	Y	2,10	100,0	100,0	508,1989	506,1838

LC/MS of compound **22** (BAY-728):

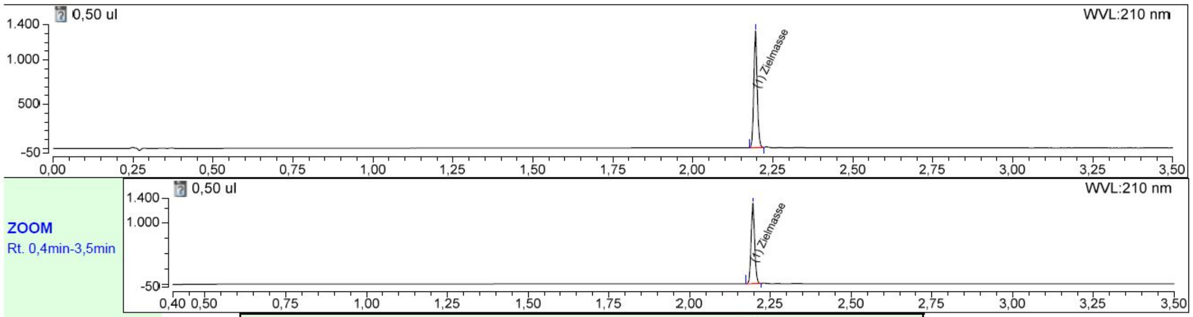

#	Spectrum	RT	Area%	Area %	Zielmasse gefunden	
					[M+H]+	[M-H]-
		min	210nm	UVMax	Pos	Neg
1	Y	2,20	100,0	99,1	508,1989	506,1838

LC/MS of compound **25**:

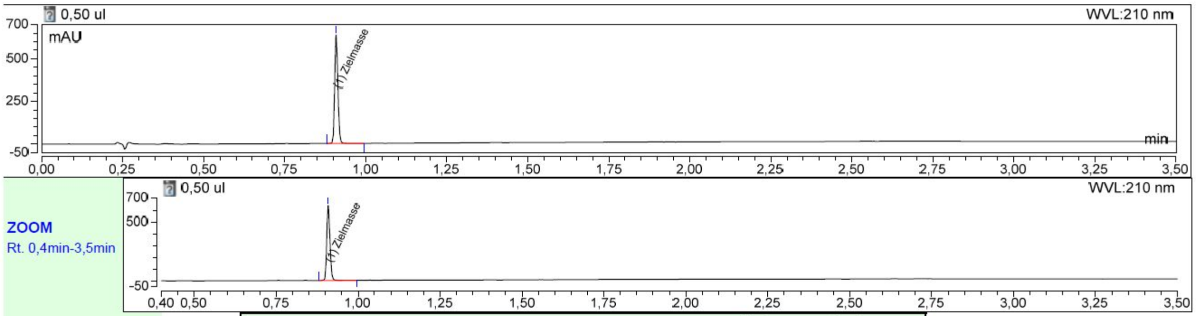

#	Spectrum	RT	Area%	Area %	Zielmasse gefunden	
					[M+H]+	[M-H]-
		min	210nm	UVMax	Pos	Neg
1	Y	0,91	100,0	100,0	217,0543	215,0392

LC/MS of compound **27**:

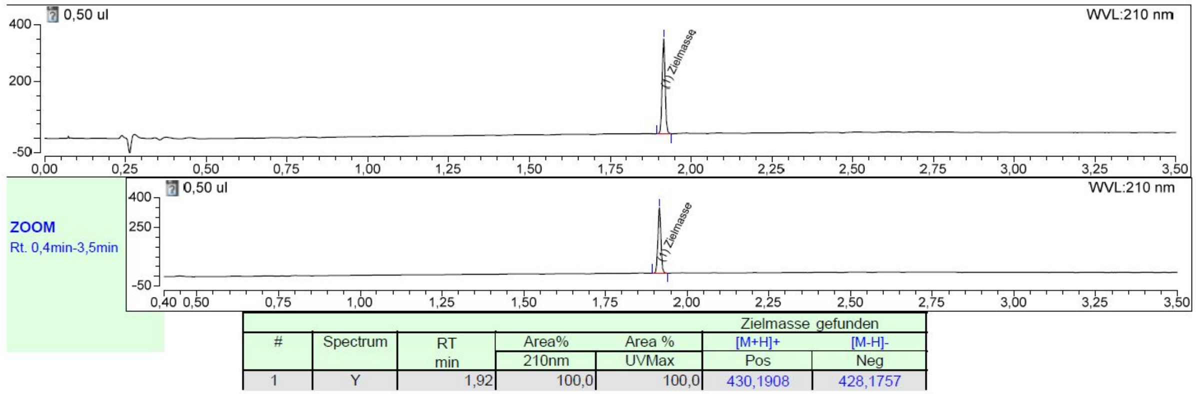

#	Spectrum	RT	Area%	Area %	Zielmasse gefunden	
					[M+H]+	[M-H]-
		min	210nm	UVMax	Pos	Neg
1	Y	1,92	100,0	100,0	430,1908	428,1757

LC/MS of compound **28**:

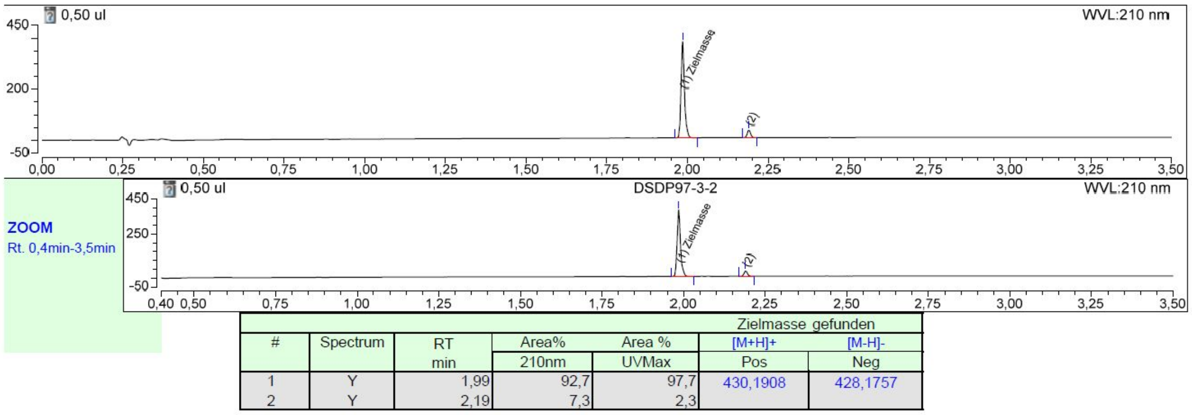

#	Spectrum	RT	Area%	Area %	Zielmasse gefunden	
					[M+H]+	[M-H]-
		min	210nm	UVMax	Pos	Neg
1	Y	1,99	92,7	97,7	430,1908	428,1757
2	Y	2,19	7,3	2,3		

LC/MS of compound **29**:

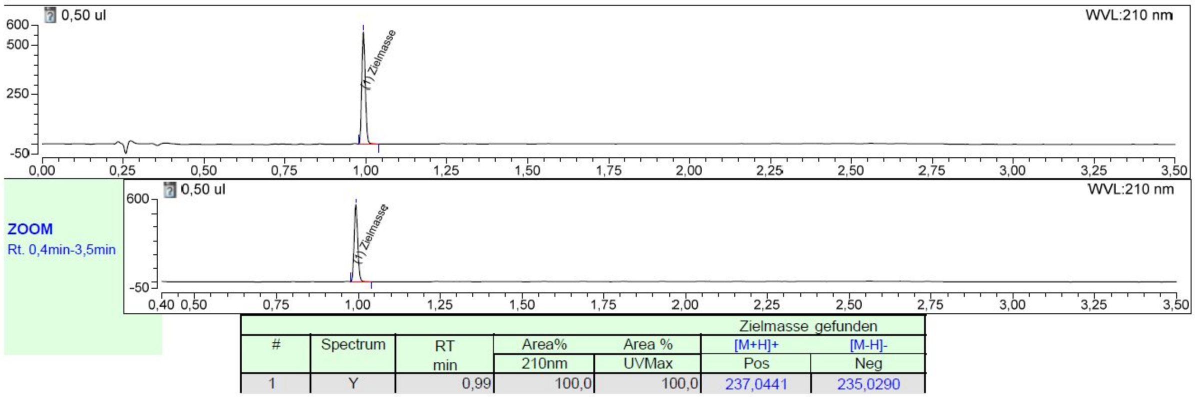

#	Spectrum	RT	Area%	Area %	Zielmasse gefunden	
					[M+H]+	[M-H]-
		min	210nm	UVMax	Pos	Neg
1	Y	0,99	100,0	100,0	237,0441	235,0290

LC/MS of compound **30**:

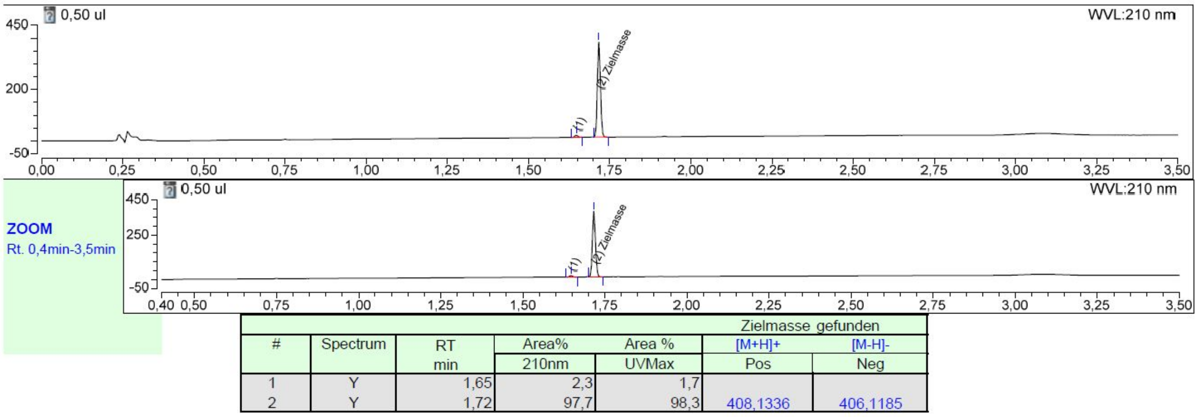

#	Spectrum	RT	Area%	Area %	Zielmasse gefunden	
					[M+H]+	[M-H]-
		min	210nm	UVMax	Pos	Neg
1	Y	1,65	2,3	1,7		
2	Y	1,72	97,7	98,3	408,1336	406,1185

LC/MS of compound **31**:

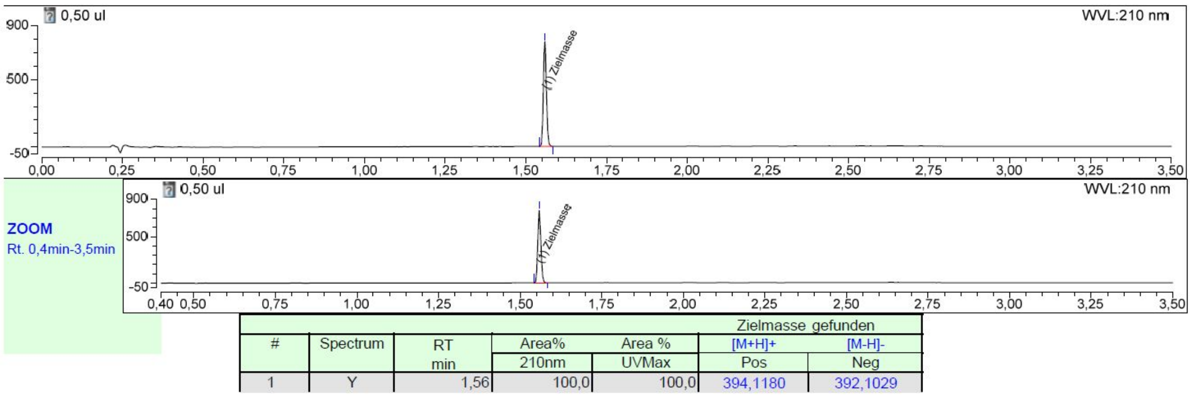

#	Spectrum	RT	Area%	Area %	Zielmasse gefunden	
					[M+H]+	[M-H]-
		min	210nm	UVMax	Pos	Neg
1	Y	1,56	100,0	100,0	394,1180	392,1029

LC/MS of compound **32**:

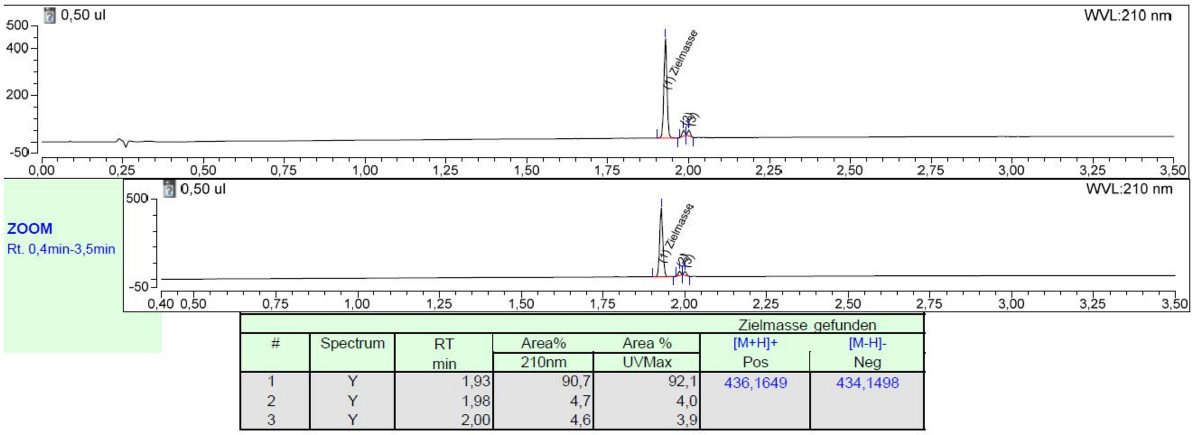

#	Spectrum	RT	Area%	Area %	Zielmasse gefunden	
					[M+H]+	[M-H]-
		min	210nm	UVMax	Pos	Neg
1	Y	1,93	90,7	92,1	436,1649	434,1498
2	Y	1,98	4,7	4,0		
3	Y	2,00	4,6	3,9		

LC/MS of compound **33**:

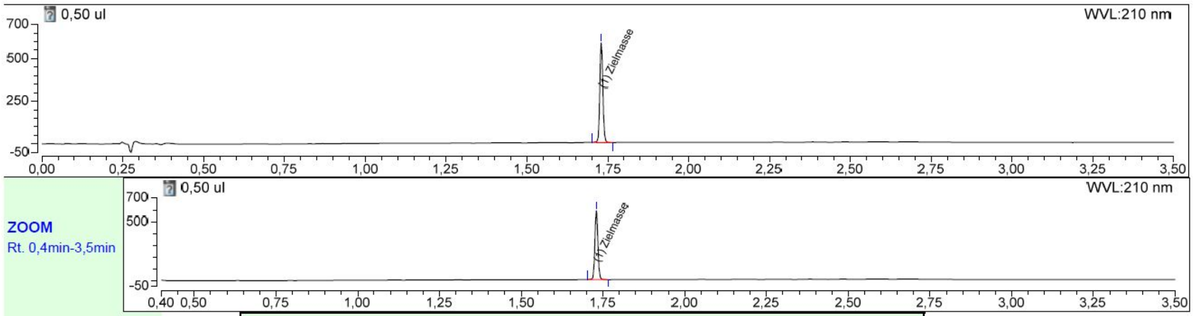

#	Spectrum	RT min	Area% 210nm	Area % UVMax	Zielmasse gefunden [M+H]+ Pos	[M-H]- Neg
1	Y	1,73	100,0	100,0	438,1442	436,1291

LC/MS of compound **34**:

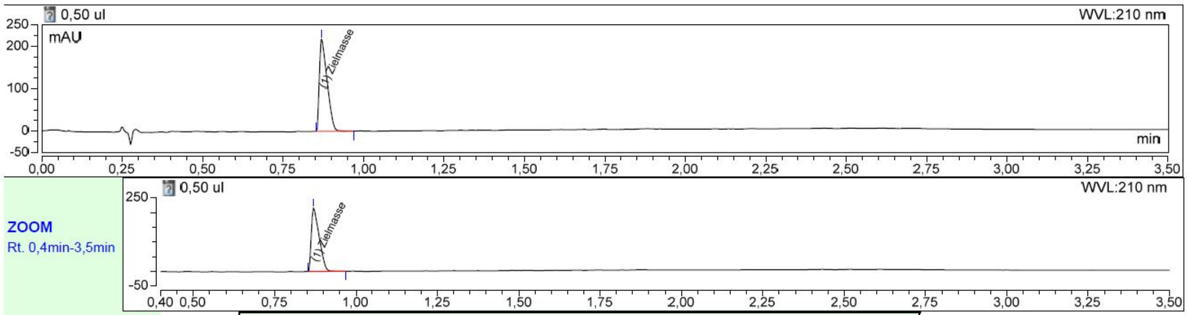

#	Spectrum	RT min	Area% 210nm	Area % UVMax	Zielmasse gefunden [M+H]+ Pos	[M-H]- Neg
1	Y	0,87	100,0	100,0	338,0918	336,0767

LC/MS of compound **35**:

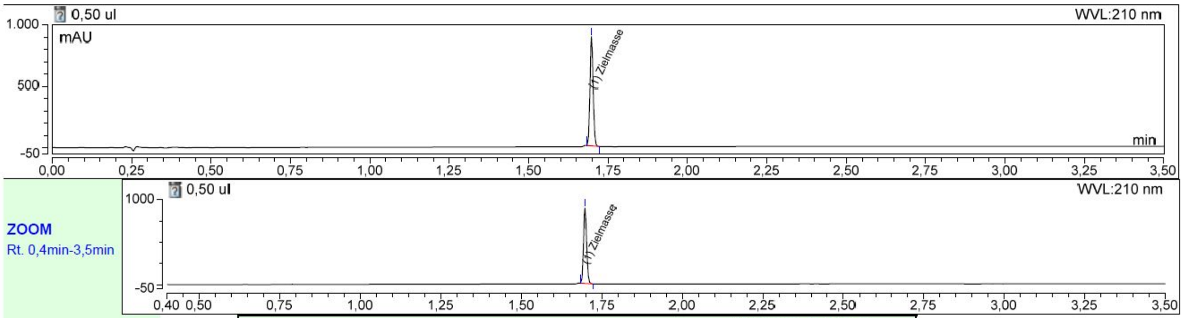

#	Spectrum	RT min	Area% 210nm	Area % UVMax	Zielmasse gefunden [M+H]+ Pos	[M-H]- Neg
1	Y	1,70	100,0	100,0	438,1442	436,1291

LC/MS of compound **36**:

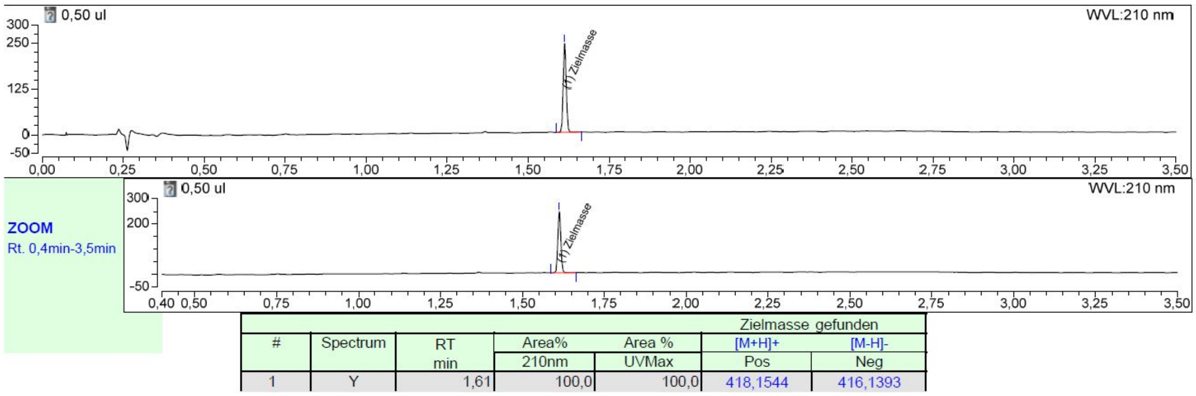

#	Spectrum	RT	Area%	Area %	Zielmasse gefunden	
					[M+H]+	[M-H]-
		min	210nm	UVMax	Pos	Neg
1	Y	1,61	100,0	100,0	418,1544	416,1393

LC/MS of compound **37**:

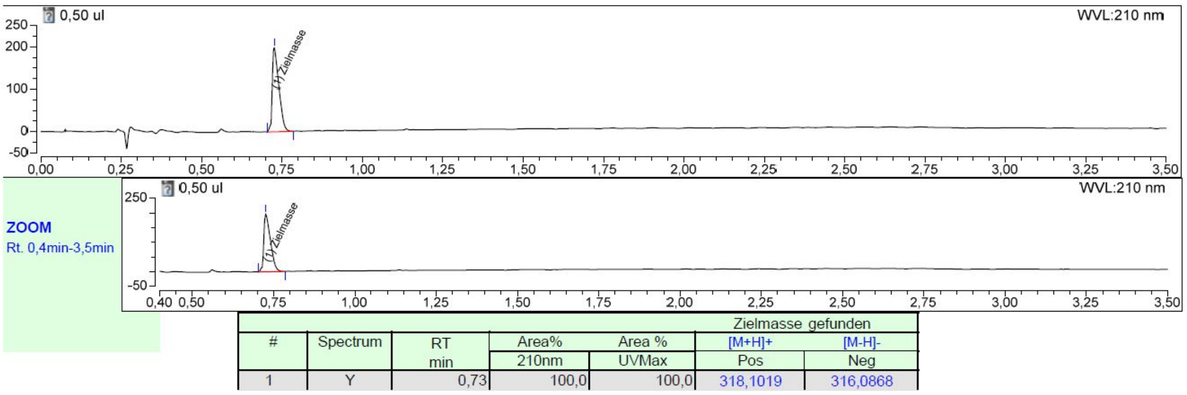

#	Spectrum	RT	Area%	Area %	Zielmasse gefunden	
					[M+H]+	[M-H]-
		min	210nm	UVMax	Pos	Neg
1	Y	0,73	100,0	100,0	318,1019	316,0868

LC/MS of compound **38**:

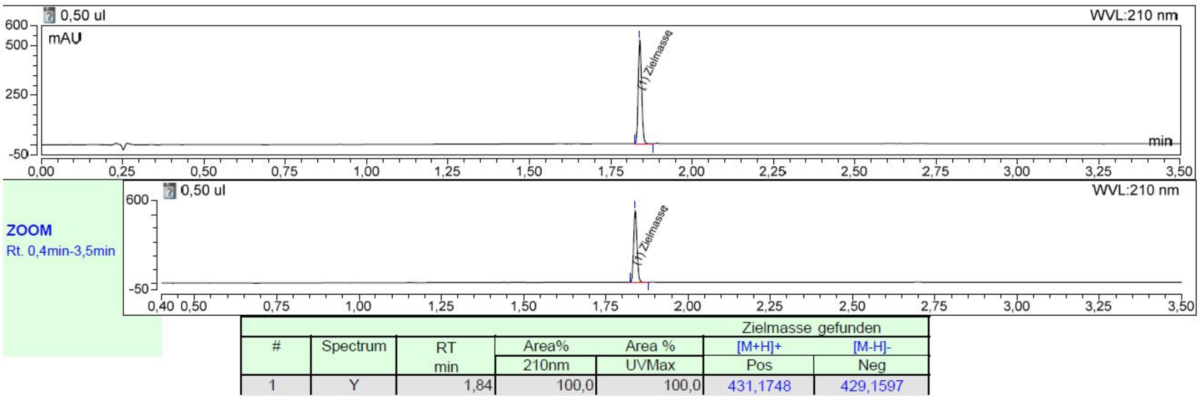

#	Spectrum	RT	Area%	Area %	Zielmasse gefunden	
					[M+H]+	[M-H]-
		min	210nm	UVMax	Pos	Neg
1	Y	1,84	100,0	100,0	431,1748	429,1597

LC/MS of compound **40**:

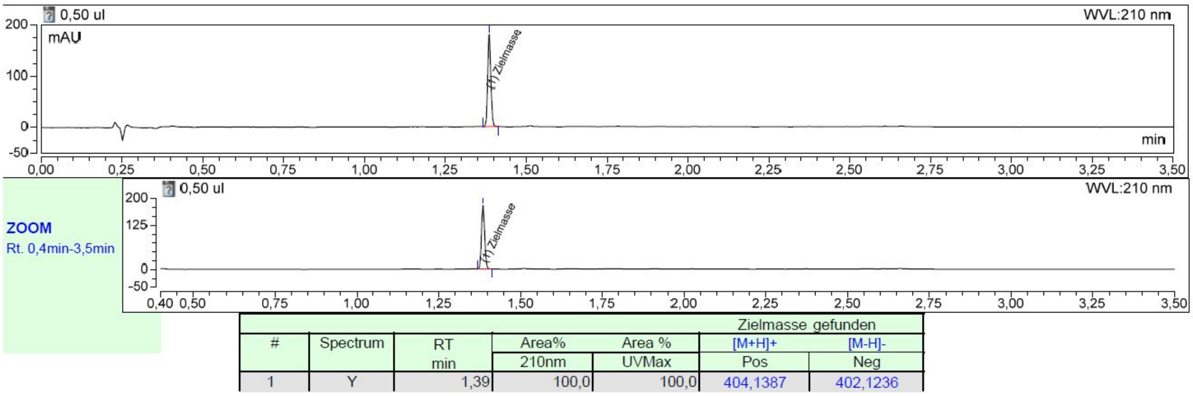

#	Spectrum	RT	Area%	Area %	Zielmasse gefunden	
					[M+H]+	[M-H]-
		min	210nm	UVMax	Pos	Neg
1	Y	1,39	100,0	100,0	404,1387	402,1236

LC/MS of compound **41**:

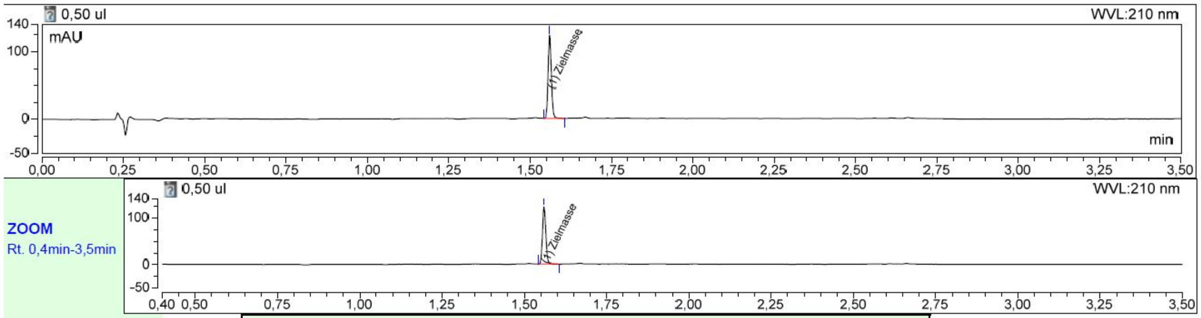

#	Spectrum	RT	Area%	Area %	Zielmasse gefunden	
					[M+H]+	[M-H]-
		min	210nm	UVMax	Pos	Neg
1	Y	1,56	100,0	100,0	432,1700	430,1549

LC/MS of compound **42**:

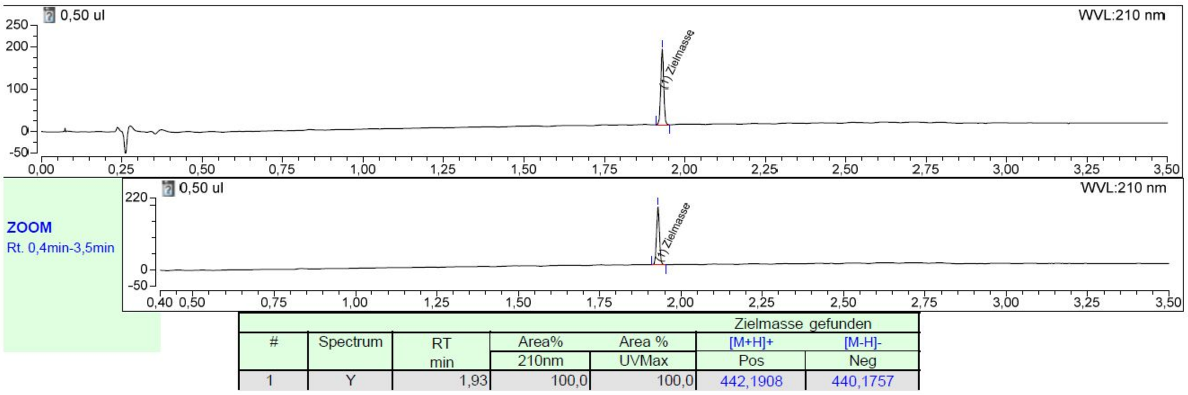

#	Spectrum	RT	Area%	Area %	Zielmasse gefunden	
					[M+H]+	[M-H]-
		min	210nm	UVMax	Pos	Neg
1	Y	1,93	100,0	100,0	442,1908	440,1757
